# RPL41 inhibits the proliferation and migration of retinoblastoma through the ARL5B-associated lysosomal trafficking

**DOI:** 10.3389/fimmu.2025.1704080

**Published:** 2025-12-10

**Authors:** Ye Li, Tian Zhan, Tianfu Chen, Qianwen Zhong, Sheng Xiao, Aiyuan Wang, Yisheng Jiao

**Affiliations:** 1 Department of Ophthalmology, Shengjing Hospital of China Medical University, Shengyang, Liaoning, China; 2 Department of Pathology, Brigham and Women’s Hospital of Harvard Medical School, Boston, MA, United States; 3 Department of Obstetrics and Gynecology, Shengjing Hospital of China Medical University, Shengyang, Liaoning, China

**Keywords:** ARL5B, ATF4, metastasis, retinoblastoma, RPL41, lysosomal function

## Abstract

**Purpose:**

Retinoblastoma is the most common intraocular cancer in infants and children, with a significant potential for metastasis. The mini-peptide ribosomal protein L41 (RPL41) has demonstrated extensive antitumor effects *in vitro* by promoting the degradation of activating transcription factor 4 (ATF4). This study aims to evaluate the therapeutic effect of RPL41 on retinoblastoma and elucidate its potential mechanisms.

**Methods:**

A xenografted retinoblastoma model was constructed in nude mice. The effects of xenografted RPL41 on tumor proliferation, invasion and metastasis were evaluated by local injection. Mass spectrometry identified differentially expressed genes in Y79 and Weri-RB1 retinoblastoma cells pre- and post-treatment. We utilized quantitative real-time PCR (qRT-PCR), Western blotting, and immunohistochemistry to assess the expression levels of ARL5B(ADP ribosylation factor like GTPase 5B) in retinoblastoma cell lines and tissues. We also explored ATF4’s regulatory role on ARL5B expression through chromatin immunoprecipitation (ChIP) experiments and luciferase reporter gene assays.

**Results:**

RPL41 inhibits the growth of subcutaneous retinoblastoma xenografts. ARL5B expression was significantly downregulated in treated Y79 and Weri-RB1 cells. ARL5B was upregulated in retinoblastoma cells and clinicopathological tissues. RPL41 treatment led to ATF4 degradation, reducing the expression levels of ARL5B and lysotransfer-related molecules. Knocking down ATF4 decreased ARL5B protein levels. ChIP experiments and dual-luciferase assays confirmed ATF4 positively regulates ARL5B. Rescue experiments indicated that ARL5B overexpression partially reversed the effects of RPL41 therapy or ATF4 knockdown on lysosomal pathways and cell migration.

**Conclusions:**

RPL41 down-regulates the expression of ARL5B by degrading ATF4 and the impaired ARL5B-related lysosomal trafficking is a mechanism to inhibit the metastasis of retinoblastoma.

## 1 Introduction

Retinoblastoma (RB) is the most common intraocular malignancy in children (1). Recent interventions such as chemotherapy (2), thermal therapy, and biomaterial treatment (3) have significantly improved survival rate. However, chemotherapeutic agents like carboplatin, vincristine, and etoposide can cause severe toxic reactions (4), including ototoxicity and nephrotoxicity, and may lead to drug resistance (5), particularly in the middle and late stages of metastatic tumors (6). Consequently, reducing the metastasis rate of retinoblastoma has become a critical issue, and drug resistance, along with adverse reactions, has garnered increased attention from researchers (7). In recent years, the significance of post-transcriptional regulation in proteomics within tumor biology has been increasingly recognized (8, 9), and investigations into oncogenes may provide insights into the mechanisms underlying metastasis and progression in retinoblastoma.

Activating transcription factor 4 (ATF4) is a crucial transcription factor that is activated by tumor cells in response to various cellular stress signals (10). It plays a significant role in tumor growth, invasion, and metastasis, as well as in tumor drug resistance (11, 12), by enhancing the adaptive protective response of cells (13). *ATF4* is over-expressed in many tumors including retinoblastoma (14), and is regarded as a candidate therapeutic target (15). Down-regulating *ATF4* expression through gene regulation methods can markedly reduce tumor growth and invasiveness, while also increasing tumor cell sensitivity to various chemotherapy (16). However, anti-*ATF4* strategy in cancer therapy has never been tested *in vivo* due to the lack of systematically usable anti-*ATF4* agents, the role of *ATF4* in retinoblastoma progression and metastasis remains poorly understood.

An early functional screening study showed that lung cancer cells were very sensitive to RPL41(ribosomal protein L41) treatment (17). RPL41 is a protein-coding gene that encodes a small peptide containing only 25 amino acids with an apparent molecular weight of approximately 3456 Da. Endogenous RPL41 is considered to be a member of the ribosome subunit, located primarily in the endoplasmic reticulum of cells (18). RPL41 is down-regulated or absent in many human tumors, and it is considered to be a promising tumor suppressor gene (19). The synthetic RPL41 has the same composition as endogenous mini-peptide, only 25 amino acids, and is rich in basic amino acids such as arginine and lysine (20). As a result, it is able to penetrate cell membranes on its own and is taken up by tumor tissue. The application of RPL41 as a therapeutic mini-peptide drug is attracting extensive interest from researchers.

Our previous studies indicated that synthetic RPL41 could induce cell cycle arrest and apoptosis of RB cells. *In vitro*, RPL41 treatment can effectively inhibit the proliferation, migration and invasion of retinoblastoma, and enhance the sensitivity of RB cells to carboplatin (20). In terms of mechanism, ATF4 is rapidly degraded by the proteasome system after treatment with RPL41, and the phosphorylation modification (site) of ATF4 is the prerequisite for degradation (20). The downregulation of ATF4 in RB cells is a key factor in tumor inhibition. However, whether the synthetic RPL41 peptide has similar effects *in vivo* and the underlying mechanisms are still poorly understood (21).

To explore the molecular details of the role of RPL41. We identified and analyzed differentially expressed genes after exogenous RPL41 treatment by 4D-Label-free quantitative proteomics. The results showed that the intervention of RPL41 affected protein homeostasis, and a considerable number of differentially expressed genes were associated with tumor progression. Among them, ARL5B(ADP ribosylation factor like GTPase 5B) was significantly down-regulated after treatment with RPL41. *ARL5B* is recognized as a member of the ADP ribosylation factor-like family and belongs to the RAS superfamily (22). Among the 30 members of the human Arf family, two paralogs of *ARL5B* (ARL5Ba and ARL5Bb) are currently known to be key members related to the function of lysosome *in vitro* expulsion (anterograde transport) (23). Importantly, during cancer invasion, cancer cells continuously adapt to the lysosomal system and its physiological processes to satisfy their intrinsic synthetic and catabolic requirements (24). Lysosomes fuse with the plasma membrane (PM) to expel soluble and granular contents from the cell, thereby altering the composition of the PM, acidifying the tumor microenvironment, and facilitating the efficient degradation of extracellular matrix (ECM) components (25). The impaired lysosomal function *in vitro* can inhibit the invasion of cancer cells (26). The anterograde transport of lysosomes involves coupling to the microtubule motor kinesin-1 via the small GTPase *ARL5B* and the adapter protein SKIP (sifa and kinesin-interacting protein, also known as PLEKHM2, Pleckstrin homology and run domain containing M2) (27). This process represents a centrifugal (outward) movement driven by ARL5B and mediated by the effector protein SKIP, which connects lysosomes to the plus-end-directed microtubule motor kinesin-1 (28). As an adaptor protein connecting lysosomes and kinesin-1, SKIP is involved in transportation processes such as lysosome positioning (29). Kinesin-1 contains a TPR(tetratricopeptide repeat) domain, and the fragment containing SKIP residues binds to the TPR domain of the kinesin light chain. This interaction is crucial for linking SKIP to the Kinesin-1 heavy chain, primarily referring to KIF5B(kinesin family member 5b) (30). However, *ARL5B* and SKIP exert their effects mainly through kinesin light chains, with the predominant non-neuronal form being KLC2(kinesin light chain 2) (31). The interaction between SKIP and KLC2 serves as the initial step. KLC2 recognizes the tryptophan-acidic (W-acidic) motif on SKIP through its TPR domain. This binding induces a conformational change in KLC2, thereby destabilizing the autoinhibited state of KIF5B and exposing a second SKIP binding site adjacent to the autoinhibitory IAK(isoleucine-alanine-lysine) region at the C-terminal tail of KIF5B. Subsequently, SKIP binds to this site, enhancing the interaction between the motor protein and its cargo, thereby facilitating transport (29, 32). Studies by Tal Keren-Kaplan and Juan S. Bonifacino demonstrated that *ARL5B* not only recruits SKIP to lysosomes but also promotes SKIP activation by disrupting its autoinhibitory state, thereby facilitating anterograde lysosomal transport driven by kinesin-1 (33). Consequently, *ARL5B* binding to SKIP establishes a link from the lysosomal membrane to extracellular movement, which also stimulates the bidirectional movement of lysosomes along microtubules (34). In the early stages, a multi-subunit complex located on the cytoplasmic surface of lysosomes regulates lysosome positioning. It recruits *ARL5B* to the lysosomal membrane, thereby interacting with the SKIP-Kinesin-1 complex and facilitating the movement of lysosomes along microtubules toward the cell periphery (35). This connection-dependent diffusion of lysosomes contributes to the spreading and movement of tumor cells (29, 36).

Our study demonstrates that *ATF4*, an important effector molecule downstream of RPL41, regulates the expression of ARL5B and activates the lysosomal trafficking mediated by it. This process subsequently contributes to the growth, invasion, and metastasis of retinoblastoma cells. RPL41 inhibits the lysosomal trafficking of the ARL5B/SKIP/Kinesin-1 signaling pathway by promoting the degradation of *ATF4*, thereby suppressing the growth and metastasis of retinoblastoma. In this study, we investigated the expression and function of ARL5B in retinoblastoma and further explored the underlying mechanisms, providing an experimental basis for the clinical application of RPL41 and identifying a new target for the treatment of retinoblastoma.

## 2 Materials and methods

### 2.1 Tissue samples

A total of 20 retinoblastoma tissue specimens were collected between 1997 and 2024 at Shengjing Hospital, affiliated with China Medical University in Shenyang, China. Clinical specimens from patients who had received any treatment prior to surgery were excluded from the study, and all diagnoses were confirmed by pathologists from the same team in the Department of Pathology of Shengjing Hospital Affiliated to China Medical University. Both primary retinoblastoma tissues and adjacent normal retinal tissues were utilized to analyze the levels of ARL5B/SKIP(PLEKHM2)/KIF5B/KLC2. The experimental protocol received approval from the Ethics Committee of Shengjing Hospital, affiliated with China Medical University(Approval number: 2025PS034K).

### 2.2 Cell culture and treatment

The Human retinoblastoma cell lines, Y79 and Weri-RB1, were respectively purchased from Shanghai Jihe Biotechnology Co., Ltd. (Shanghai, China) and Wuhan Procell Life Technology Co., Ltd. (Wuhan, China). The authenticity of all cell lines was confirmed by short tandem repeat (STR) DNA profiling analysis. All cells were cultured in Roswell Park Memorial Institute 1640 (RPMI-1640) medium (Keygen, Jiangsu, Chian), supplemented with 10% fetal bovine serum (FBS; Procell, China) and 1% penicillin-streptomycin-glutamine (100X; Gibco), at 37°C and 5% CO_2_. During the experiments, cells were exposed to an equal volume of the control or RPL41 peptide (40 μM in double-distilled water) for 24hr.

### 2.3 RPL41 synthesis

The RPL41 peptide (NH2-MRAKWRKKRMRRLKRKRRKMRQRSKOH) was synthesized by GenScript (Nanjing, China), following the methodology outlined in a previous study (20). The purity of the peptide was assessed to be greater than 95% using high-performance liquid chromatography (HPLC), and it was further characterized by mass spectrometry and HPLC analysis. The peptide was then reconstituted in double-distilled water to achieve a final concentration of 10 mM.

### 2.4 Quantitative reverse transcription-PCR

Total RNA was extracted from cells utilizing the Trizol reagent, followed by cDNA synthesis with the PrimeScript RT reagent kit (Takara, Kusatsu, Japan). Quantitative Real-Time PCR (qRT-PCR) was performed using SYBR-Green Master Mix (Takara) on a 7500 Fast Real-time system (ABI, USA). β-Actin was employed as the endogenous control for mRNA quantification, and fold changes were calculated using the relative quantification method (2^−ΔΔCt^). Relevant primer sequences were designed and procured from Sangon Bioengineering Co., LTD (Shanghai, China). The following primer sequences were used:

ARL5B forward: 5’- GTGGGATATTGGTGGTCAGGAGTC -3’; ARL5B reverse: 5’- AGCTAGTCGTTCCCTGTCAATGC -3’; ATF4 forward: 5’- TCCTGTCCTCCACTCCAGATCATTC -3’; ATF4 reverse: 5’- TCATGGCAACGTAAGCAGTGTAGTC -3’; PLEKHM2 forward: 5’- GAGGAGGAGGAGAGGGACAGAC -3’; PLEKHM2 reverse: 5’- TCGCTTGAGCTGGCAGAGAAC-3’; KIF5B forward: 5’- TGTGTCGCTTCAGACCTCTCAAC -3’; KIF5B reverse:5’- GACTGGAACACCCGATCAAATGC -3’; KLC2 forward: 5’- CGTGGCTGCGACACTAAACAAC -3’; KLC2 reverse:5’- TGAAACTTGCCCAGGACCTTCTC-3’; β-actin forward: 5’-TATGCTCTCCCTCACGCCATCC -3’; β-actin reverse: 5’-GTCACGCACGATTTCCCTCTCAG -3’.

### 2.5 Western blotting

Briefly, Y79 and Weri-RB1 cells were harvested and lysed following transfection or treatment with the small molecule peptide. Protein concentration was quantified utilizing the BCA protein concentration assay kit (Epizyme, Shanghai, China). Equal amounts of protein were separated by 10%, 12.5% and 15% sodium dodecyl sulfate-polyacrylamide gel electrophoresis (SDS-PAGE) and transferred onto polyvinylidene difluoride (PVDF) membranes (Millipore, USA). Membranes were incubated with primary antibodies at 4°C overnight and then secondary antibodies at room temperature for 2h. After washing, signals were detected using a Tanon 5200 Luminous imaging system(Tanon, Shanghai, China). The following antibodies were purchased: anti-ARL5B (1:2000, Thermo, USA), anti-ATF4 (1:1000, Huabio, Hangzhou, Zhejiang, China), anti-PLEKHM2(1:2000, Thermo, USA), anti-KIF5B(1:7000, Proteintech, Wuhan, Hubei, China), anti-KLC2(1:2500, Proteintech, Wuhan, Hubei, China) and anti-β-actin (1:5000, Proteintech, Wuhan, Hubei, China) and secondary antibodies anti-rabbit IgG (1:3000, Bioss, Beijing, China) and anti-mouse IgG (1:3000, Proteintech, Wuhan, Hubei, China).

### 2.6 RNA transfection

Small interfering RNAs (siRNAs) designed for ATF4 (si-ATF4) and negative control siRNA (si-Ctrl) were obtained from RiboBio Co., Ltd. (Guangzhou, Guangdong, China). Transfection was conducted using Lipofectamine 3000 (Invitrogen, Thermo, USA) according to the manufacturer’s instructions.

### 2.7 Generation of overexpressing cell lines

Lentivirus overexpressing ARL5B or containing a control vector was obtained from HanBio (Shanghai, China). Y79 and Weri-RB1 cells were cultured in 6-well plates. Upon reaching 70% confluence, the medium was supplemented with lentiviruses and polybrene (10 μg/ml; Hanbio) at a multiplicity of infection (MOI) of 20, followed by mixing with the cells. Polybrene was utilized to enhance infection efficiency. After an incubation period of 24h, the supernatants in the wells were replaced with DMEM containing FBS and further cultured for an additional 48h for subsequent analyses.

### 2.8 Luciferase reporter assay

The DNA fragments containing either the wild-type or mutant ARL5B promoter were inserted into the pGL3-basic vector(Hanbio, Shanghai, China), respectively. Using the Lipofiter transfection reagent(Hanbio, Shanghai, China), 1.2 µg of the target plasmid (PRL: 0.2 µg, promoter: 0.4 µg, transcription factor: 0.6 µg) was transfected into 293T cells. Following transfection, the cells were cultured for an additional 48 hours before sample collection. After lysing the cells, a dual-luciferase assay was performed, and a microplate reader was used to measure both the Firefly luciferase and Renilla luciferase values to evaluate the binding of ATF4 to ARL5B.

### 2.9 Transwell assay

The migration of retinoblastoma Y79 and Weri-RB1 cells was evaluated using a transwell assay with an insert chamber featuring an 8-μm pore size (Corning, NY, USA). Briefly, after appropriate processing, equal quantities of Y79 and Weri-RB1 cells were processed and suspended in 200 μl of RPMI-1640 medium containing 1% FBS, which were then seeded into the upper chambers. In the lower chambers, 300 μl of RPMI-1640 medium supplemented with 20% FBS was added. Following a 24-hour incubation at 37°C, the cells in the lower chambers were collected. Viable cells were assessed using trypan blue exclusion dye and counted with an automatic cell counter (RWD, Shenzhen, China), ensuring that any observed reduction in cell migration was not due to cell death.

### 2.10 *In vivo* tumor xenograft models

A total of 12 male Balb/c nude mice (weight: 16–18 g; age: 4–5 weeks) were obtained from Beijing Huafu Kang Biotechnology Co., Ltd. (Beijing, China). All nude mice were housed in a specific-pathogen-free animal facility maintained at 60-65% humidity and a temperature of 22-25°C under a 12-h light/dark cycle. The experiments commenced after the mice had free access to food and water for one week. The nude mice were randomly assigned to two groups, with five mice in each group: the Blank group and the RPL41 group. A xenograft tumor model was established by subcutaneously injecting between 10^6^ and 10^7^ cells (suspended in 100 μl of PBS) into the flanks of the nude mice. In the RPL41 group, following successful tumor formation, an injection of 15–20 mg/kg RPL41 was administered daily on the tumor side. Tumor size was recorded every seven days, and images of each tumor were captured after four weeks. At the conclusion of the experiment, all mice were euthanized, and then the tumors were harvested for weight measurement and other analyses(For intraperitoneal administration of sodium pentobarbital at a dose of 150 mg/kg body weight, confirmation of absence of respiration and lack of reflex response is required to ensure euthanasia has been successfully achieved). The tumor volume (mm^3^) was calculated with the formula (0.5×length×width^2^). Animal study was approved by the research Ethics Committee of Shengjing Hospital affiliated to China Medical University (Approval number: 2024PS1604K).

### 2.11 Immunohistochemistry

Fresh tumor tissues from nude mice were fixed in 4% paraformaldehyde for 24 hours, followed by dehydration using ethanol solutions and embedding in paraffin. Subsequently, the paraffin-embedded tissue was sectioned into 4-μm thick slides. The sections were then washed, blocked, and incubated overnight at 4°C with primary antibodies against Ki67(diluted 1:100; Cell Signaling Technology, CST, USA), ARL5B (diluted 1:150; Thermo, USA) and ATF4 (diluted 1:100; Huabio, Hangzhou, Zhejiang, China). After thorough washing, the sections were incubated for 30 minutes at room temperature with an appropriate secondary antibody (diluted 1:150; Proteintech, Wuhan, Hubei, China). The sections were counterstained with hematoxylin, dehydrated, and subsequently sealed with neutral gum. Images were captured using computerized image acquisition software (NIS‐Elements F3.0) and an Olympus microscope (Nikon E800).

### 2.12 Chromatin immunoprecipitation quantitative PCR assays

The ChIP-qPCR experiment was performed by Seqhealth Technology Co., Ltd., Wuhan, China (Wuhan, China). The detailed procedures were as follows: Approximately 2×10^7^ 293T cells were harvested, washed with phosphate-buffered saline (PBS), and cross-linked with 1% formaldehyde at room temperature for 10 minutes. Cross-linking was terminated by the addition of 0.125 M glycine and incubation for 5 minutes. Subsequently, cells were collected and washed twice with PBS. The cell pellet was lysed on ice for 5 minutes using a lysis buffer containing 10 mM HEPES (pH 7.5), 0.1 mM EDTA, 0.5% NP-40, and a protease inhibitor cocktail. The nuclei were pelleted by centrifugation at 2000×g for 10 minutes at 4°C. Chromatin was then fragmented by ultrasonication to an average size of 100–500 base pairs (bp). A 10% aliquot of the sonicated chromatin was retained as the “input” control. Eighty percent of the sample was subjected to immunoprecipitation (IP) using an anti-ATF4 antibody (Huabio, Hangzhou, Zhejiang, China), while the remaining 10% was incubated with rabbit IgG (Abcam, Shanghai, China) as a negative control (“IgG”). Following immunoprecipitation, the complexes were washed, eluted, and de-crosslinked. DNA was extracted from both the input and IP samples using the phenol-chloroform method and quantified using a Qubit 3 fluorometer (Thermo, USA). The quality of chromatin fragmentation was assessed by agarose gel electrophoresis of the input DNA, and the success of immunoprecipitation was confirmed by Western blot analysis. Finally, qPCR was carried out using the extracted DNA as a template. The primer sequence used for detecting the ATF4 binding site within the ARL5B enhancer region is as follows:

Site 1 forward: 5’- AGGAGGTTGAAACTTGCCTT -3’;
Site 1 reverse: 5’- GACTTACGTCCTCCCAGTGC -3’;
Site 2 forward: 5’- CAATTCTCCTGCCTCAGCCT -3’;
Site 2 reverse: 5’- GGGTGGATCACGAAGTCAAGA -3’;
Site 3 forward: 5’- AGGAGGTTGAAACTTGCCTT -3’;
Site 3 reverse: 5’- GACTTACGTCCTCCCAGTGC -3’;

### 2.13 Mass spectrometry analysis

First, protein extraction and peptide enzymatic hydrolysis were performed. Protein extraction was carried out using the SDT lysis method (4% (w/v) SDS, 100 mM Tris/HCl pH 7.6, 0.1 M DTT). Subsequently, protein quantification was conducted using the BCA method. For each experimental group, 3 independent biological replicates were designed to account for inter-batch variability. An appropriate amount of protein from each sample was enzymatically hydrolyzed with trypsin via the Filter-Aided Sample Preparation (FASP) method. The resulting peptide segments were desalted using C18 Cartridges. Following freeze-drying of the peptide segments, they were re-dissolved in 40 μL of a 0.1% formic acid solution. Peptide quantification was performed at OD280. Secondly, LC-MS/MS data acquisition was performed. Each sample was separated using a nanoflow HPLC system (Easy nLC). After chromatographic separation, the samples were analyzed by mass spectrometry using a timsTOF Pro mass spectrometer. Both MS and MS/MS analyses were performed using Time-of-Flight (TOF) detection. The mass spectrometry scanning range was set to 100–1700 m/z. Data acquisition was performed in Parallel Accumulation Serial Fragmentation (PASEF) mode. Finally, protein identification and quantitative analysis were conducted. The raw mass spectrometry data were processed using MaxQuant software (version 1.6.14) for database searching, protein identification, and quantification. To ensure the reliability of the results, strict false discovery rate (FDR) control was applied: both peptide-level FDR and protein-level FDR were set to < 0.01 using the target-decoy database strategy. Additionally, data normalization was uniformly performed by professional technicians from Shanghai Applied Protein Technology Co., Ltd. following standard proteomic data processing protocols, which included missing value imputation, intensity correction, and batch effect elimination to ensure data accuracy and comparability across all samples. All reagents and technical support were provided by Shanghai Applied Protein Technology Co., Ltd.

### 2.14 Statistical analysis

All experimental results in this study were statistically analyzed using GraphPad Prism 9.0 software. Prior to conducting parametric statistical analyses, the Shapiro–Wilk test was employed to assess the normality of data distributions. The calculated data were expressed as mean ± standard deviation from at least three independent experiments, where ‘n’ denotes the number of animals and the number of independent experiments. Statistical differences between two or more experimental groups were assessed using either Student’s t-test, one-way analysis of variance (one-way ANOVA) or Tukey’s Multiple Comparison Test. In cases where the sample size was small or the data distribution deviates from the normal state, non-parametric tests were adopted. *P* < 0.05 indicated that the differences between treatment groups were considered statistically significant (*P < 0.05; **P < 0.01; ***P < 0.001; ****P < 0.0001).

## 3 Results

### 3.1 RPL41 inhibits the proliferation of retinoblastoma *in vivo*

**Figure 1:**
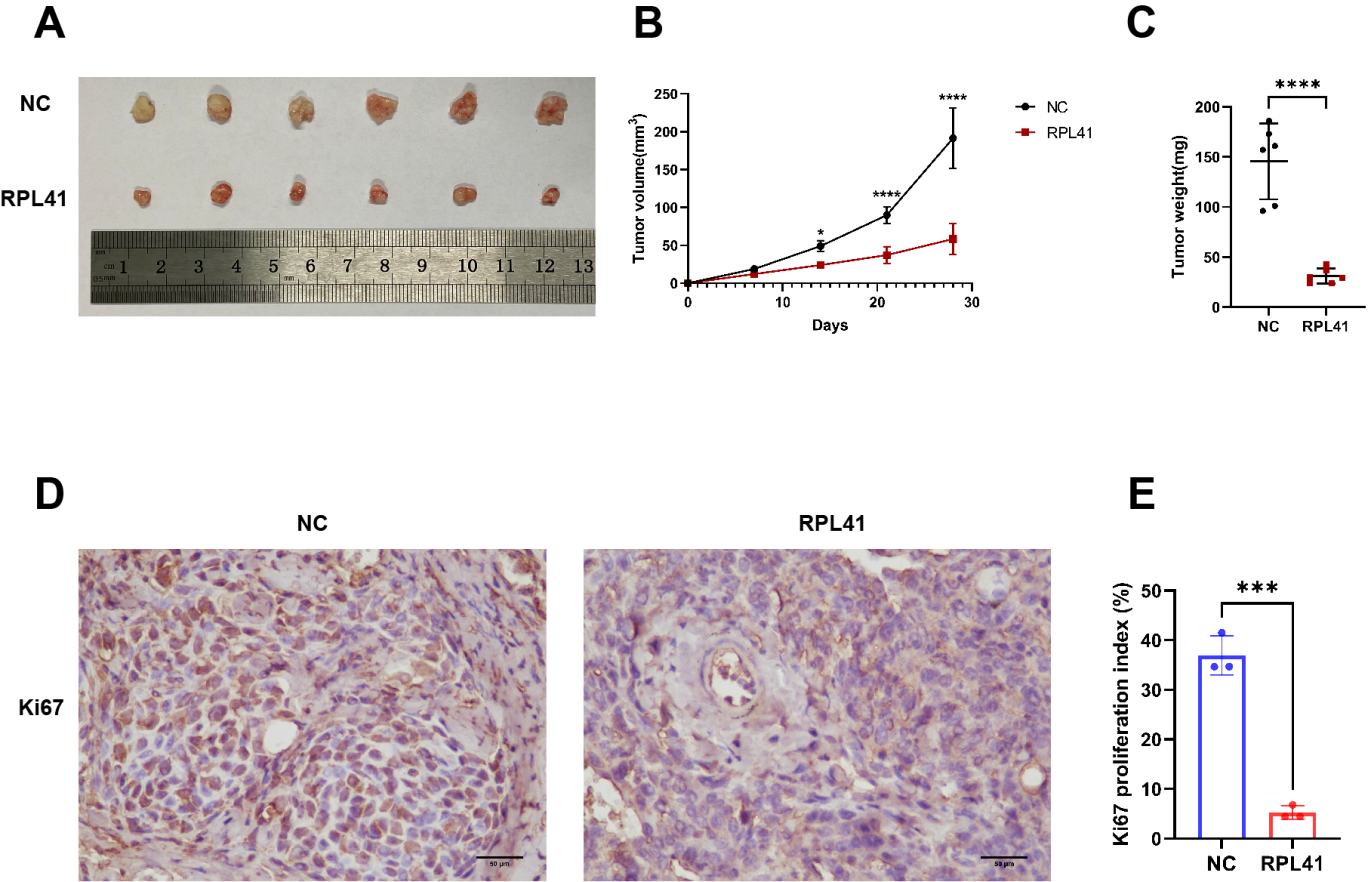
Mini peptide RPL 41 inhibits retinoblastoma proliferation of ectopic RB nude mice. **(A)** Representative images of xenograft tumor formation. (n=6, concentration of RPL41: 80 μM). n=3, Student’s t-test. **(B)** Quantitative map of tumor volume, abscissa represents sample collection time. **(C)** Quantitative analysis of tumor weight. n=3, Student’s t-test. **(D-E)** Representative images and statistical maps of immunohistochemical staining. Ki67 as a marker of tumor proliferation. Image analysis software separated cells from the background, distinguished individual cells, and localized cell nuclei. Based on preset color thresholds, it identified Ki67-positive cells (tan/brown-yellow nuclei), counted positive and total cells, and calculated the proliferation index (Proliferation Index = [Ki67+ cells/total cells] × 100%). Three fields of view were analyzed, and the average value was used for accuracy. NC group, the blank control group. RPL41 group, RPL41 treated the tumor group. Data are presented as mean ± SD, n=3. Student’s t-test. Scale bar: 50 μm, *P < 0.05, ***P < 0.001, ****P < 0.0001.

Our previous research demonstrated that RPL41 inhibits the growth, migration, and invasion of retinoblastoma cells (21). To further investigate whether RPL41 can regulate the growth of RB *in vivo*, we established an ectopic RB nude mouse model and administered the peptide adjacent to the tumor on the third day following successful model establishment. The results indicated that tumors in the negative control (NC) group gradually increased in size and weight; however, treatment with RPL41 resulted in a reduction in tumor size and a slowed growth rate (**Figures 1A–C**). Consistently, treatment with RPL41 inhibited the proliferation of retinoblastoma (**Figures 1D, E**).

### 3.2 Identification of differentially expressed genes in response to RPL41 treatment

**Figure 2:**
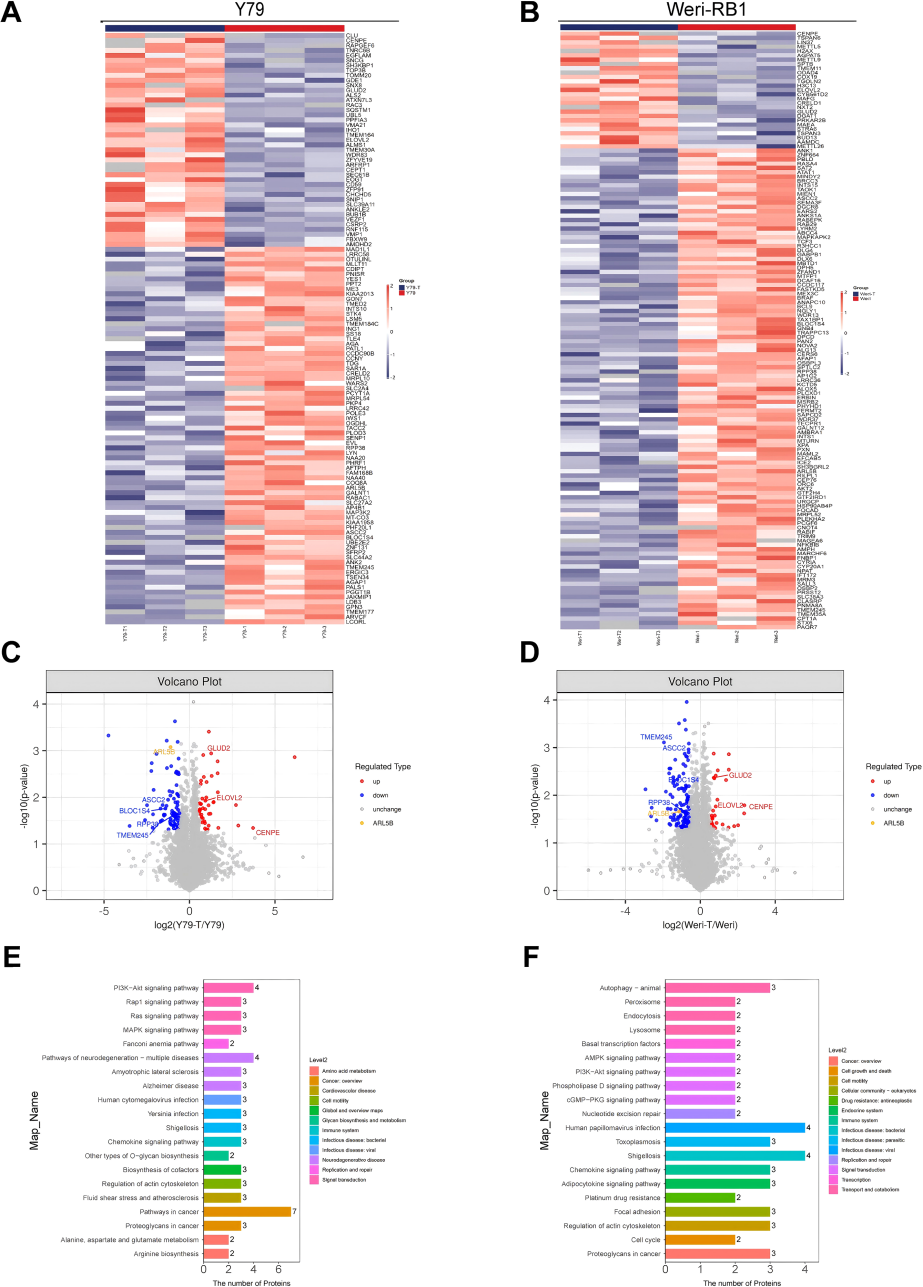
Differential gene expression and functional enrichment analysis following mini-peptide RPL41 treatment. **(A, B)** Heatmap illustrating the differences in protein expression between Y79 **(A)** and Weri-RB1 **(B)** cells treated with mini-peptide RPL41, compared to negative control cells, where blue indicates downregulation and red indicates upregulation. The annotations on the right side indicate the groups, n=3. **(C, D)** Volcano plots depicting the differentially expressed genes in Y79 **(C)** and Weri-RB1 **(D)** cells before and after treatment with mini-peptide RPL41, with blue representing downregulated proteins, red representing upregulated proteins, and yellow highlighting the protein of interest, ARL5B, n=3. **(E, F)** Pathway enrichment analysis of protein target genes in Y79 **(E)** and Weri-RB1 **(F)** cells treated with mini-peptide RPL41, where the numbers indicate the count of target genes enriched in each pathway, and Level 2 denotes the pathway name. Y79 group vs Y79-T group, Weri-RB1 group vs Weri-RB1-T group. Y79 and Weri-RB1 groups: untreated Y79 and Weri-RB1 cells, Y79-T and Weri-RB1-T groups: Y79 and Weri-RB1 cells treated with mini-peptide RPL41, n=3.

To elucidate the mechanism by which RPL41 inhibits retinoblastoma, we conducted mass spectrometry analysis using the retinoblastoma Y79 and Weri-RB1 cells. Following treatment with RPL41, we employed label-free mass spectrometry for quantification and utilized MaxQuant software to collect and analyze LC-MS/MS data. A comparative analysis of proteomics and bioinformatics was performed before and after RPL41 treatment. In the retinoblastoma Y79 cells, we observed that 81 genes were significantly down-regulated while 43 genes were significantly up-regulated (**Figure 2A**). In the retinoblastoma Weri-RB1 cells, 111 genes were significantly down-regulated and 27 genes were significantly up-regulated (**Figure 2B**). Commonly up- or down-regulated genes across both cells were presented in **Figures 2C, D**. Furthermore, the KEGG pathway enrichment analysis of these target genes indicated that cancer pathways were intensely clustered, followed by pathway in neurodegeneration-multiple diseases and PI3K-Akt signaling pathway. Additionally, lysosome-related target genes were found to be significantly enriched (**Figures 2E, F**). In both cell lines, the most significantly downregulated gene, ARL5B, along with its downstream lysosomal trafficking, was believed to be closely related to the proliferation of various tumors, including ovarian and breast cancers (37, 38). Therefore, we hypothesized that RPL41 may influence the growth of retinoblastoma cells by downregulating ARL5B. Based on this, we selected the ARL5B gene as the focus of subsequent research to further explore the therapeutic mechanisms of RPL41 in retinoblastoma.

### 3.3 ARL5B and downstream lysosomal trafficking related genes are highly expressed in retinoblastoma

**Figure 3:**
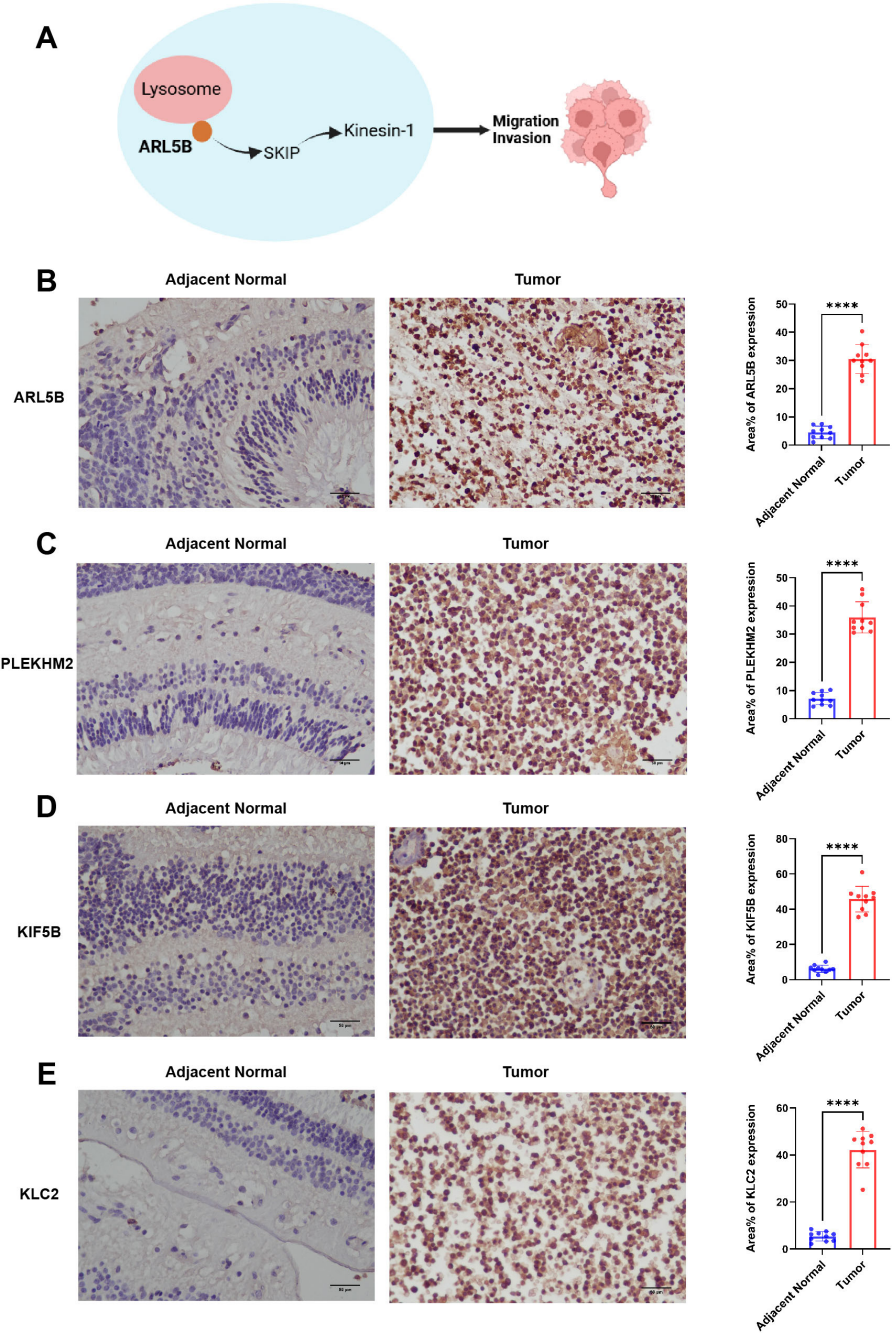
ARL5B and genes associated with the lysosomal diffusion pathway exhibit elevated expression levels in retinoblastoma. **(A)** A schematic diagram depicting the relationship between ARL5B and other genes associated with the lysosomal diffusion pathway: ARL5B promotes the movement of Kinesin-1 by recruiting SKIP, which drives lysosomes to move extracellularly, thereby facilitating tumor cell migration and invasion. **(B-E)** Representative immunohistochemistry (IHC) images and statistical plots illustrating the expression of ARL5B, SKIP (also known as PLEKHM2), KIF5B, and KLC2 in tumor tissues and adjacent normal retinal tissues(Number of cases=20). Three fields of view were analyzed, and the average value was used for accuracy. Data are presented as mean ± SD, n=3. Student’s t-test. Scale bar: 50 μm, ****P < 0.0001.

Genes associated with ARL5B and lysosome transport pathways *in vitro* are closely linked to tumor invasion (**Figure 3A**). We initially investigated the expression levels of ARL5B in retinoblastoma tissues. Immunohistochemical analysis revealed a marked increase in ARL5B expression in these tissues compared to adjacent normal retinal tissues (**Figure 3B**). Furthermore, SKIP (PLEKHM2), KIF5B and KLC2 were also significantly upregulated (**Figures 3C–E**). These findings indicated that the expression of ARL5B and lysosome transport-related genes was highly active in retinoblastoma, suggesting that it may facilitate the lysosomal expulsion pathway *in vitro*, thereby promoting tumor invasion and metastasis.

### 3.4 RPL41 treatment decreased the expression of ARL5B and genes associated with lysosome diffusion pathway

**Figure 4:**
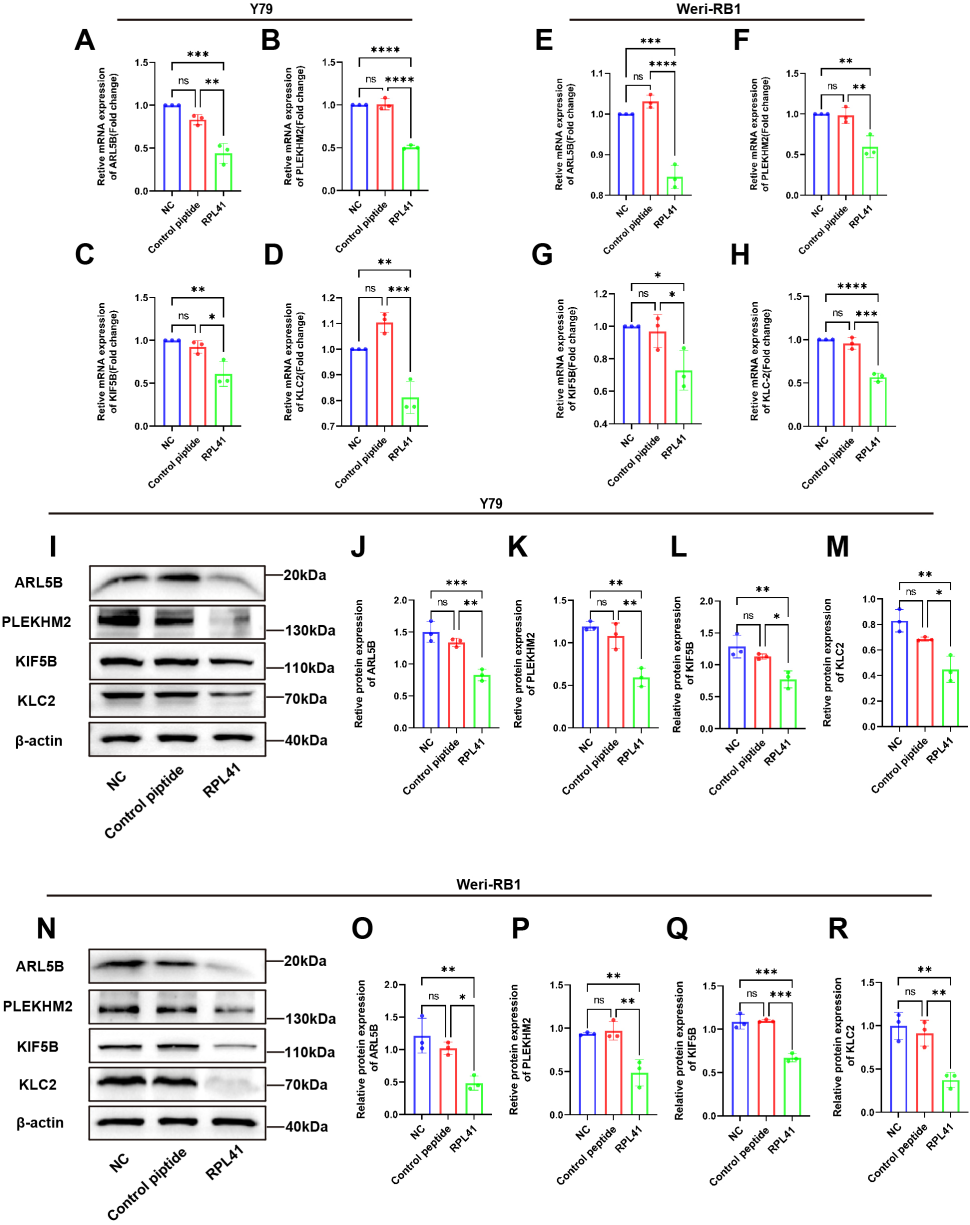
Mini-peptide RPL41 inhibits ARL5B gene and lysosomal diffusion pathway in Y79 and Weri-RB1 cells. **(A-H)** Quantitative analysis of mRNA levels of ARL5B, PLEKHM2, KIF5B and KLC2 in **(A-D)** Y79 and **(E-H)** Weri-RB1 cells in each group. n=3, ANOVA test. **(I-R)** Protein expression levels of ARL5B, PLEKHM2, KIF5B and KLC2 in **(I-M)** Y79 and **(N-R)** Weri-RB1 cells in each group. Data are presented as mean ± SD, n=3. ANOVA test, NC group vs Control peptide group, NC group vs RPL41 group, Control peptide group vs RPL41 group, *P < 0.05; **P < 0.01; ***P < 0.001; ****P < 0.0001. NC group, normal culture medium for 24h; Control peptide group, complete culture medium containing control peptide for 24h; RPL41 group, complete culture medium containing mini-peptide RPL41 was cultured for 24h.

To further validate the results of our proteomics study and investigate the effects of the RPL41 on ARL5B and its associated functions, we treated the Y79 and Weri-RB1 cell lines with RPL41. qRT-PCR analysis indicated that compared to the NC group and the control peptide group, the mRNA levels of ARL5B, SKIP, KIF5B, and KLC2 in Y79 and Weri-RB1 cells treated with RPL41 were significantly reduced, whereas no significant differences were observed in the mRNA expression levels between the NC group and the control peptide group (**Figures 4A–H**). Concurrently, Western blot analysis revealed that treatment with RPL41 led to a significant decrease in the protein expression levels of ARL5B, SKIP, KIF5B, and KLC2 when compared to the NC group and the control peptide group. Additionally, there were no significant changes in the protein expression of these markers in the NC group when compared to the control peptide group (**Figures 4I–R**). These results indicated that the RPL41 not only downregulated the expression of ARL5B but also suppressed the expression of other critical components in the lysosomal outward transport pathway. This implied that synthetic RPL41 peptide may modulate the invasion and metastasis of RB via the ARL5B-SKIP-Kinesin-1 pathway.

### 3.5 RPL41 restrains the expression of ATF4 and ARL5B *in vivo* in retinoblastoma

**Figure 5:**
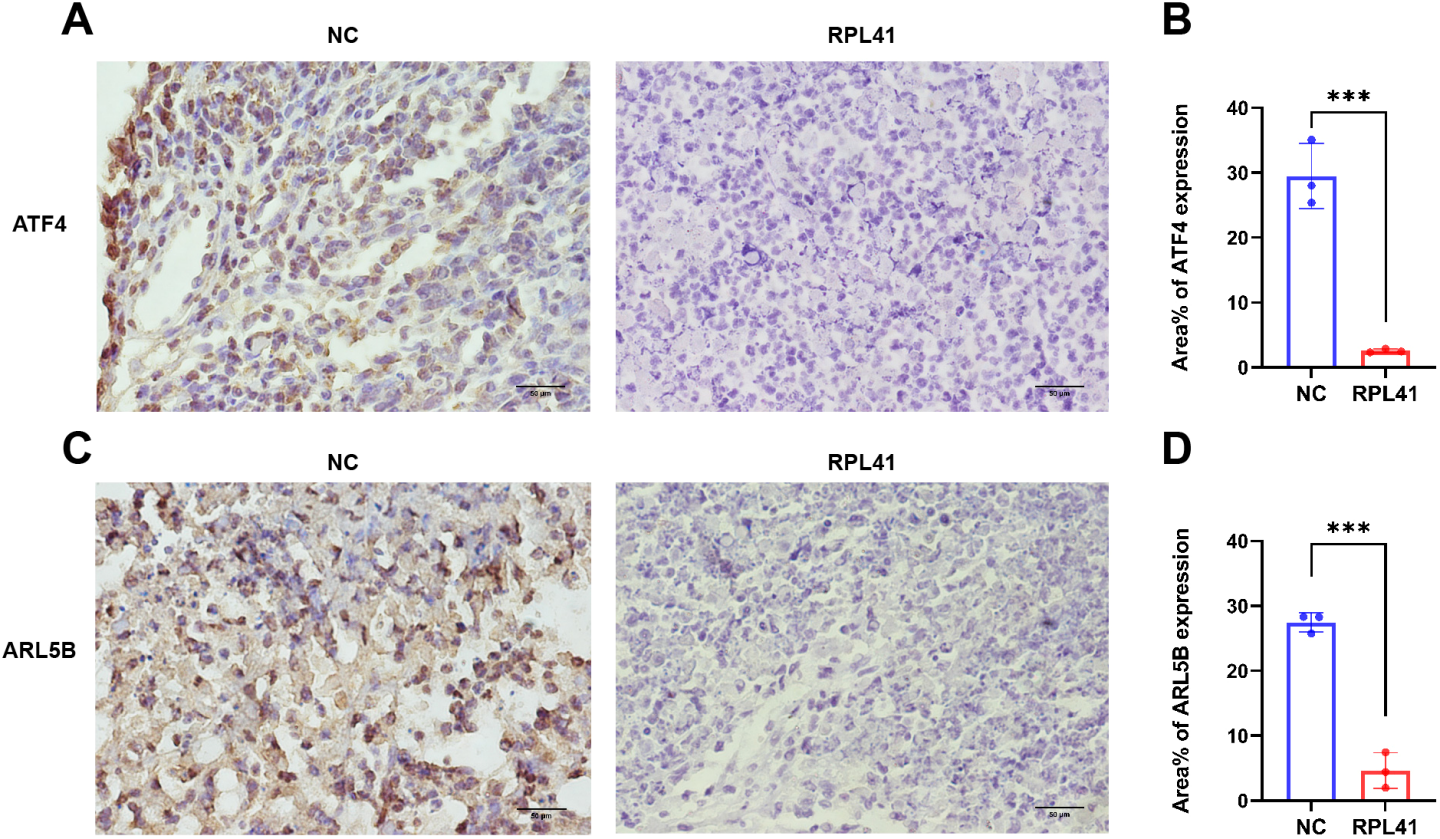
Mini-peptide RPL41 restrains the expression of ATF4 and ARL5B *in vivo* in retinoblastoma. **(A-D)** Immunohistochemical analysis of ATF4 and ARL5B expression in mice injected with Y79 cells and treated with mini-peptide RPL41. NC group, the blank control group. RPL41 group, RPL41 treated the tumor group. Data are presented as mean ± SD, n=3. Student’s t-test. Scale bar=50 μm. ***P < 0.001.

Previous studies have demonstrated that ATF4 undergoes degradation in cytoplasmic proteasomes following phosphorylation of the serine residue at position S219. Notably, the proteasomal inhibitor MG132 significantly blocked RPL41-induced ATF4 degradation, confirming that mini-peptide RPL41 mediates ATF4 degradation via the proteasomal pathway (20). Consistent with the previous verification (39), immunohistochemical staining of tumor tissues harvested from nude mice revealed that RPL41 treatment led to decreased expression levels of ATF4 (**Figures 5A, B**) and ARL5B (**Figures 5C, D**) in RB tissue, suggesting that RPL41 could inhibit the expression of these proteins. Moreover, the expression patterns of ATF4 and ARL5B were found to be consistent, which further suggested a potential regulatory relationship between these two genes. These *in vivo* findings aligned with the *in vitro* changes observed in cell models, supporting the conclusion that RPL41 effectively inhibited the growth of retinoblastoma.

### 3.6 Overexpression of ARL5B weakened the inhibitory effect of RPL41 on cell proliferation

**Figure 6:**
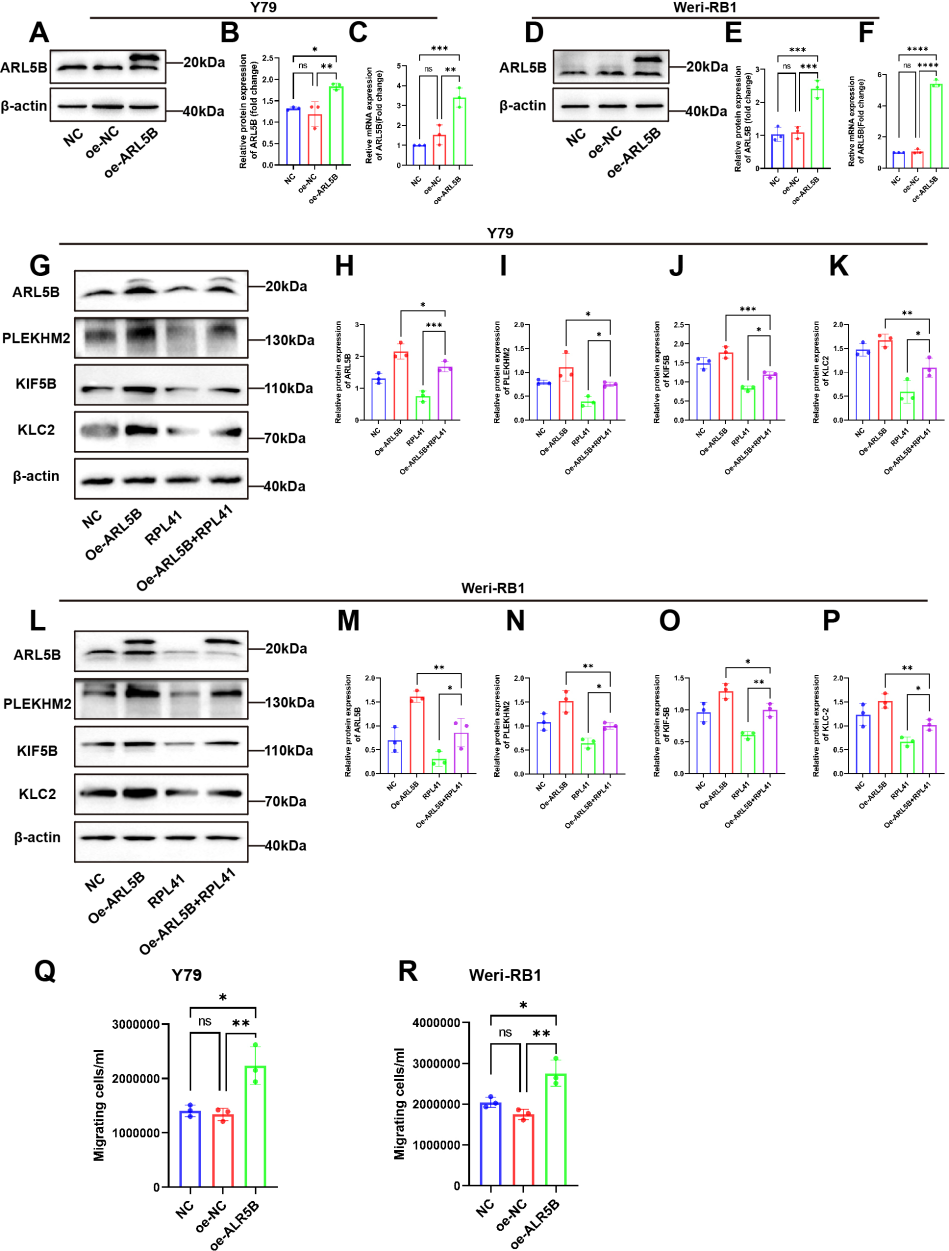
The overexpression of ARL5B impaired the inhibitory effect of mini-peptide RPL41 on the RB cells. **(A-F)** Expression of ARL5B protein and mRNA levels were examined after transfection of lentiviral empty vector or overexpression of ARL5B in **(A-C)** Y79 and **(D-F)** Weri-RB1 cells. Data are presented as mean ± SD, n=3. ANOVA test. **(G-P)** Mini-peptide RPL41 interfered with stable cell lines overexpressing ARL5B **(G-K)** Y79 and **(L-P)** Weri-RB1 for 24h compared with transfected ARL5B lentivirus alone and treated with mini-peptide RPL41 alone, Western Blot results and statistical plots of ARL5B, PLEKHM2, KIF5B and KLC2. n=3, ANOVA test. **(Q, R)** The number of migrating cells in the lower chamber **(Q)** Y79 and **(R)** Weri-RB1 were counted. n=3, ANOVA test. *P < 0.05; **P < 0.01; ***P < 0.001; ****P < 0.0001.

In order to further investigate the regulatory mechanism of the RPL41 on the behavior of Y79 and Weri-RB1 cell lines, we established a stable cell line for the lentiviral-mediated expression of ARL5B. This was achieved by transfecting lentivirus and co-transfecting with RPL41. qRT-PCR and Western blot analysis confirmed the successful establishment of the cell line (**Figures 6A–F**). The results indicated that, compared to the NC group and the oe-NC empty group, the mRNA and protein expression levels of ARL5B were significantly increased in the oe-ARL5B group. Furthermore, there was no significant difference in expression levels between the NC group and the oe-NC empty group. In the Western blot results, the electrophoretic migration bands of the fusion protein comprising ARL5B and the FLAG tag were slower compared to those of endogenous ARL5B. These findings confirmed the successful establishment of stable transfected cell lines of Y79 and Weri-RB1 overexpressing ARL5B.

Subsequently, we intervened with RPL41 in the stably transfected Y79 and Weri-RB1 cell lines that overexpressed ARL5B. Western blot analysis revealed that treatment with RPL41 alone inhibited the expression of ARL5B, SKIP, KIF5B and KLC2 in both Y79 and Weri-RB1 cells, while the protein expression levels of ARL5B, SKIP, KIF5B and KLC2 increased in Y79 and Weri-RB1 cells transfected with lentivirus for ARL5B overexpression. However, overexpression of ARL5B partially mitigated the downregulation of lysosomal diffusion-related genes induced by RPL41 (**Figures 6G–P**). The results of the Transwell assay indicated that the number of cells passing through the membrane pores in the oe-ARL5B group was significantly higher than that in both the NC group and the oe-NC control group (**Figures 6Q, R**). In contrast, no significant difference was observed in the number of cells passing through the membrane pores between the NC group and the oe-NC group. These findings suggested that the overexpression of ARL5B enhances the migratory ability of Y79 and Weri-RB1 cells.

These findings suggested that RPL41 can reduce the expression of ARL5B and inhibit the lysosomal trafficking. This was consistent with the results of proteomic analysis. When ARL5B expression was upregulated via lentiviral transfection, the inhibitory effect of RPL41 was diminished, which also impacted the migratory capacity of the cells. Therefore, it could be concluded that RPL41 may exert its inhibitory effect on retinoblastoma by modulating the ARL5B/SKIP/Kinesin-1 pathway.

### 3.7 ARL5B is a possible downstream target gene of ATF4

**Figure 7:**
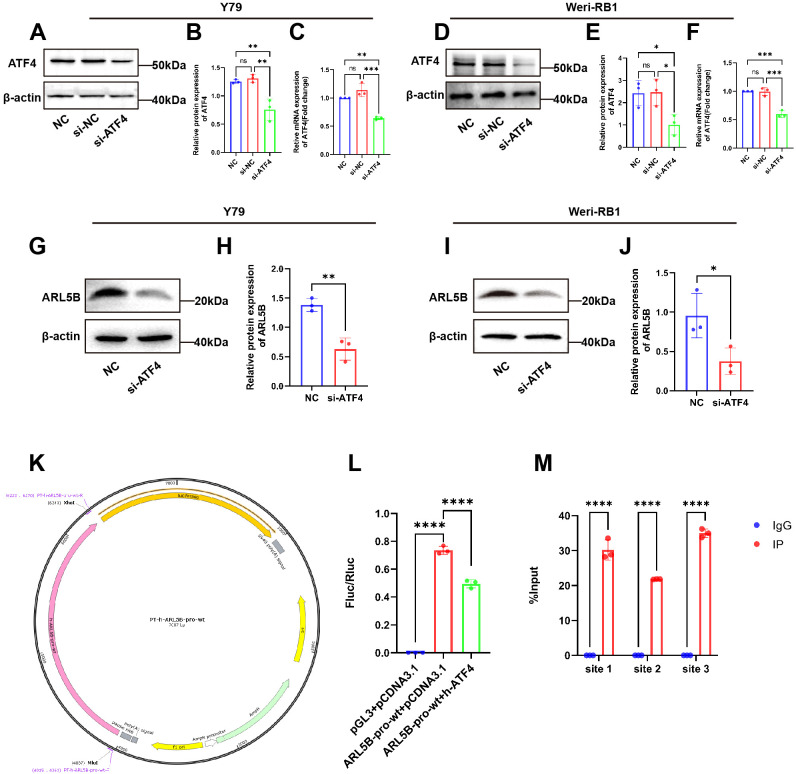
ARL5B is a possible downstream target gene of ATF4. **(A-F)** The transfection efficiency of ATF4 siRNA in **(A-C)**Y79 and **(D-F)**Weri-RB1 cells was confirmed by qRT-PCR and Western blotting. n=3, ANOVA test. **(G-J)** The protein level of ARL5B in ATF4-silenced Y79 and Weri-RB1 cells, n=3, Student’s t-test. **(K)** pGL3-Basic carrier map. **(L)** The DNA fragments of the wild-type or mutant ARL5B promoter were inserted into the pGL3-basic vector and co-transfected with the target plasmid into 293T cells for Luciferase reporter gene assay. n=3, ANOVA test. **(M)** Chromatin immunoprecipitation (ChIP) assays revealed that ATF4 binds to Site 1, Site 2, and Site 3 within the ARL5B promoter. Data are presented as mean ± SD, n=3. Student’s t-test. *P < 0.05, **P < 0.01, ***P < 0.001, ****P < 0.0001.

Our previous research identified that RPL41 processing was a micro-peptide that triggered the degradation of the transcription factor ATF4 (39). Consequently, we hypothesized that ATF4 may serve as an upstream factor affecting the expression of the ARL5B gene. To validate this hypothesis, we performed a knockdown of ATF4 expression in Y79 and Weri-RB1 cells and assessed its impact on ARL5B. Western Blot and qRT-PCR results indicated that the mRNA and protein expression levels of ATF4 in the si-ATF4 group were reduced compared to the NC and si-NC groups, with no significant difference observed between the NC and si-NC groups (**Figures 7A–F**). This finding demonstrated the successful transfection of ATF4 small interfering RNA. Subsequently, we discovered that knocking down ATF4 in Y79 and Weri-RB1 cells significantly reduced ARL5B protein expression levels (**Figures 7G–J**). This suggested that ATF4 influences ARL5B, indicating the potential for a transcriptional regulatory relationship between the two entities. We utilized the JASPAR database (https://jaspar.elixir.no/) to predict the upstream promoter sequences of the ARL5B gene. A dual luciferase reporter gene system was then constructed to verify the transcriptional activation activity of ATF4 on the ARL5B gene. These data suggested that ARL5B may be a downstream target gene of ATF4 (**Figures 7K, L**), and Chromatin immunoprecipitation-quantitative real-time PCR (ChIP-qPCR) confirmed that ATF4 binds to the specific regions within the ARL5B promote (**Figure 7M**).

### 3.8 Knockdown of ATF4 diminishes the enhancing effect of overexpressed ARL5B on Y79 and Weri-RB1 retinoblastoma cells

**Figure 8:**
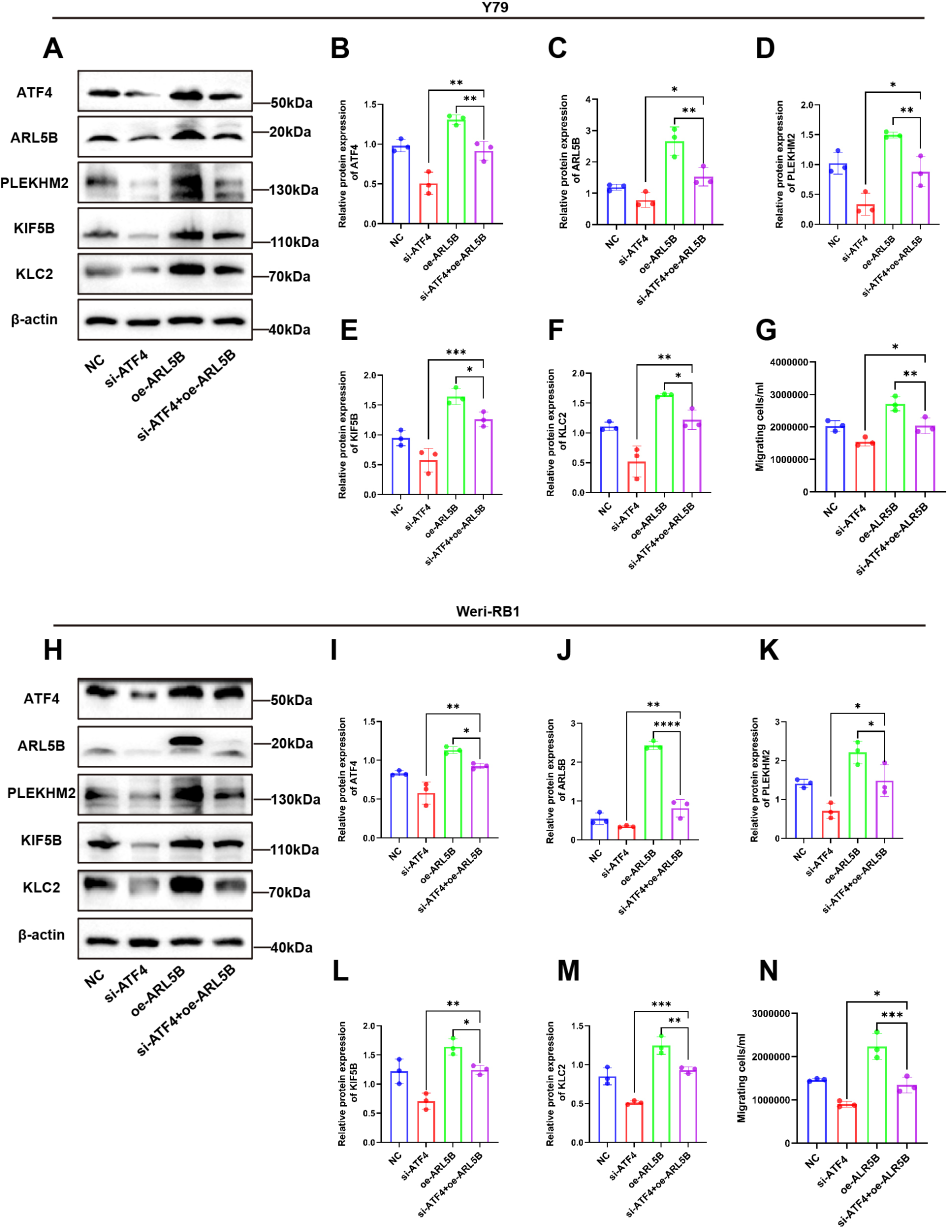
ATF4 knockdown affected the promotion effect of overexpressed ARL5B on Y79 and Weri-RB1 cells. **(A-F)** Western Blot images and statistical results for ATF4, ARL5B, SKIP, KIF5B, and KLC2 in Y79 cell groups are presented. n=3, ANOVA test. **(G)** Statistical results from Transwell experiments in Y79 cells. n=3, ANOVA test. **(H-M)** Western Blot images along with corresponding statistical results for ATF4, ARL5B, SKIP, KIF5B, and KLC2 in Weri-RB1 cell groups are shown. n=3, ANOVA test. **(N)** The number of migrating cells in the lower chamber Weri-RB1 were counted. Data are presented as mean ± SD, n=3. ANOVA test.*P < 0.05; **P < 0.01; ***P < 0.001; ****P < 0.0001.

To further elucidate the regulatory role of ATF4 on ARL5B, we established stable cell lines overexpressing ARL5B in Y79 and Weri-RB1 using lentiviral technology. Based on this, we applied small interfering RNA targeting ATF4 to knock down its expression level, aiming to verify the impact of ATF4 on ARL5B and the associated lysosomal spreading pathway. Western Blot results indicated that after transfection with si-ATF4 alone, the expression levels of ATF4, ARL5B, SKIP, KIF5B, and KLC2 proteins in Y79 and Weri-RB1 cells significantly decreased. In Y79 and Weri-RB1 cells with stable transfection overexpressing ARL5B, the expression levels of ATF4, ARL5B, SKIP, KIF5B, and KLC2 proteins were markedly increased. Subsequently, after transfection with si-ATF4 in cells overexpressing ARL5B, the expression levels of ATF4, ARL5B, SKIP, KIF5B, and KLC2 proteins in Y79 (**Figures 8A–F**) and Weri-RB1 (**Figures 8H–M**) cells decreased. Transwell assay results showed that the number of cells passing through the membrane pores in the si-ATF4+oe-ARL5B group was significantly higher than that in the si-ATF4 group. In comparison to the oe-ARL5B group, the number of cells passing through the membrane pores was significantly reduced (**Figures 8G, N**). The results indicated that the knockdown of ATF4 effectively inhibits the promoting effect of overexpressed ARL5B in Y79 and Weri-RB1 cells.

## 4 Discussion

**Figure 9:**
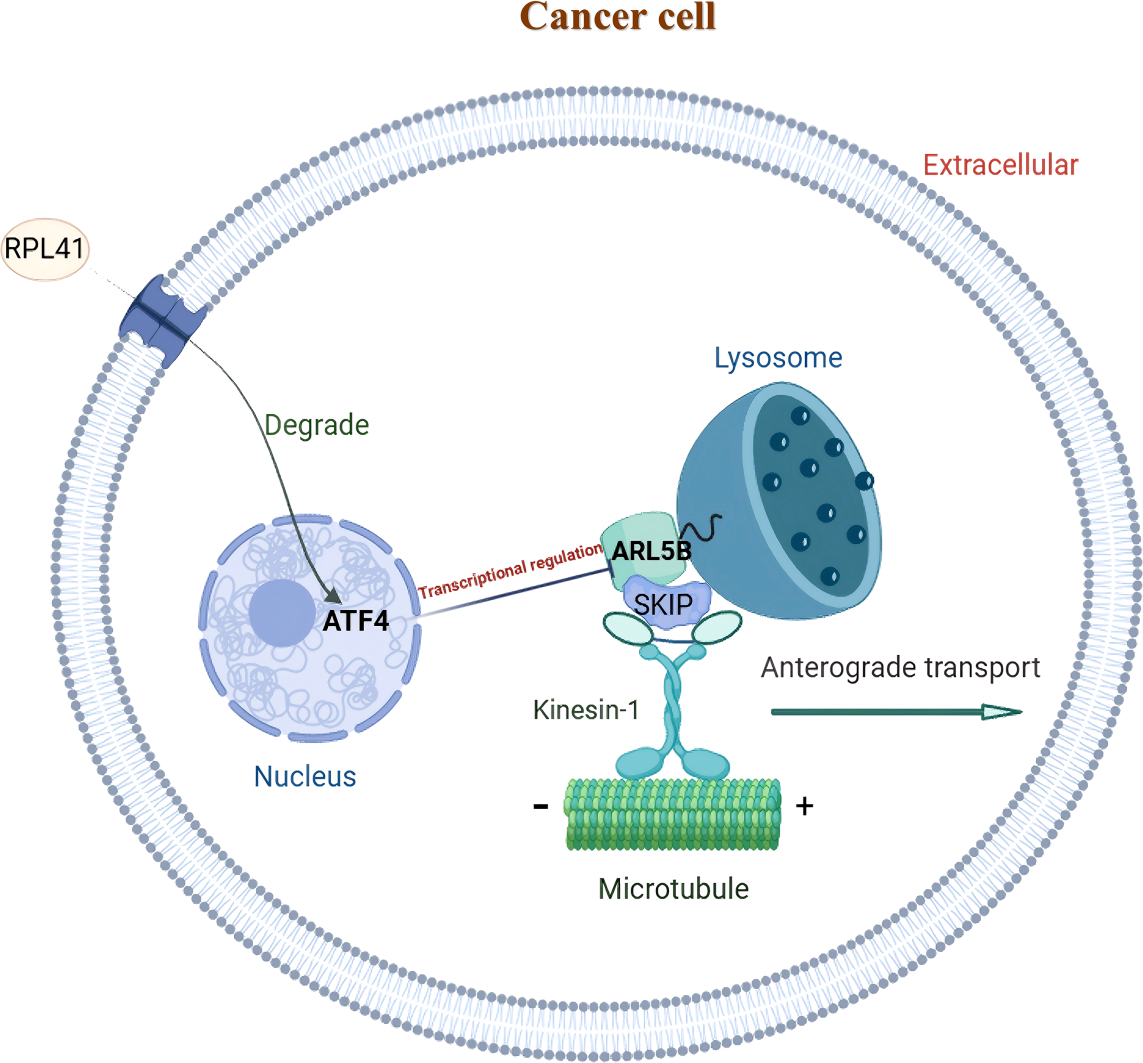
Schematic representation of the functional mechanism of mini-peptide RPL41 in retinoblastoma. ARL5B may function as a downstream target gene of the transcription factor ATF4 in retinoblastoma. The down-regulation of ATF4, mediated by mini-peptide RPL41, can attenuate the lysosomal trafficking pathway associated with ARL5B/SKIP/Kinesin-1 and inhibit RB cells proliferation and migration.

This study systematically investigated the mechanism by which RPL41 exerts its therapeutic effects in tumor treatment. Through proteomic analysis, we identified *ARL5B* as a significantly down-regulated gene following exposure to RPL41. Notably, *ARL5B* is highly expressed in retinoblastoma, and the activity of the ARL5B/SKIP/Kinesin-1 lysosomal trafficking facilitates the growth and metastasis of retinoblastoma. Additionally, ARL5B has been validated as a downstream target gene regulated by the transcription factor *ATF4* in retinoblastoma. Collectively, these findings suggest that RPL41 may effectively down-regulate ARL5B expression by inducing the degradation and activation of the transcription factor ATF4 (**Figure 9**).

In this study, *in vivo* experiments demonstrated that RPL41 significantly inhibits the proliferation of ectopically transplanted retinoblastoma in nude mice. Subsequently, proteomic identification and bioinformatics analysis revealed that ARL5B is downregulated following treatment with RPL41. Both *in vivo* and *in vitro* experiments confirmed the suppression of ARL5B expression after RPL41 treatment. Furthermore, overexpression of ARL5B was shown to promote the migration of retinoblastoma cells and partially counteract the inhibitory effects of RPL41 on these cells. These findings provide novel insights into the mechanism of action of RPL41 in the treatment of retinoblastoma. The results of the *in vivo* tumorigenesis experiments demonstrated that RPL41 inhibits tumor growth and slows the progression of RB by suppressing the expression of ATF4 and ARL5B in RB cells. Furthermore, in Y79 and Weri-RB1 cells, the expression level of ARL5B was significantly elevated. RPL41 not only reduced the expression of ARL5B but also inhibited the expression of SKIP (also known as PLEKHM2), a crucial molecule in the lysosome-dependent pathway, as well as the heavy chain KIF5B and light chain KLC2 of Kinesin-1 (40). Notably, the overexpression of ARL5B could partially counteract the inhibitory effects of RPL41 on the growth and metastasis of Y79 and Weri-RB1 cells. In summary, these findings indicate that the ARL5B/SKIP/Kinesin-1 lysosomal transport pathway is closely associated with the proliferation and metastasis of RB cells, and that RPL41 exerts its suppressive effects by inhibiting the activation of this pathway. From an immunological perspective, this pathway plays a significantly underappreciated role in tumor immune evasion, as lysosomes function not merely as “waste disposal units,” but as critical regulators of antigen presentation—a fundamental mechanism underlying T cell-mediated tumor recognition (41).

*ARL5B* is a protein-coding gene and the only small G protein present on mature lysosomes (34, 42, 43). Lysosomes are essential for the degradation of endogenous and exogenous antigens into peptide fragments, which are then loaded onto major histocompatibility complex (MHC) class I/II molecules and presented on the tumor cell surface to activate cytotoxic T cells or helper T cells (43). Du et al. demonstrated that in rectal cancer, lysosomal sorting and degradation of IFNGR1(interferon gamma receptor 1) compromise the integrity of the IFNγ(interferon-gamma) and MHC-I signaling pathways, thereby promoting immune evasion and intrinsic resistance to immunotherapy in colorectal cancer (44). Lysosomes move along microtubules toward the cell periphery in an ARL5B/SKIP/Kinesin-1 pathway-dependent manner, which is critical for tumor cell migration (45–47). Currently, many scholars are conducting relevant research on the *ARL5B* gene and have achieved significant findings. Ma et al. discovered that *ARL5B* was a key oncogene in esophageal squamous cell carcinoma (ESCC), promoting ESCC cell proliferation, invasion, and tumor growth *in vivo*, and potentially serving as a regulatory mechanism for lipid metabolism (48). Zhao et al. highlighted that targeting *ARL5B* can inhibit the mitochondrial function of ovarian cancer cells, which affects the metabolic reprogramming of tumor cells and consequently inhibits tumor growth, invasion, and metastasis in ovarian cancer (37). Moreover, studies have indicated that *ARL5B* may enhance the migratory and invasive capabilities of breast cancer cells by accelerating lysosomal redistribution to the cell periphery (38, 49). Based on the study by Shi et al. further indicated that in non-small cell lung cancer, *ARL5B* is essential for inhibiting cell proliferation, invasion, and mitochondrial function, while promoting apoptosis signaling pathways (50). These results suggest that *ARL5B* has a cancer-promoting effect and that its involvement in lysosomal movement is closely associated with tumor growth, migration, and invasion (47). Consistent with these findings, our data indicate that the overexpression of ARL5B in retinoblastoma enhances the expression of genes associated with the lysosomal trafficking pathway and increases the migration of RB cells. This may disrupt the antigen processing mechanism in two ways: firstly, the accelerated transport of lysosomes to the cell periphery may reduce the time antigens spend in the lysosomal lumen, limiting their complete degradation into immunogenic peptide fragments; secondly, abnormal lysosomal localization may alter the co-localization of MHC molecules with the antigen-loading compartments, thereby reducing the efficiency of peptide-MHC complex formation (26, 46). Notably, our experimental results indicate that the introduction of the exogenous RPL41 significantly downregulated the expression of ARL5B and restored lysosomal homeostasis. This finding suggests that RPL41 may have an indirect effect on antigen presentation—research related to prostate cancer also supports this hypothesis, demonstrating that the silencing of *ARL5B* is associated with an increased expression of MHC class I molecules on the surface of tumor cells (51). The transport process dependent on *ARL5B* can promote lysosomal exocytosis, releasing tissue cathepsins and other proteases into the tumor microenvironment (TME). These proteases not only degrade the extracellular matrix to facilitate tumor invasion but can also cleave immune checkpoint molecules (such as PD-L1, programmed death ligand 1) or chemokines (such as CXCL10, c-x-c motif chemokine ligand 10) in the TME (52, 53). For instance, it has been demonstrated that cathepsin B released via lysosomal exocytosis can truncate the chemokine that recruits CD8+ T cells, thereby reducing T cell infiltration into RB tumors (23). Therefore, RPL41 may reduce protease release and maintain the chemokine gradient by inhibiting ARL5B-mediated lysosomal exocytosis, thereby promoting the recruitment of anti-tumor immune cells. The role of RPL41 in regulating signals within the tumor immune microenvironment warrants further investigation. This discovery offers a novel research direction for exploring the regulatory mechanisms of the lysosomal trafficking.

Mechanistically, *ATF4* may function as an upstream transcription factor that initiates the expression of the ARL5B gene. Initial observations from clinical retinoblastoma pathological sections revealed a consistent expression trend, wherein the levels of ATF4 and ARL5B were both lower in normal para-cancerous retinal tissues compared to RB tumor tissues. In cell-based experiments, ARL5B protein expression was significantly reduced following ATF4 knockdown, while overexpression of ARL5B partially reversed the downregulation effects induced by ATF4 knockdown. Furthermore, results from double luciferase reporter assays and ChIP-qPCR assays provided robust support for these conclusions. Collectively, these findings indicate that ATF4 plays a crucial role in the regulation of ARL5B expression.

Malabanan et al. proposed that ATF4 specifically activates or binds to regulate vascular endothelial growth factor-A (VEGF-A), endothelial cell selectin (E-selectin) and urokinase-type plasminogen activator (uPA), which play crucial roles in tumor invasion and metastasis (11). In this study, we propose that ATF4 may promote tumor growth and metastasis by activating the expression of ARL5B, thereby influencing the SKIP/Kinesin-1 pathway. Our experimental results indicate that knocking down ATF4 significantly inhibits the proliferation and migration of tumor cells. Given that RPL41 exhibits a substantial degrading effect on ATF4, we speculate that the anti-tumor effect of RPL41 may operate through at least the aforementioned two pathways. However, the specific contributions and interrelationships of these different pathways remain unclear, and further research is urgently needed to explore this mechanism in greater depth. It is noteworthy that our proteomics analysis identified several differentially expressed proteins in addition to ARL5B, which warrant further investigation. While ARL5B was prioritized due to its consistent downregulation in Y79 and Weri-Rb1 cells and its functional association with retinoblastoma cell migration—supported by preliminary overexpression experiments—the remaining candidate proteins require systematic validation and will be the focus of future studies. These include proteins involved in vesicle transport, such as Rab10, a pathway closely linked to ARL5B-mediated lysosomal trafficking (54), as well as ELOVL5(elovl fatty acid elongase 5), which has been implicated in tumor immunity through immunometabolic regulation (55). Further exploration of these candidates will contribute to a more comprehensive understanding of the anti-tumor mechanisms of RPL41 beyond the ATF4-ARL5B axis. In addition, a notable limitation of this study is the use of a subcutaneous RB xenograft model, which fails to recapitulate the physiological ocular microenvironment—including the composition of the retinal ECM, intraocular immune characteristics, and signaling interactions with resident ocular cells. This discrepancy may result in altered RB cell behavior (e.g., invasion patterns) and differential therapeutic responses to RPL41 compared to those occurring in the native intraocular environment *in vivo*, potentially limiting the translational relevance of the study’s *in vivo* findings.

## 5 Conclusions

In summary, we presented *in vivo* and *in vitro* evidence that *ARL5B*, which is highly expressed in retinoblastoma, promotes cell migration via the ARL5B/SKIP/Kinesin-1 signaling pathway. Furthermore, our findings underscore the direct regulatory role of *ATF4* on *ARL5B* and demonstrate that RPL41 can inhibit lysosomal trafficking along the ARL5B/SKIP/Kinesin-1 signaling axis by promoting the degradation of ATF4. From a therapeutic standpoint, these findings highlight the potential of RPL41 as a dual-functional candidate for RB treatment: it can serve as a targeted agent to suppress the ATF4-driven oncogenic pathway and act as a chemosensitizer to enhance the efficacy of current platinum-based therapeutic regimens. This not only offers a novel strategy to overcome chemoresistance and optimize RB treatment protocols but also holds particular relevance for pediatric patients with advanced or recurrent RB, who often face limited therapeutic alternatives. To advance this promising therapeutic strategy against retinoblastoma, further research is essential to explore the clinical significance of RPL41 in this context, identify potential predictive factors, and conduct additional experiments as well as preclinical studies.

## Data Availability

The original contributions presented in the study are included in the article/**Supplementary Material**. Further inquiries can be directed to the corresponding authors.
