# Supplementary material for: RPL41 inhibits the proliferation and migration of retinoblastoma through the ARL5B-associated lysosomal trafficking
Source: Front Immunol. 2025 Dec 10;16:1704080. doi: 10.3389/fimmu.2025.1704080 (PMC12727911; doi:10.3389/fimmu.2025.1704080)

FIGURE-4

Y79(ARL5B)

The change in ARL5B protein expression was verified by Western blot using a 12.5% separating gel in the Y79 cells among the control group, control peptide group, and RPL41 intervention group.

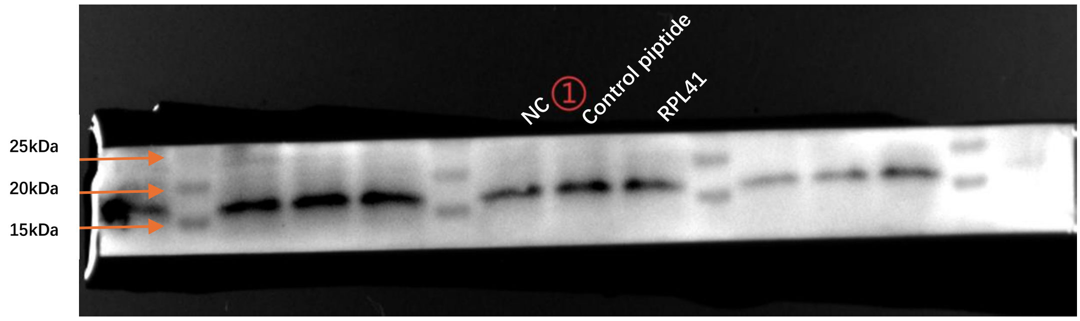

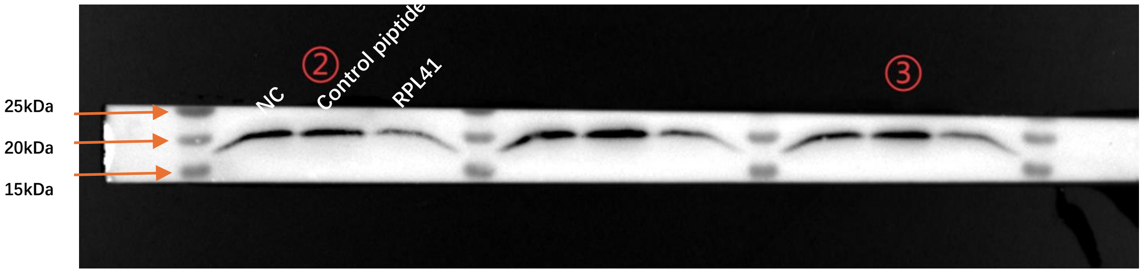

(β-Actin)

The change in β-Actin protein expression was verified by Western blot using a 12.5% separating gel in the Y79 cells among the control group, control peptide group, and RPL41 intervention group.

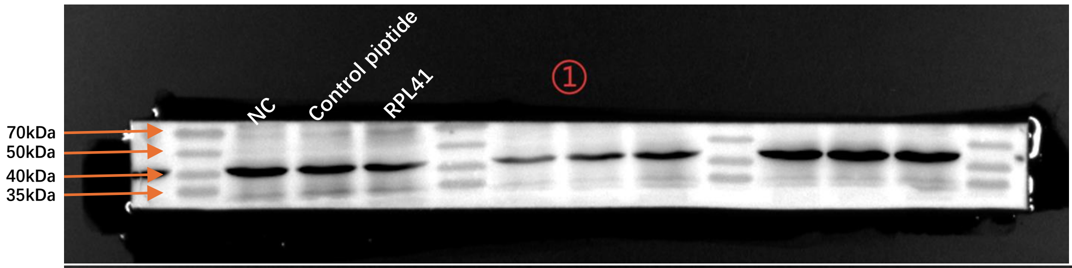

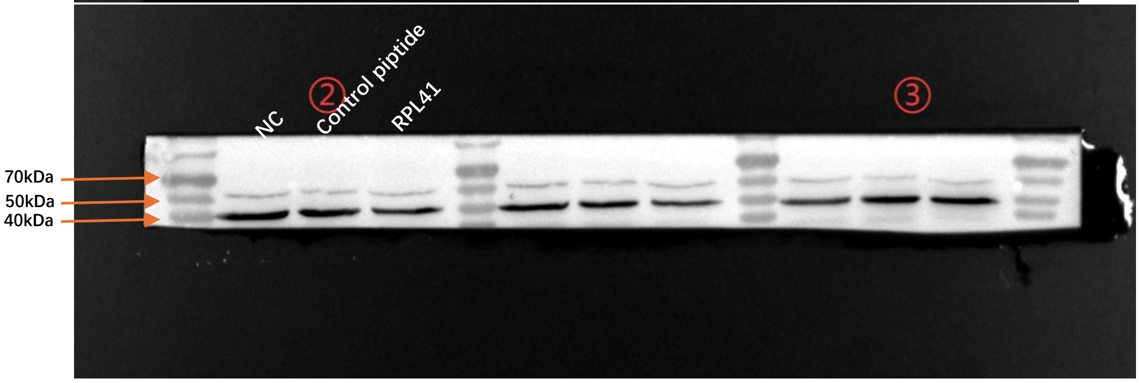

The change in SKIP protein expression was verified by Western blot using a 12.5% separating gel in the Y79 cells among the control group, control peptide group, and RPL41 intervention group.

Y79(SKIP)

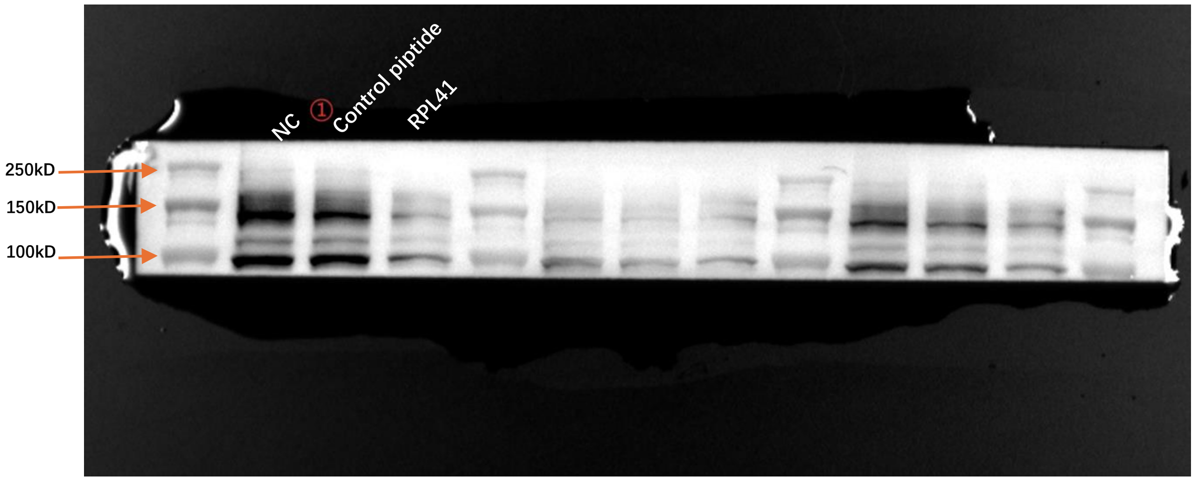

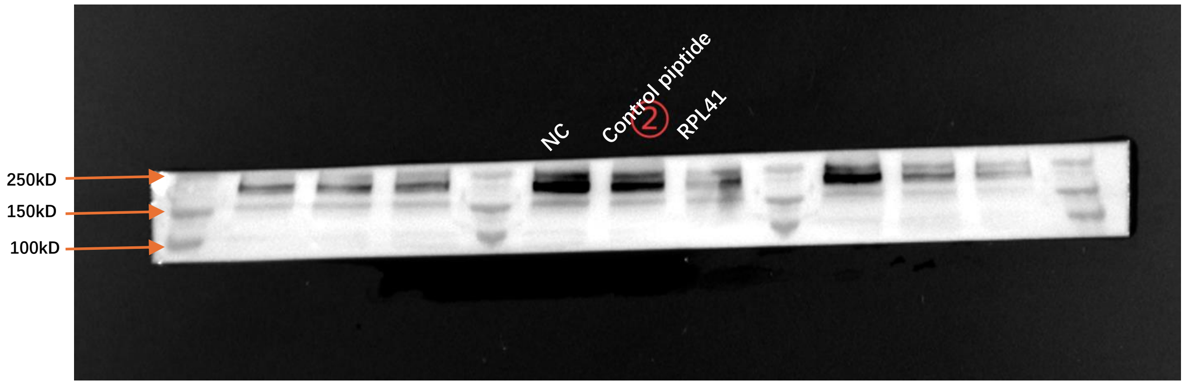

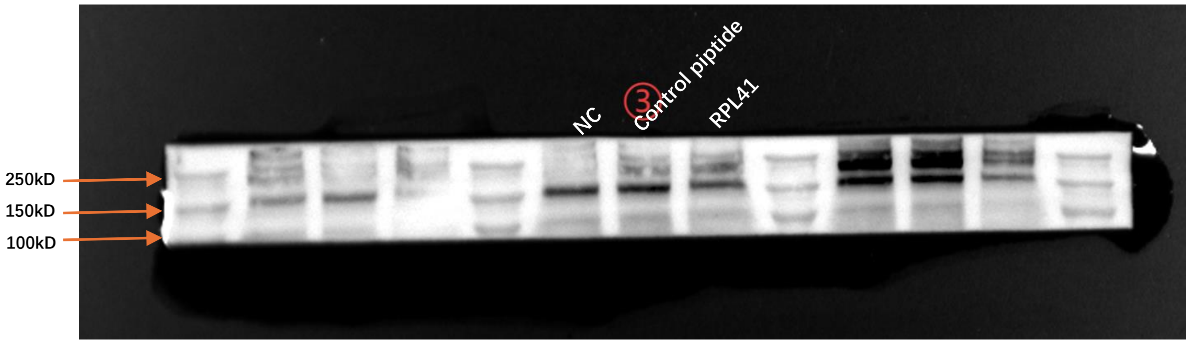

The change in β-Actin protein expression was verified by Western blot using a 12.5% separating gel in the Y79 cells among the control group, control peptide group, and RPL41 intervention group.

(β-Actin)

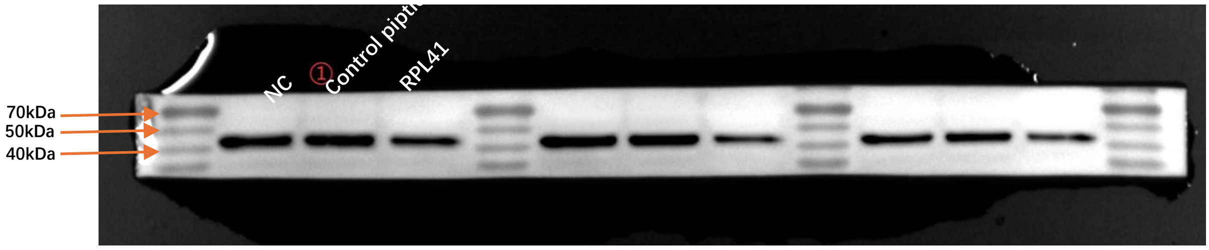

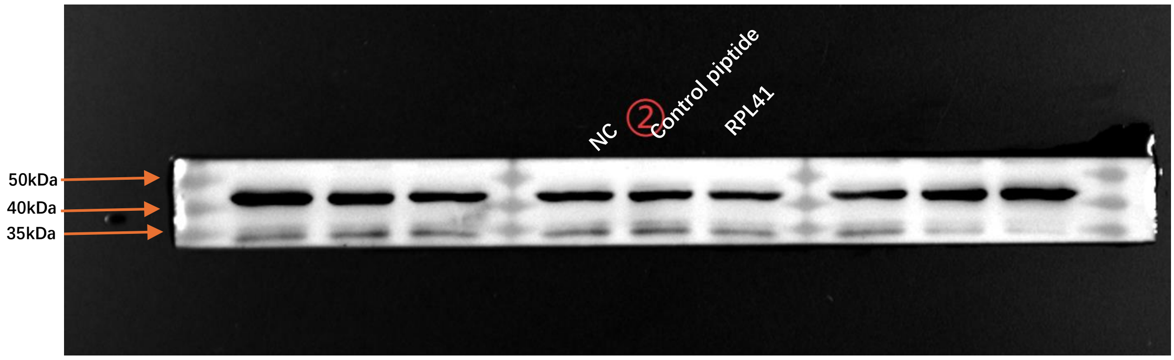

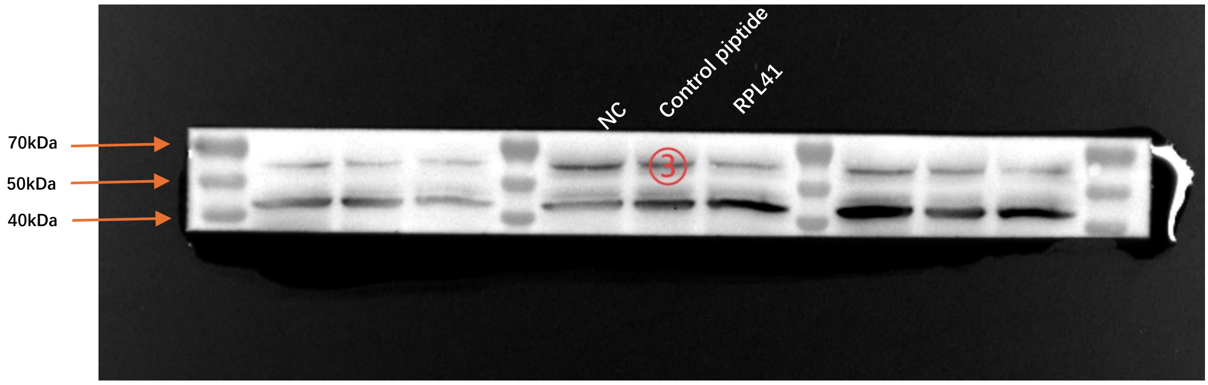

The change in KIF5B protein expression was verified by Western blot using a 7.5% separating gel in the Y79 cells among the control group, control peptide group, and RPL41 intervention group.

Y79(KIF5B)

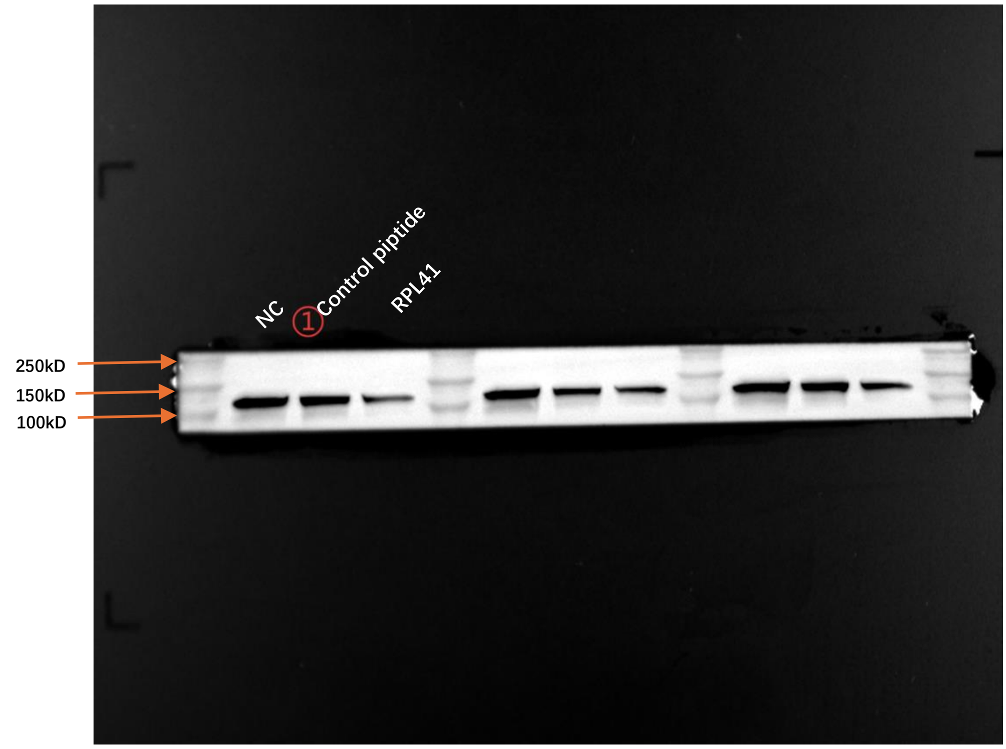

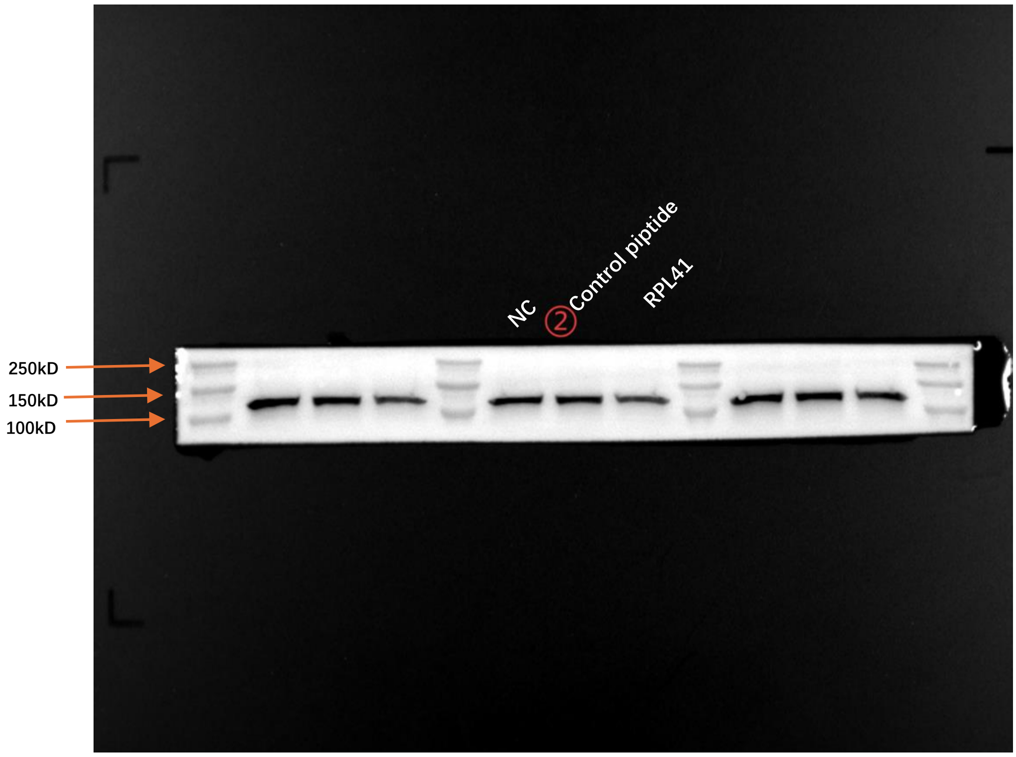

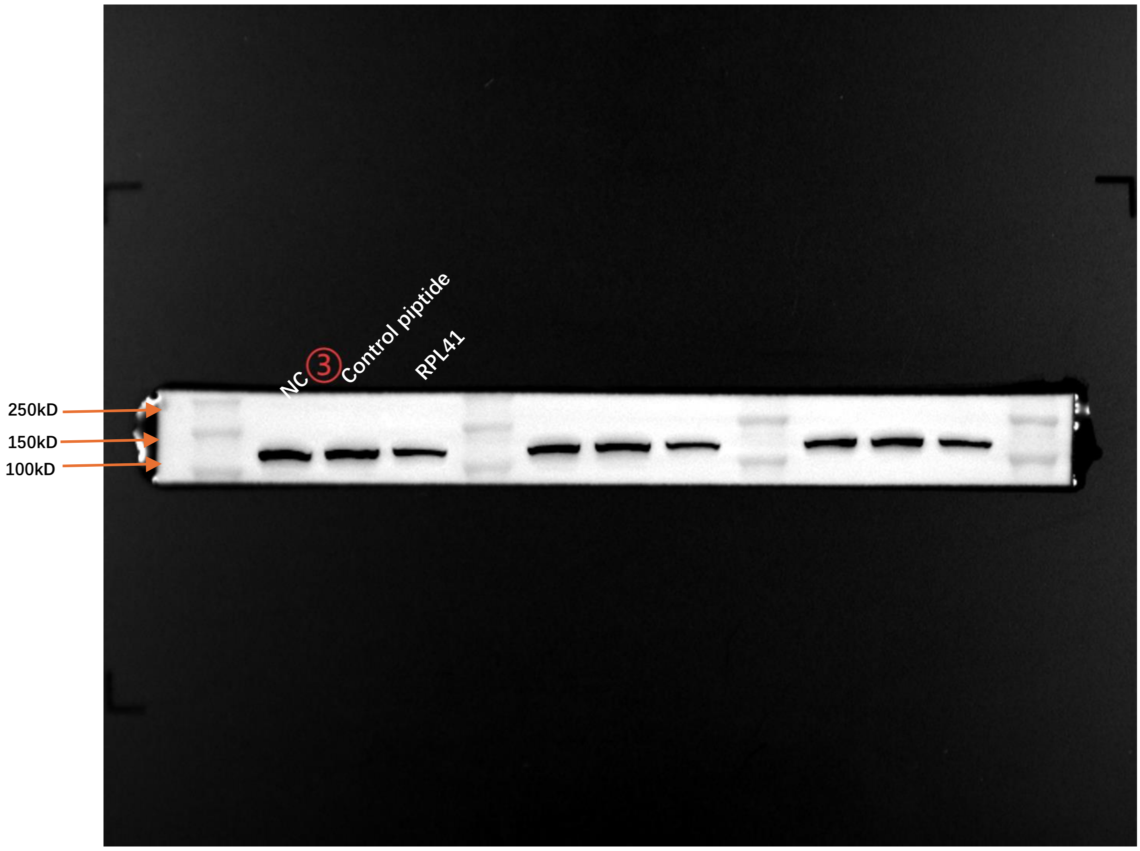

(β-Actin)

The change in β-Actin protein expression was verified by Western blot using a 7.5% separating gel in the Y79 cells among the control group, control peptide group, and RPL41 intervention group.

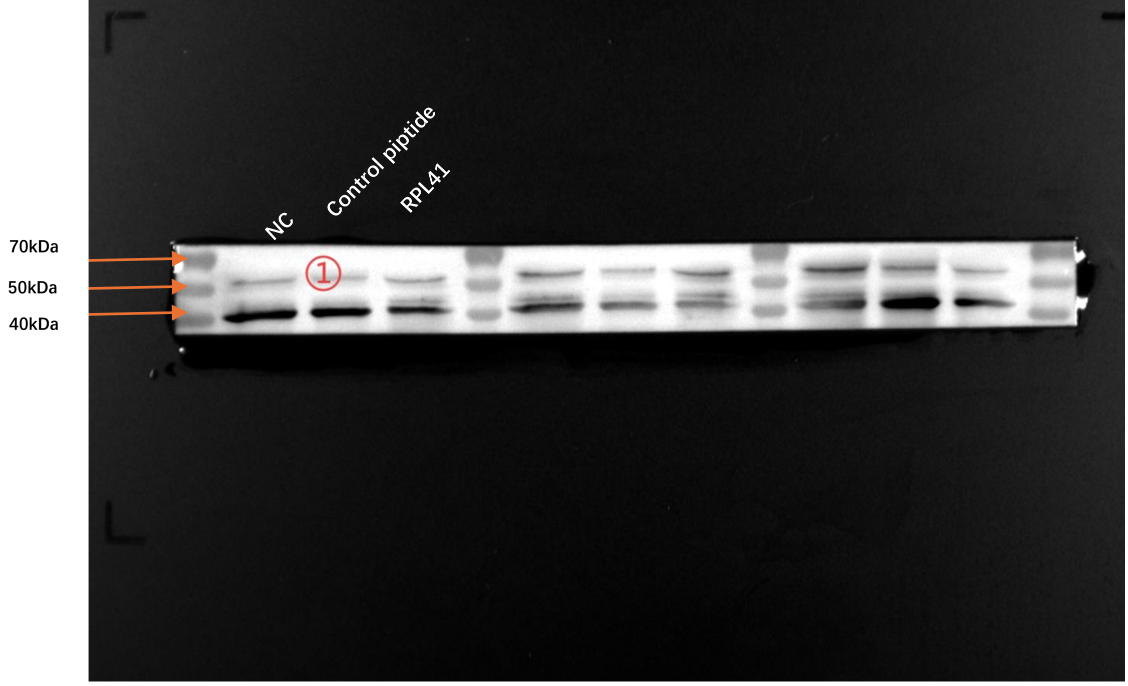

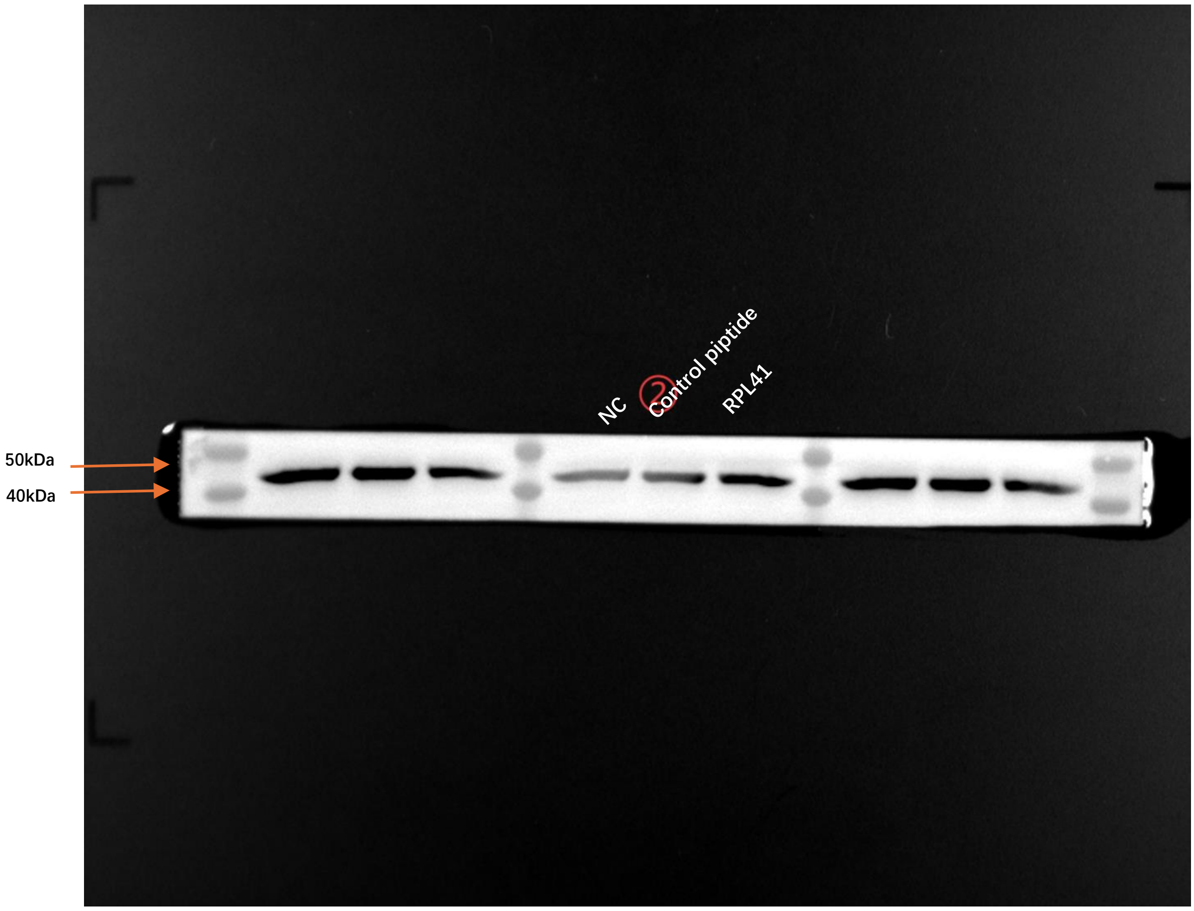
NC
②
Control piptide
RPL41
50kDa
40kDa

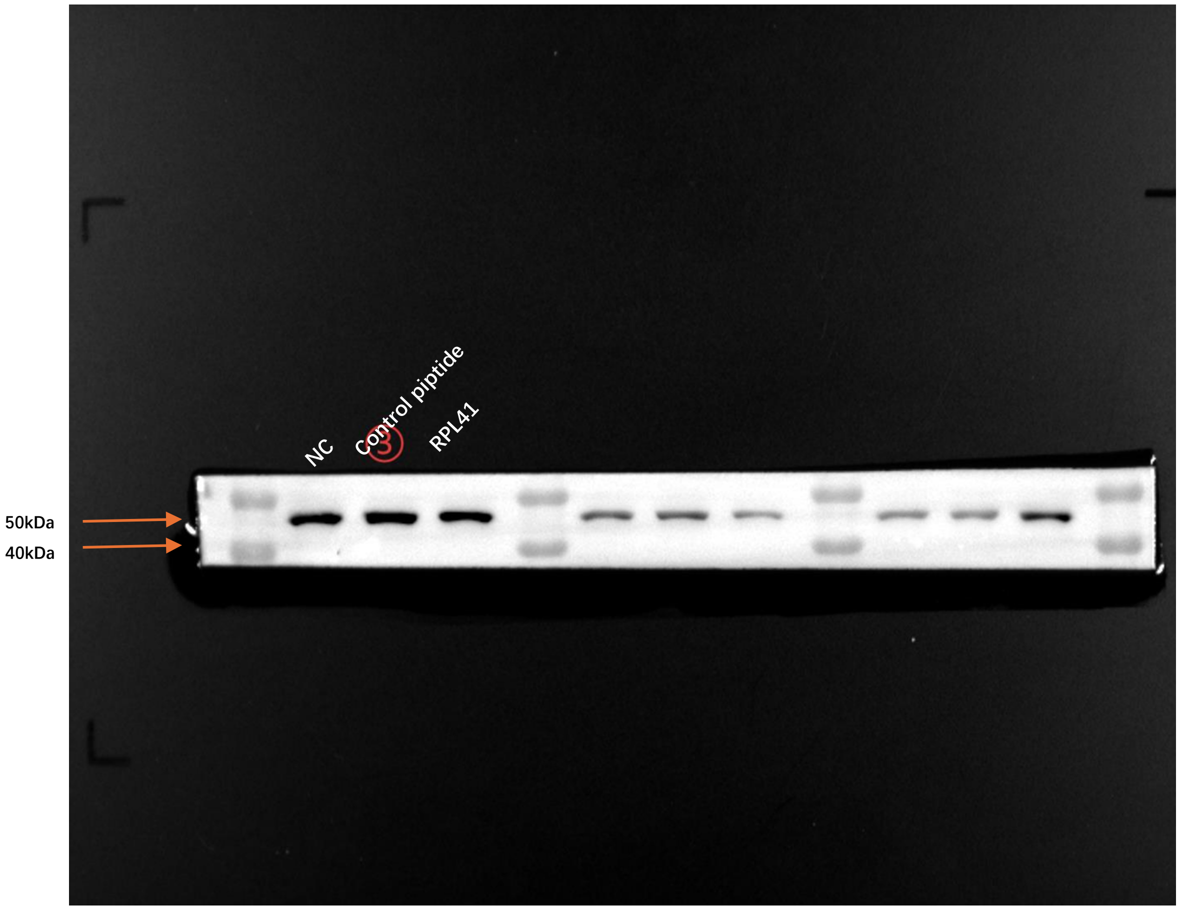
NC
③
Control piptide
RPL41
50kDa
40kDa

Y79(KLC2)

70kDa

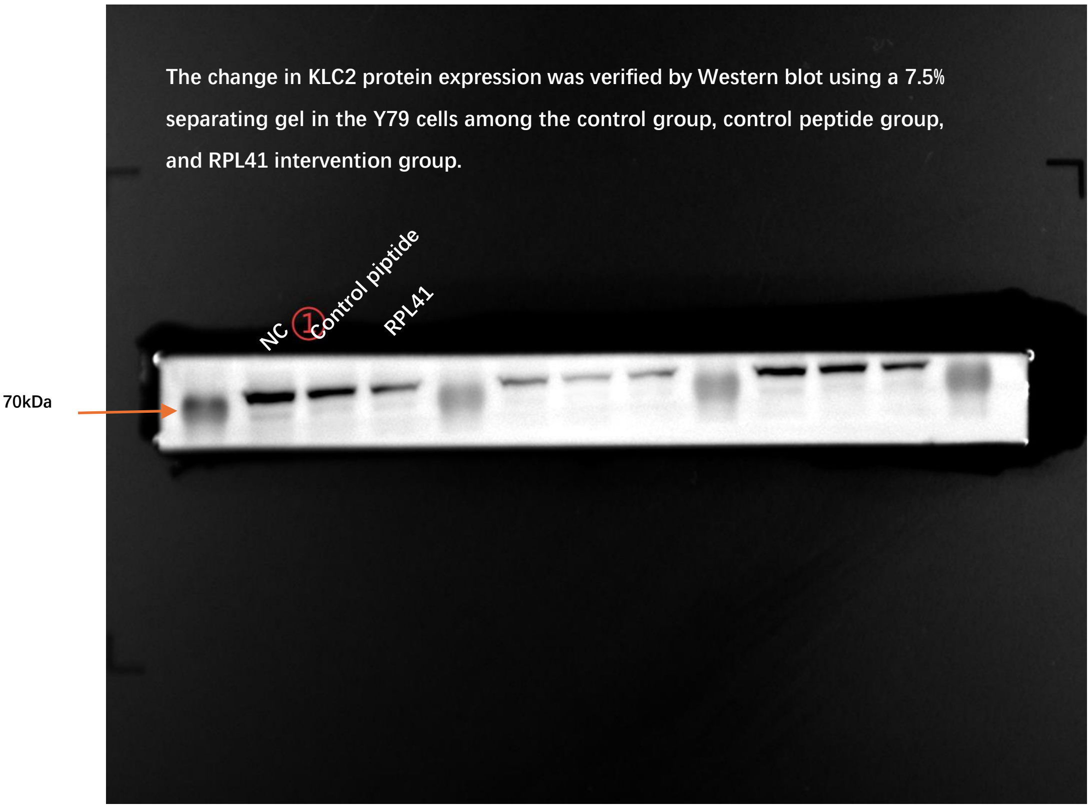

70kDa

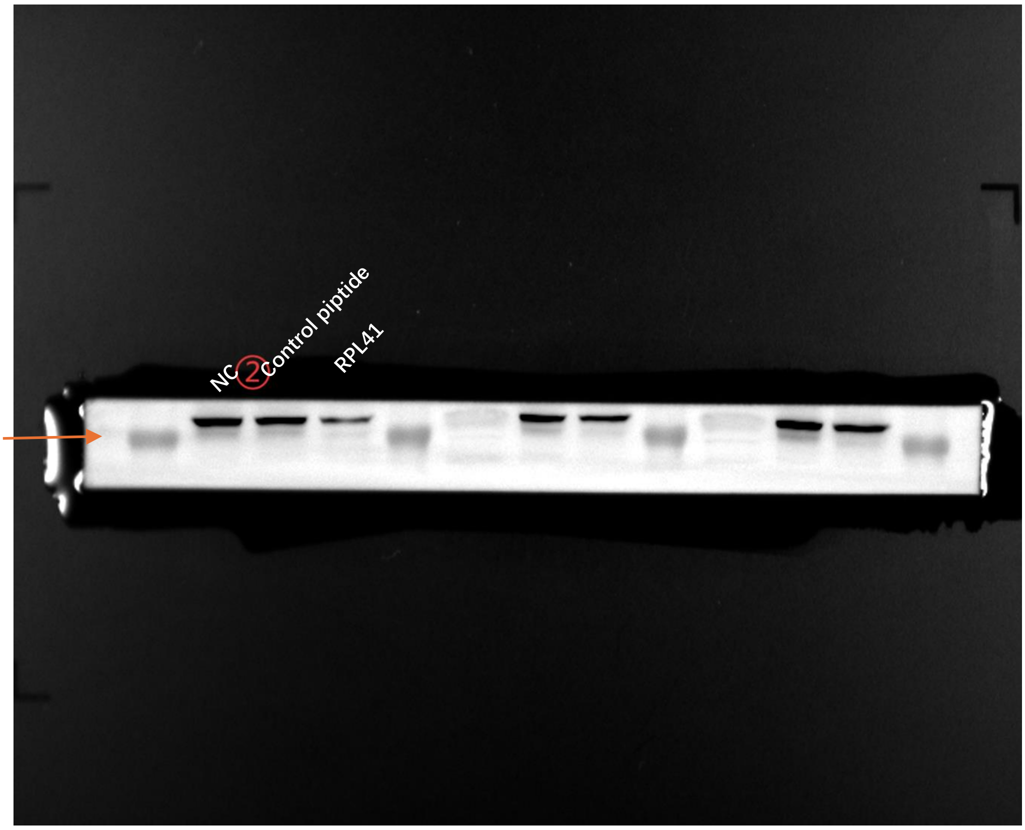

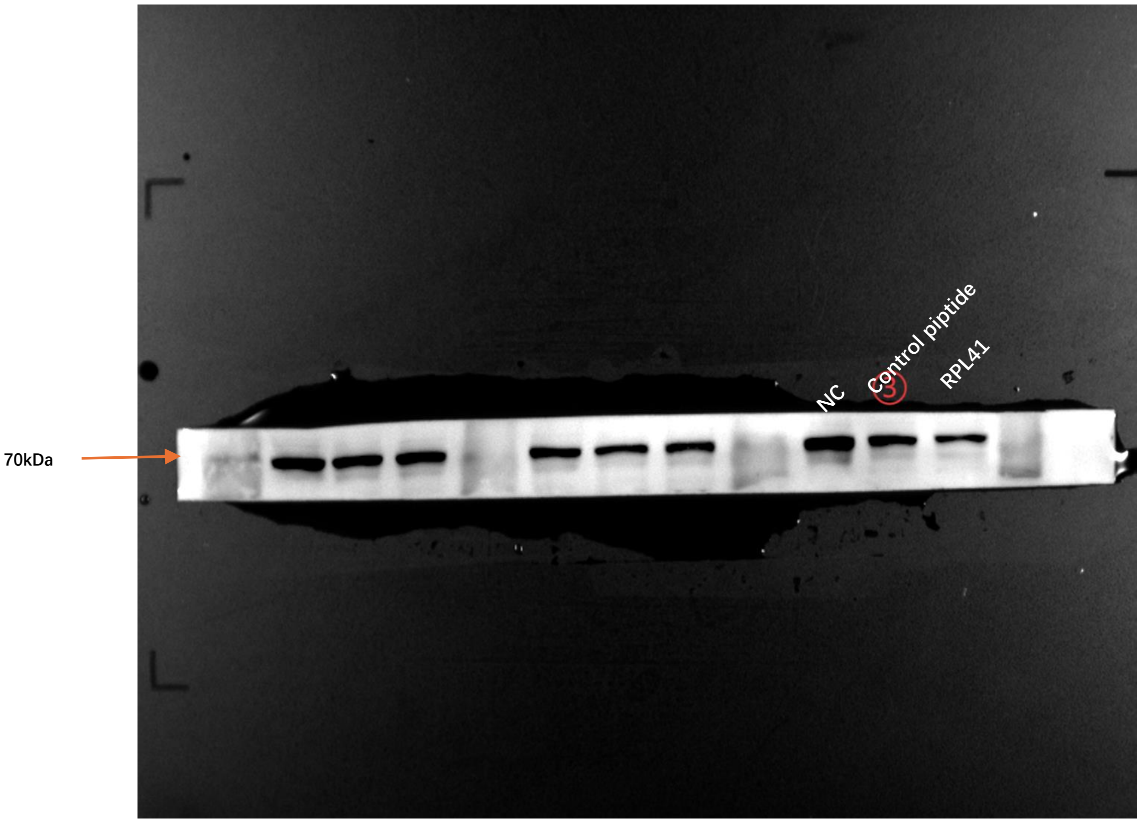
NC
Control piptide
RPL41
70kDa

(β-Actin)

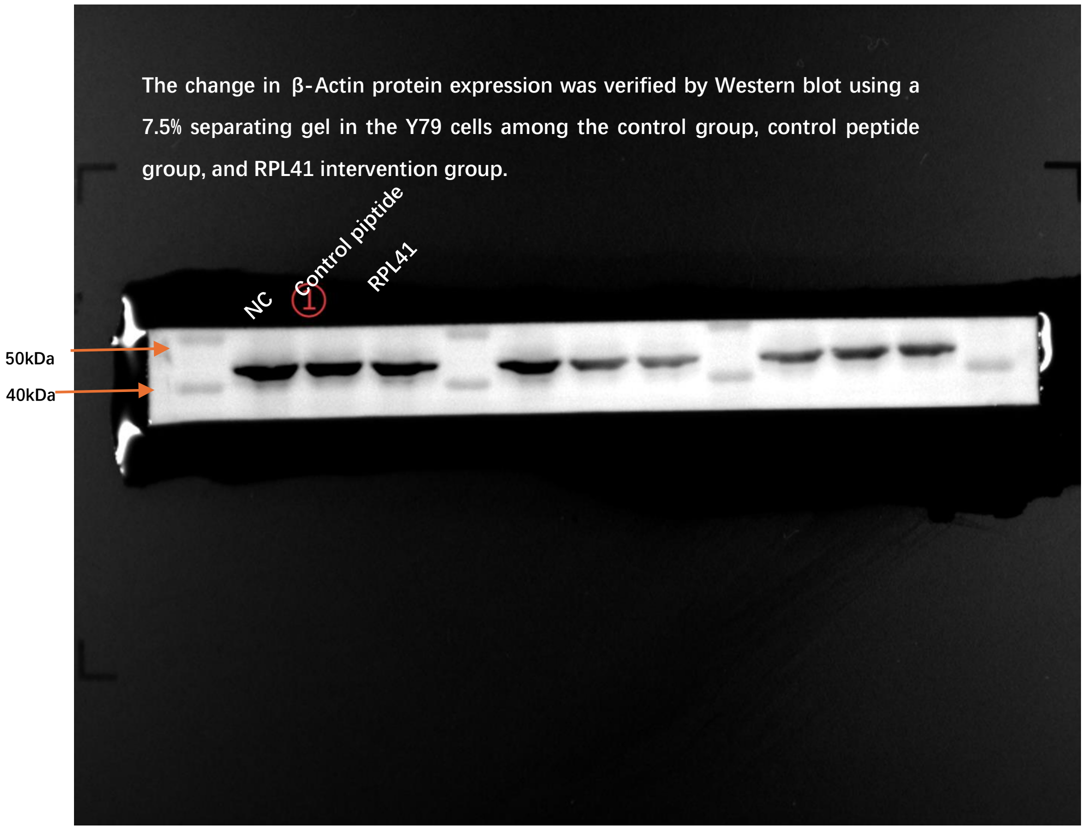
The change in β-Actin protein expression was verified by Western blot using a 7.5% separating gel in the Y79 cells among the control group, control peptide group, and RPL41 intervention group.
NC
Control piptide
RPL41
50kDa
40kDa

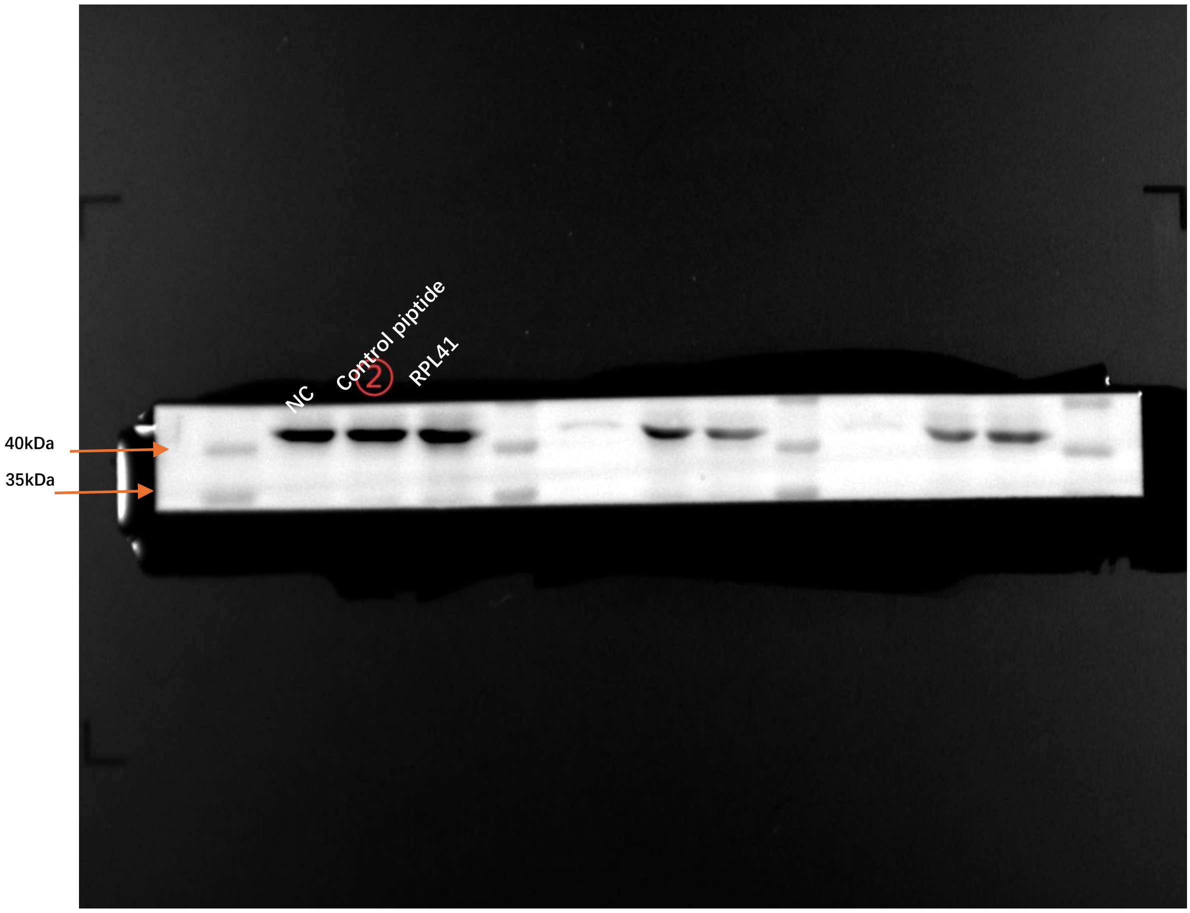
NC
Control piptide
2
RPL41
40kDa
35kDa

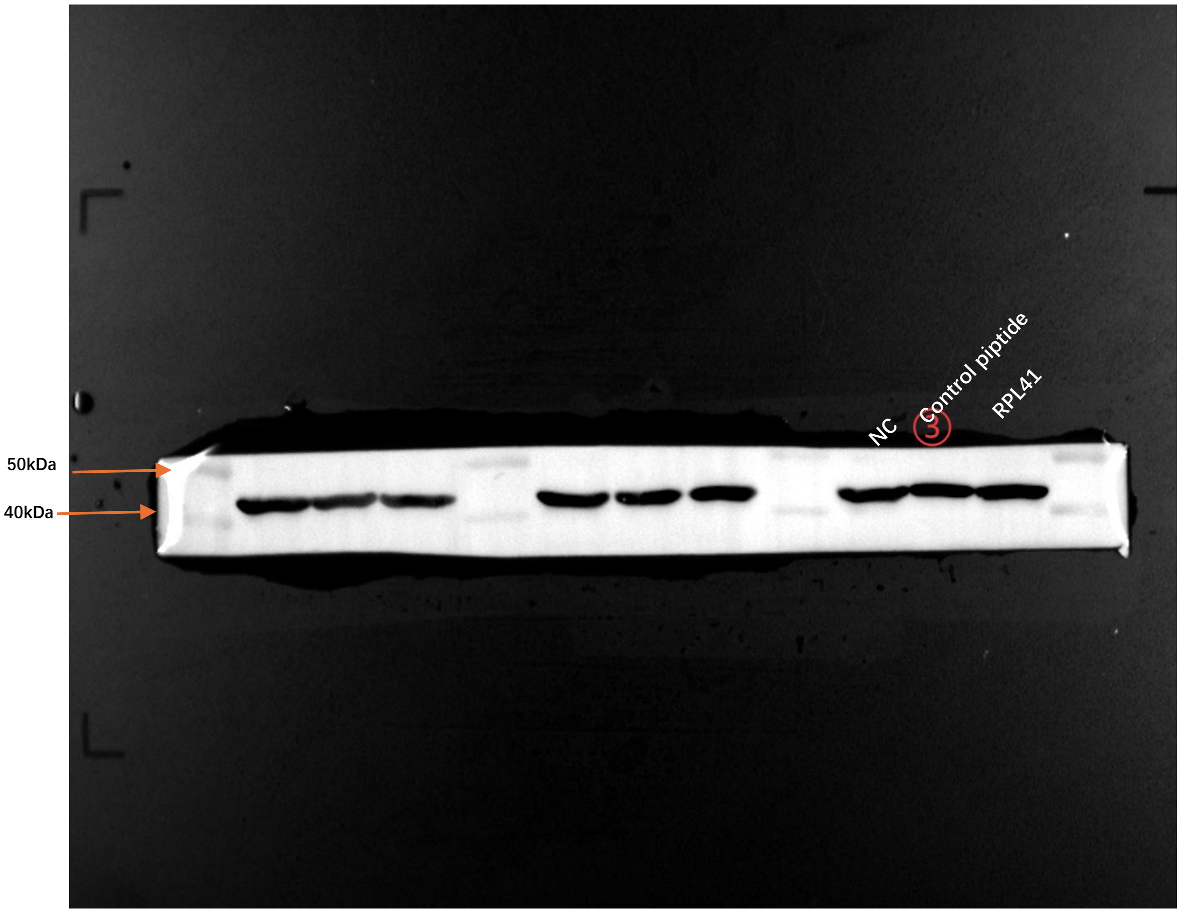
NC
Control piptide
3
RPL41
50kDa
40kDa

Weri-RB1(ARL5B)

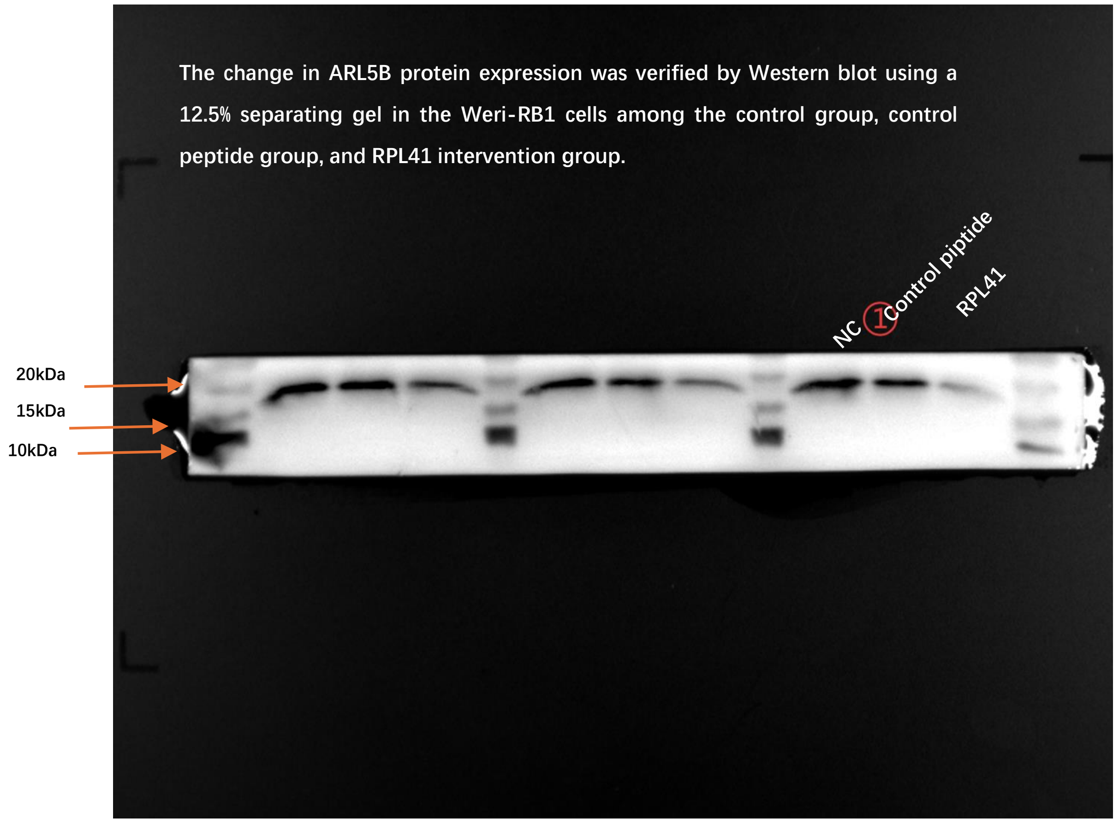

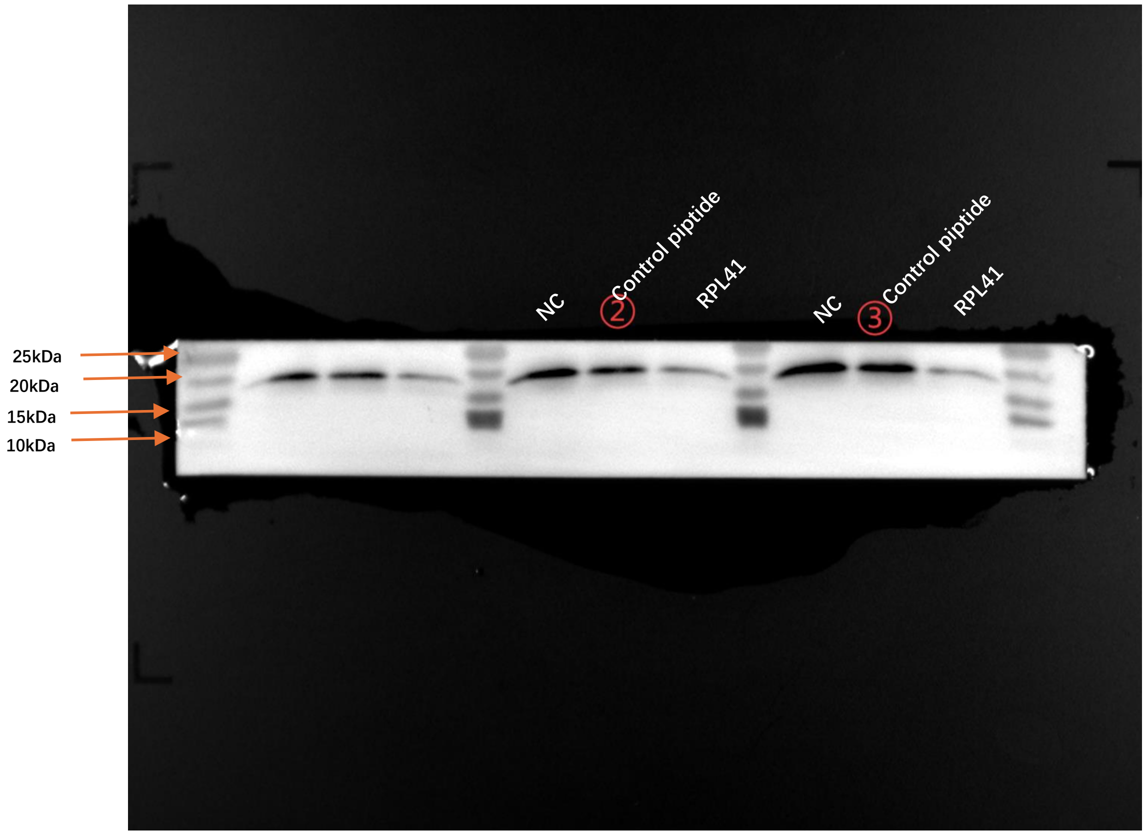

(β-Actin)

The change in β-Actin protein expression was verified by Western blot using a 12.5% separating gel in the Weri-RB1 cells among the control group, control peptide group, and RPL41 intervention group.

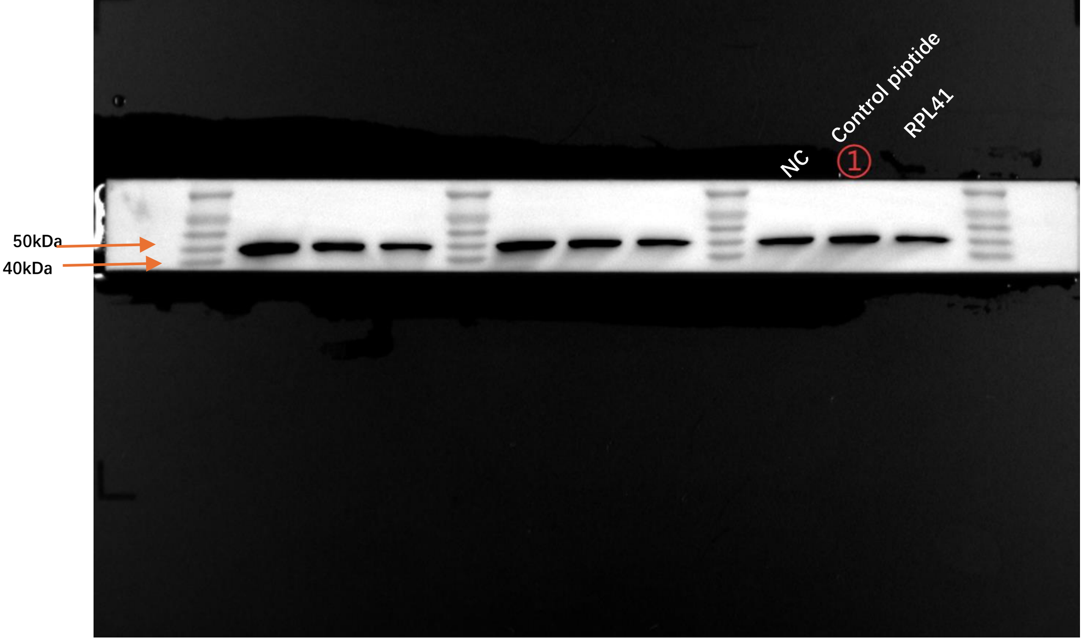

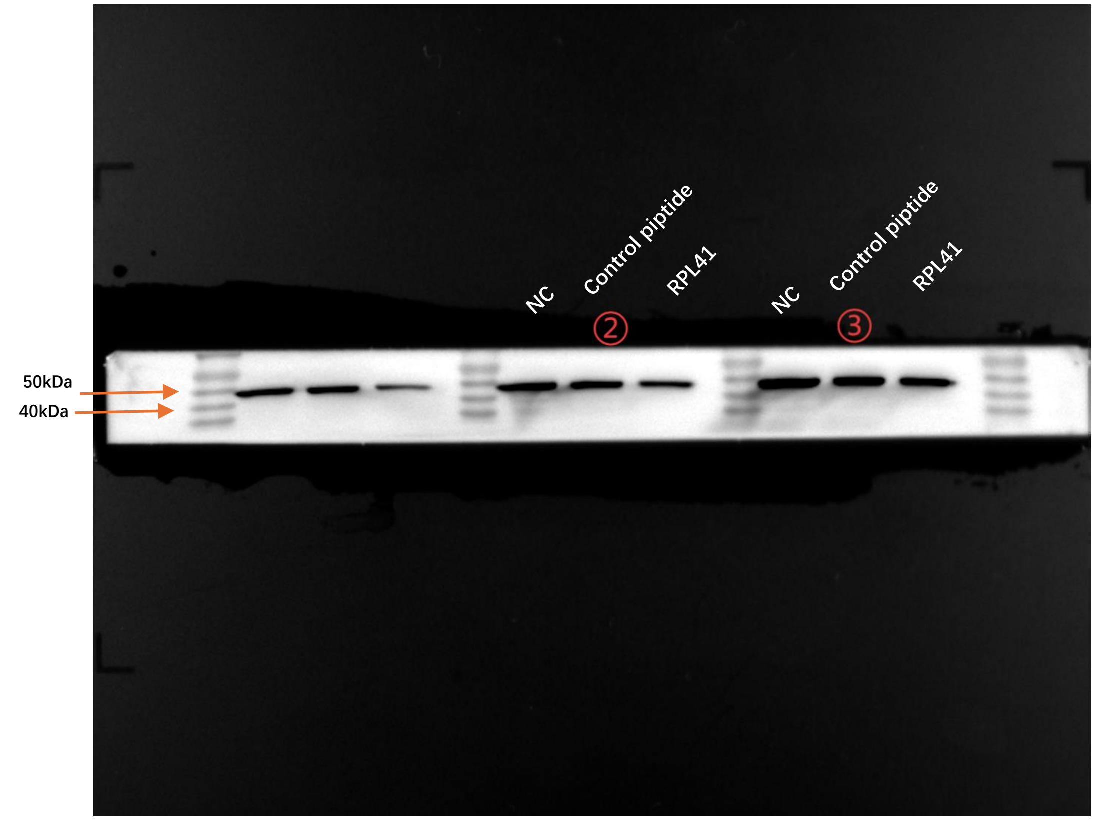

Weri-RB1(SKIP)

The change in SKIP protein expression was verified by Western blot using a 7.5% separating gel in the Weri-RB1 cells among the control group, control peptide group, and RPL41 intervention group.

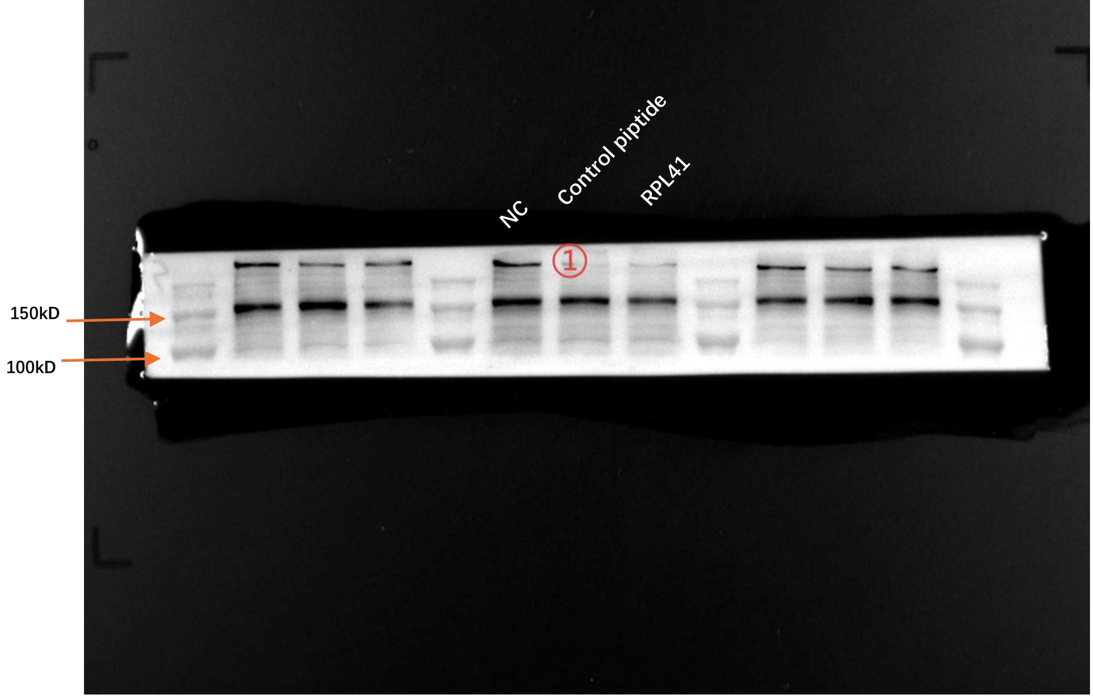

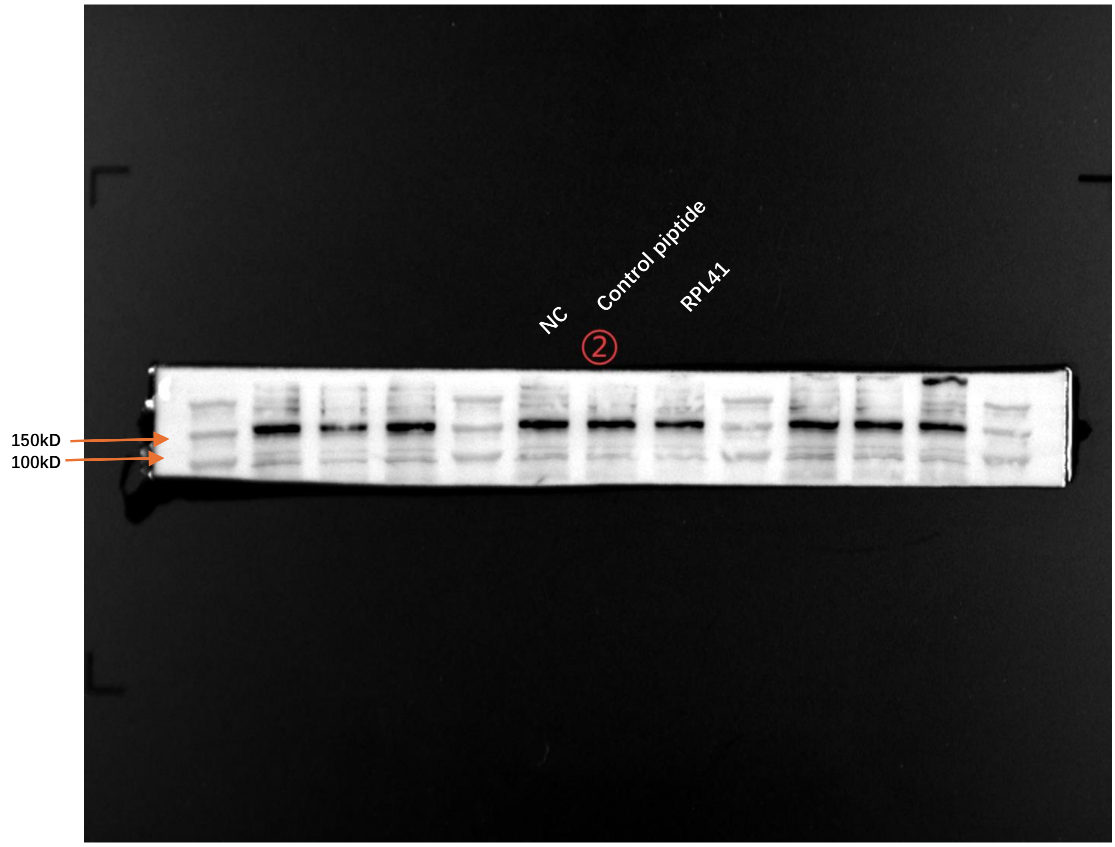

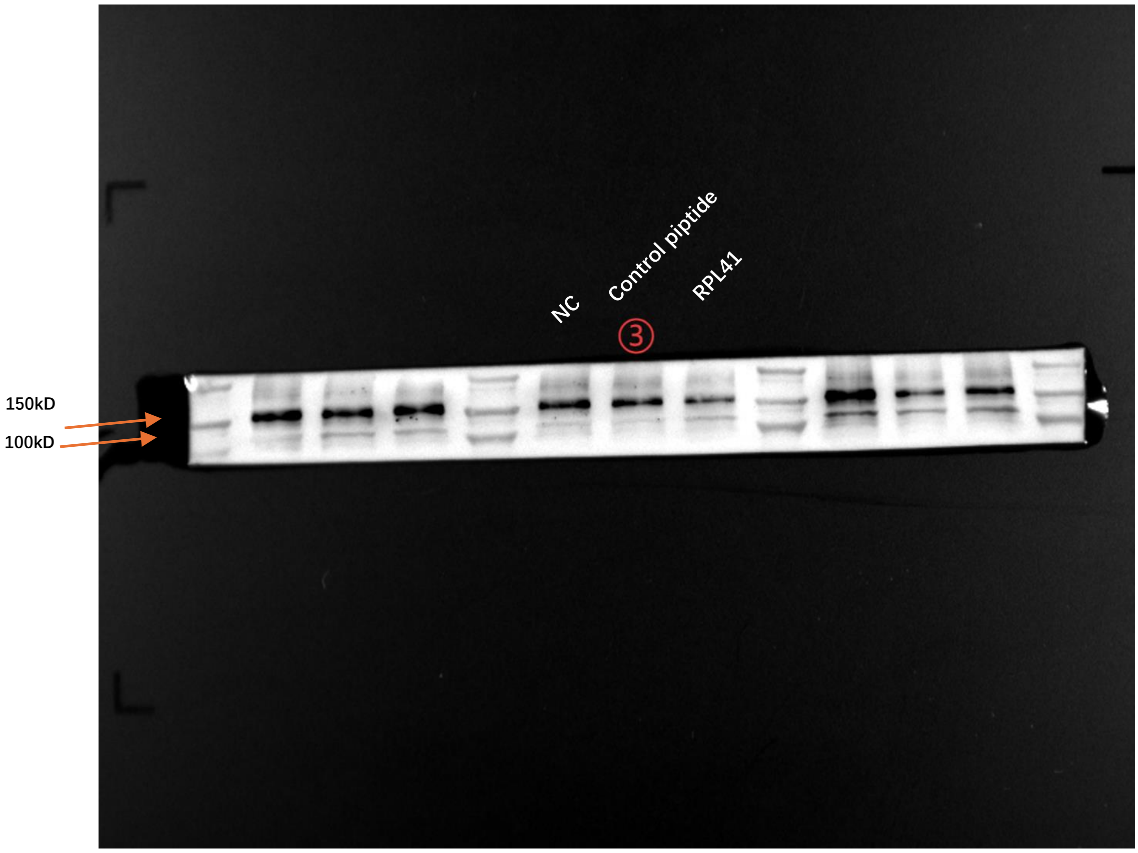

(β-Actin)

The change in β-Actin protein expression was verified by Western blot using a 7.5% separating gel in the Weri-RB1 cells among the control group, control peptide group, and RPL41 intervention group.

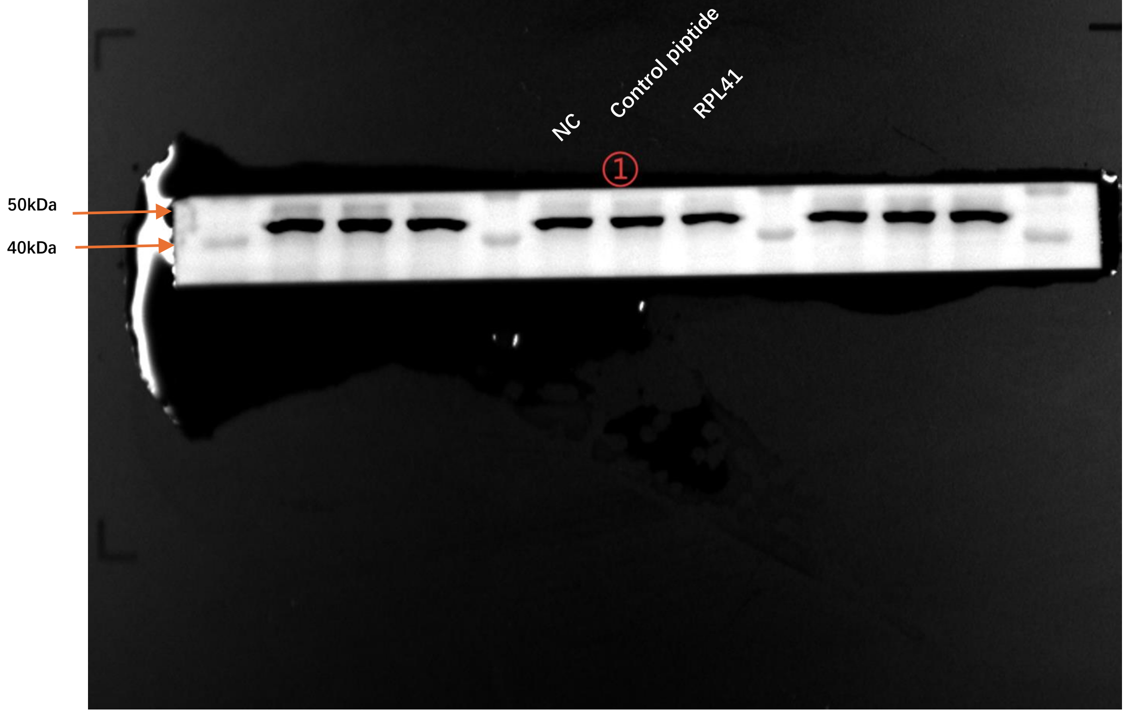

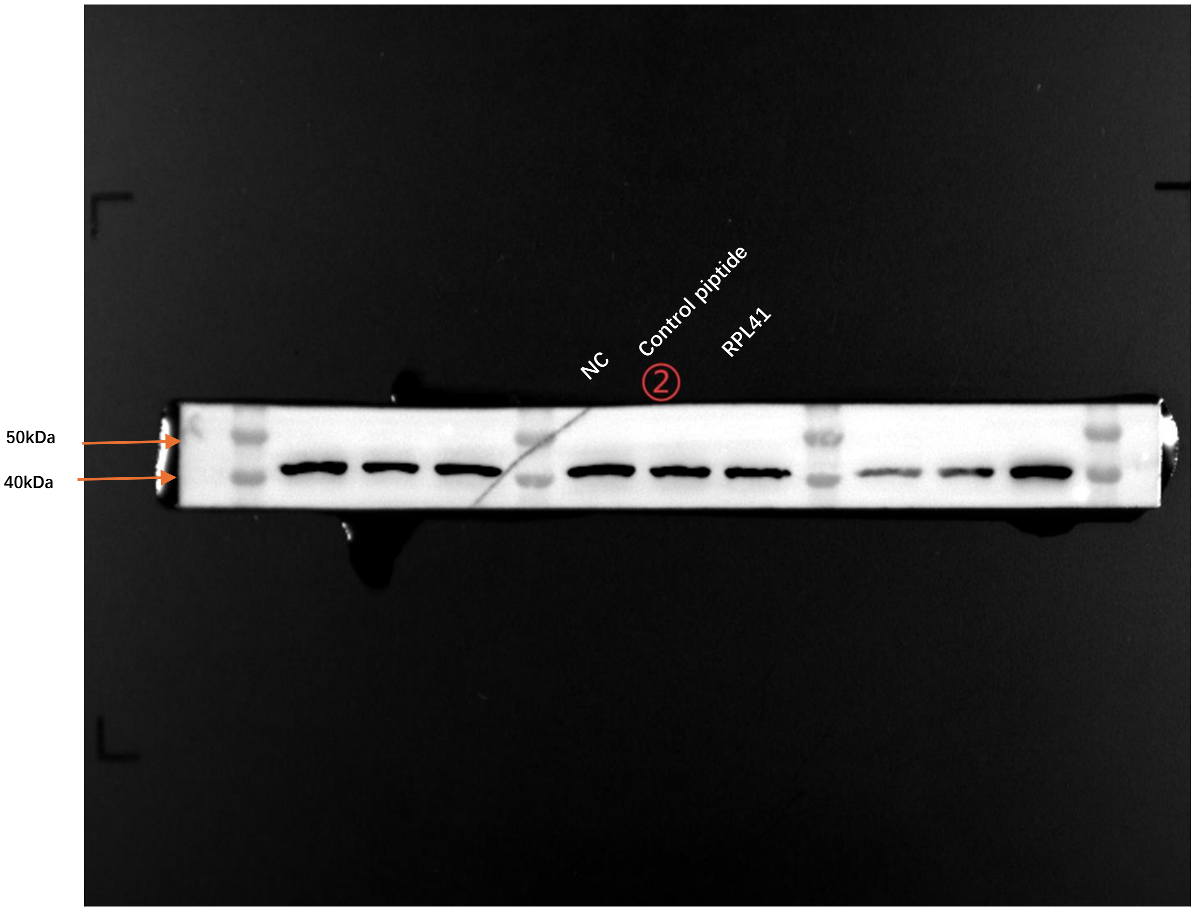
50kDa
40kDa
NC
Control piptide
RPL41
②

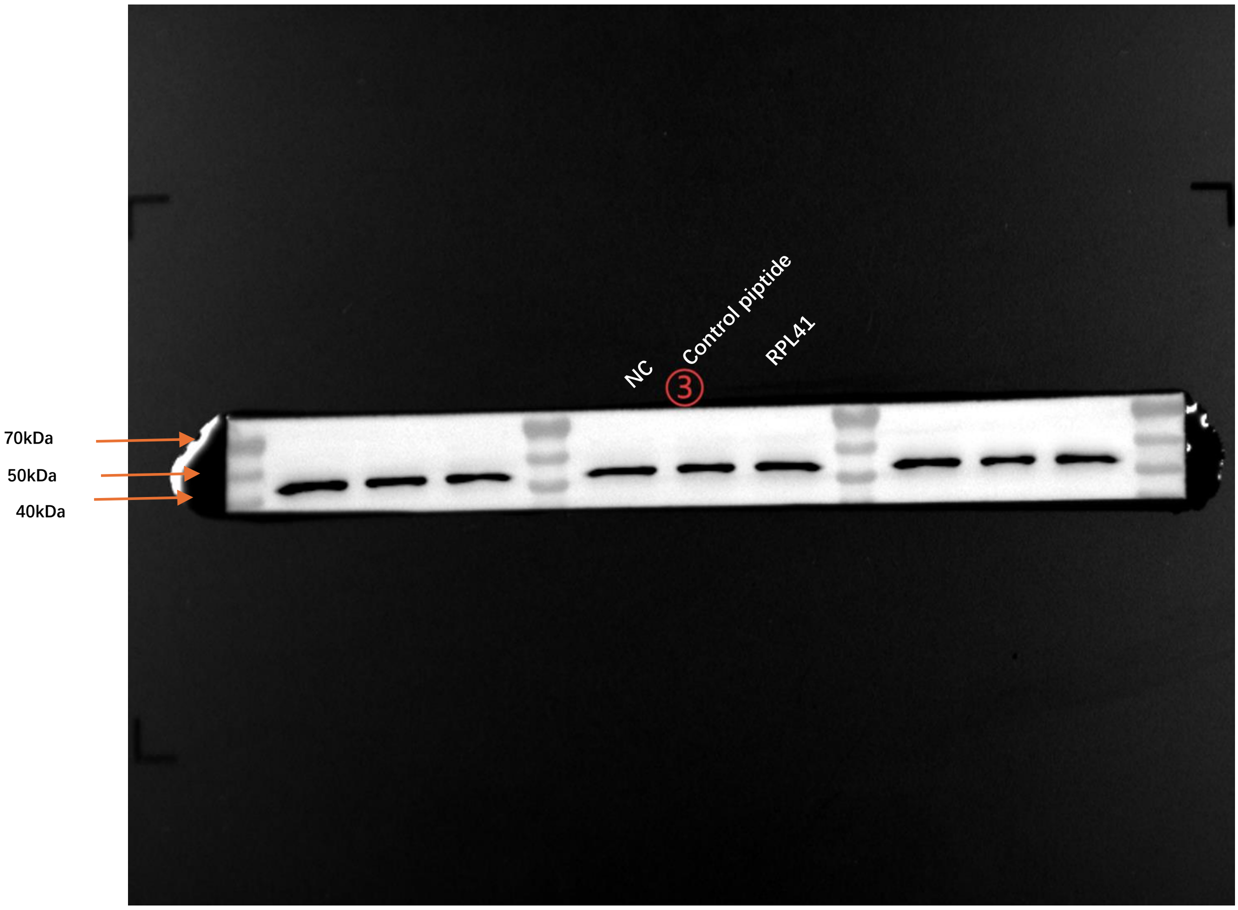
70kDa
50kDa
40kDa
NC
Control piptide
RPL41
③

Weri-RB1(KIF5B)

The change in KIF5B protein expression was verified by Western blot using a 7.5% separating gel in the Weri-RB1 cells among the control group, control peptide group, and RPL41 intervention group.

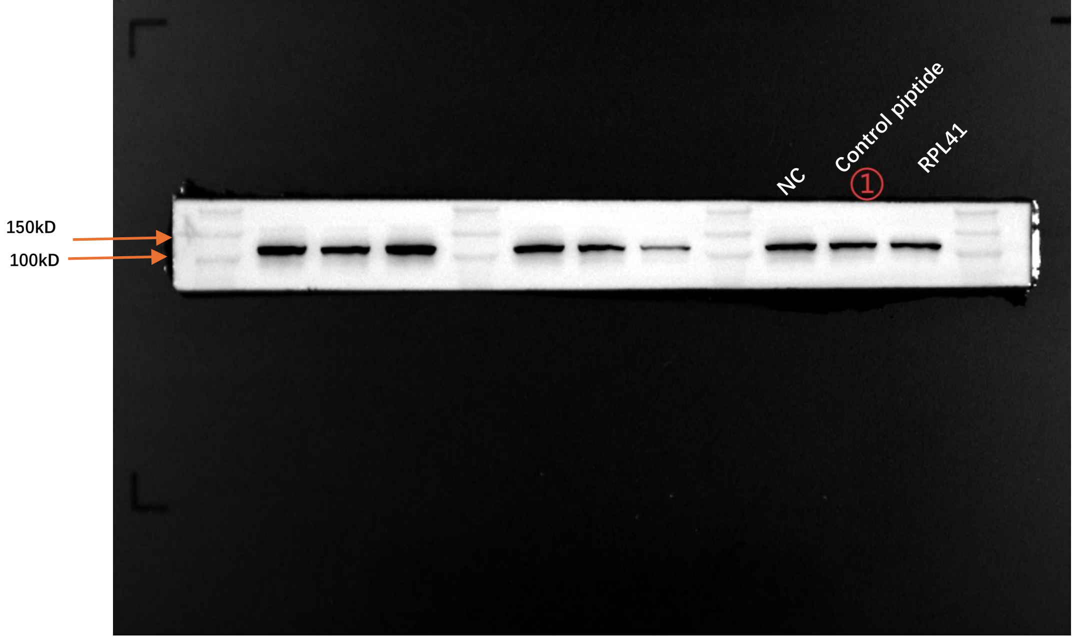

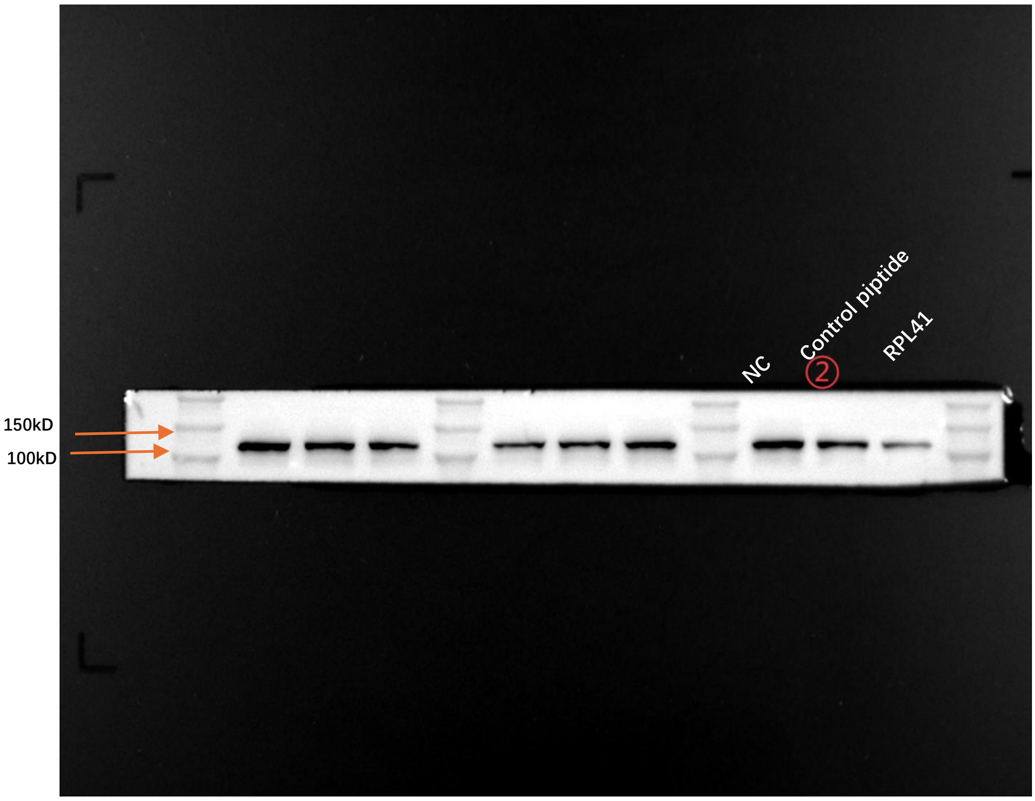

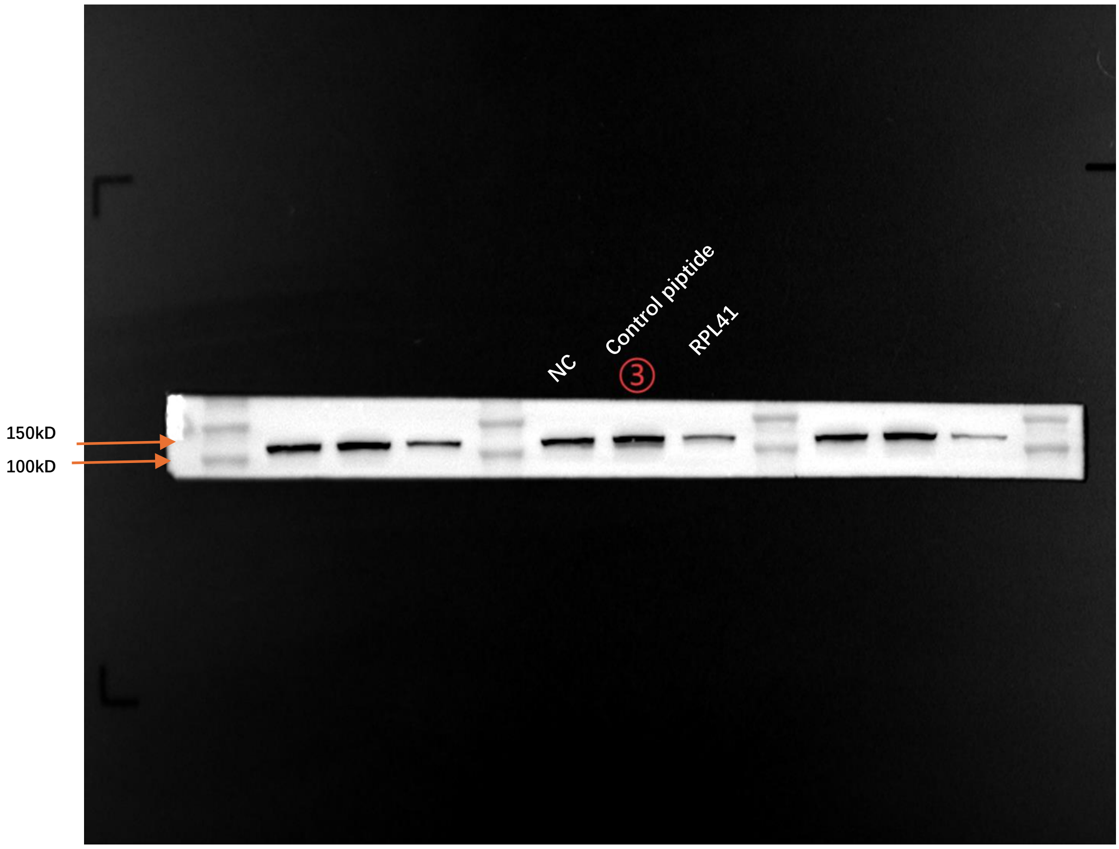

(β-Actin)

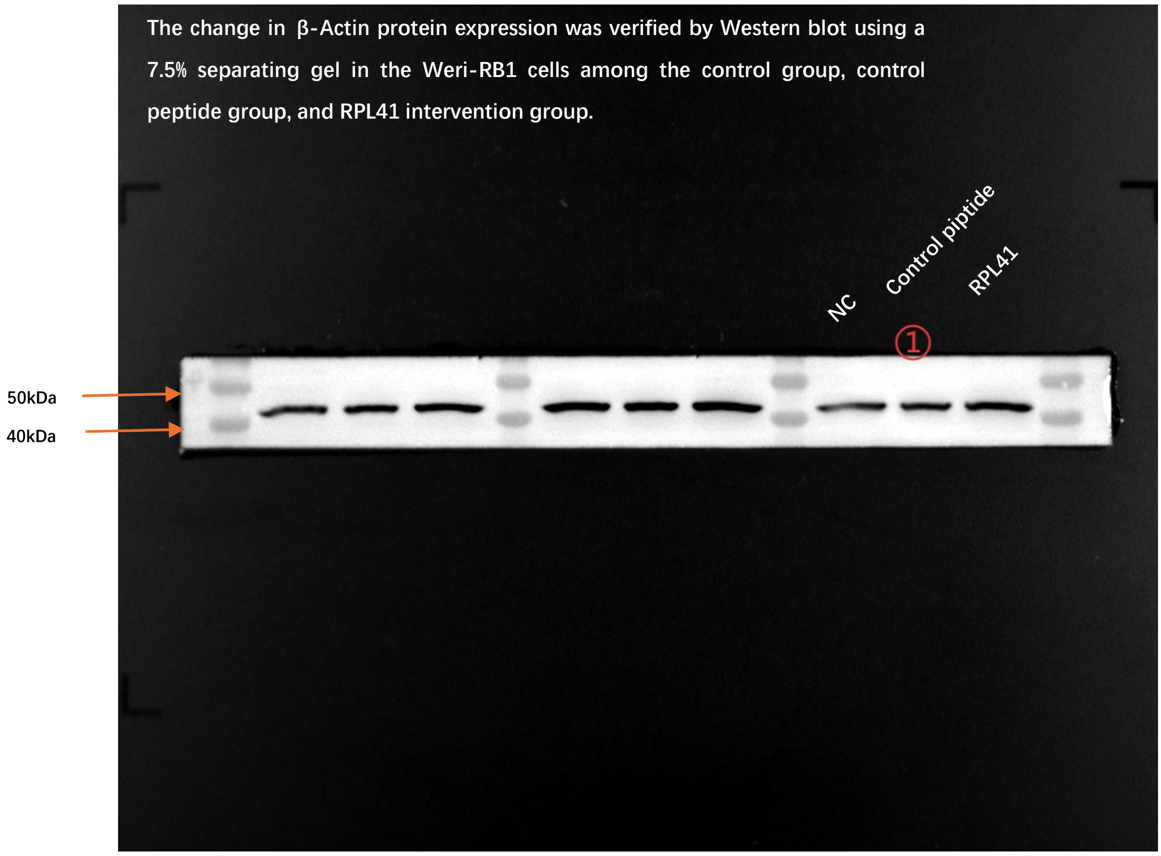

The change in β-Actin protein expression was verified by Western blot using a 7.5% separating gel in the Weri-RB1 cells among the control group, control peptide group, and RPL41 intervention group.

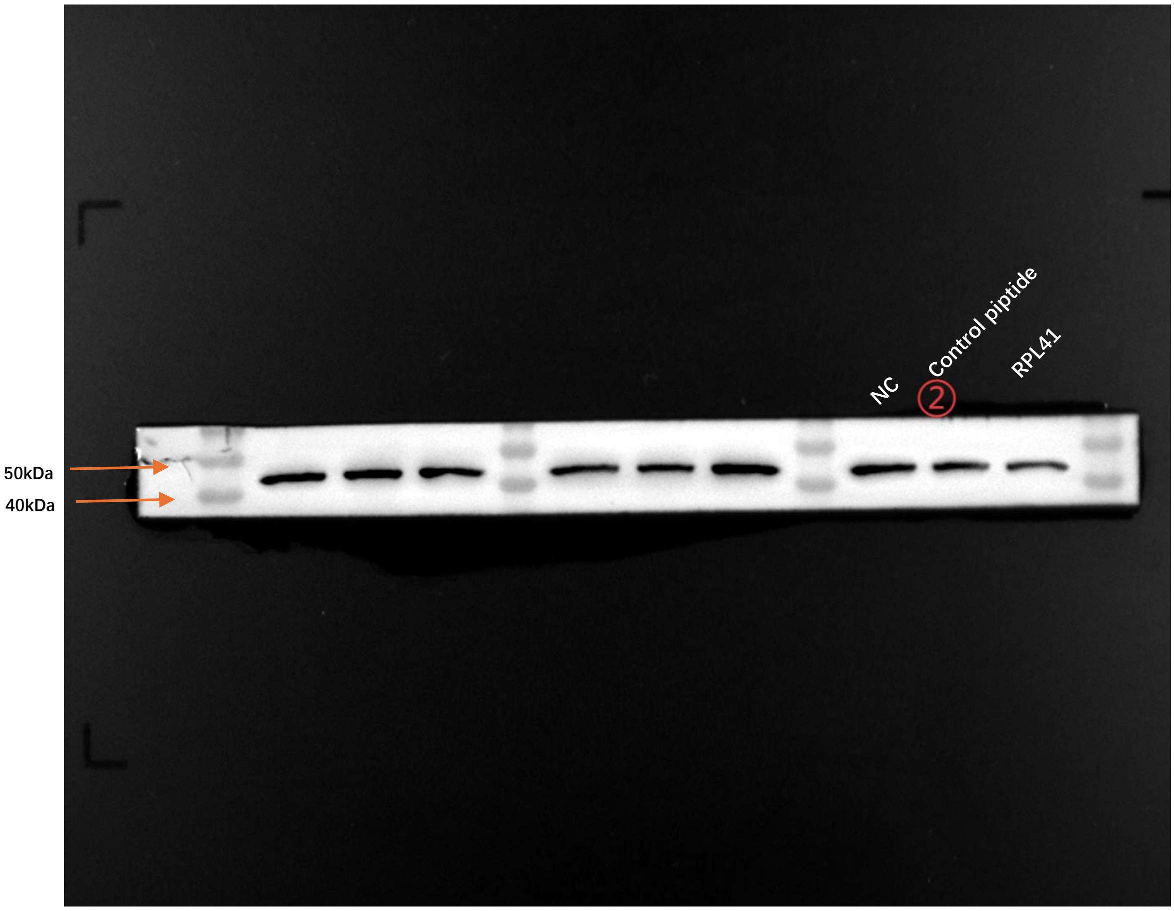
50kDa
40kDa
NC
Control piptide
②
RPL41

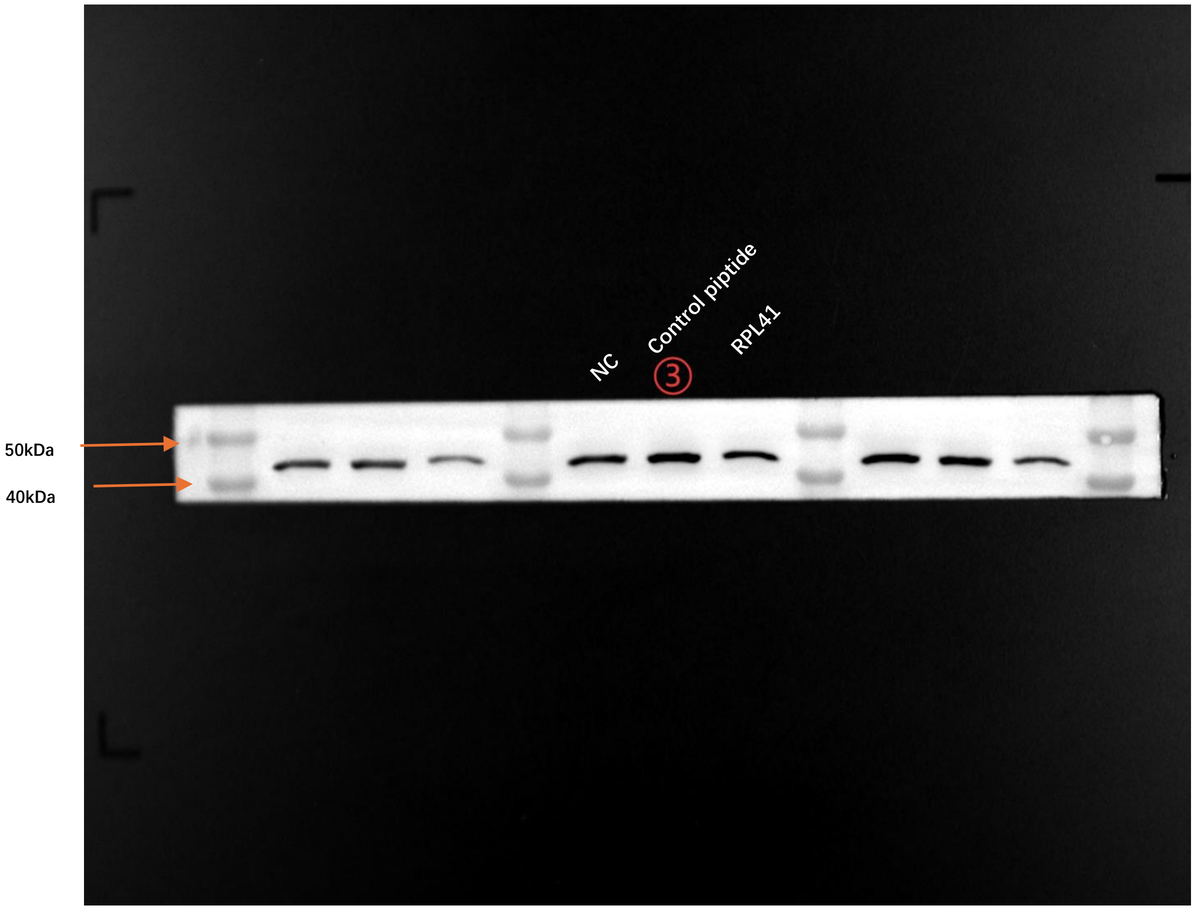
50kDa
40kDa
NC
Control piptide
③
RPL41

Weri-RB1(KLC2)

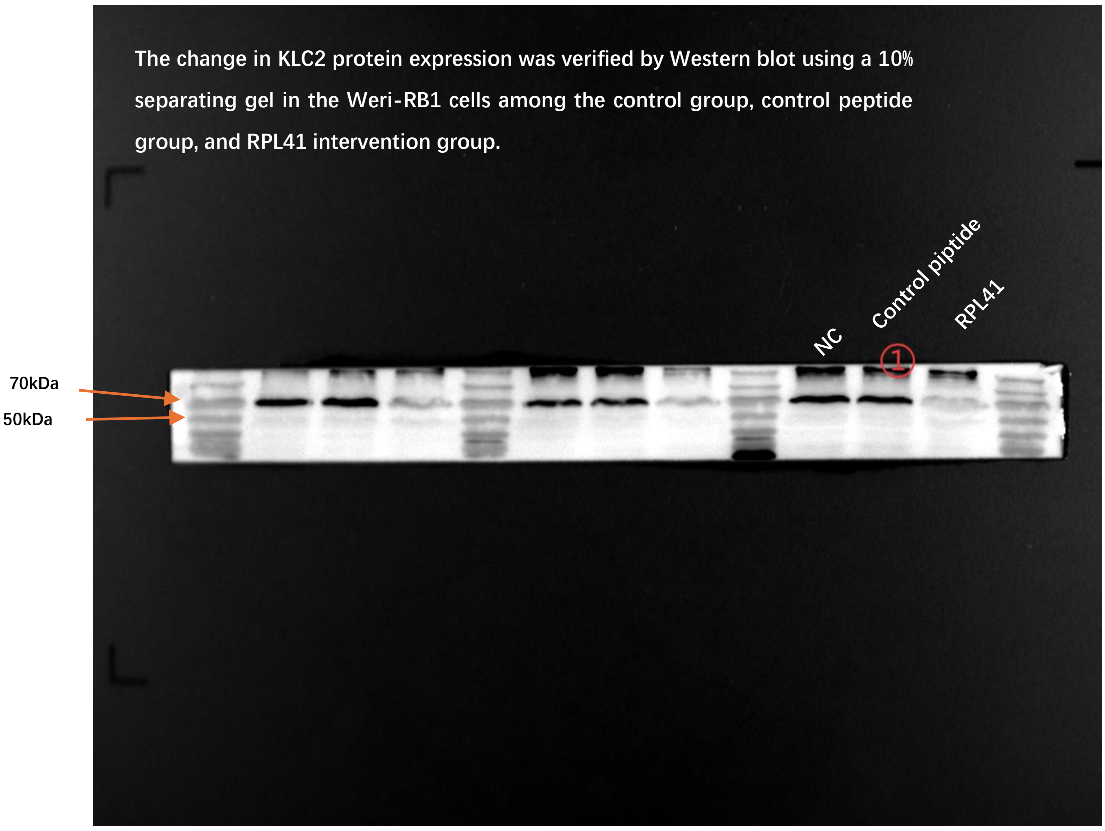

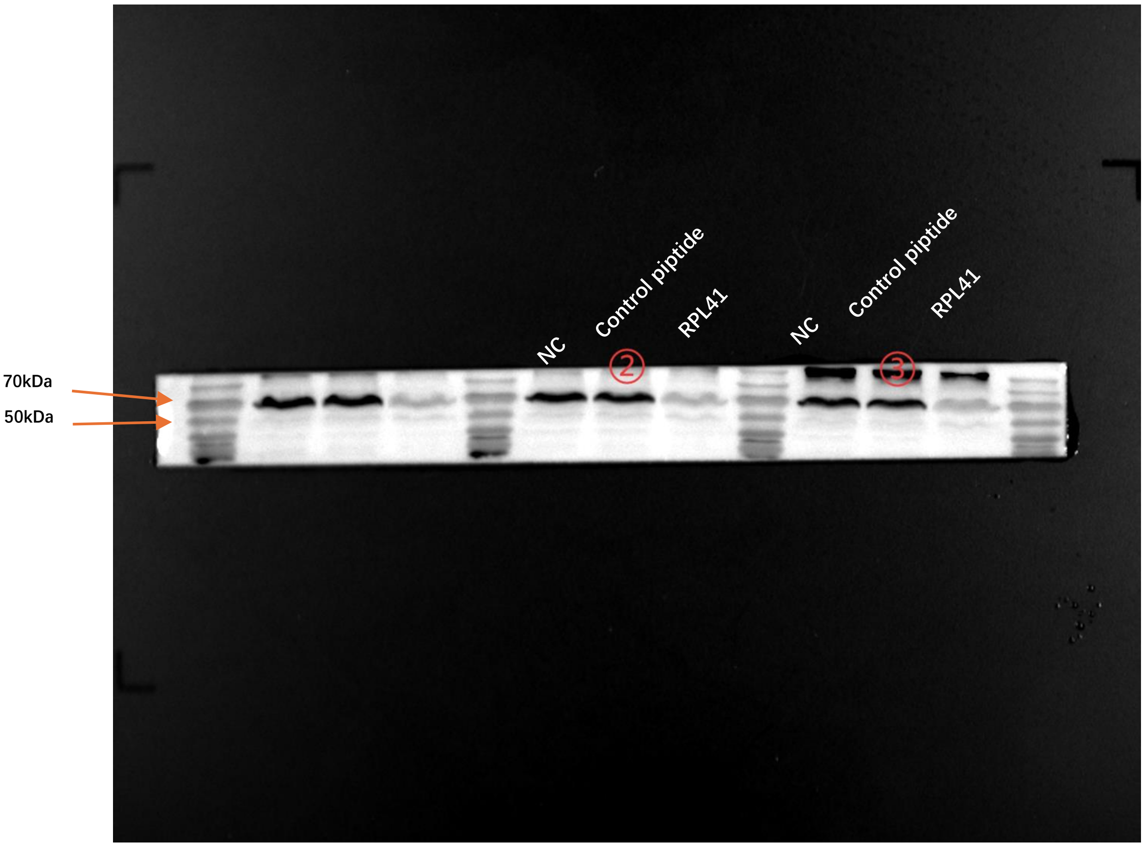

(β-Actin)

The change in β-Actin protein expression was verified by Western blot using a 10% separating gel in the Weri-RB1 cells among the control group, control peptide group, and RPL41 intervention group.

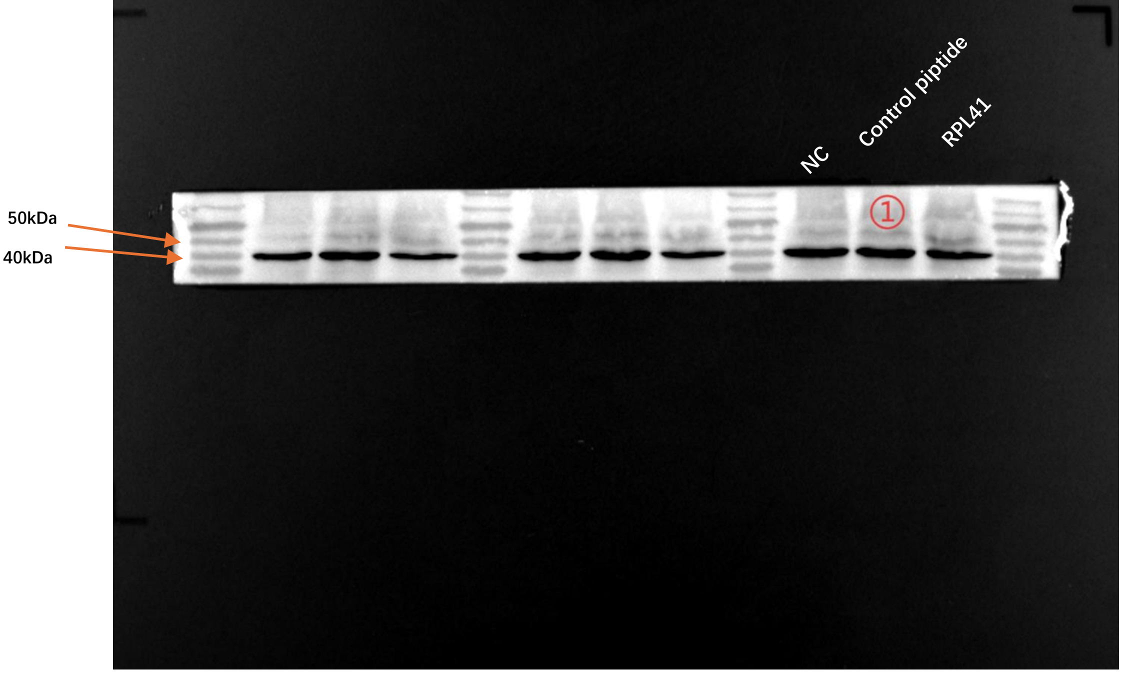

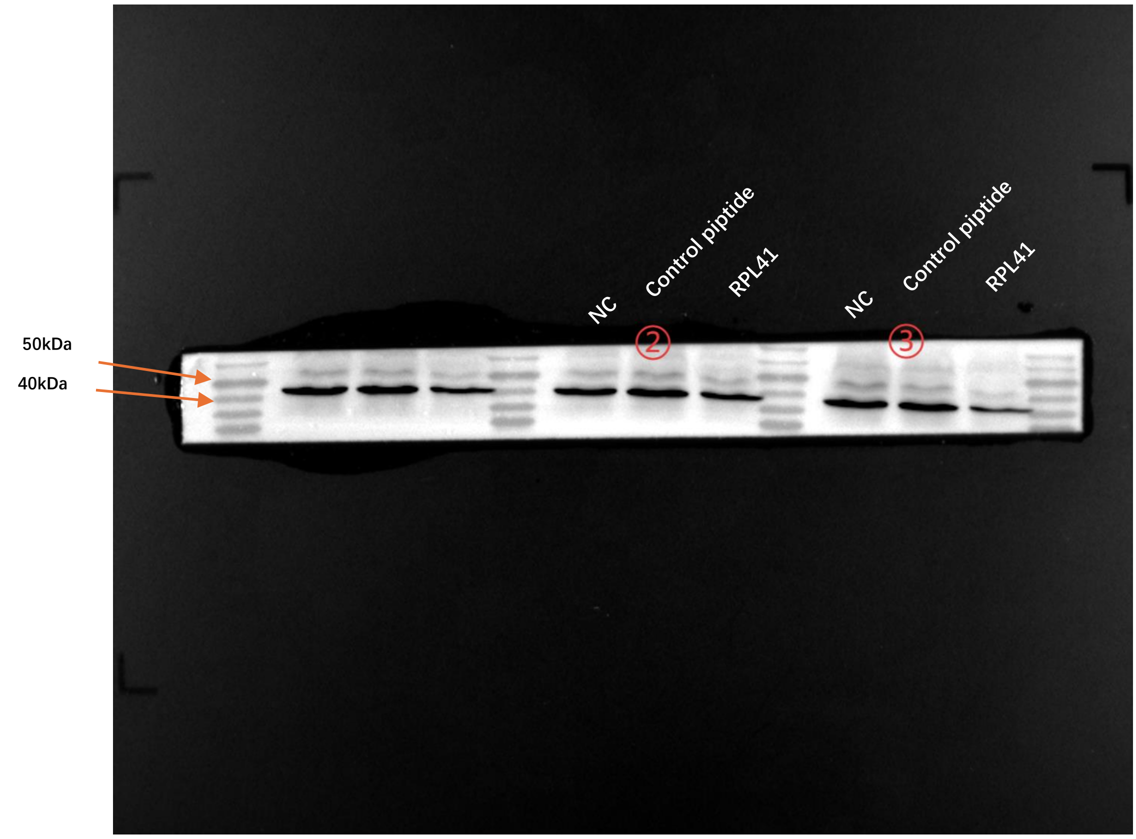

FIGURE-6

Y79(Transfection efficiency-oeARL5B)

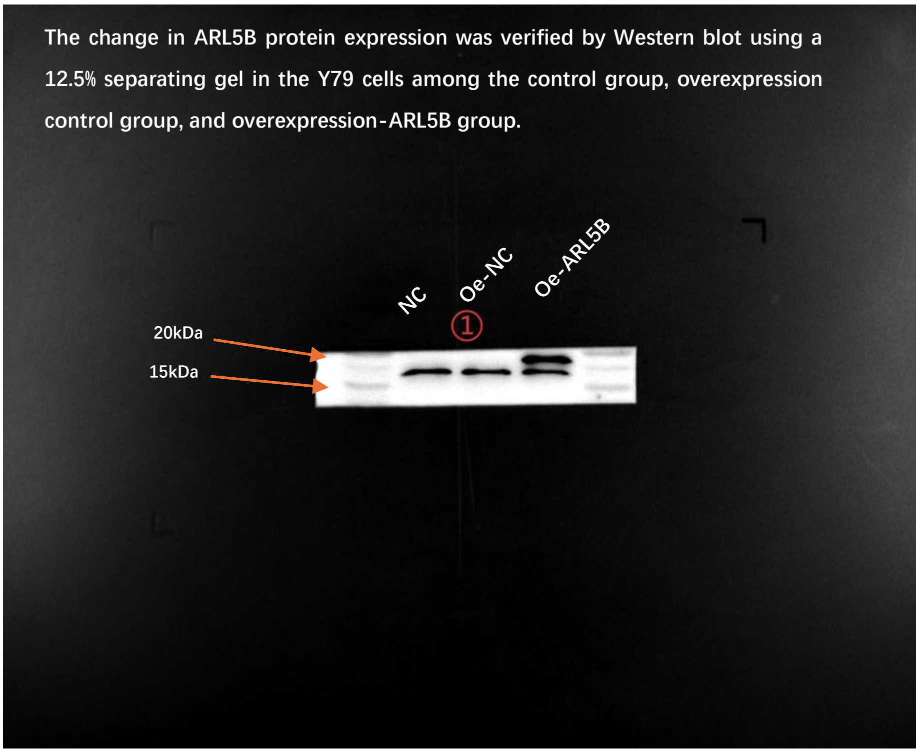

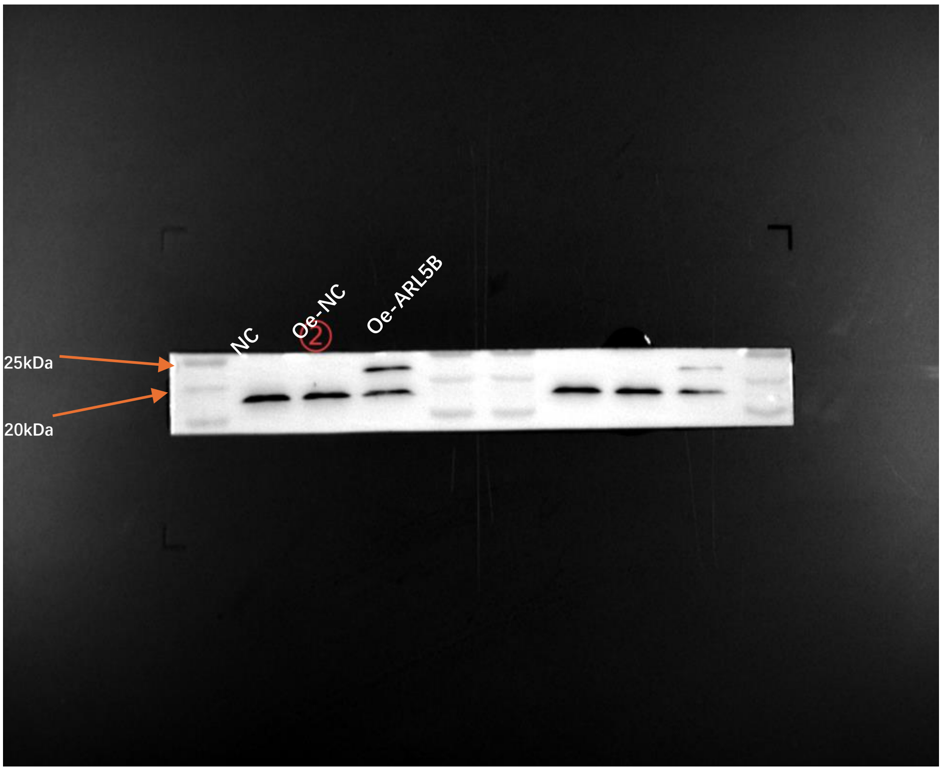

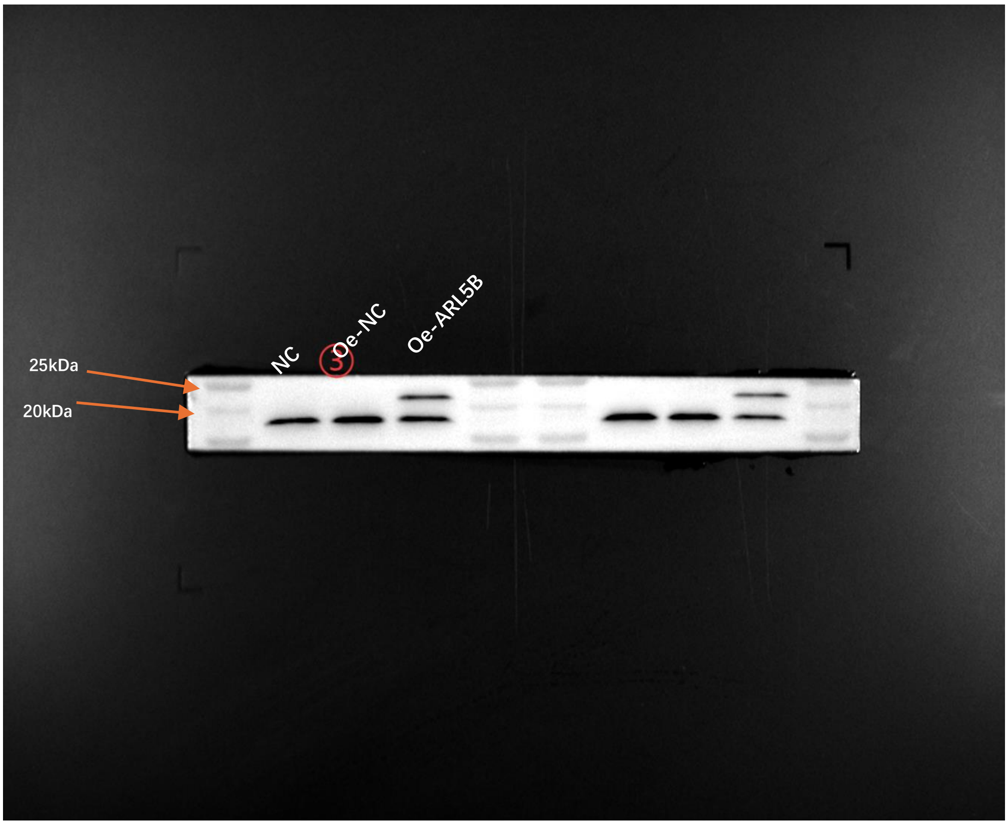
25kDa
20kDa
NC
Oe-NC
Oe-ARL5B
5

(β-Actin)

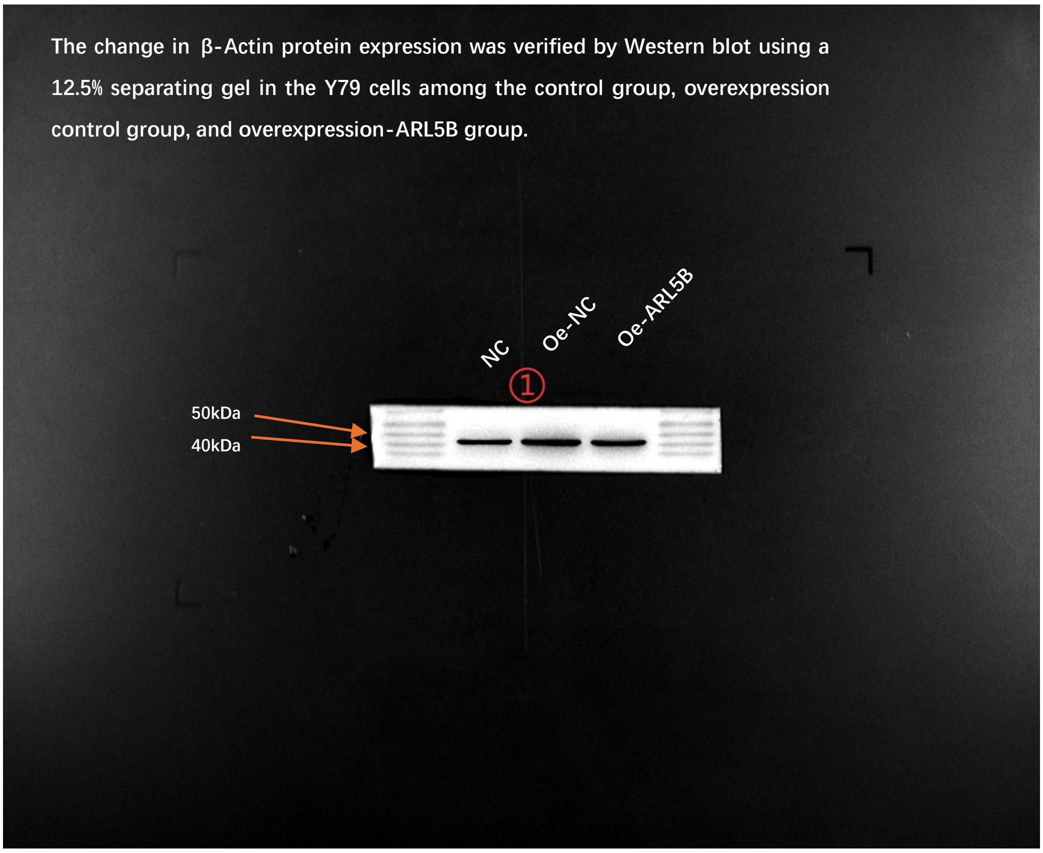
The change in β-Actin protein expression was verified by Western blot using a 12.5% separating gel in the Y79 cells among the control group, overexpression control group, and overexpression-ARL5B group.
NC
Oe-NC
Oe-ARL5B
1
50kDa
40kDa

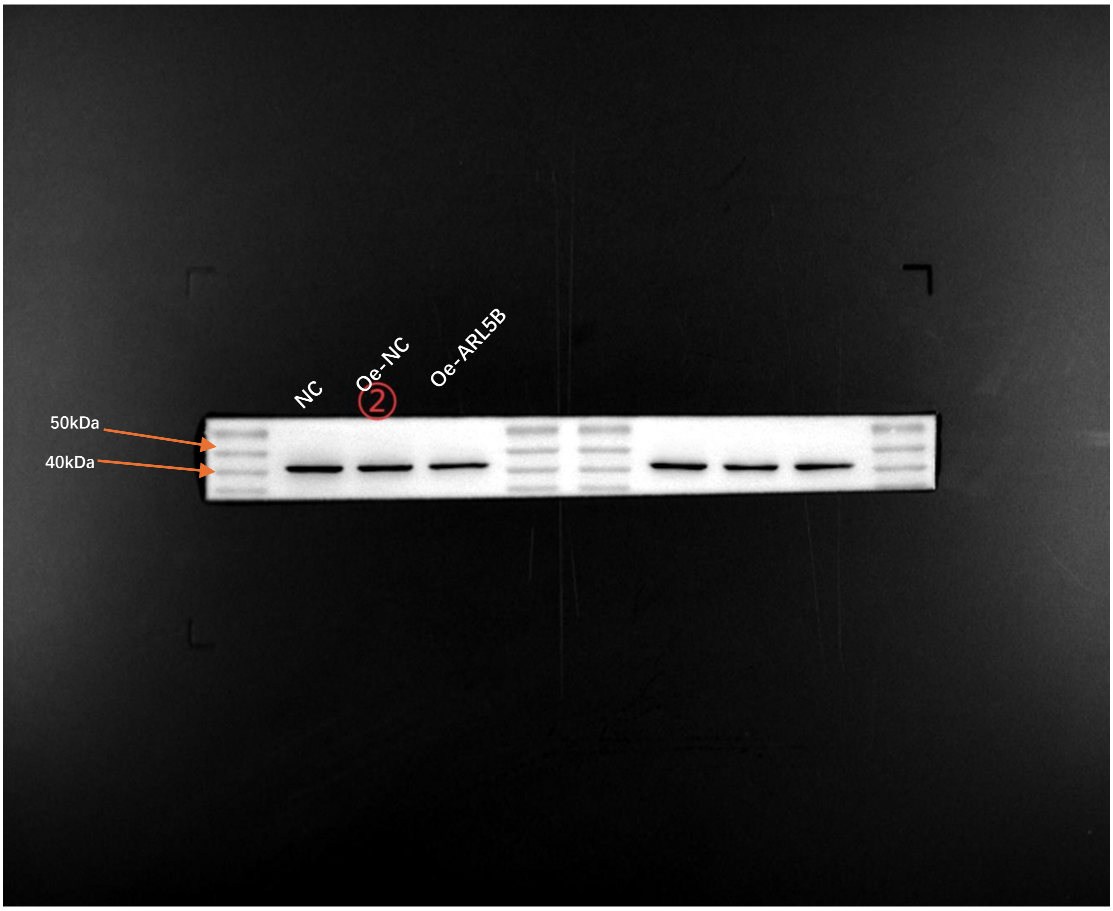
NC
Oe-NC
Oe-ARL5B
2
50kDa
40kDa

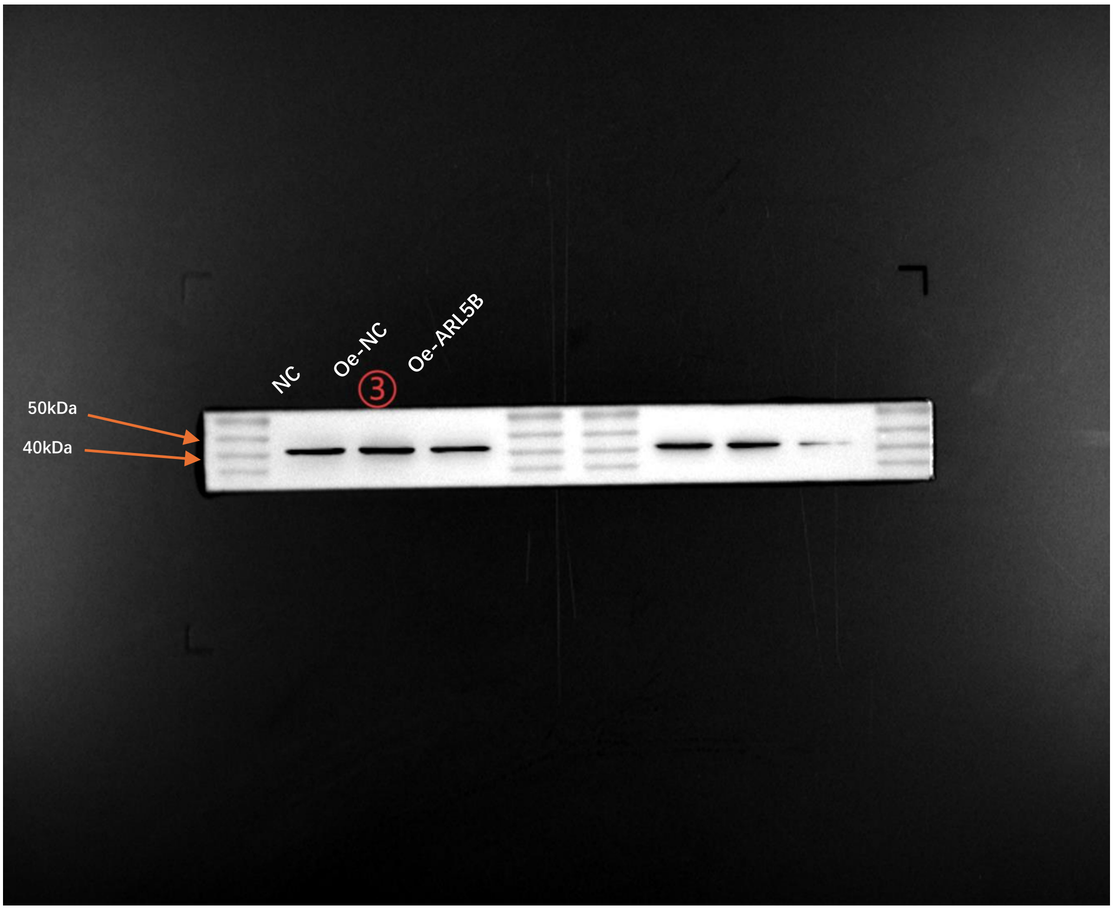
NC
Oe-NC
Oe-ARL5B
3
50kDa
40kDa

Weri-RB1 (Transfection efficiency-oeARL5B)

The change in ARL5B protein expression was verified by Western blot using a 12.5% separating gel in the Weri-RB1 cells among the control group, overexpression control group, and overexpression-ARL5B group.

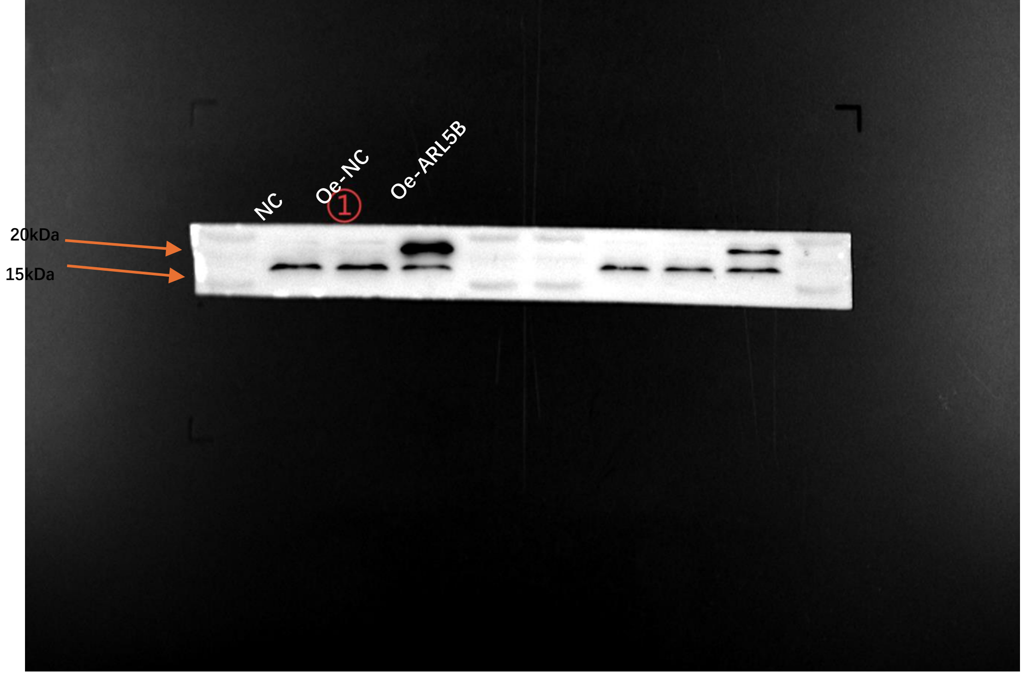

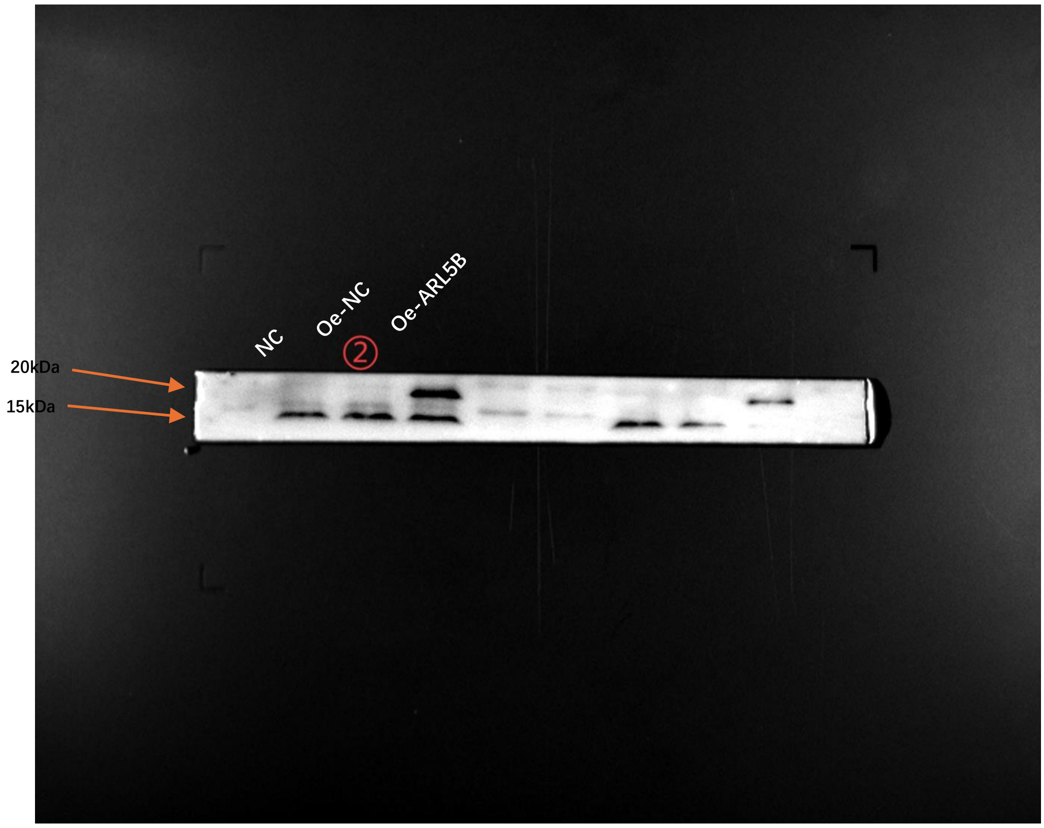

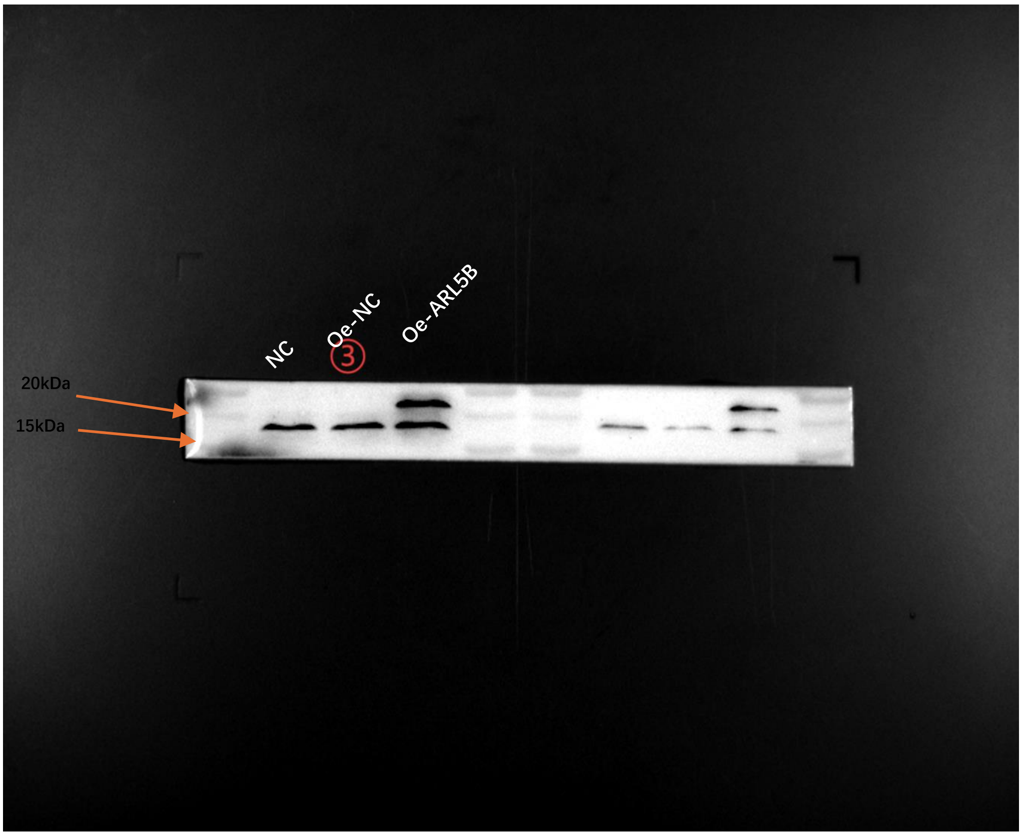

(β-Actin)

The change in β-Actin protein expression was verified by Western blot using a 12.5% separating gel in the Weri-RB1 cells among the control group, overexpression control group, and overexpression-ARL5B group.

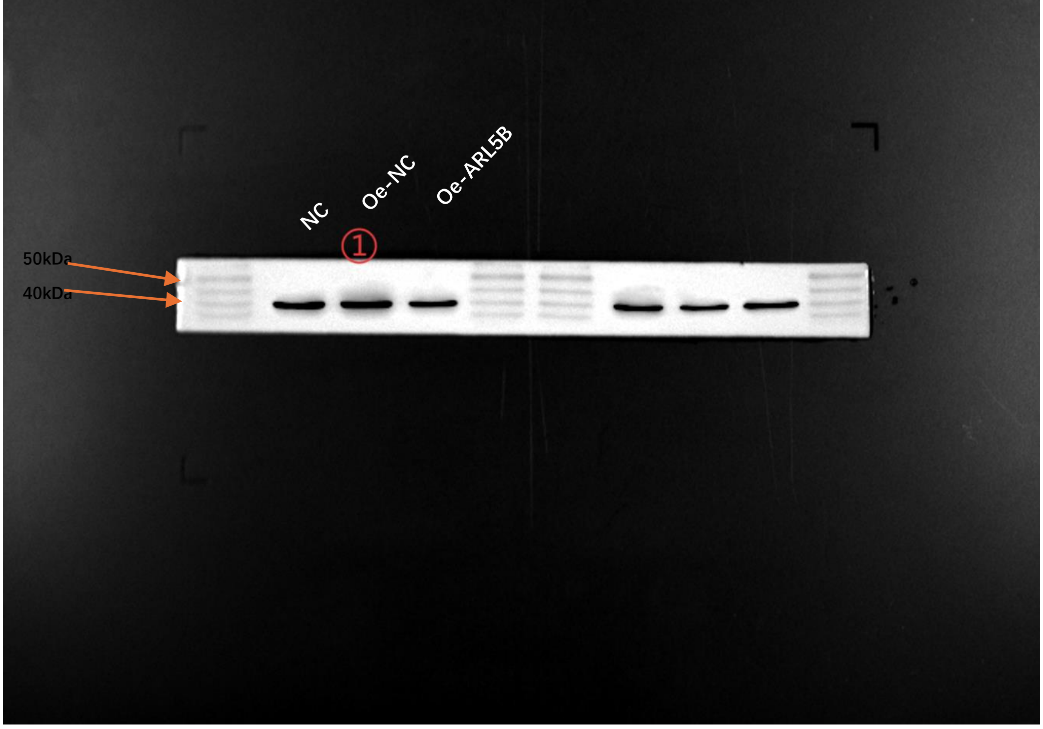

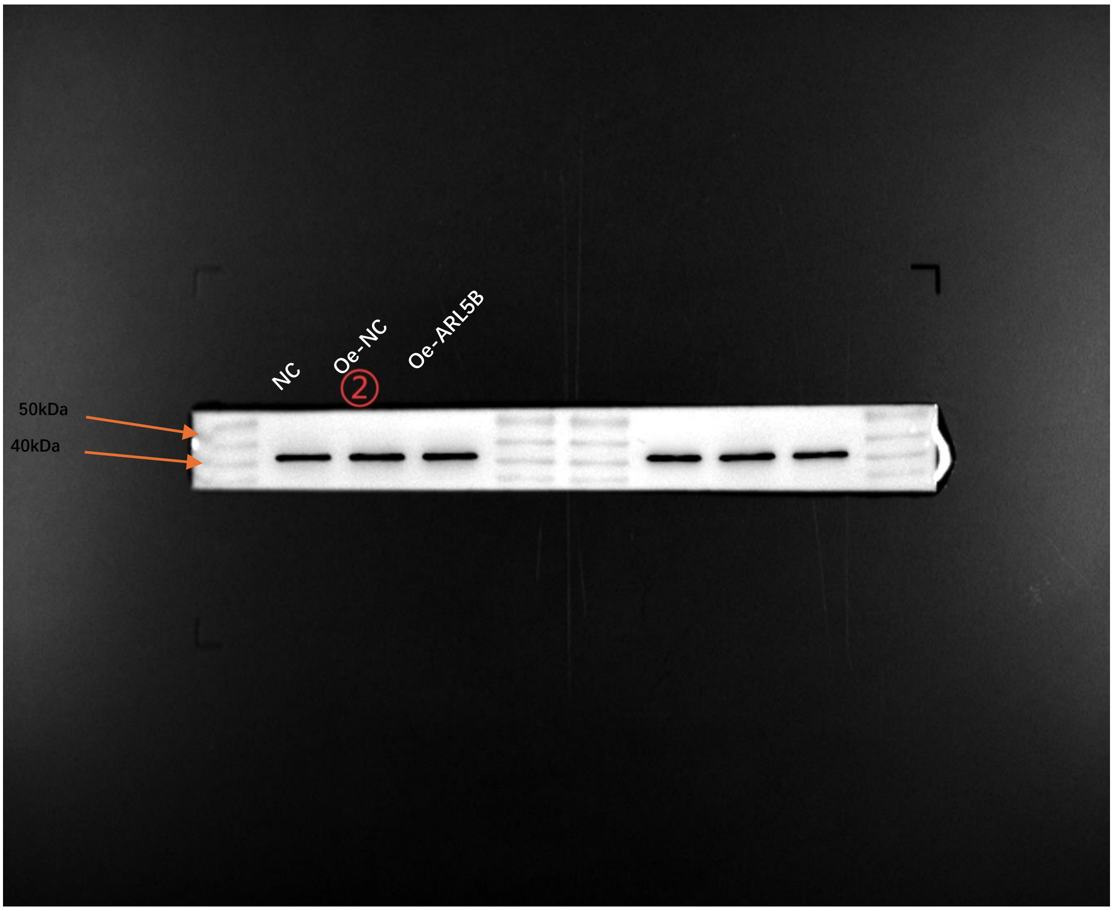
NC
Oe-NC
Oe-ARL5B
②
50kDa
40kDa

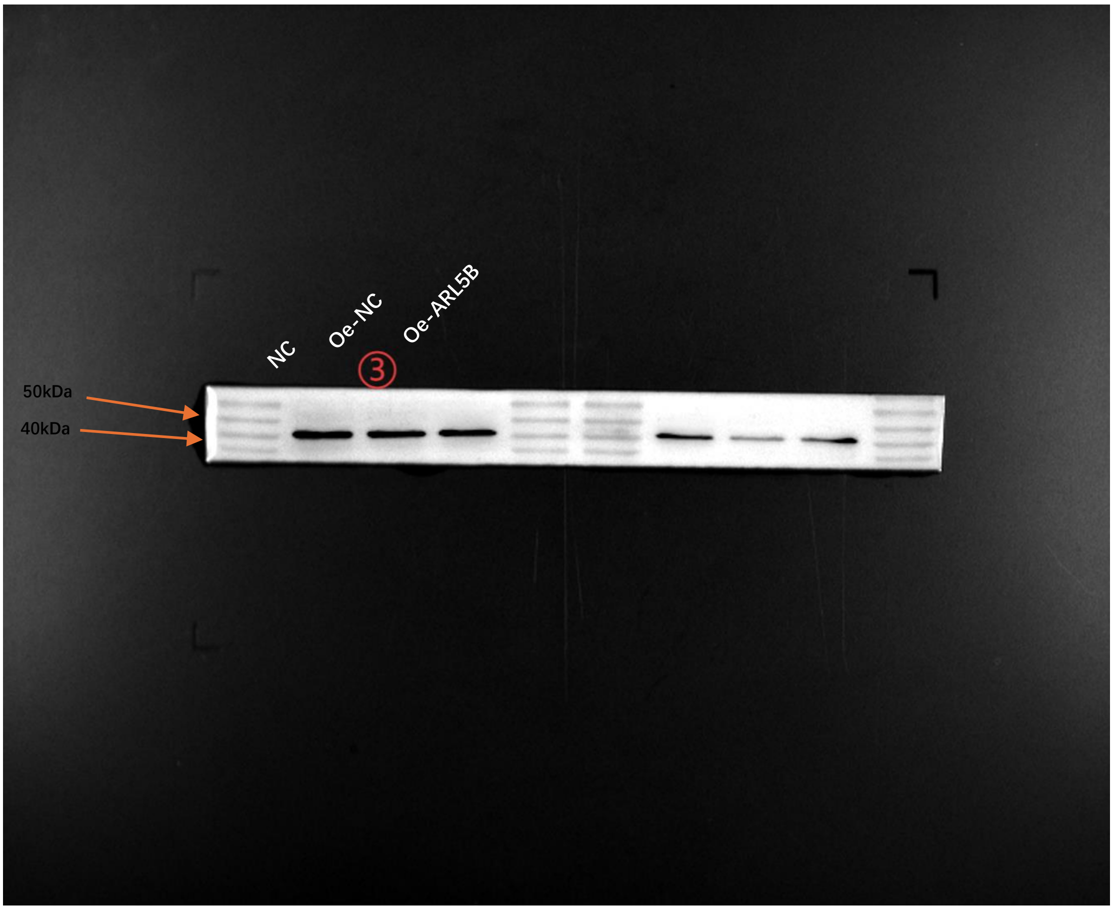
NC
Oe-NC
Oe-ARL5B
③
50kDa
40kDa

Y79(ARL5B)

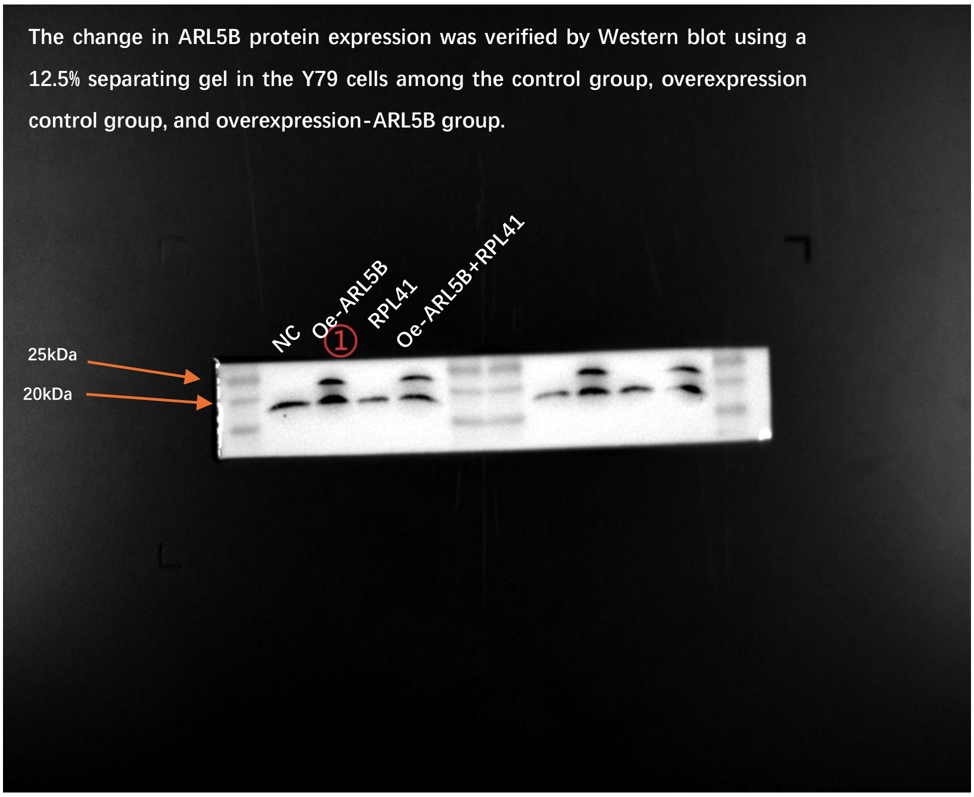
The change in ARL5B protein expression was verified by Western blot using a 12.5% separating gel in the Y79 cells among the control group, overexpression control group, and overexpression-ARL5B group.
NC
Oe-ARL5B
RPL41
Oe-ARL5B+RPL41
①
25kDa
20kDa

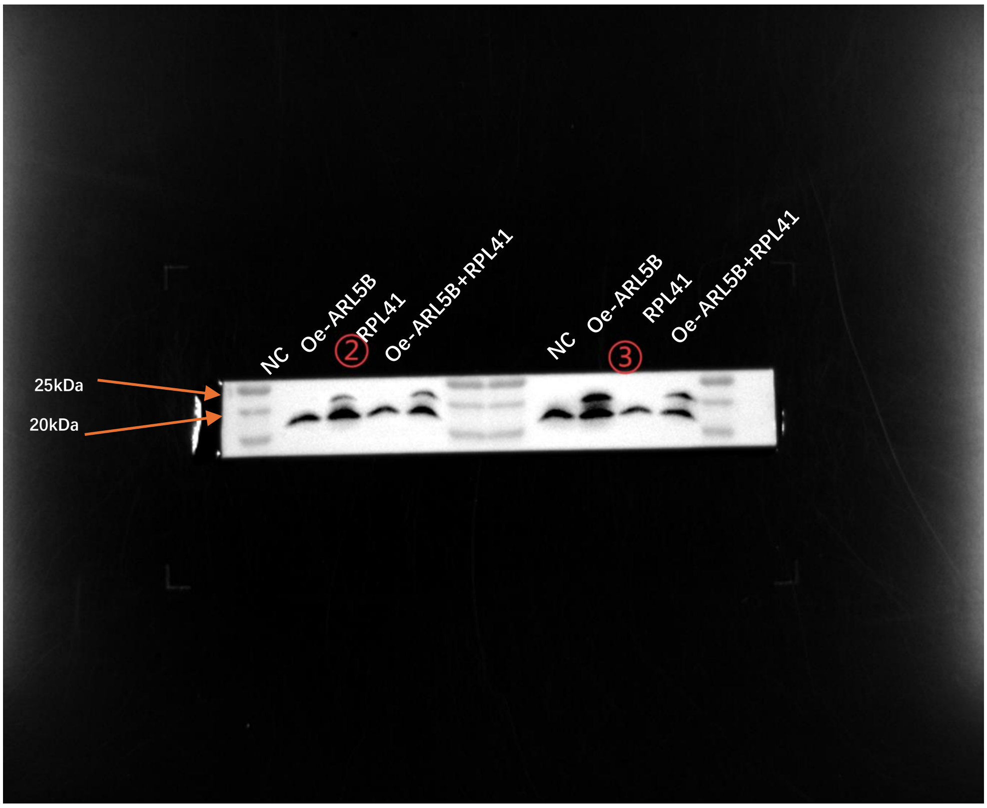
NC
Oe-ARL5B
RPL41
Oe-ARL5B+RPL41
②
NC
Oe-ARL5B
RPL41
Oe-ARL5B+RPL41
③
25kDa
20kDa

(β-Actin)

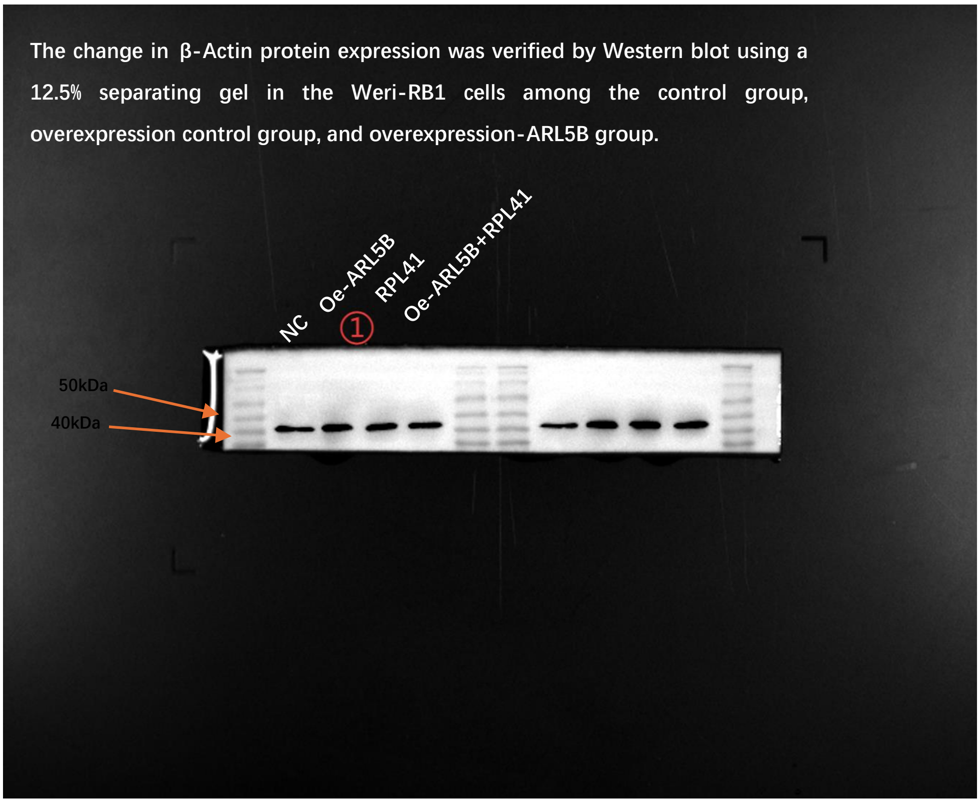
The change in β-Actin protein expression was verified by Western blot using a 12.5% separating gel in the Weri-RB1 cells among the control group, overexpression control group, and overexpression-ARL5B group.
NC
Oe-ARL5B
RPL41
Oe-ARL5B+RPL41
①
50kDa
40kDa

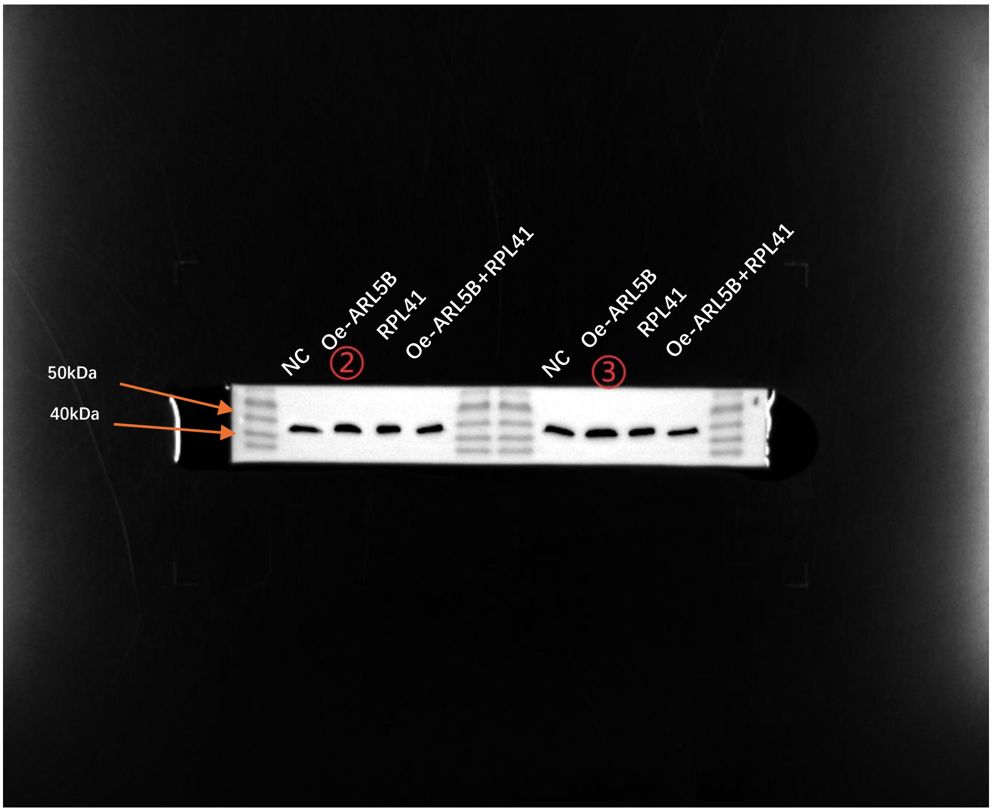
NC
Oe-ARL5B
RPL41
Oe-ARL5B+RPL41
②
NC
Oe-ARL5B
RPL41
Oe-ARL5B+RPL41
③
50kDa
40kDa

(MERGE-1)

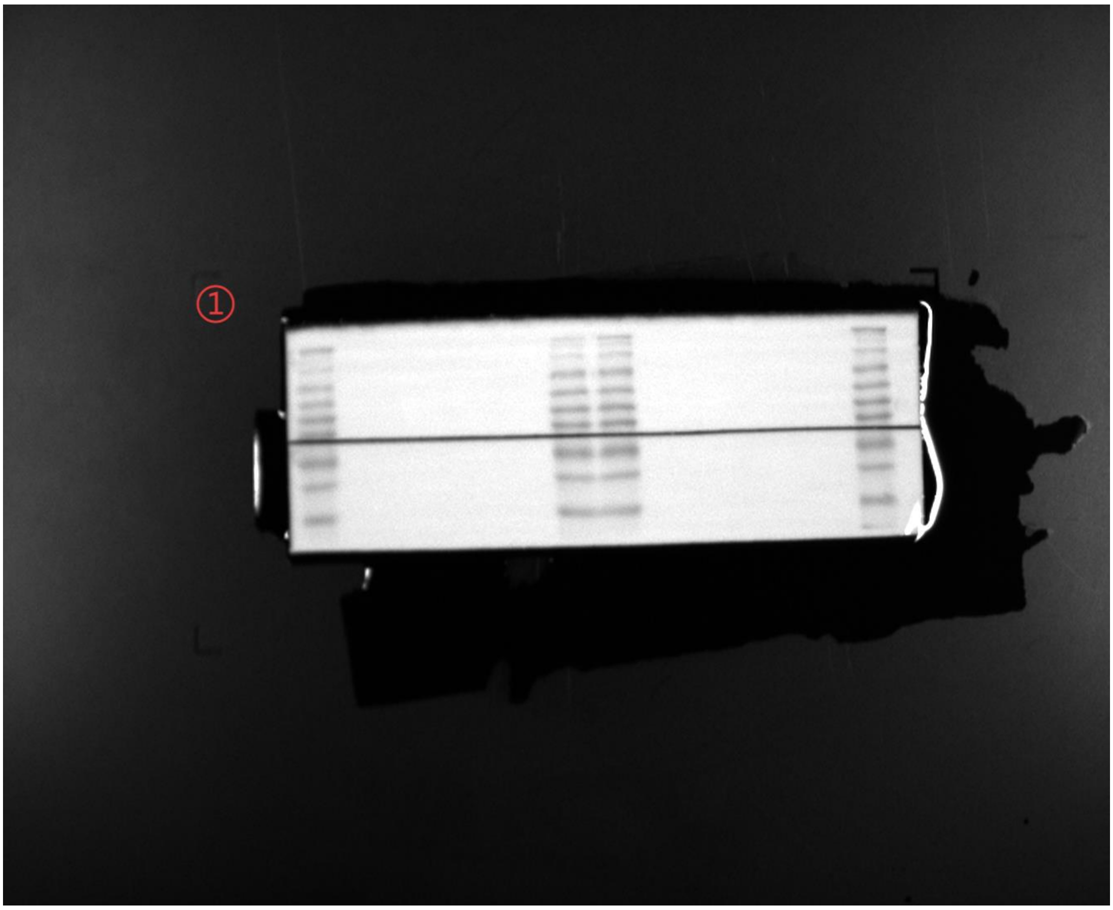

Y79(SKIP)

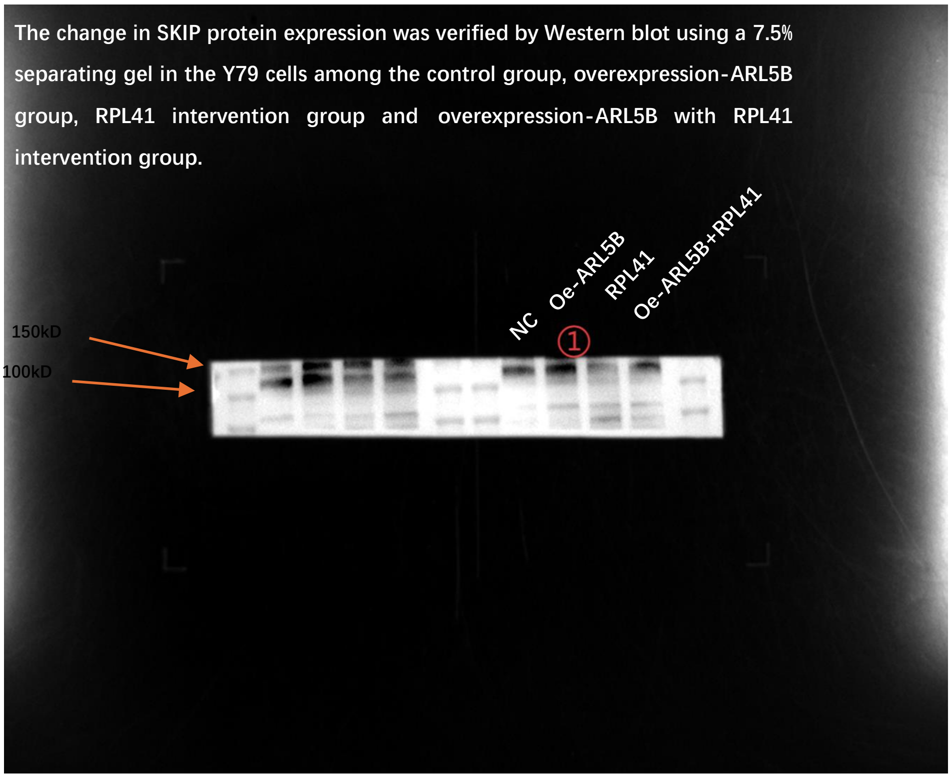

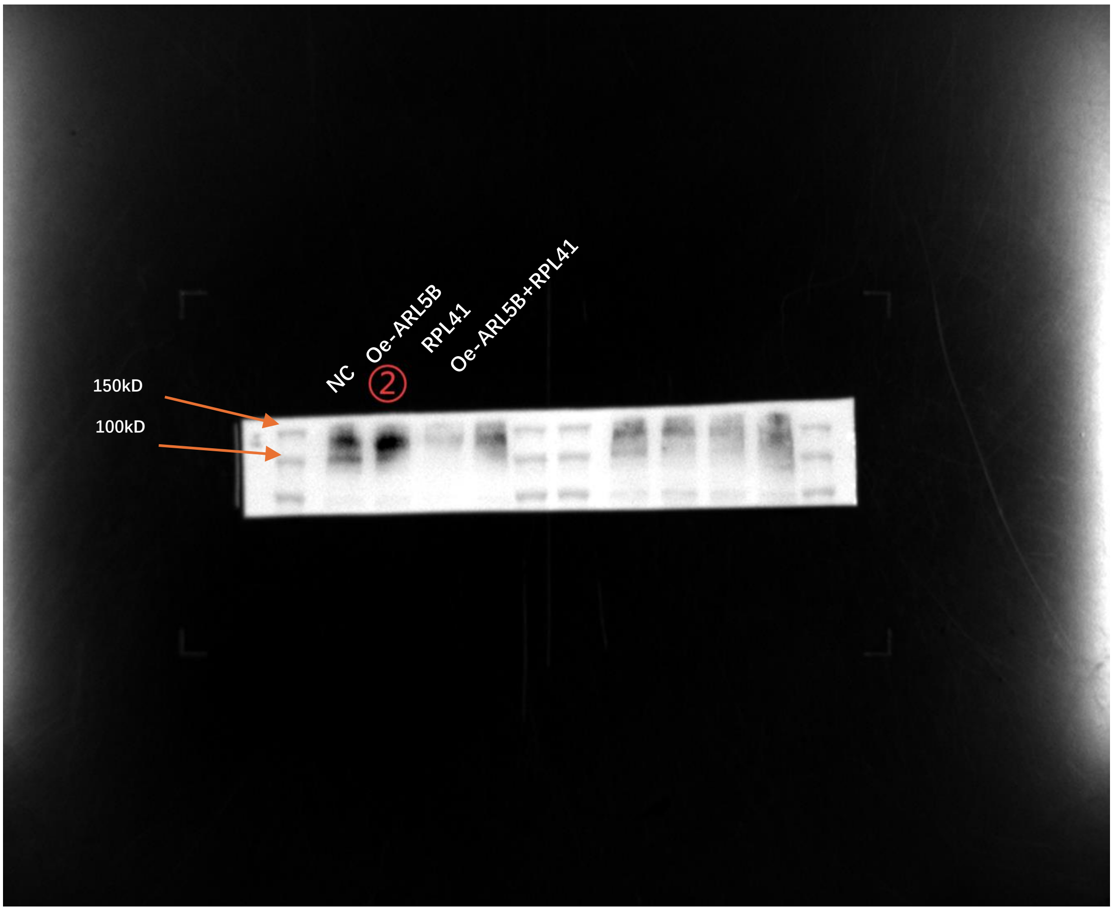
150kD
100kD
NC
Oe-ARL5B
RPL41
Oe-ARL5B+RPL41
2

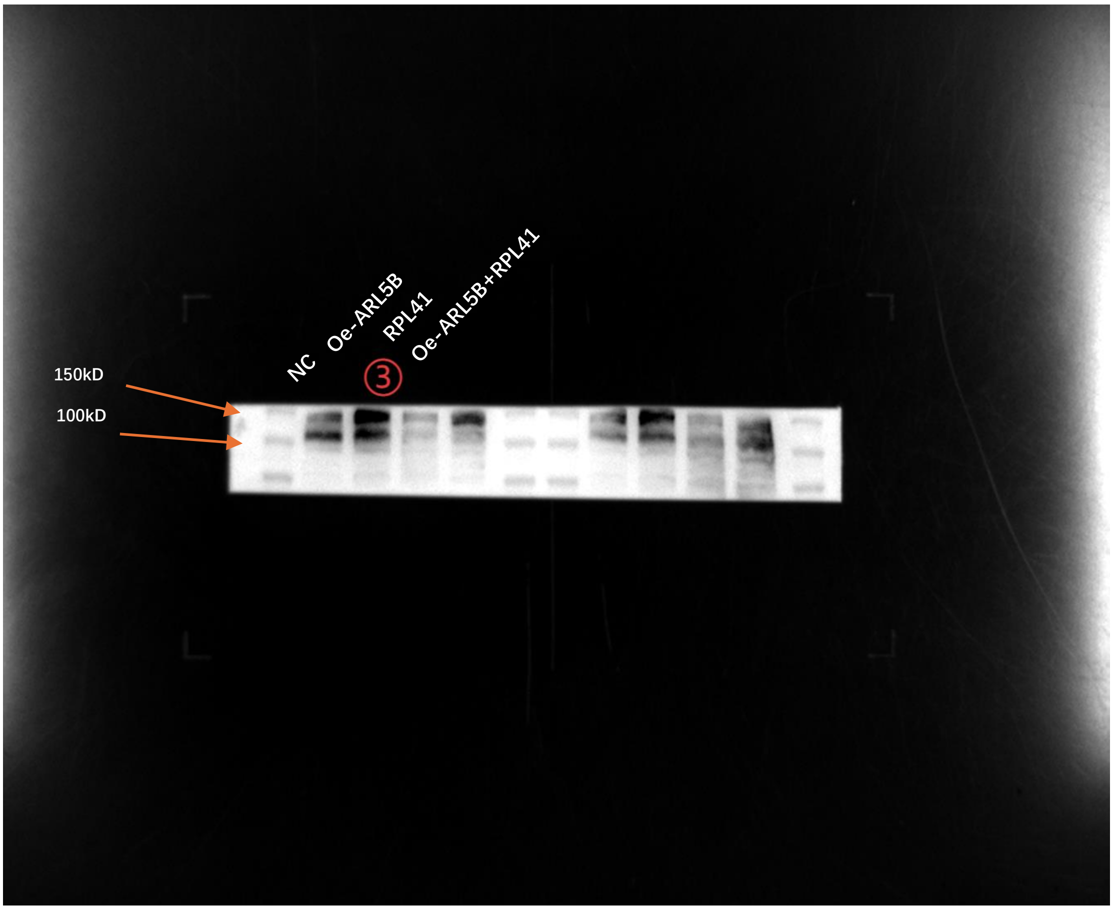
150kD
100kD
NC
Oe-ARL5B
RPL41
Oe-ARL5B+RPL41
3

(β-Actin)

The change in β-Actin protein expression was verified by Western blot using a 7.5% separating gel in the Y79 cells among the control group, overexpression-ARL5B group, RPL41 intervention group and overexpression-ARL5B with RPL41 intervention group.

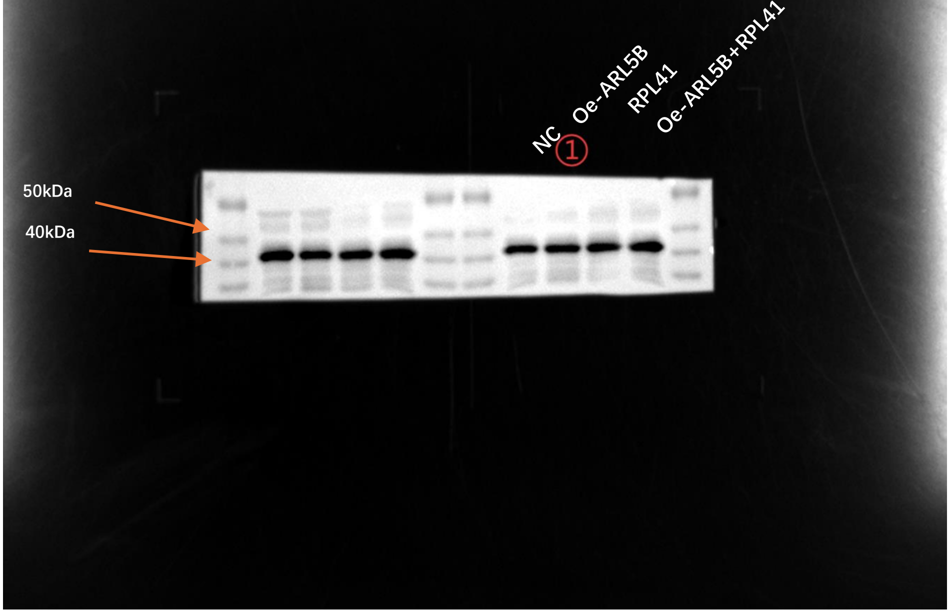

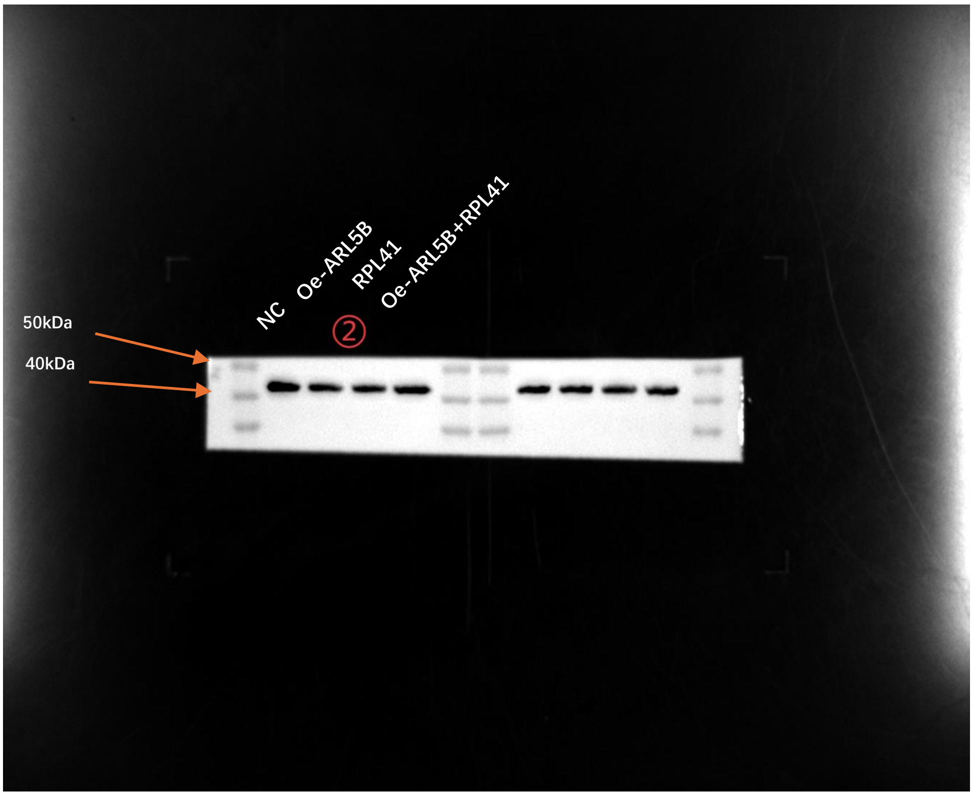

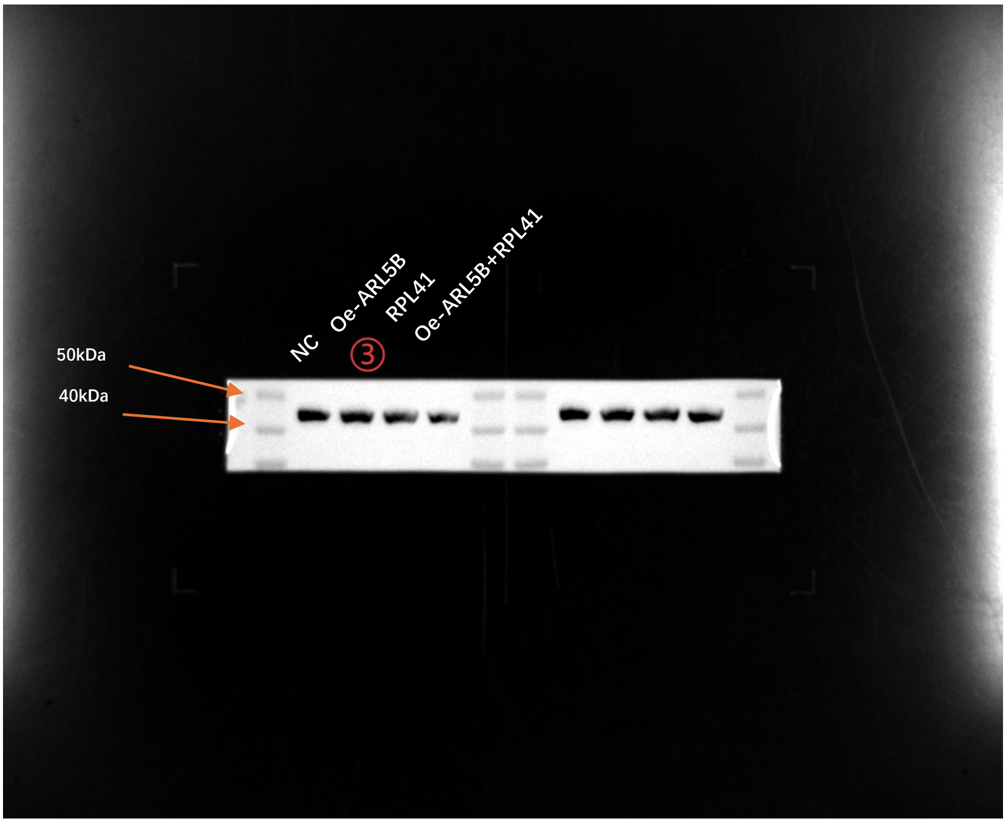

(MERGE)

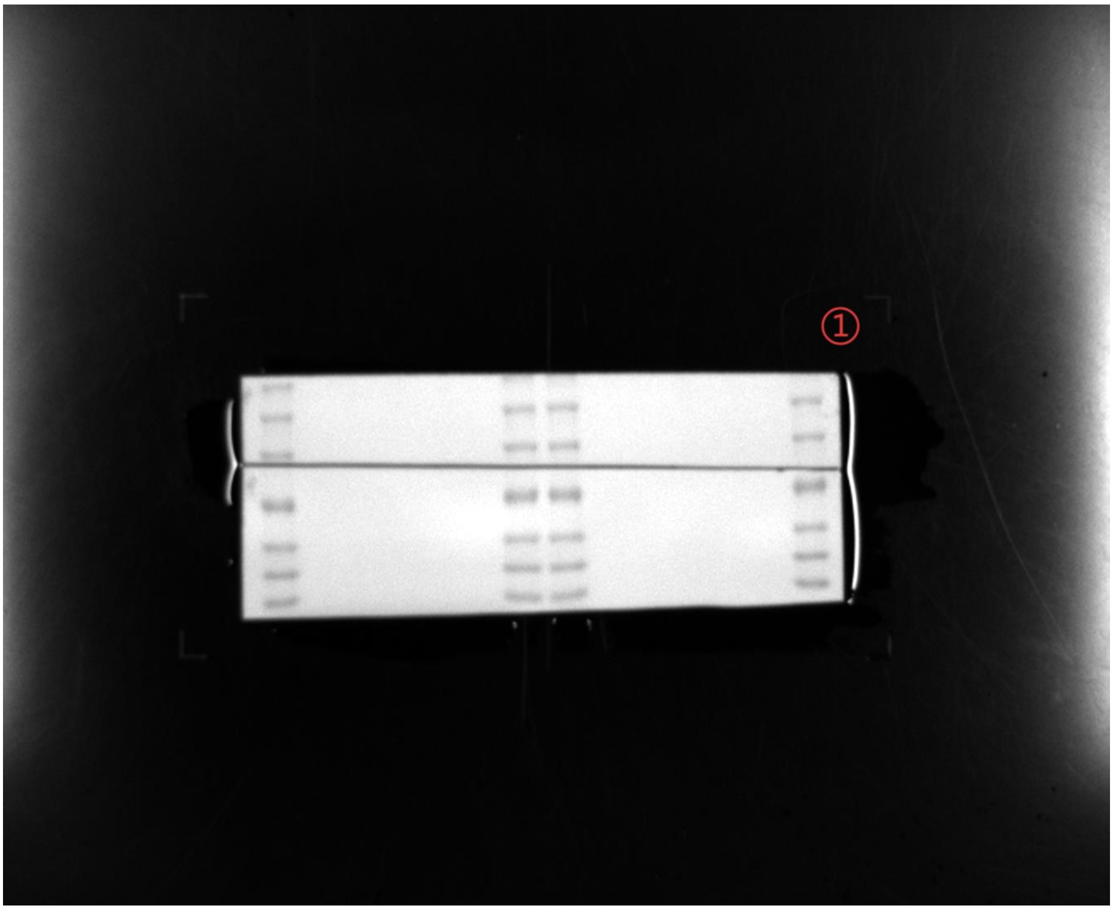

②

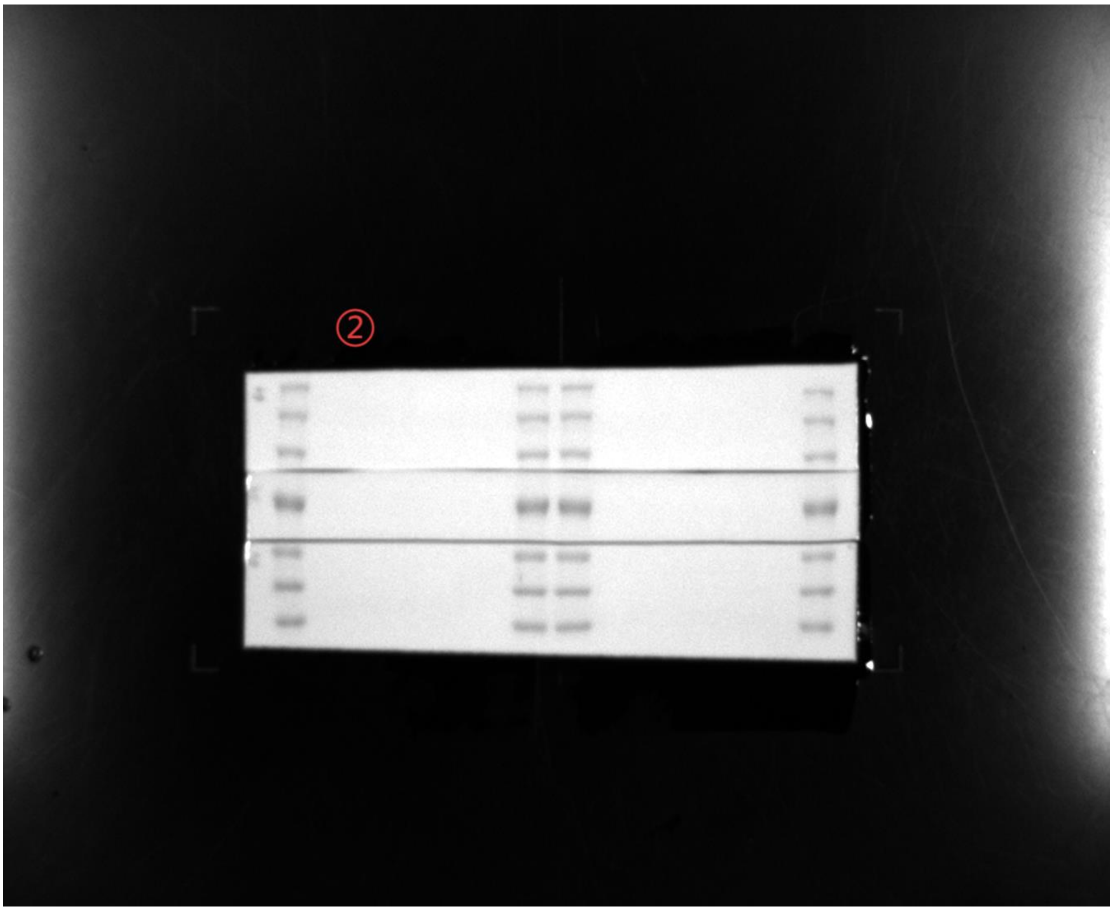

③

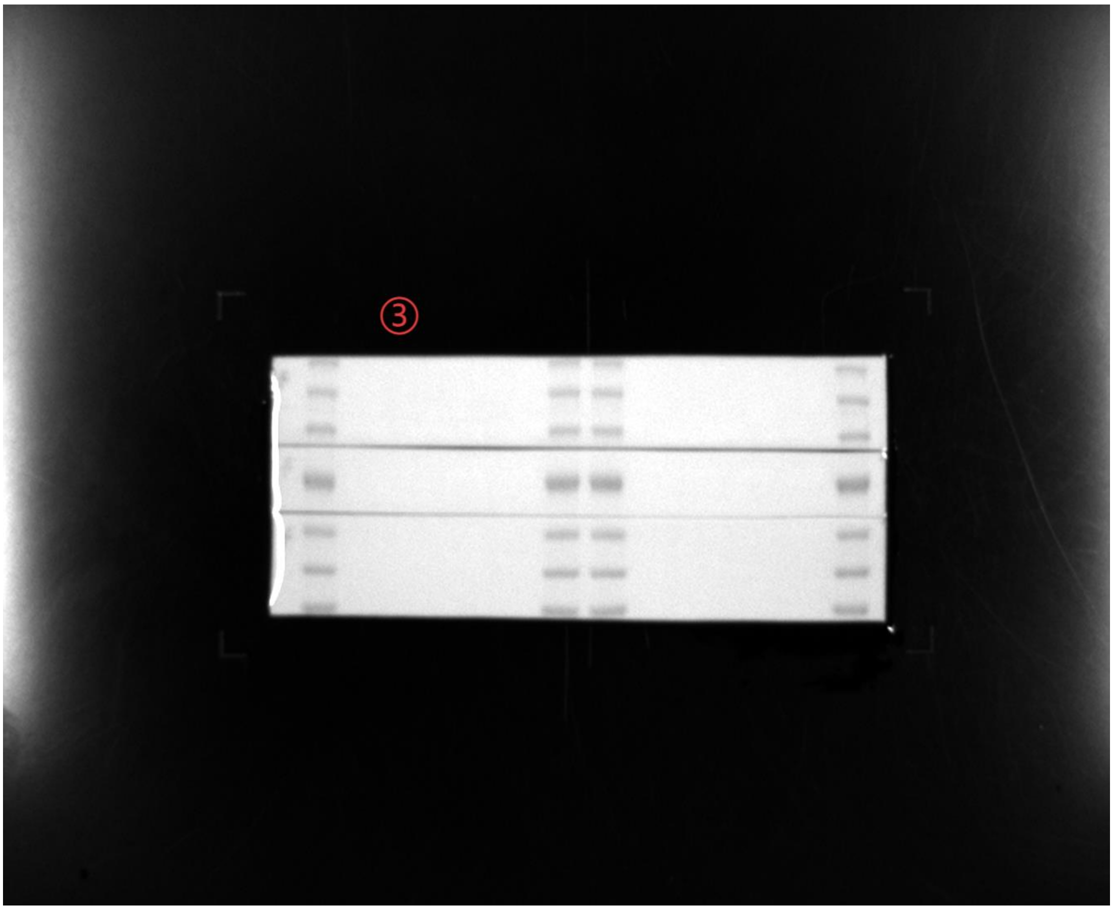

Y79(KIF5B)

The change in KIF5B protein expression was verified by Western blot using a 7.5% separating gel in the Y79 cells among the control group, overexpression-ARL5B group, RPL41 intervention group and overexpression-ARL5B with RPL41 intervention group.

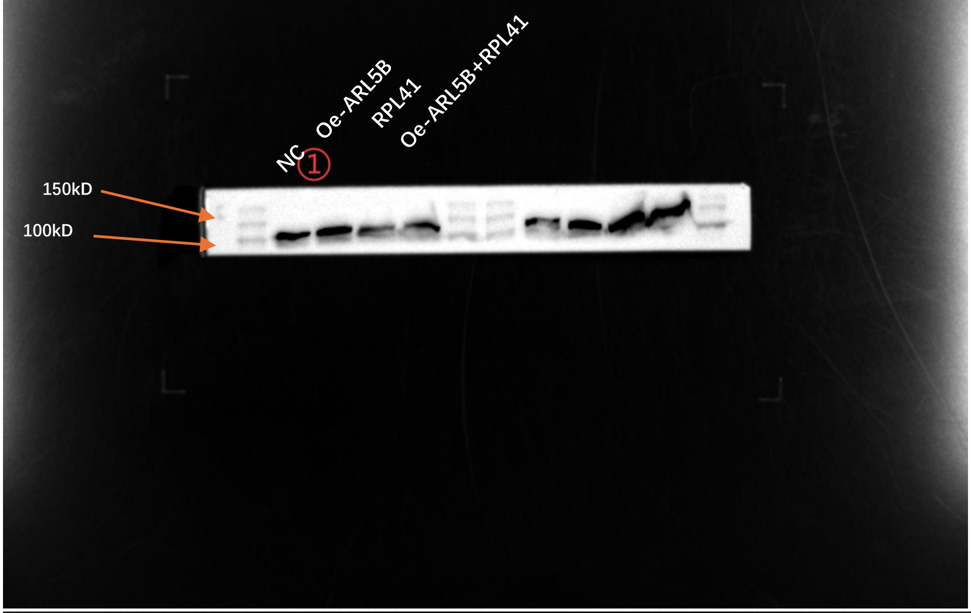

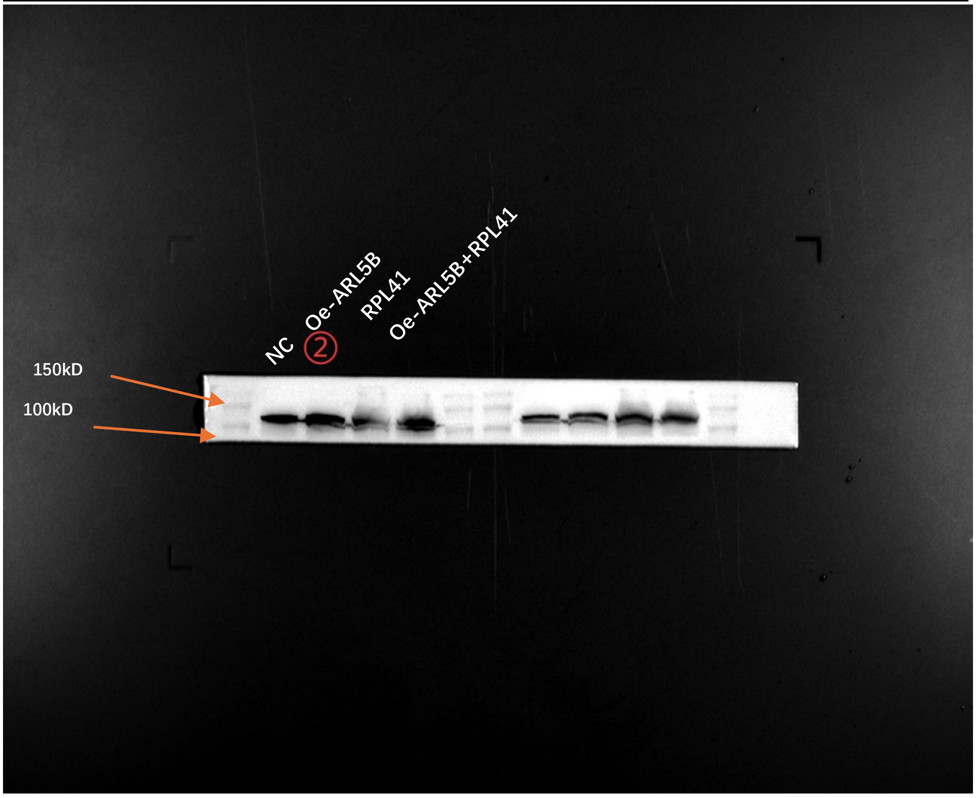

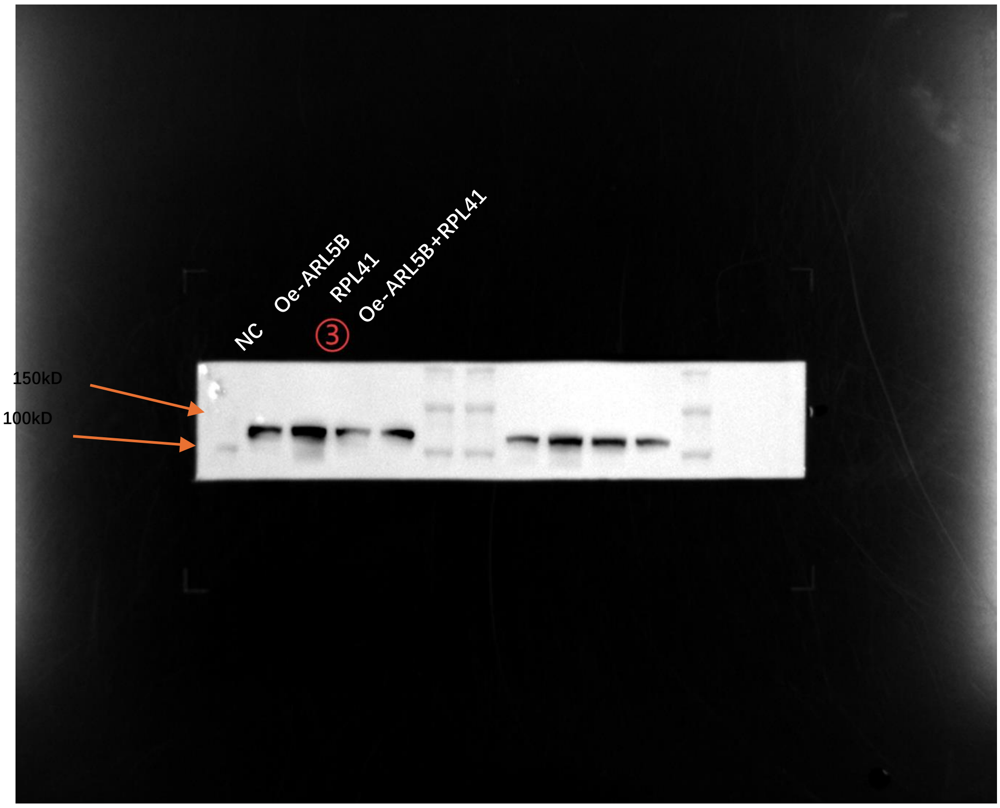

(β-Actin)

The change in β-Actin protein expression was verified by Western blot using a 7.5% separating gel in the Y79 cells among the control group, overexpression-ARL5B group, RPL41 intervention group and overexpression-ARL5B with RPL41 intervention group.

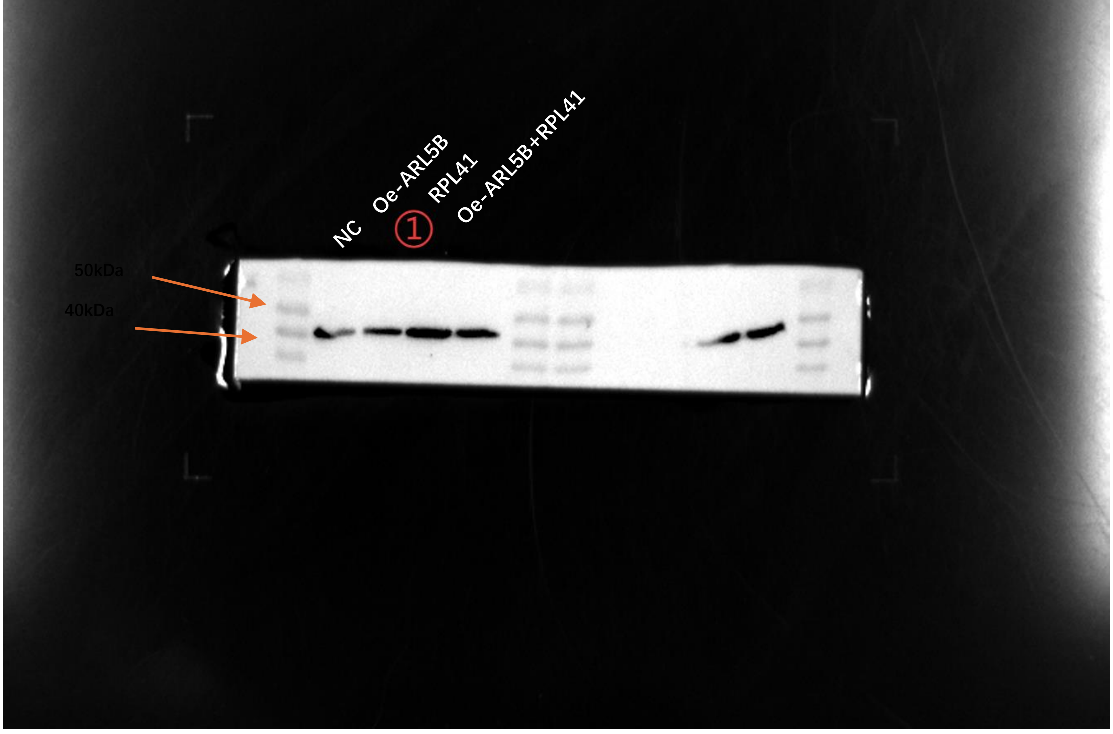

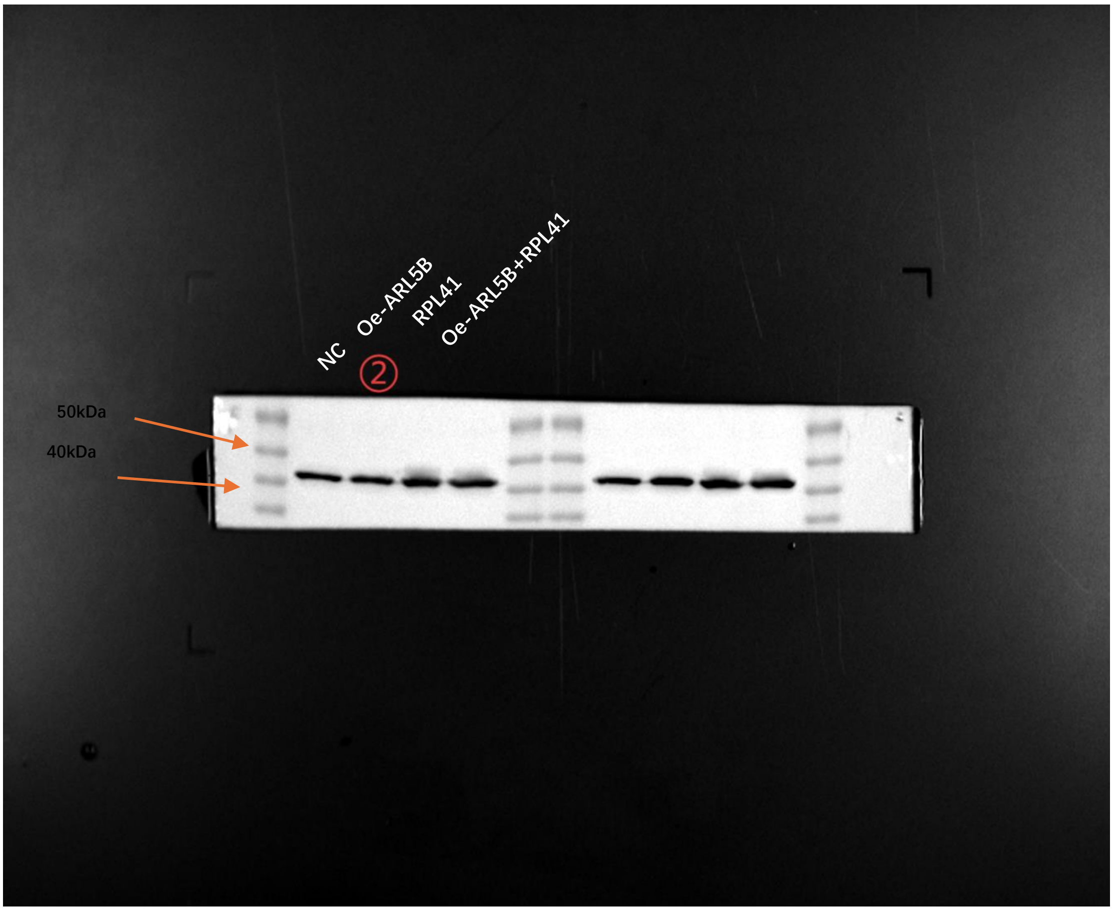
NC
Oe-ARL5B
RPL41
Oe-ARL5B+RPL41
②
50kDa
40kDa

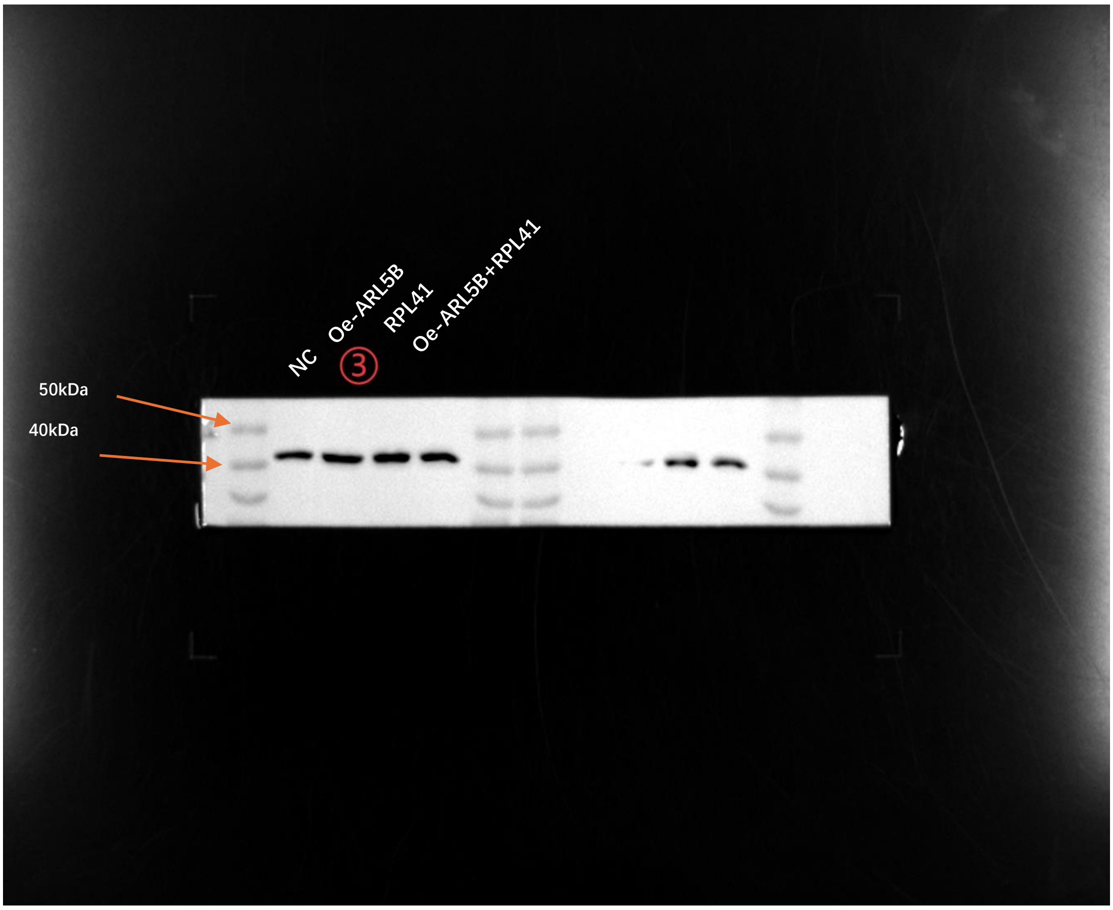
NC
Oe-ARL5B
RPL41
Oe-ARL5B+RPL41
③
50kDa
40kDa

Y79(KLC2)

The change in KLC2 protein expression was verified by Western blot using a 10% separating gel in the Y79 cells among the control group, overexpression-ARL5B group, RPL41 intervention group and overexpression-ARL5B with RPL41 intervention group.

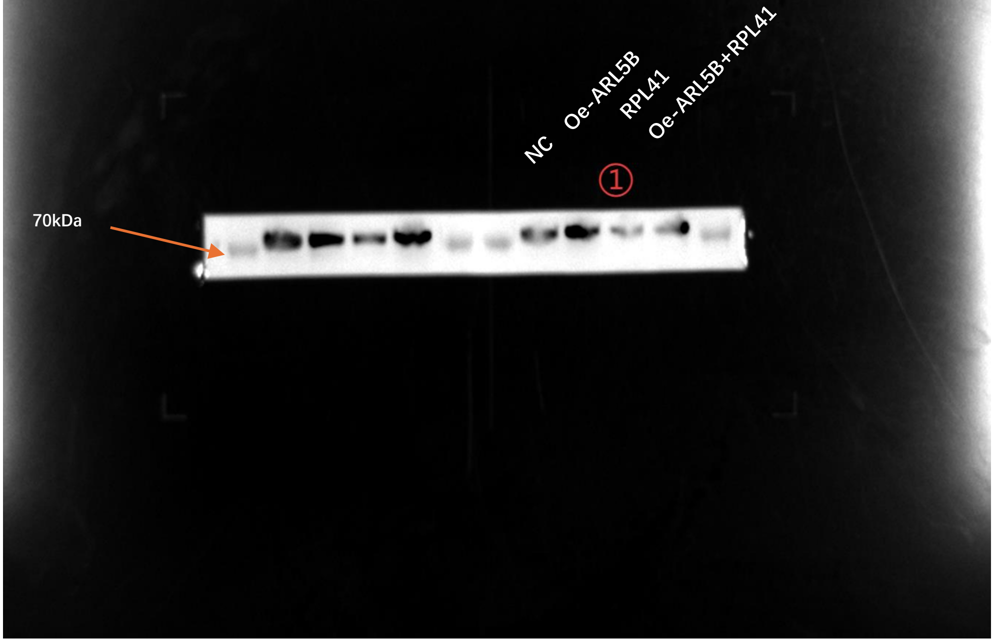

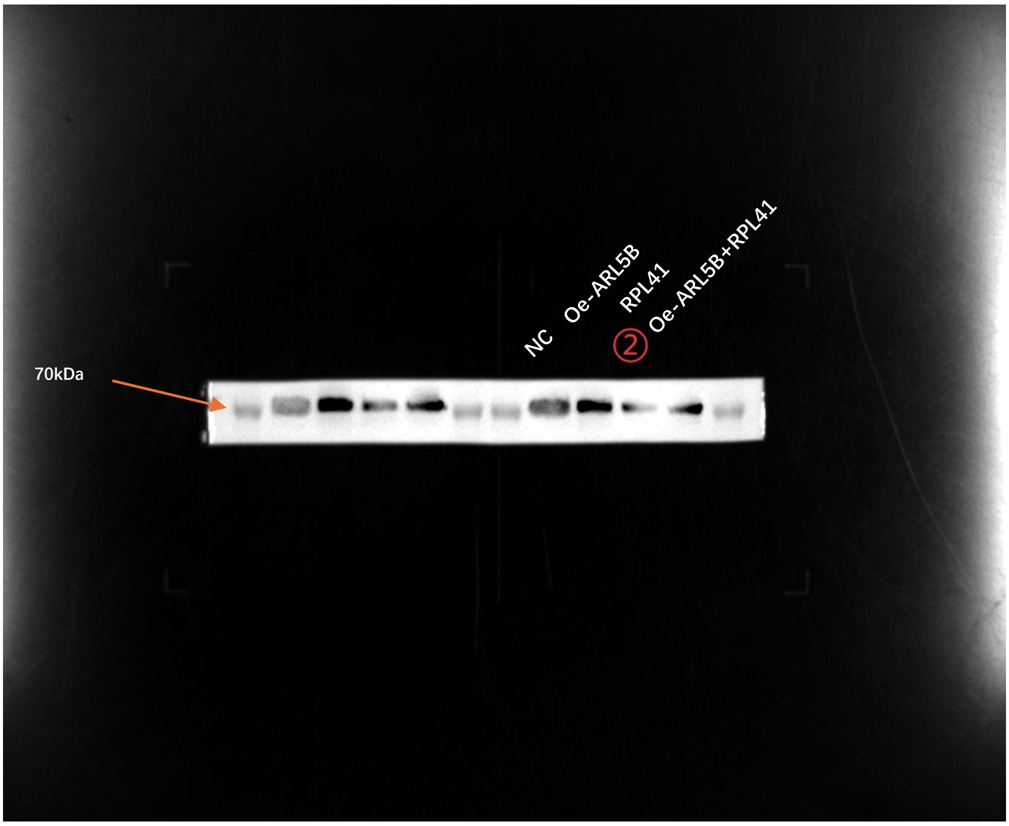

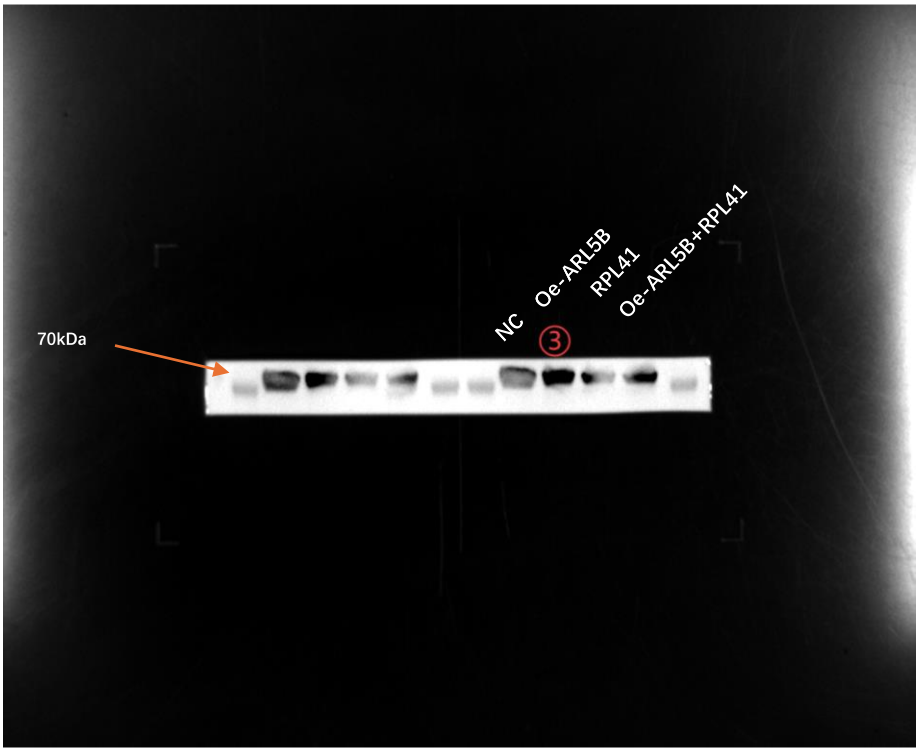
70kDa
NC
Oe-ARL5B
RPL41
Oe-ARL5B+RPL41
③

(β-Actin)

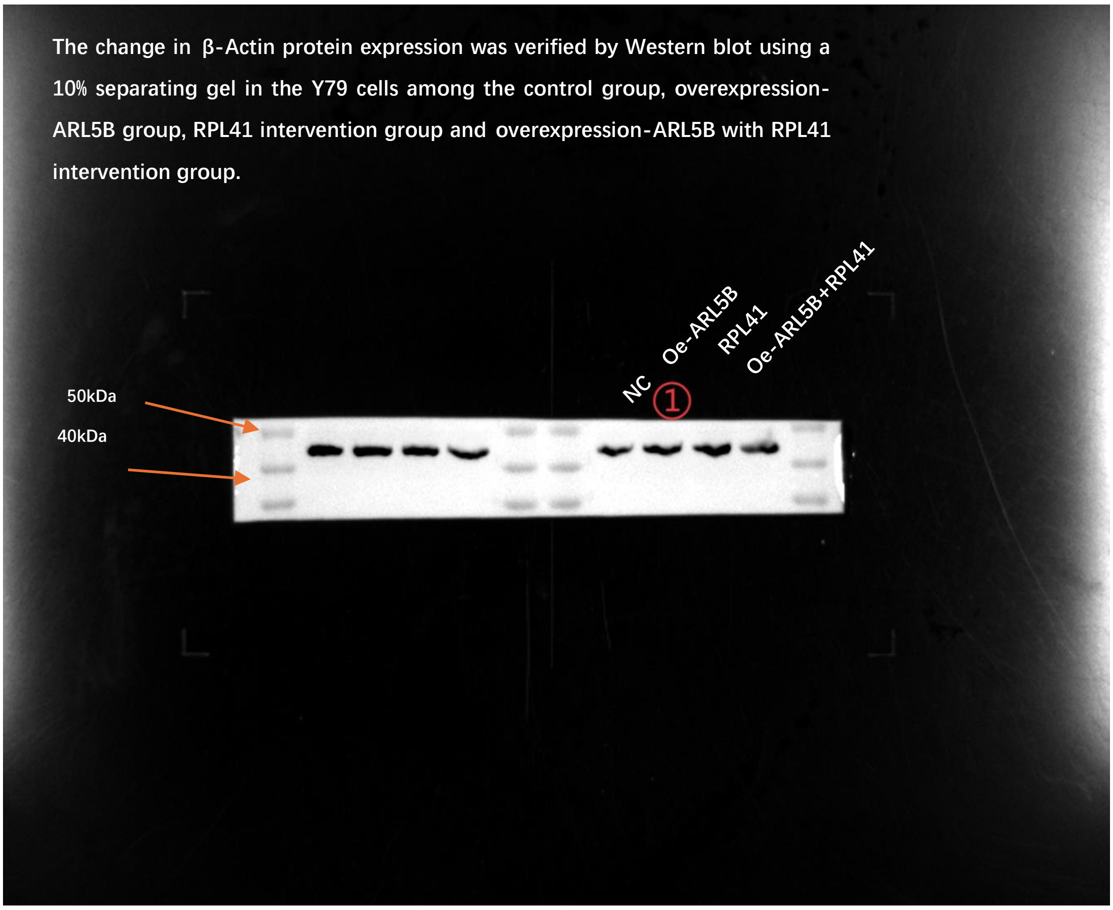
The change in β-Actin protein expression was verified by Western blot using a 10% separating gel in the Y79 cells among the control group, overexpression-ARL5B group, RPL41 intervention group and overexpression-ARL5B with RPL41 intervention group.
50kDa
40kDa
NC
Oe-ARL5B
RPL41
Oe-ARL5B+RPL41
①

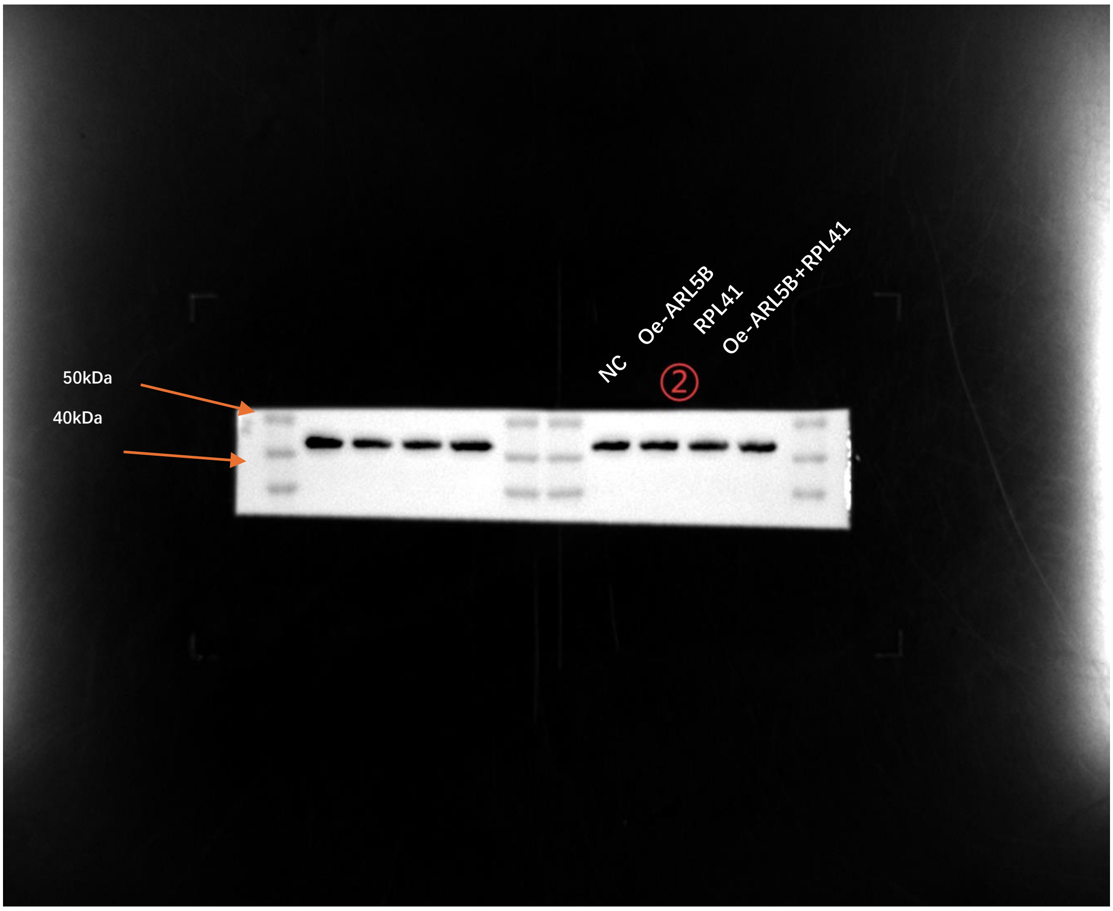

50kDa
40kDa
NC
Oe-ARL5B
RPL41
Oe-ARL5B+RPL41
②

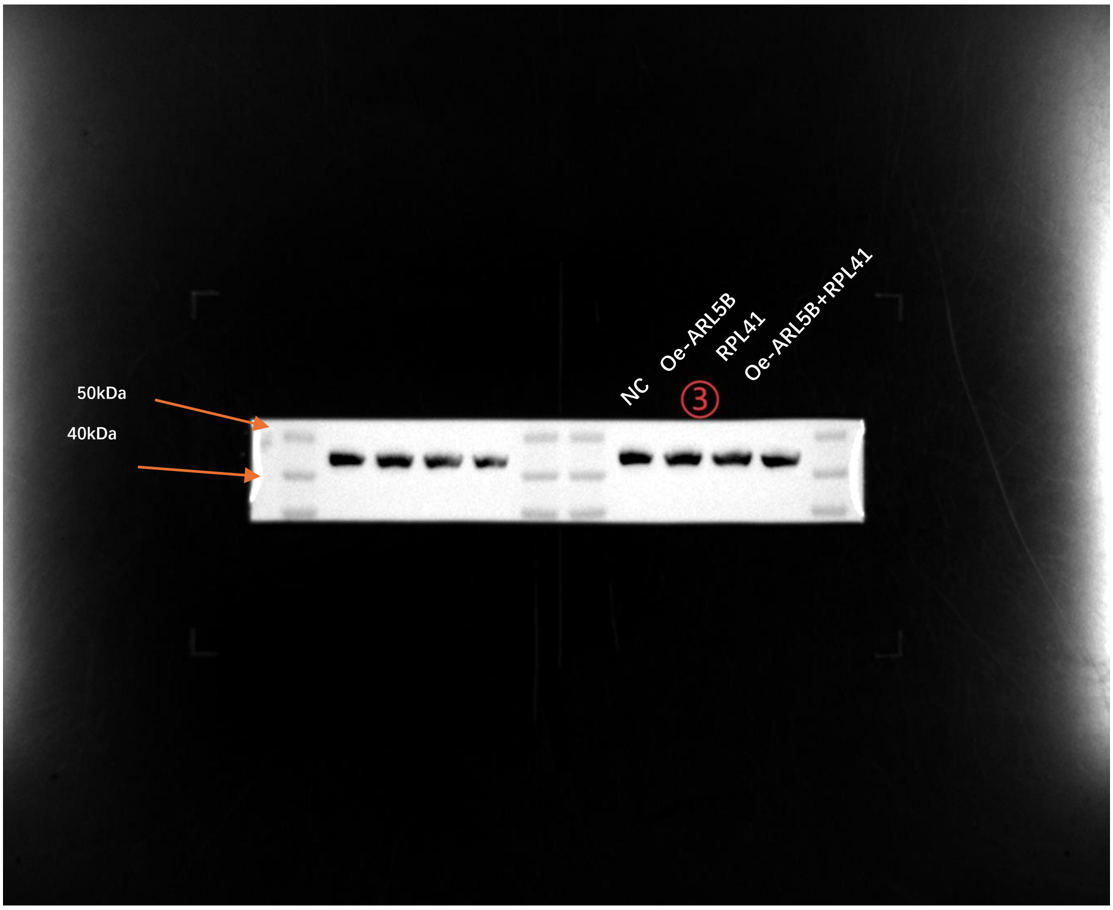

50kDa
40kDa
NC
Oe-ARL5B
RPL41
Oe-ARL5B+RPL41
③

(MERGE)

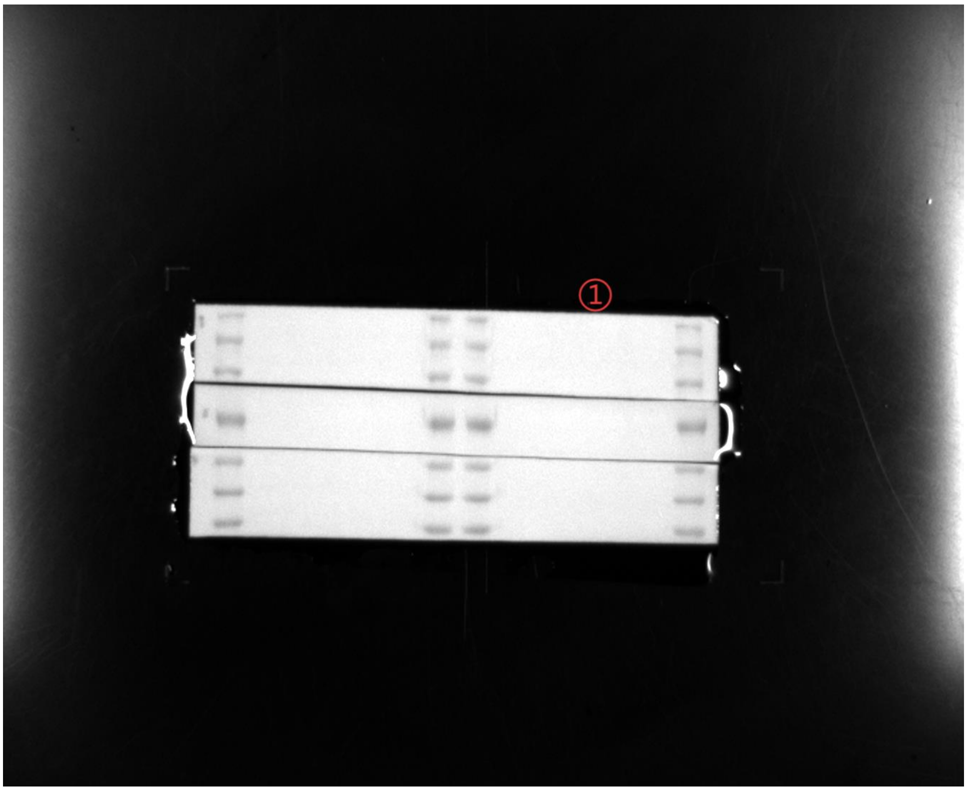

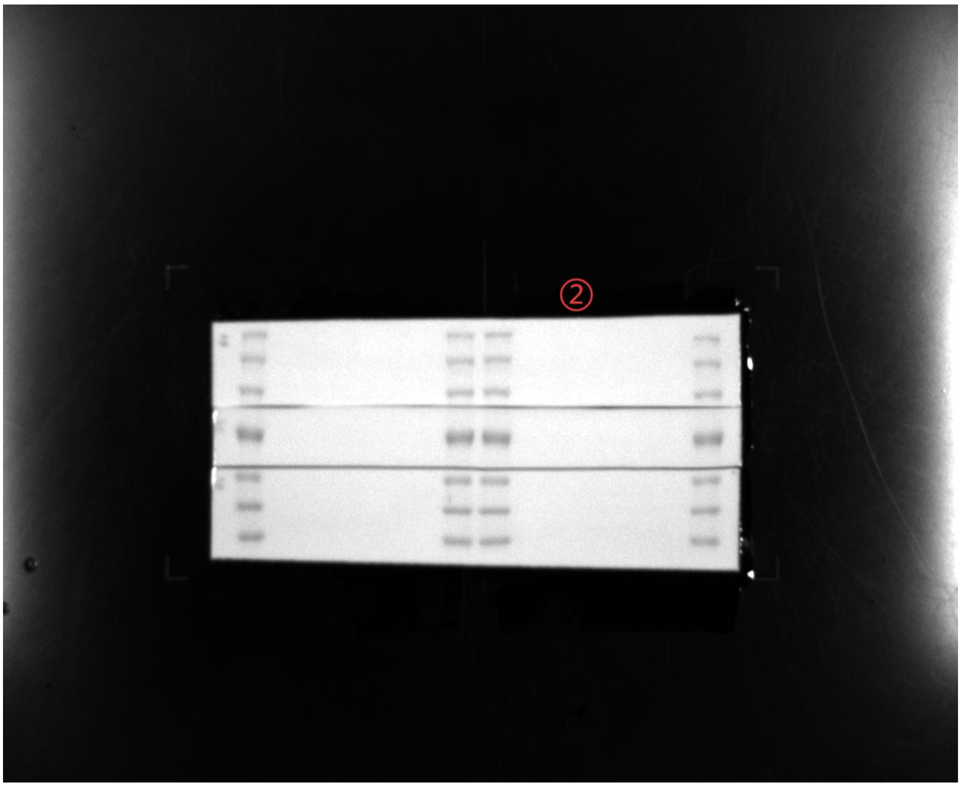

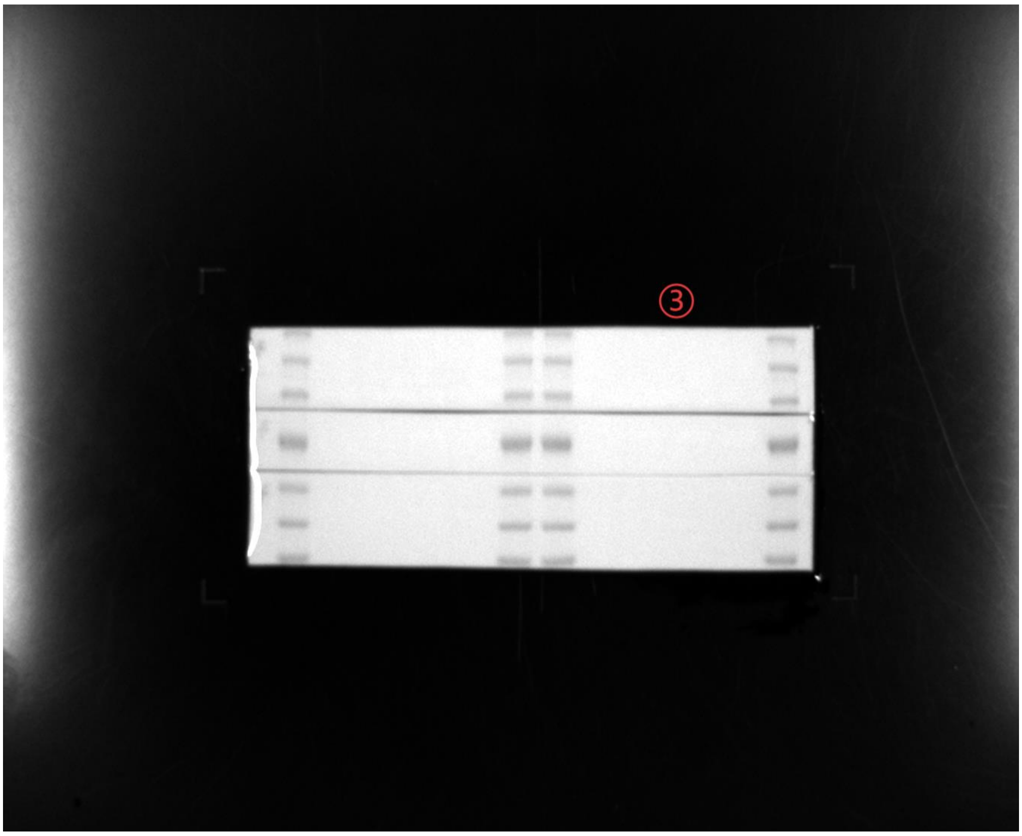

Weri-RB1(ARL5B)

The change in ARL5B protein expression was verified by Western blot using a 12.5% separating gel in the Weri-RB1 cells among the control group, overexpression-ARL5B group, RPL41 intervention group and overexpression-ARL5B with RPL41 intervention group.

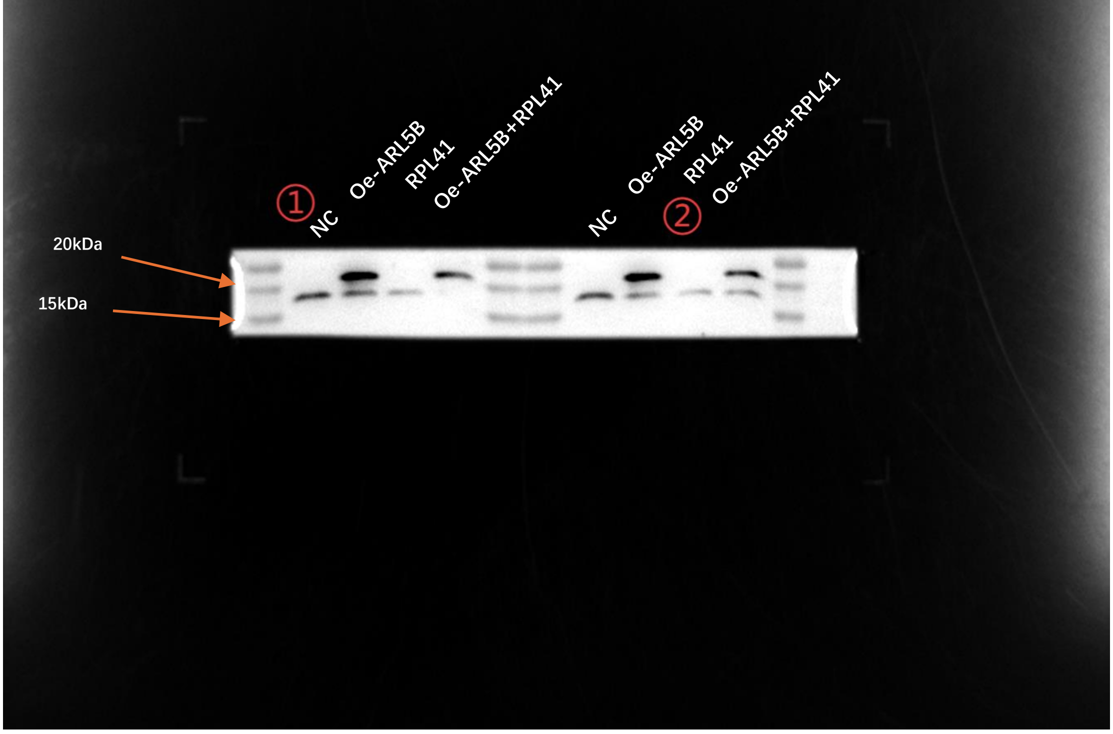

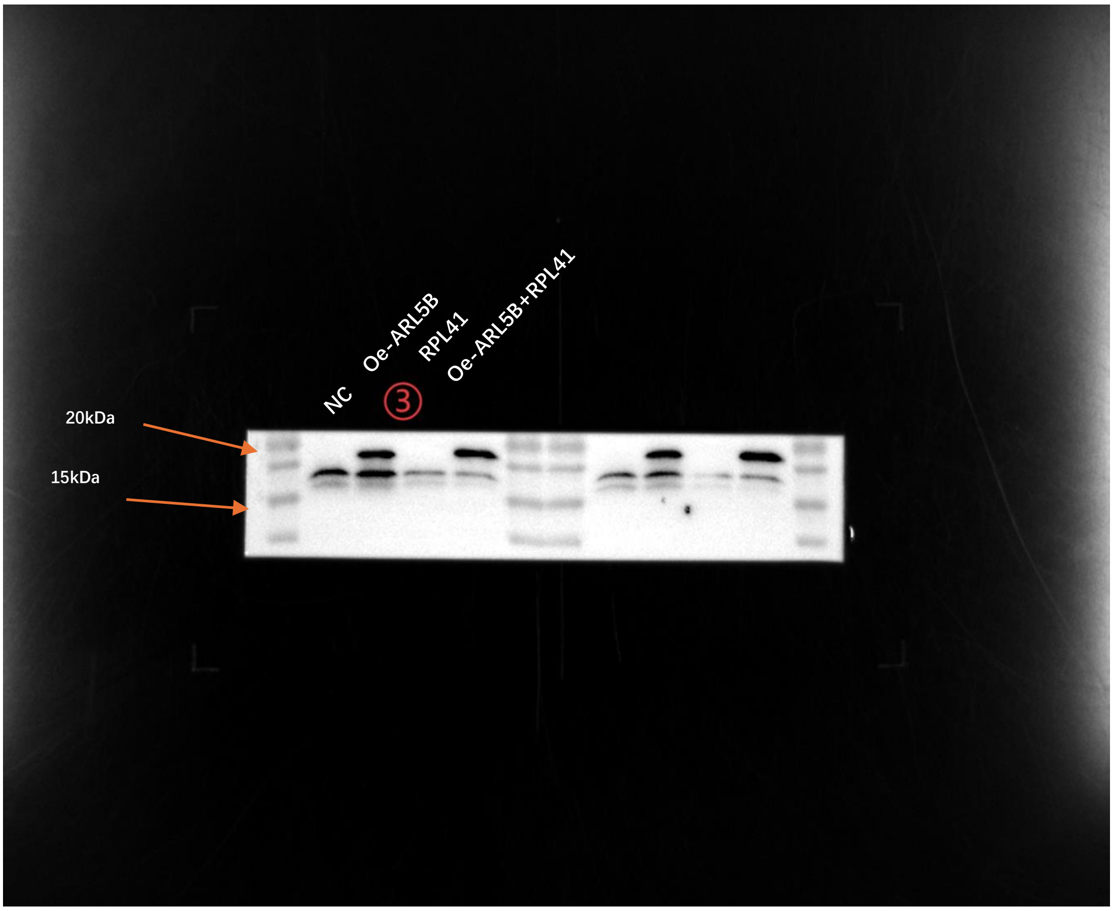

(β-Actin)

The change in ARL5B protein expression was verified by Western blot using a 12.5% separating gel in the Weri-RB1 cells among the control group, overexpression-ARL5B group, RPL41 intervention group and overexpression-ARL5B with RPL41 intervention group.

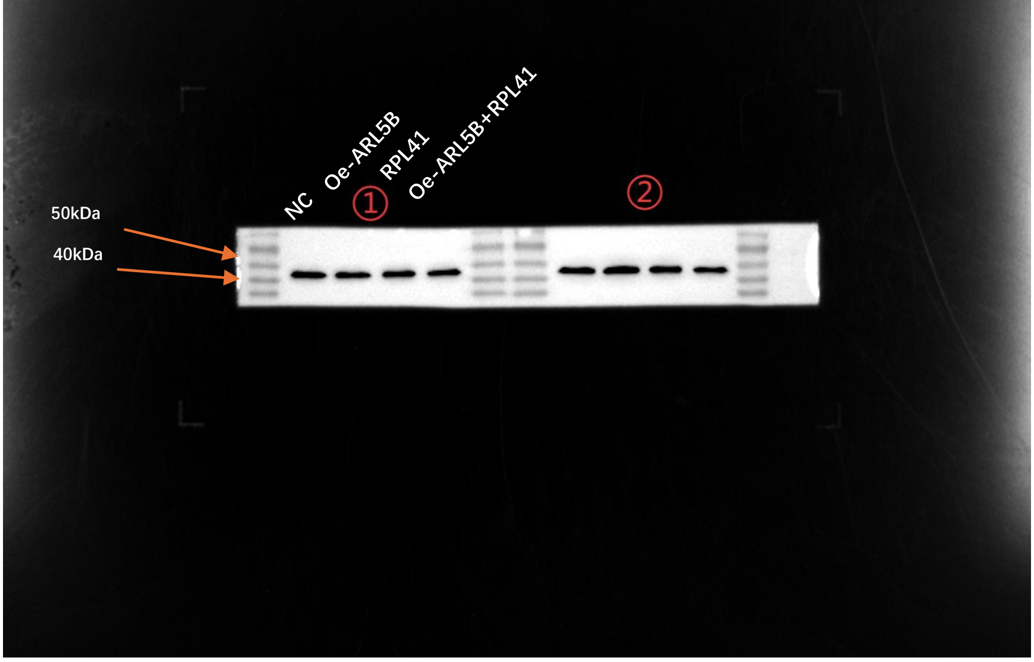

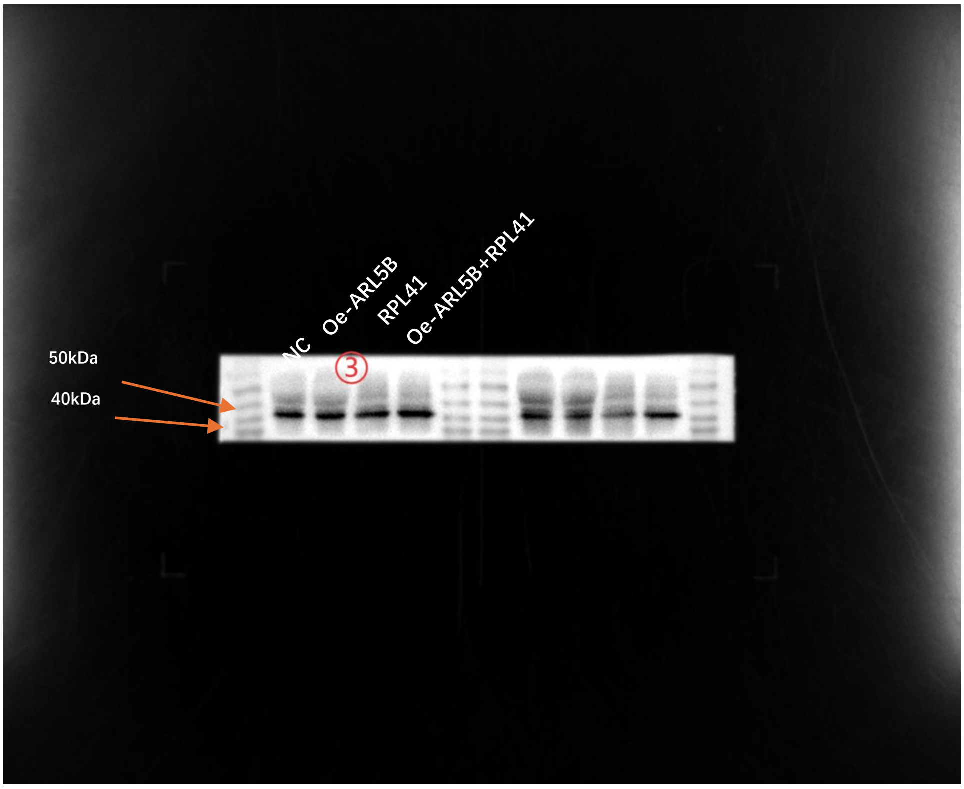

(MERGE-3)

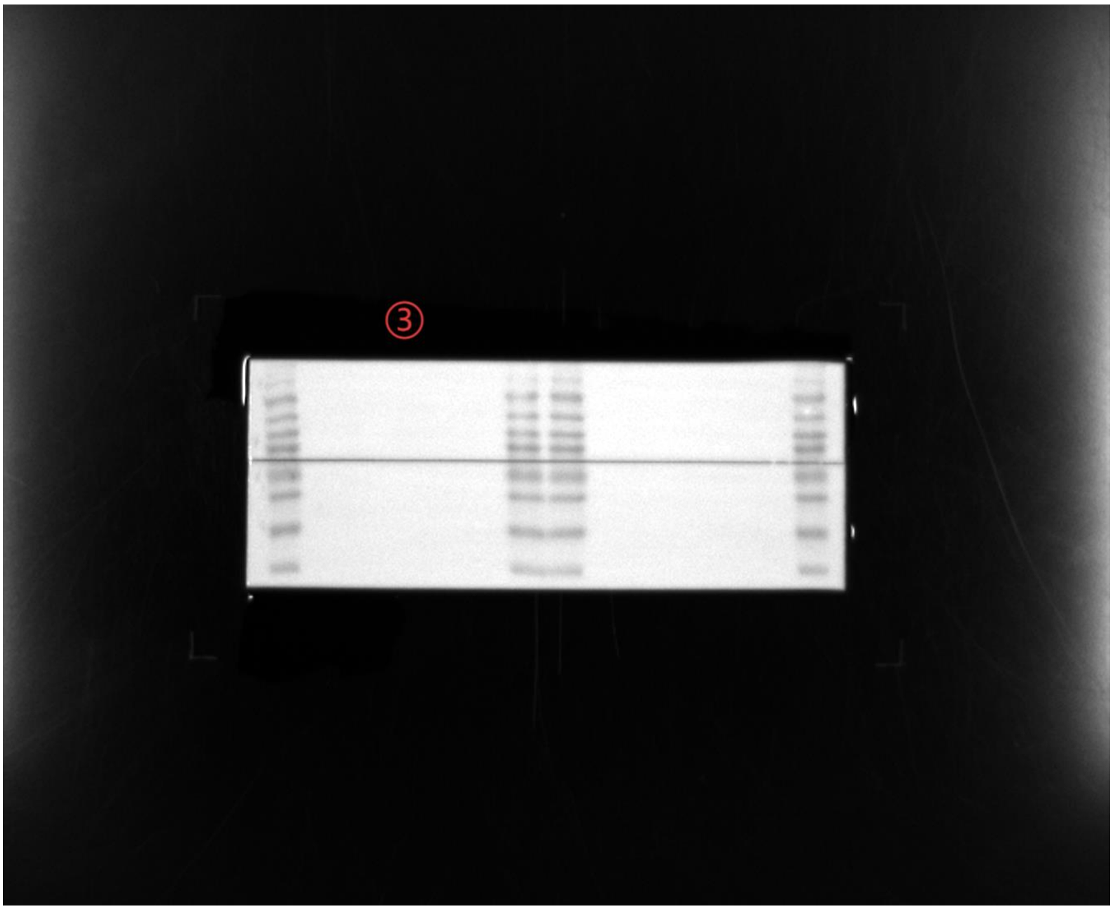

Weri-RB1(SKIP)

The change in SKIP protein expression was verified by Western blot using a 7.5% separating gel in the Weri-RB1 cells among the control group, overexpression-ARL5B group, RPL41 intervention group and overexpression-ARL5B with RPL41 intervention group.

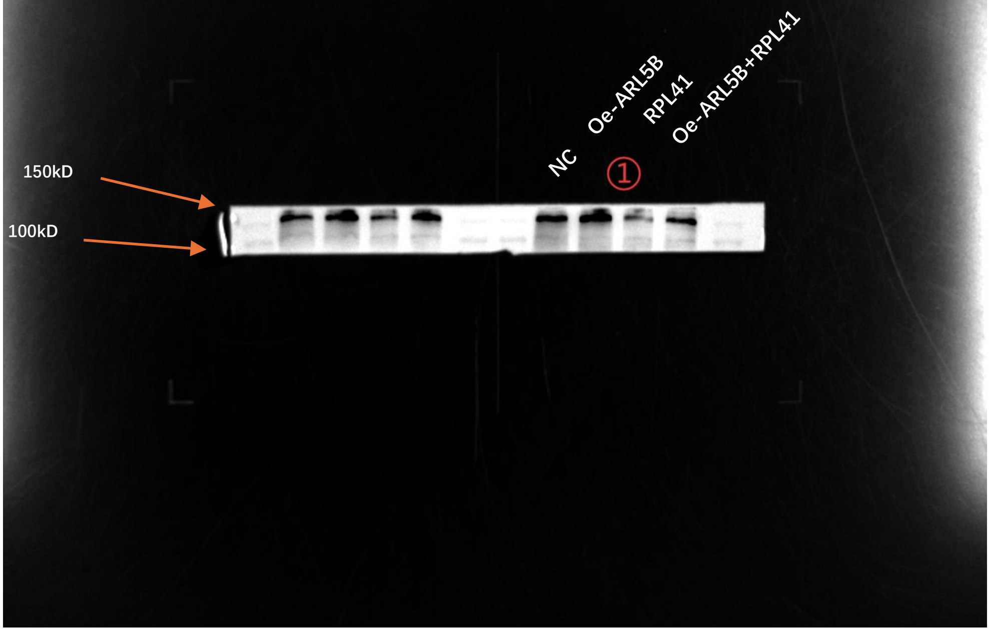

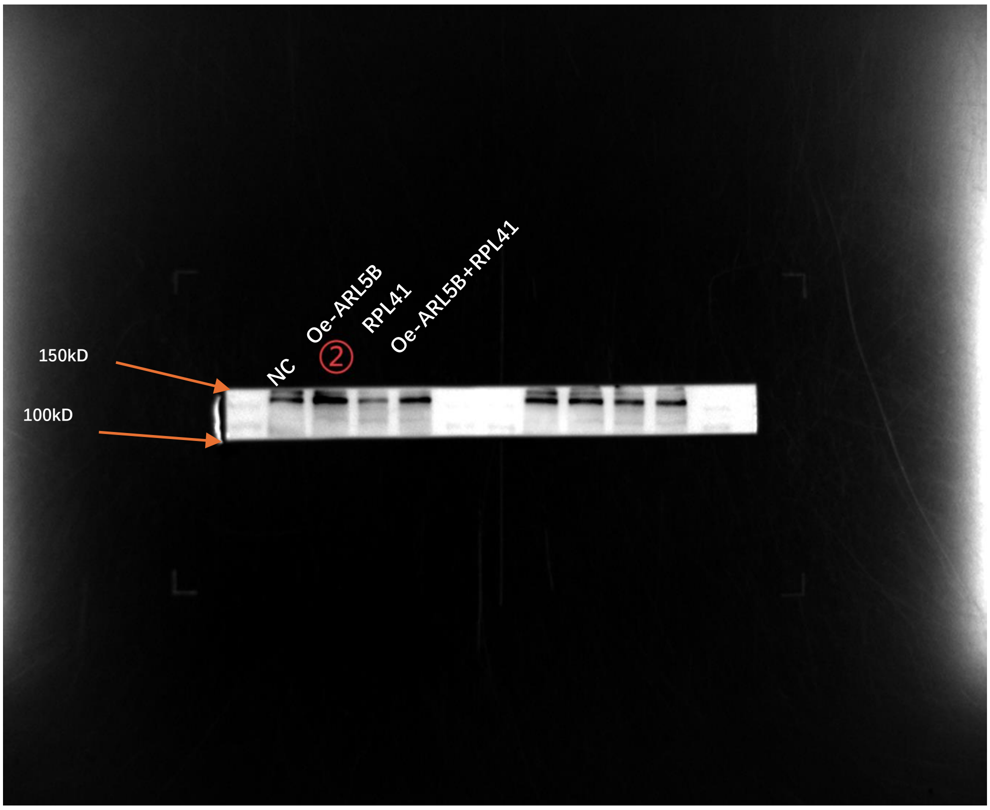

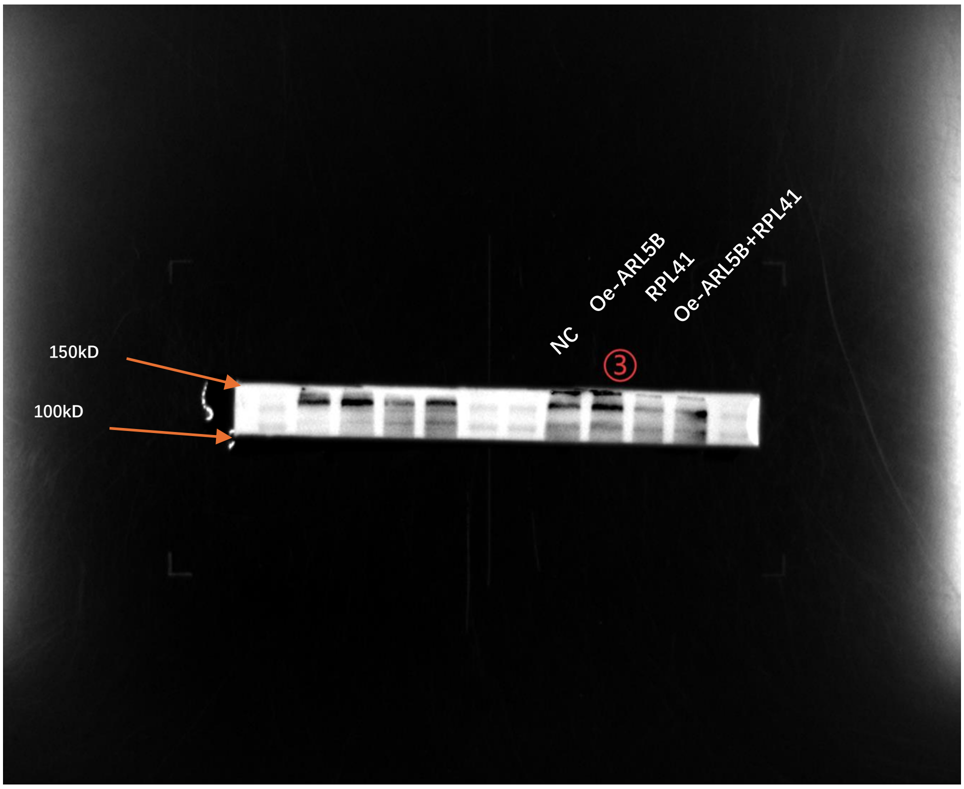

(β-Actin)

The change in β-Actin protein expression was verified by Western blot using a 7.5% separating gel in the Weri-RB1 cells among the control group, overexpression-ARL5B group, RPL41 intervention group and overexpression-ARL5B with RPL41 intervention group.

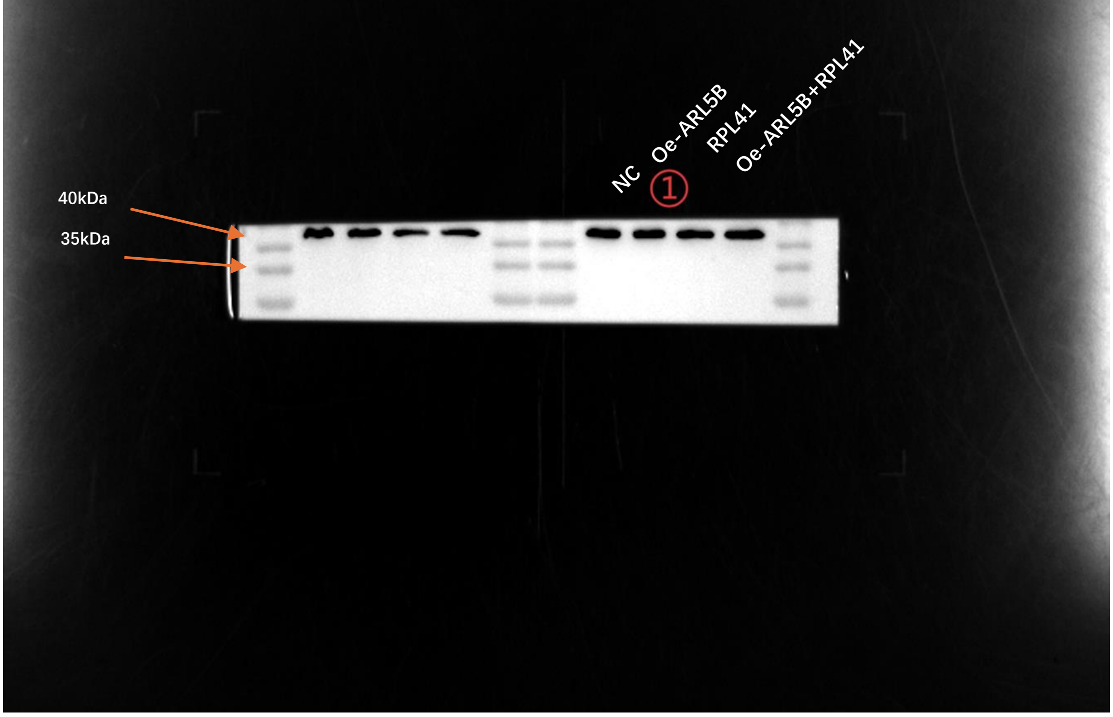

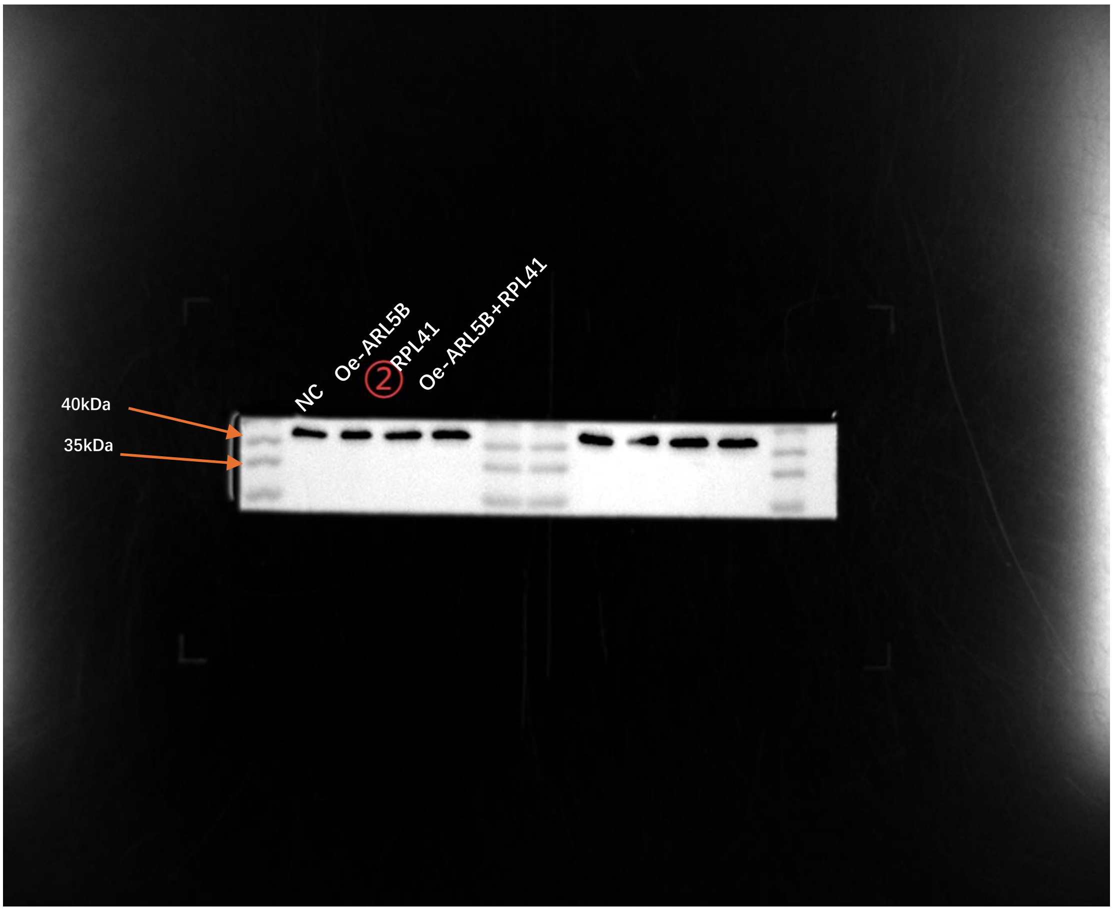
40kDa
35kDa
NC
Oe-ARL5B
RPL41
Oe-ARL5B+RPL41
②

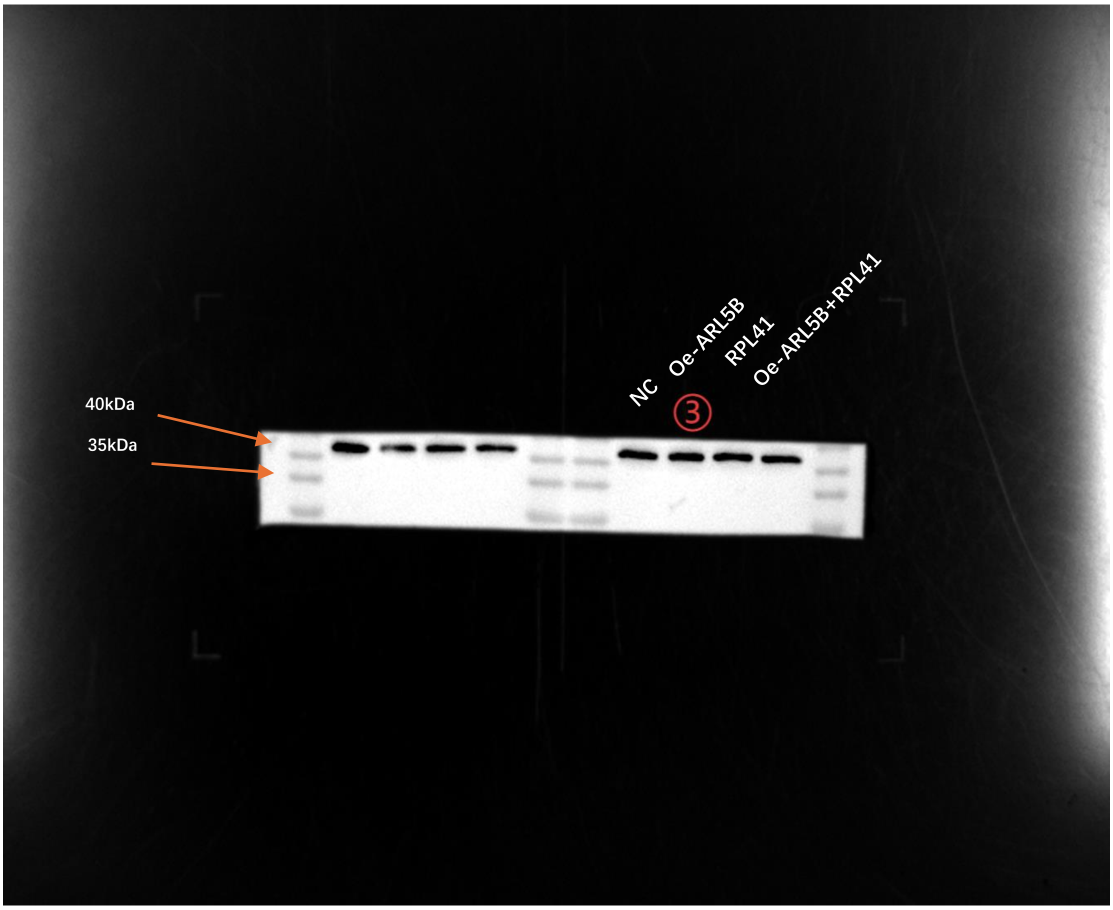
40kDa
35kDa
NC
Oe-ARL5B
RPL41
Oe-ARL5B+RPL41
③

(MERGE)

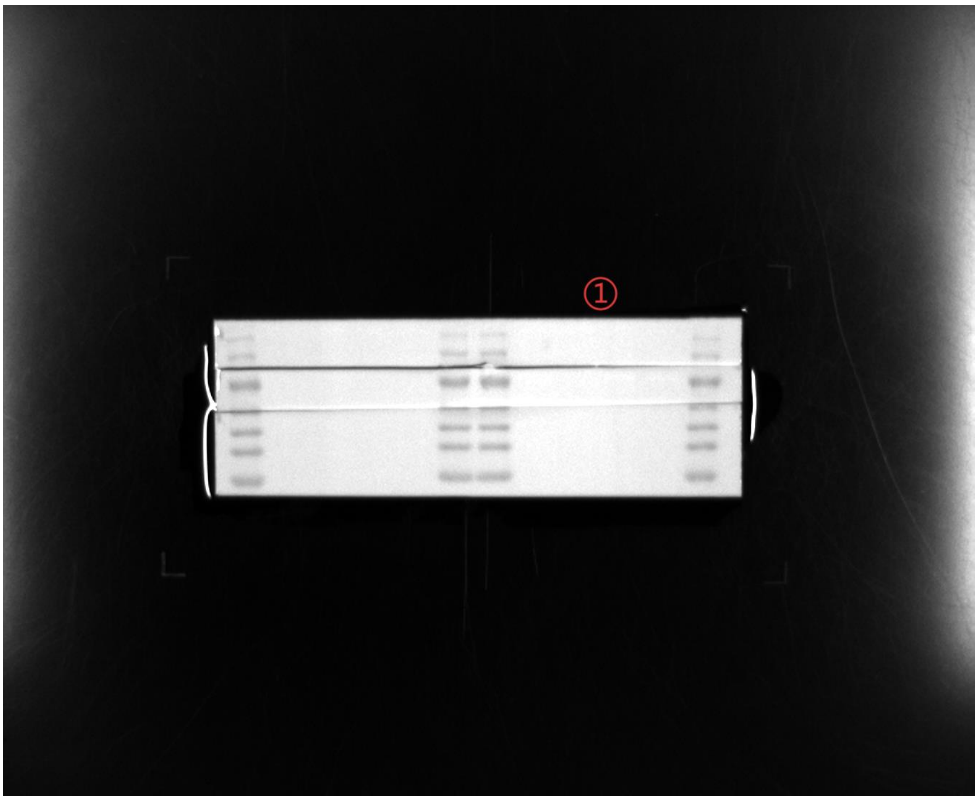

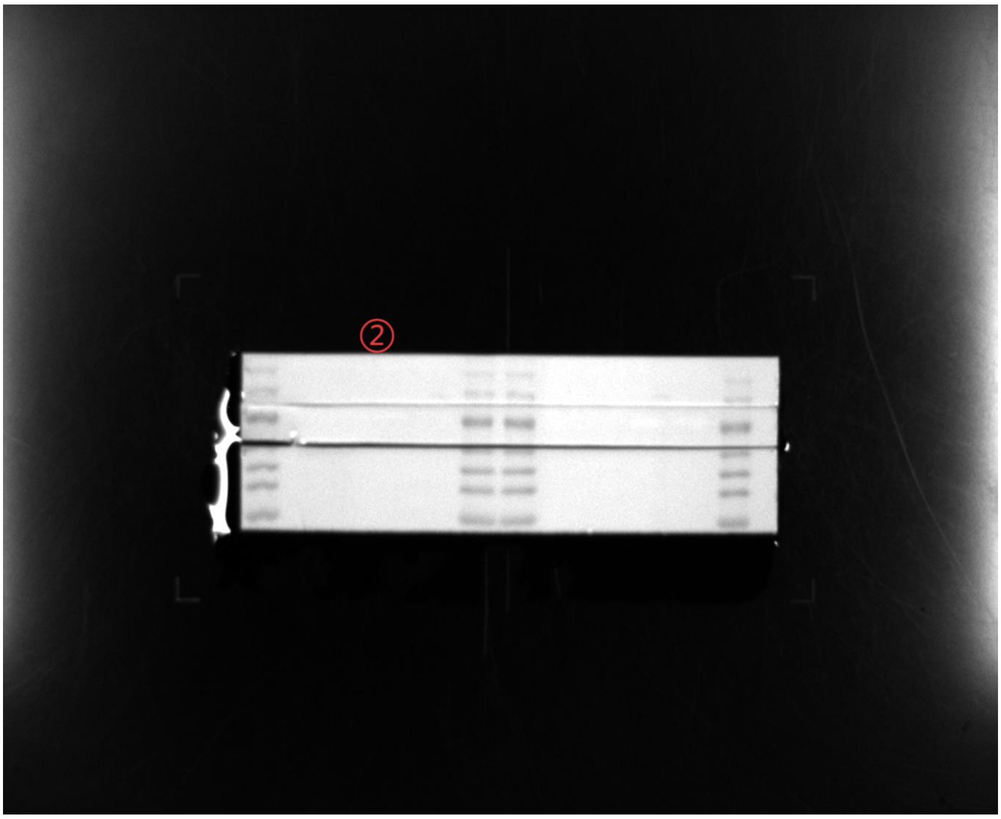

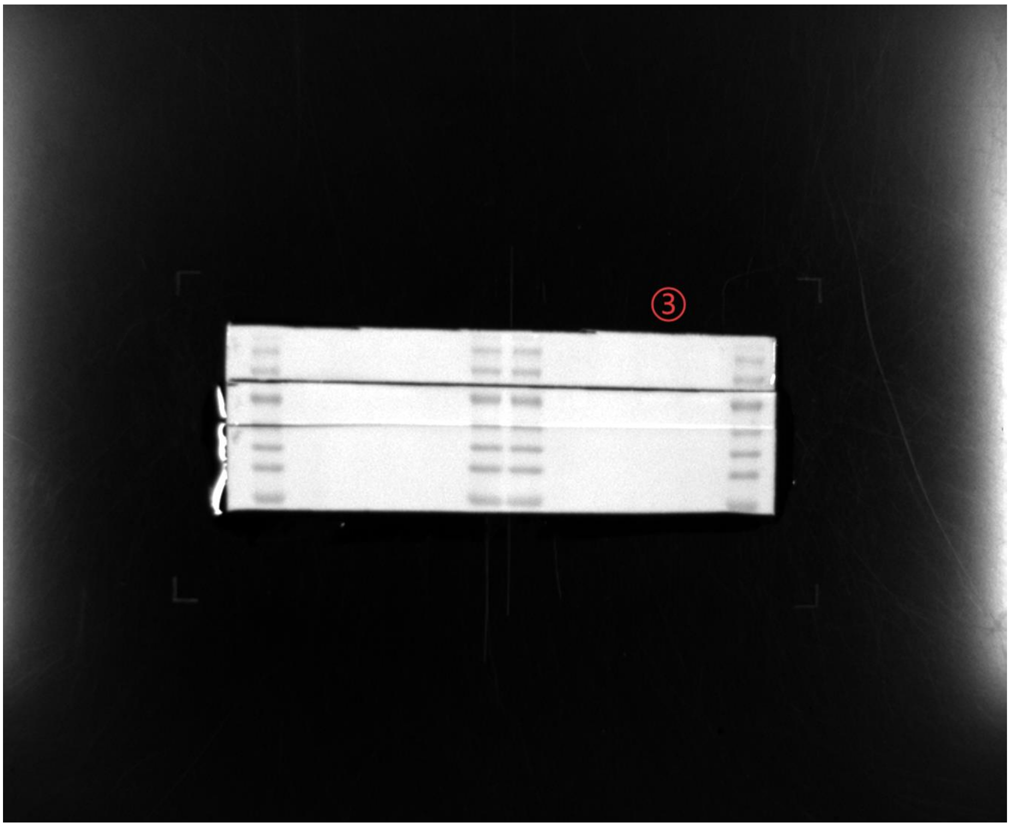

Weri-RB1(KIF5B)

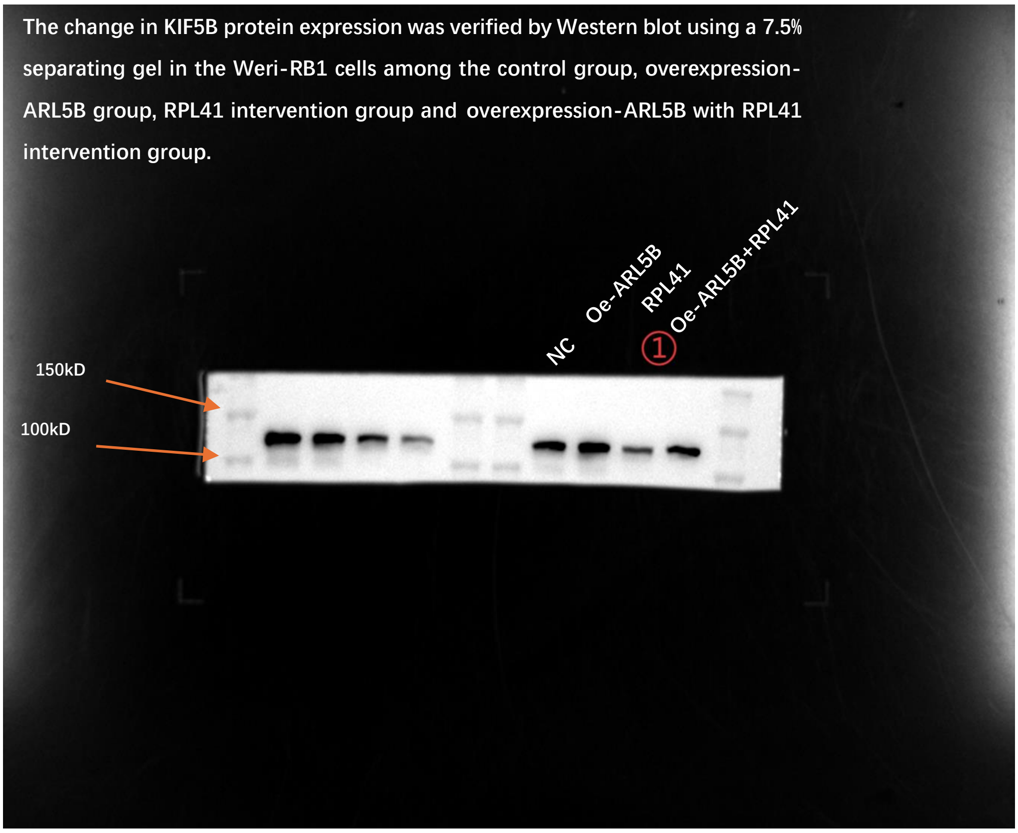

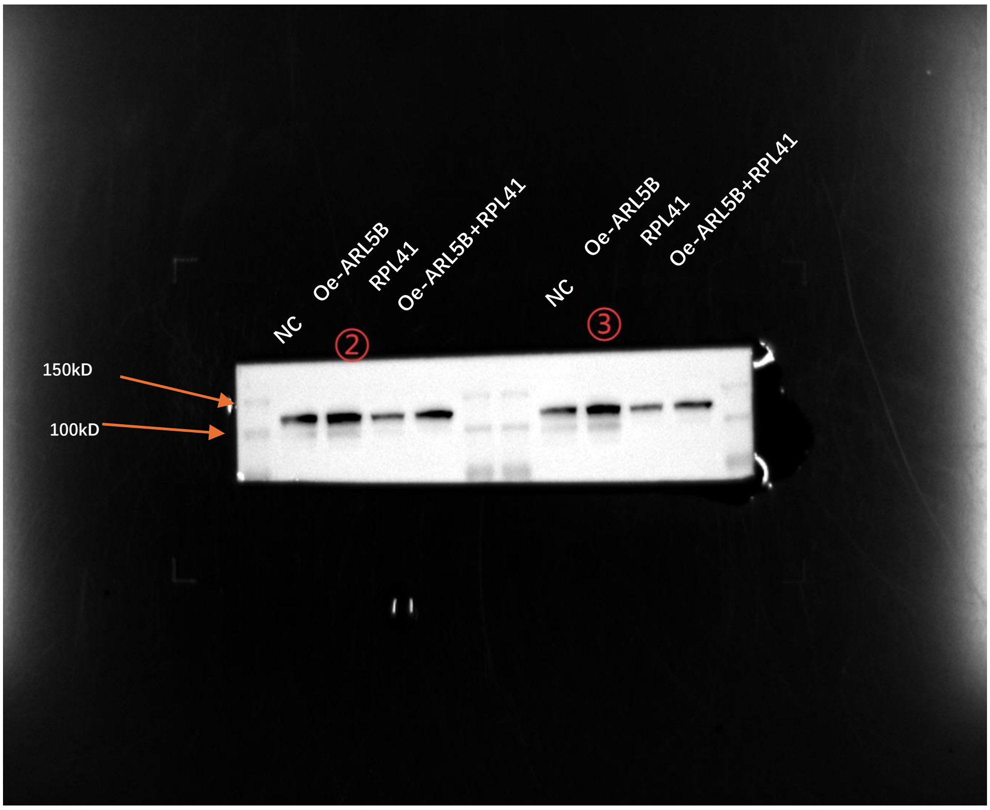

(β-Actin)

The change in β-Actin protein expression was verified by Western blot using a 7.5% separating gel in the Weri-RB1 cells among the control group, overexpression-ARL5B group, RPL41 intervention group and overexpression-ARL5B with RPL41 intervention group.

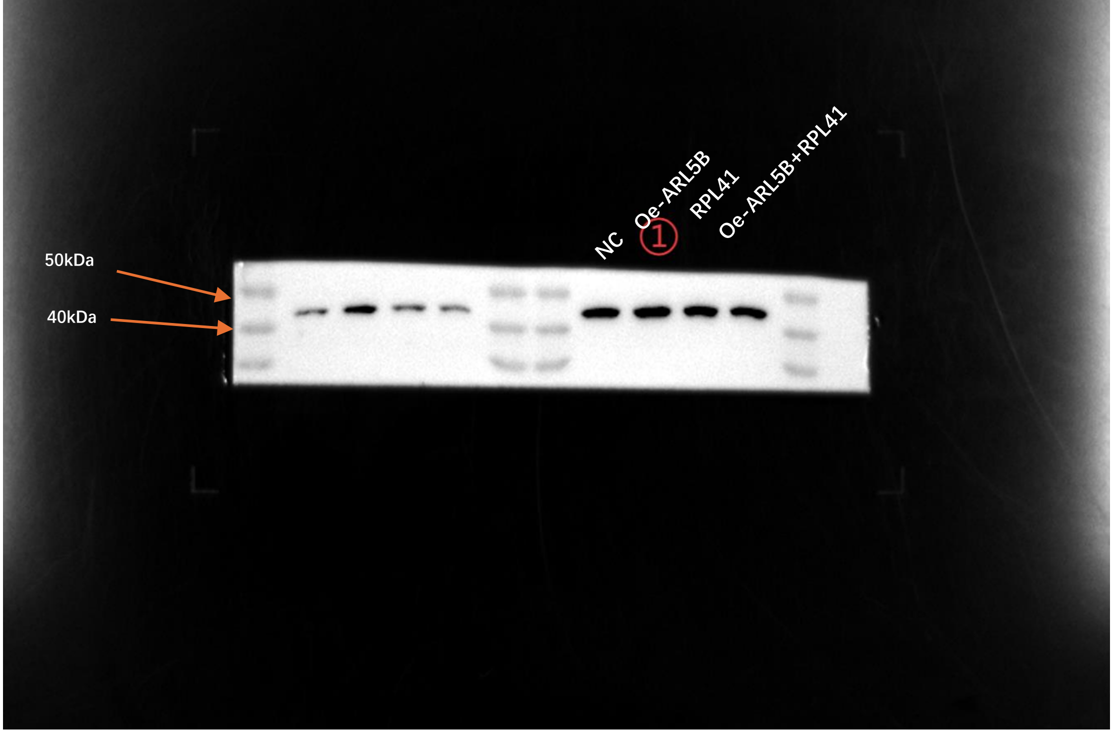

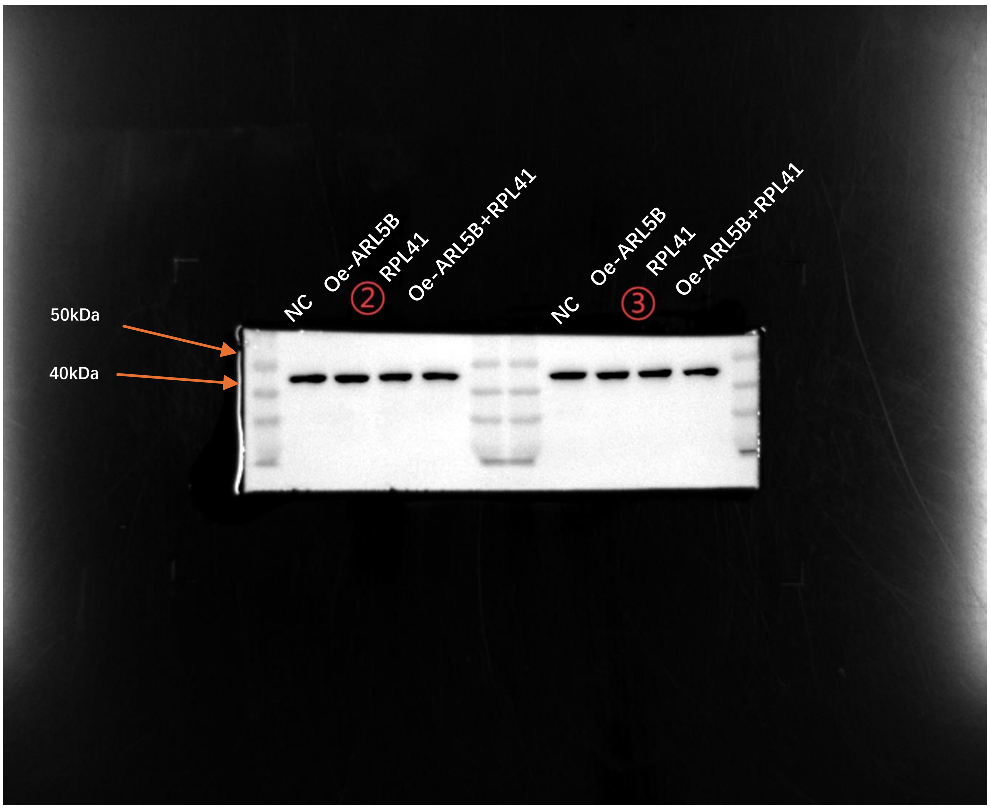
NC
Oe-ARL5B
RPL41
Oe-ARL5B+RPL41
②
NC
Oe-ARL5B
RPL41
Oe-ARL5B+RPL41
③
50kDa
40kDa

(MERGE-2.3)

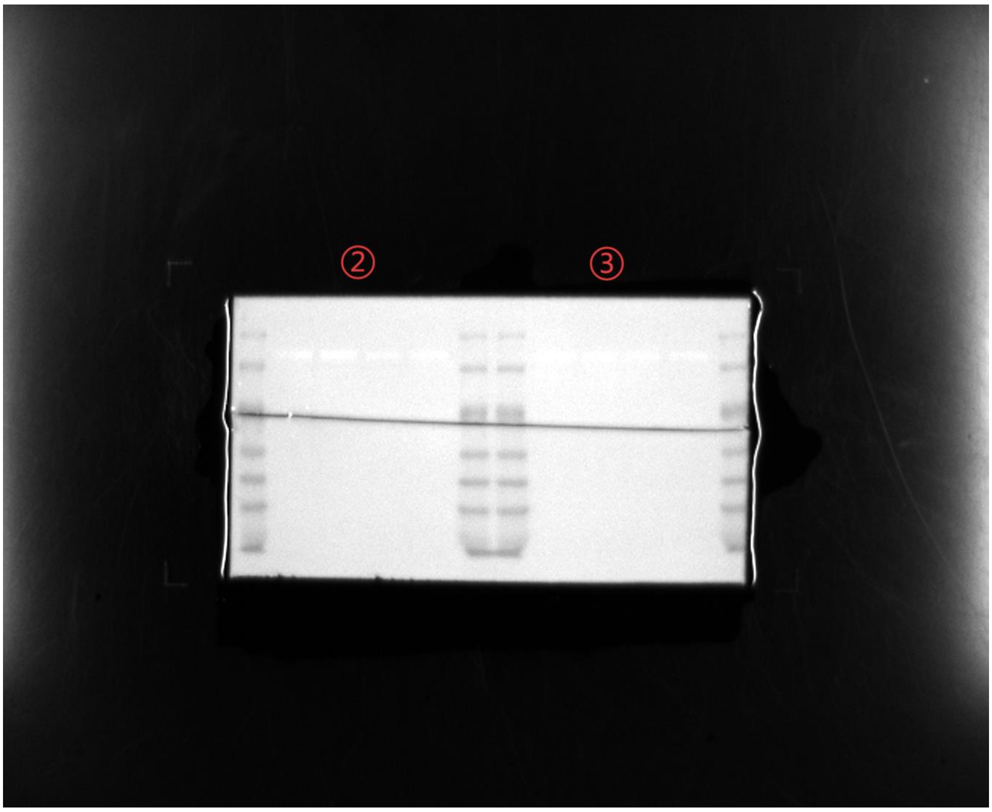
②
③

Weri-RB1(KLC2)

The change in KLC2 protein expression was verified by Western blot using a 10% separating gel in the Weri-RB1 cells among the control group, overexpression-ARL5B group, RPL41 intervention group and overexpression-ARL5B with RPL41 intervention group.

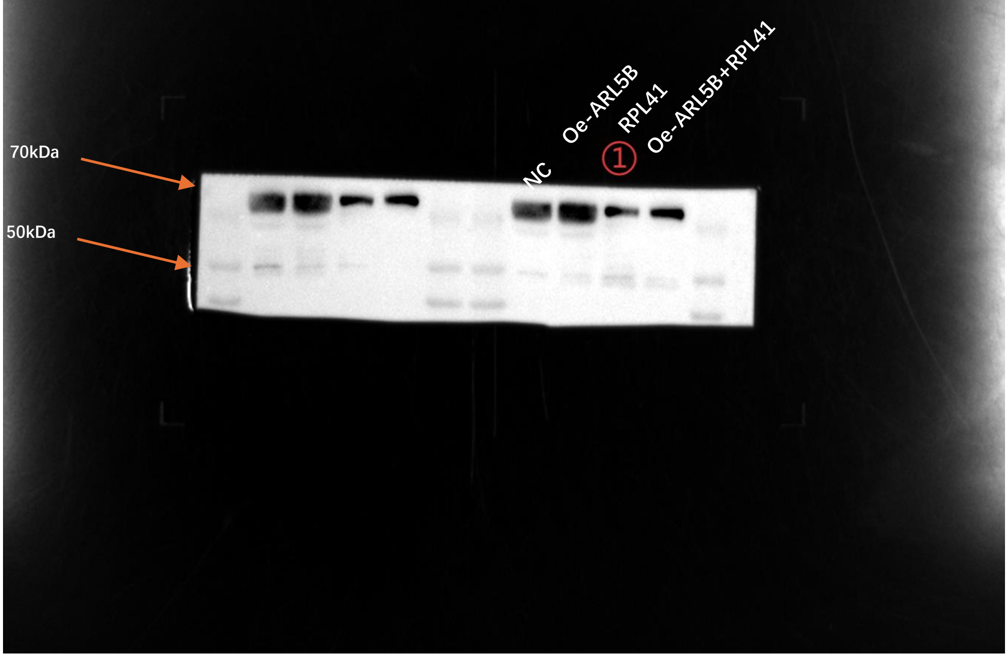

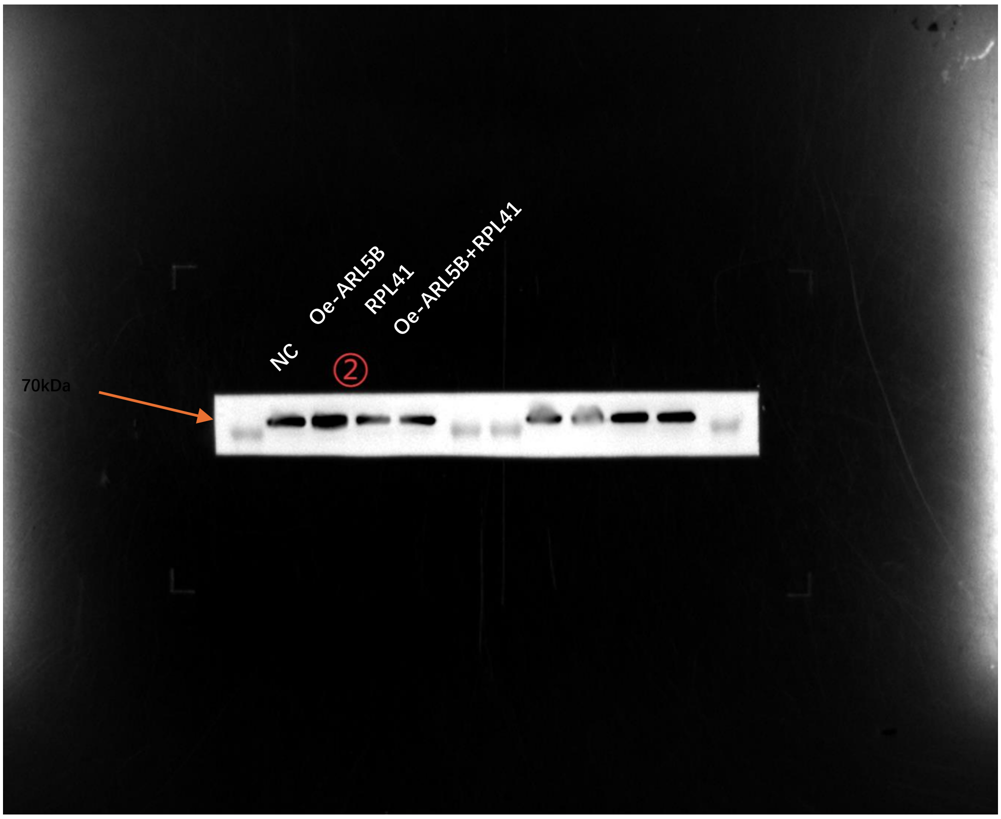

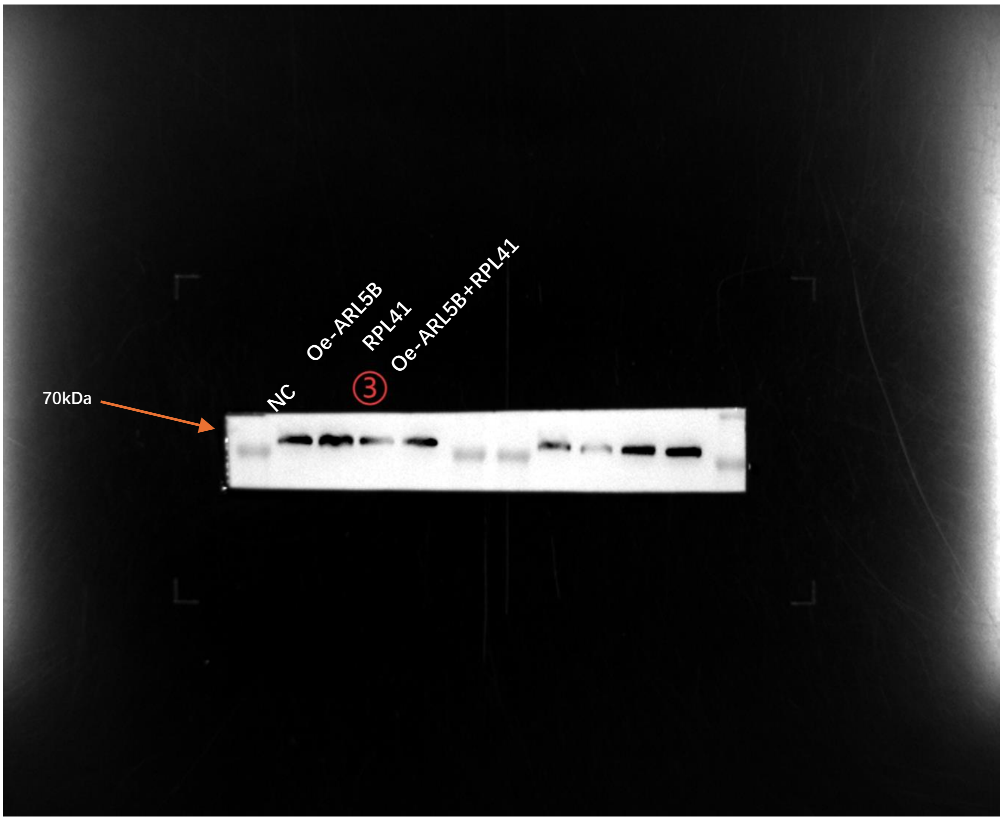

(β-Actin)

The change in β-Actin protein expression was verified by Western blot using a 10% separating gel in the Weri-RB1 cells among the control group, overexpression-ARL5B group, RPL41 intervention group and overexpression-ARL5B with RPL41 intervention group.

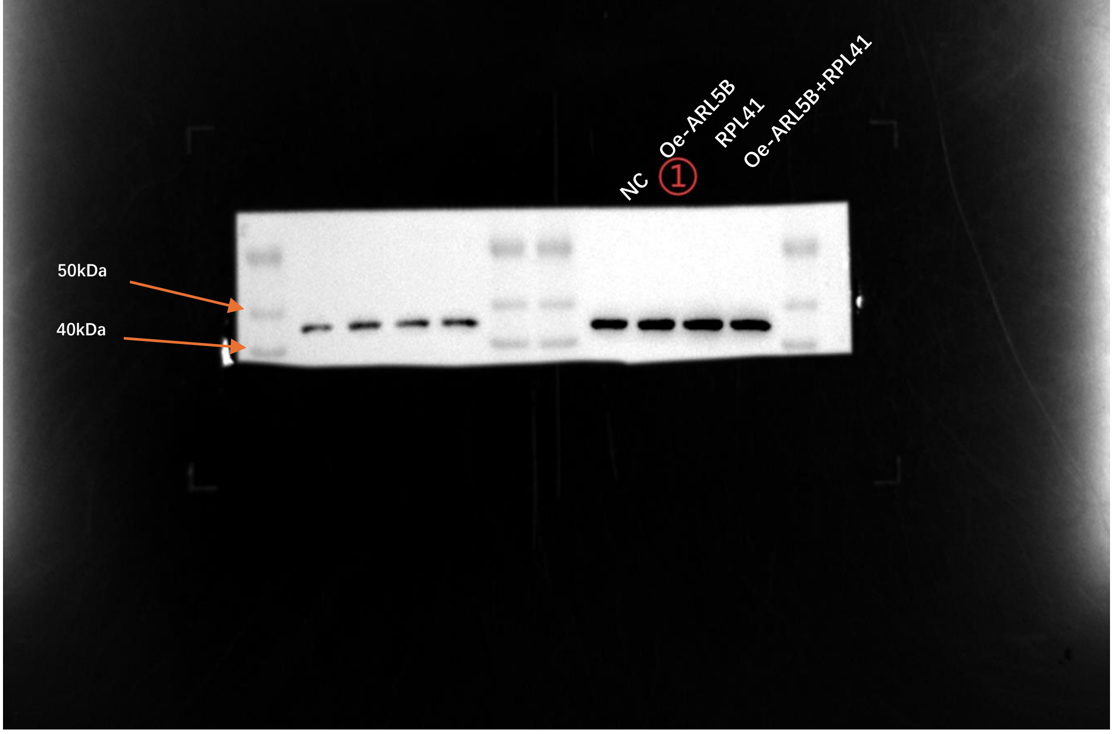

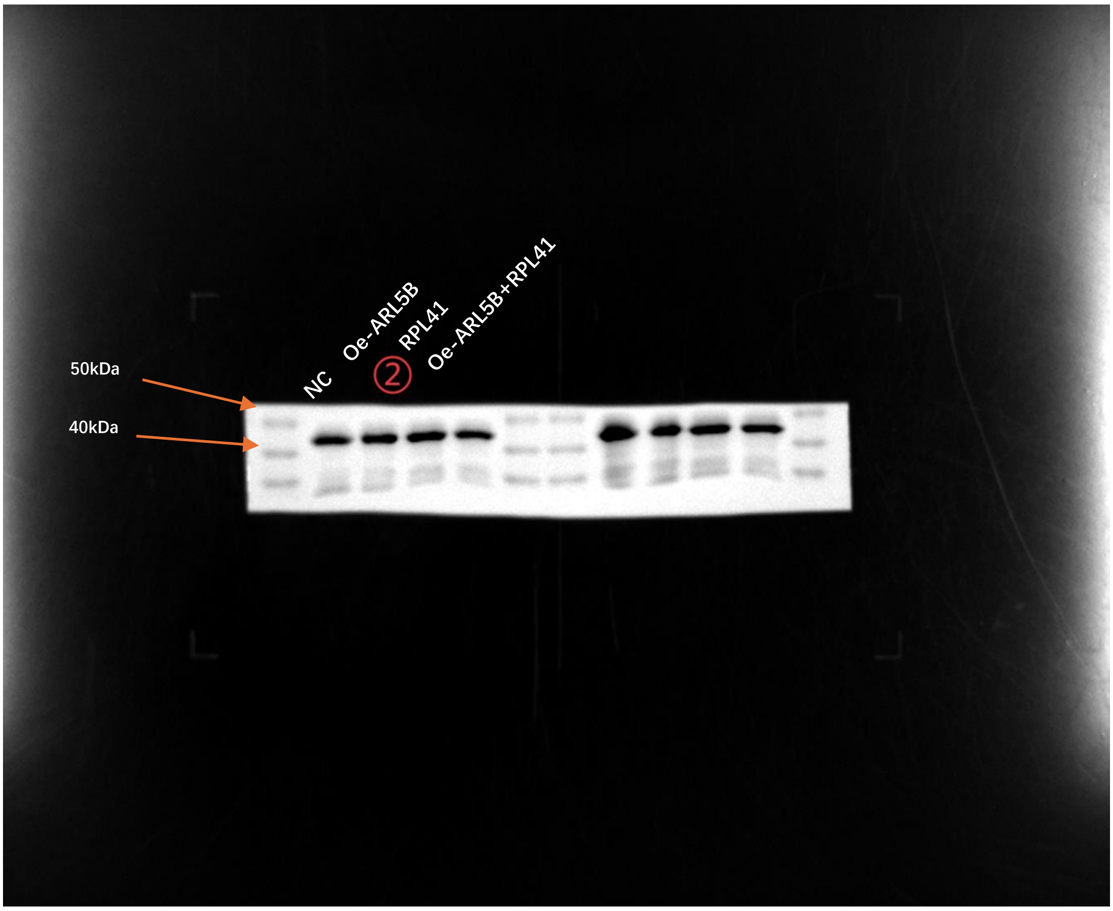
NC
Oe-ARL5B
RPL41
Oe-ARL5B+RPL41
2
50kDa
40kDa

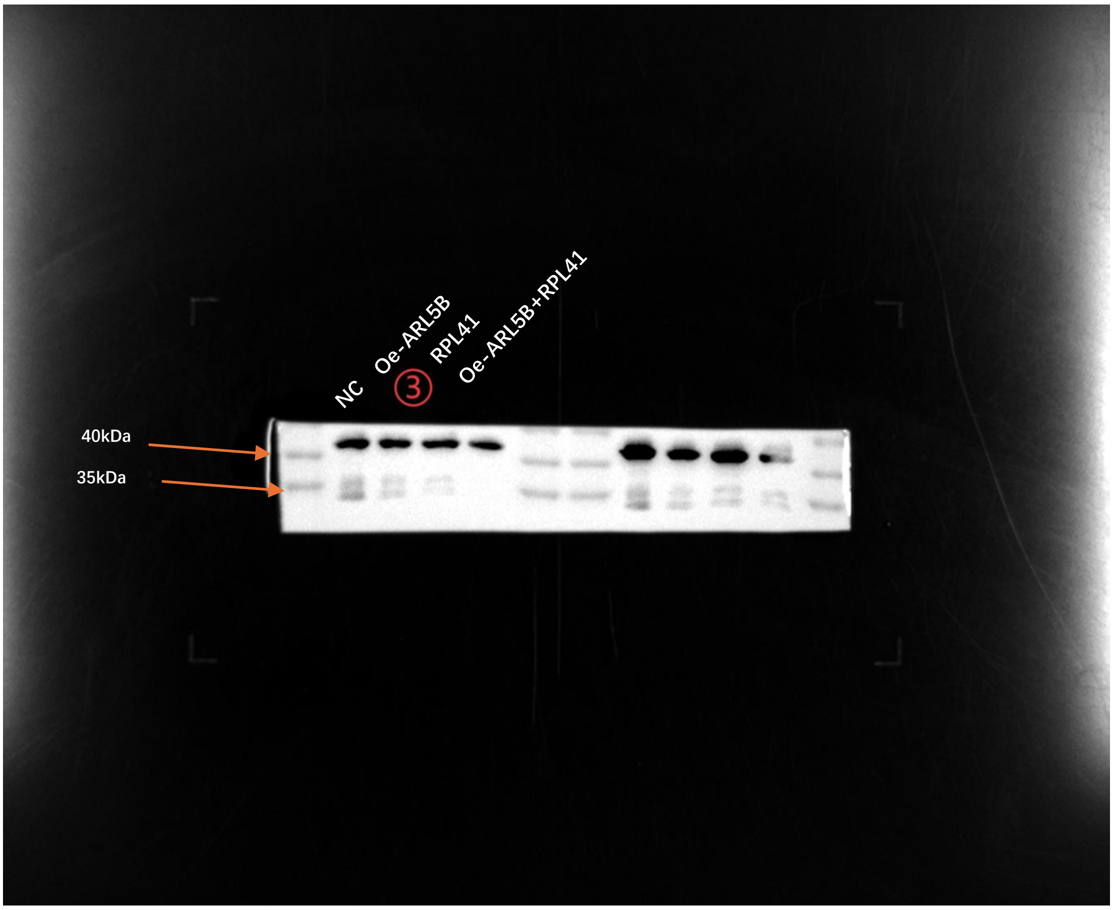
NC
Oe-ARL5B
RPL41
Oe-ARL5B+RPL41
3
40kDa
35kDa

(MERGE)

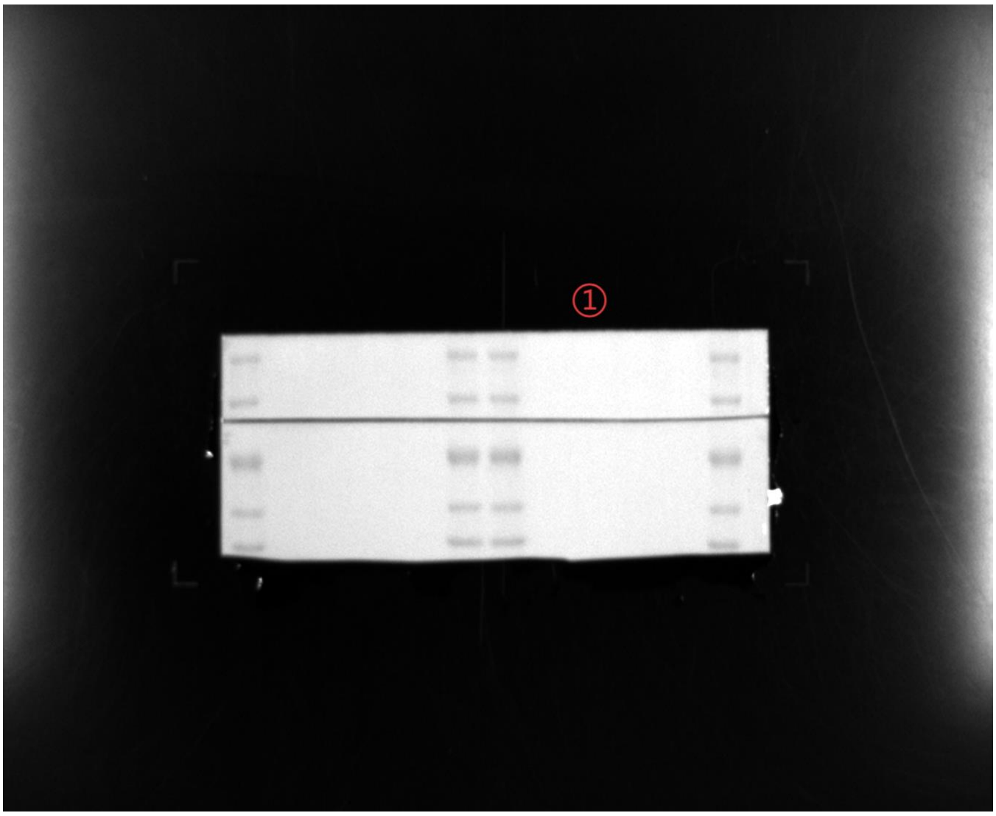

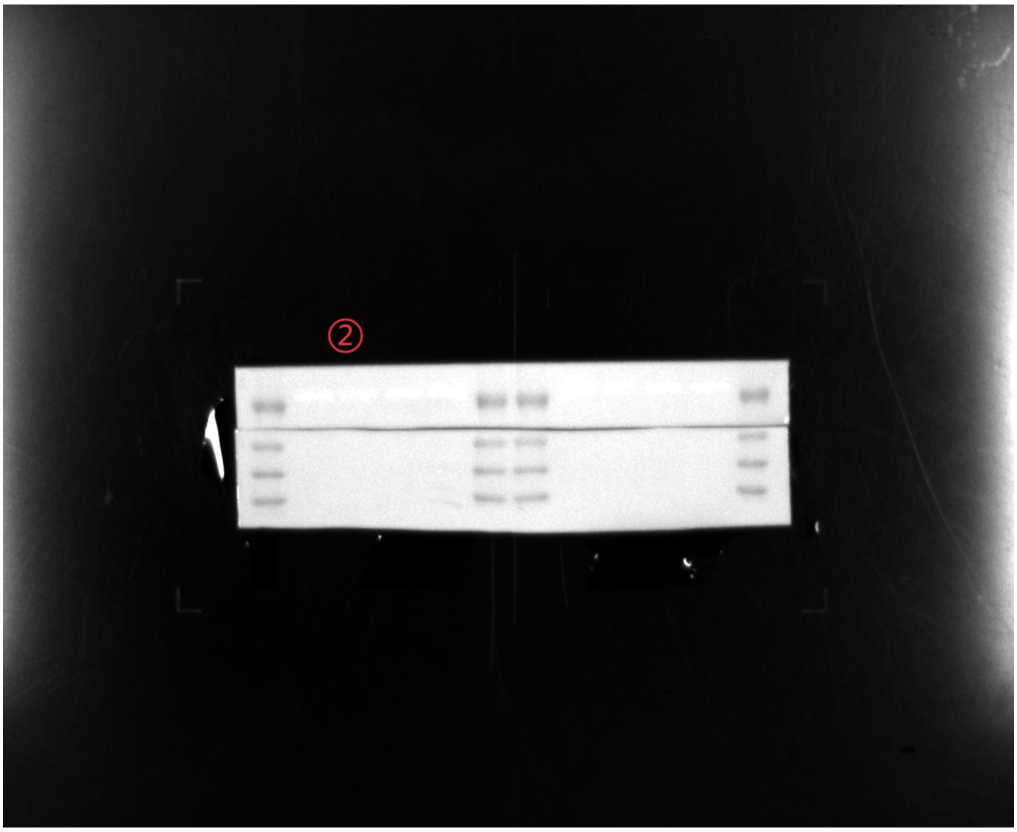

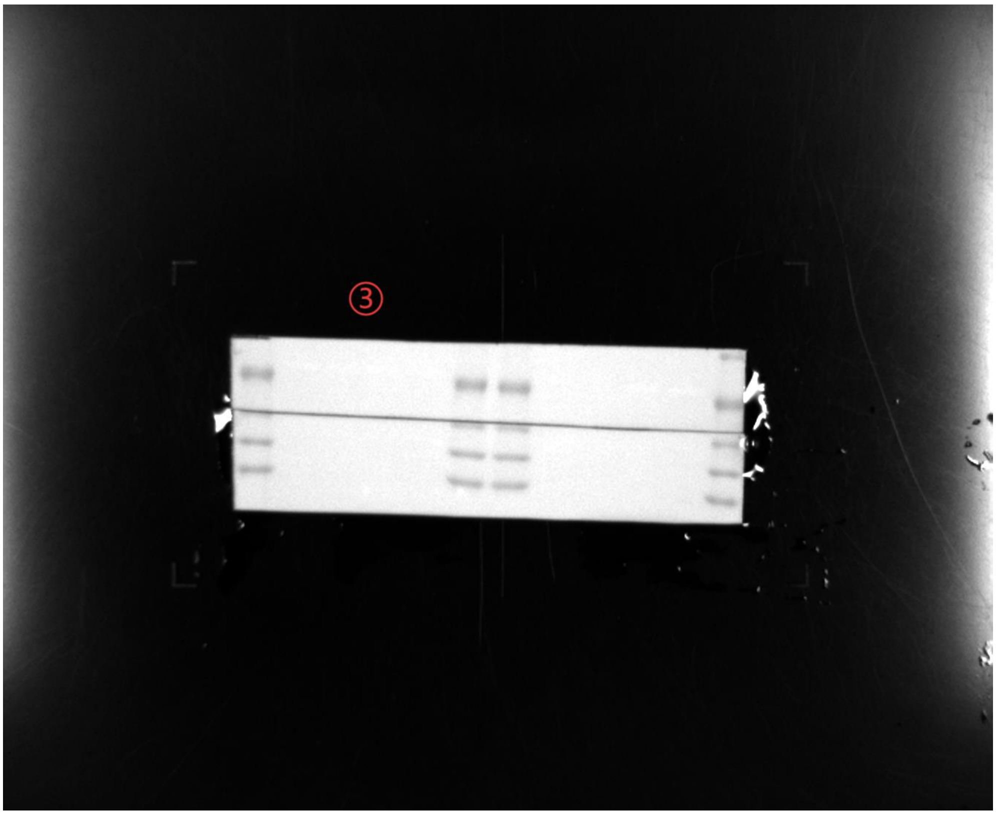

FIGURE-7

Y79(Transfection efficiency-siATF4)

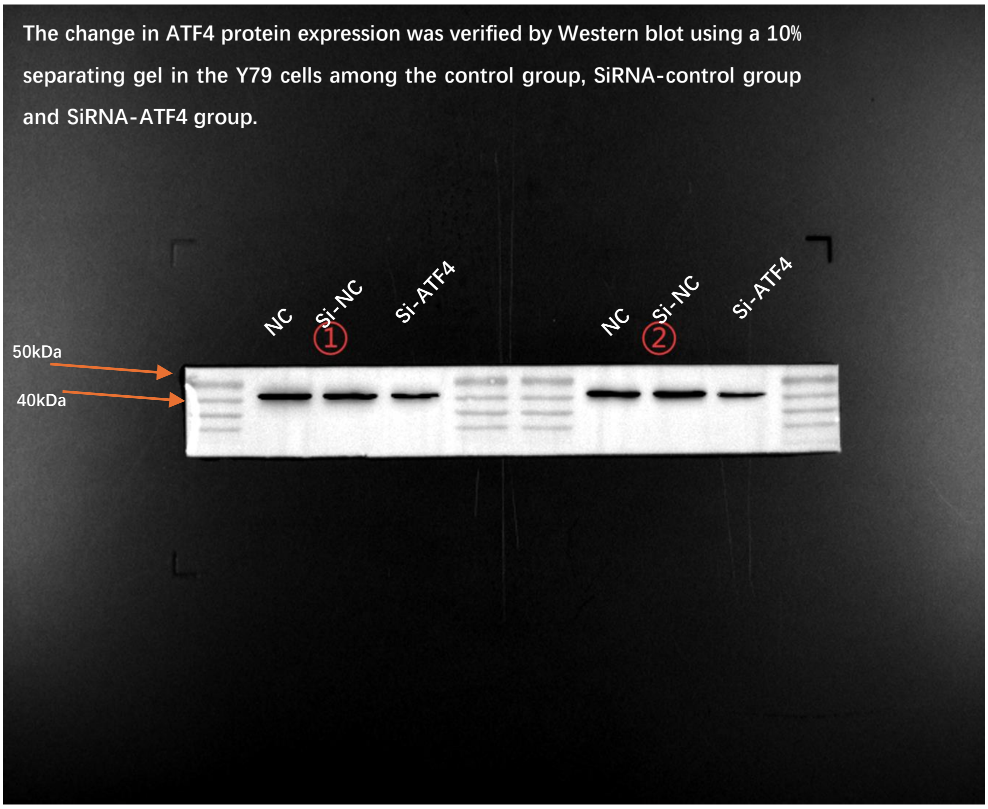

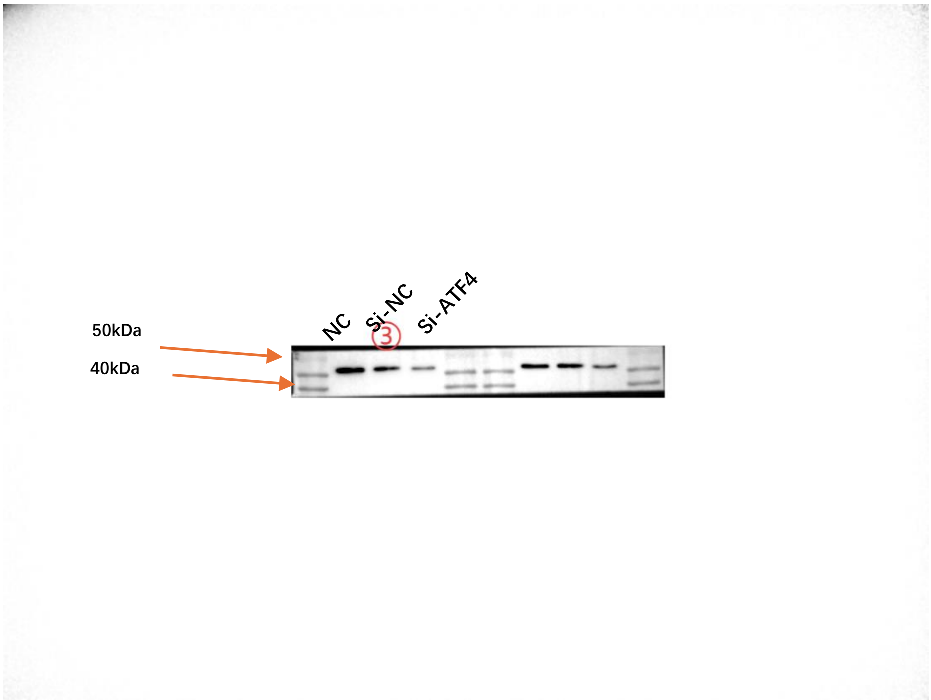
50kDa
40kDa
NC
Si-NC
③
Si-ATF4

(β-Actin)

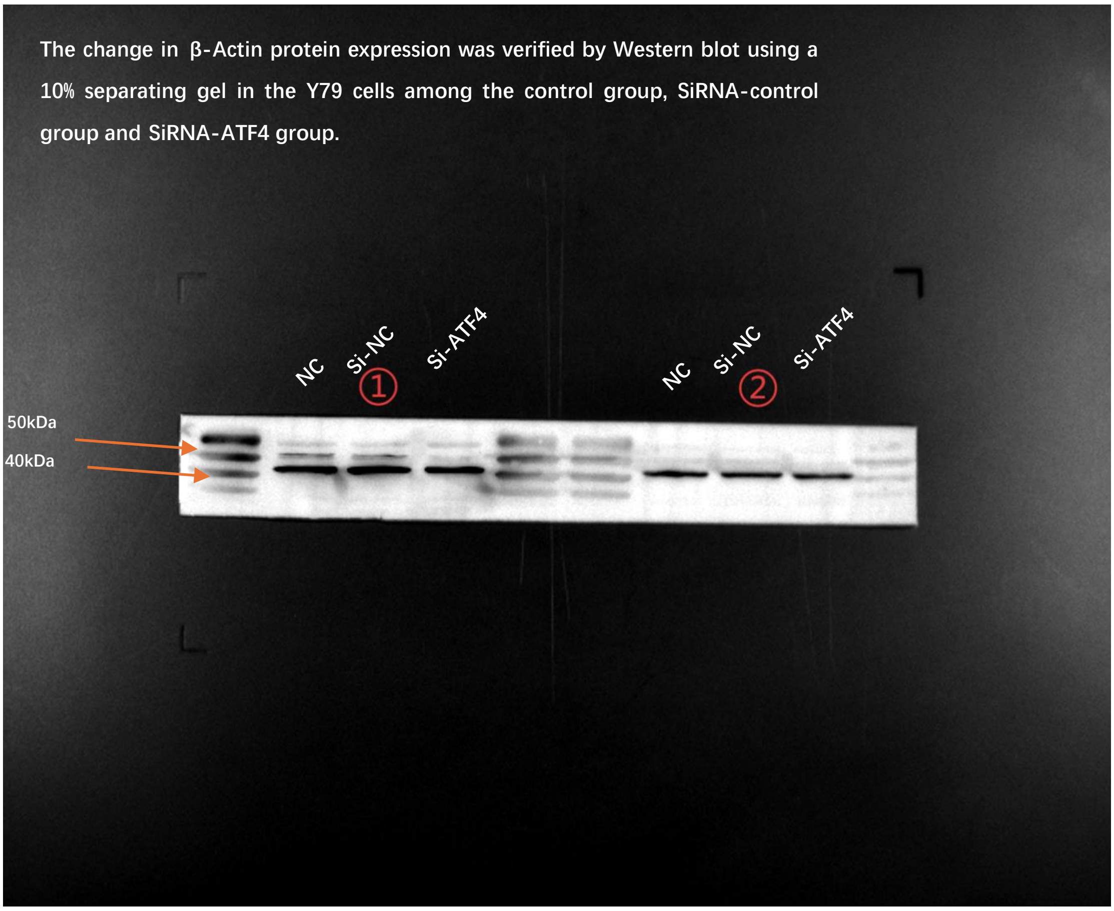
The change in β-Actin protein expression was verified by Western blot using a 10% separating gel in the Y79 cells among the control group, SiRNA-control group and SiRNA-ATF4 group.
50kDa
40kDa
NC
Si-NC
①
Si-ATF4
NC
Si-NC
②
Si-ATF4

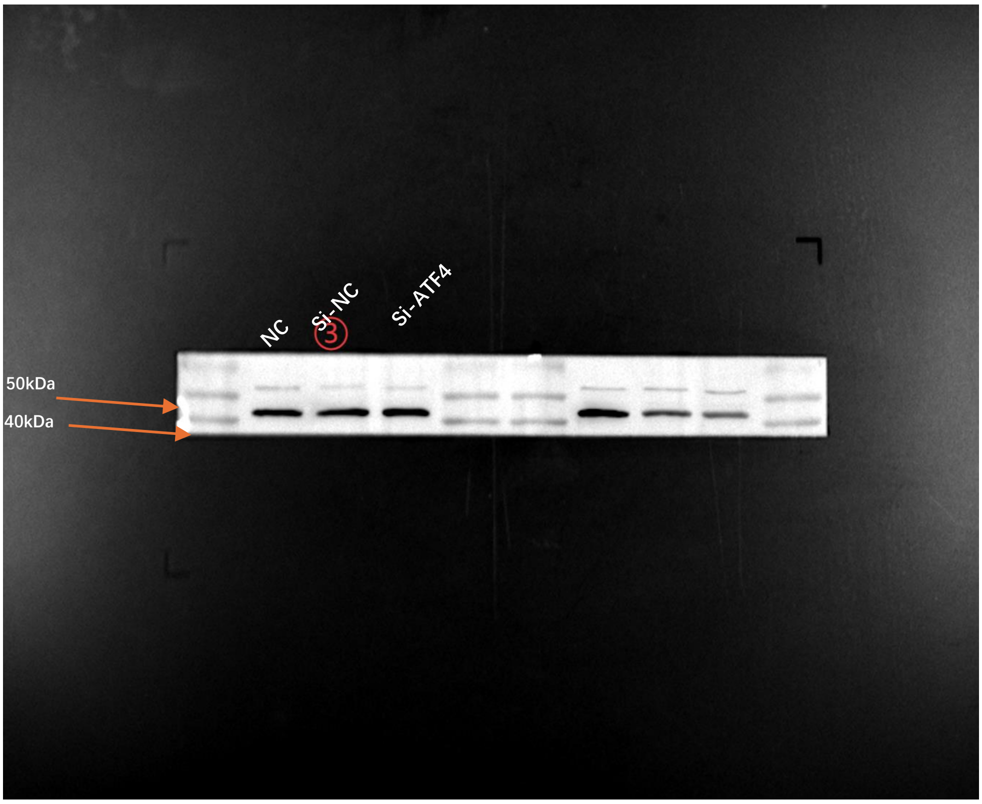

Weri-RB1 (Transfection efficiency-si-ATF4)

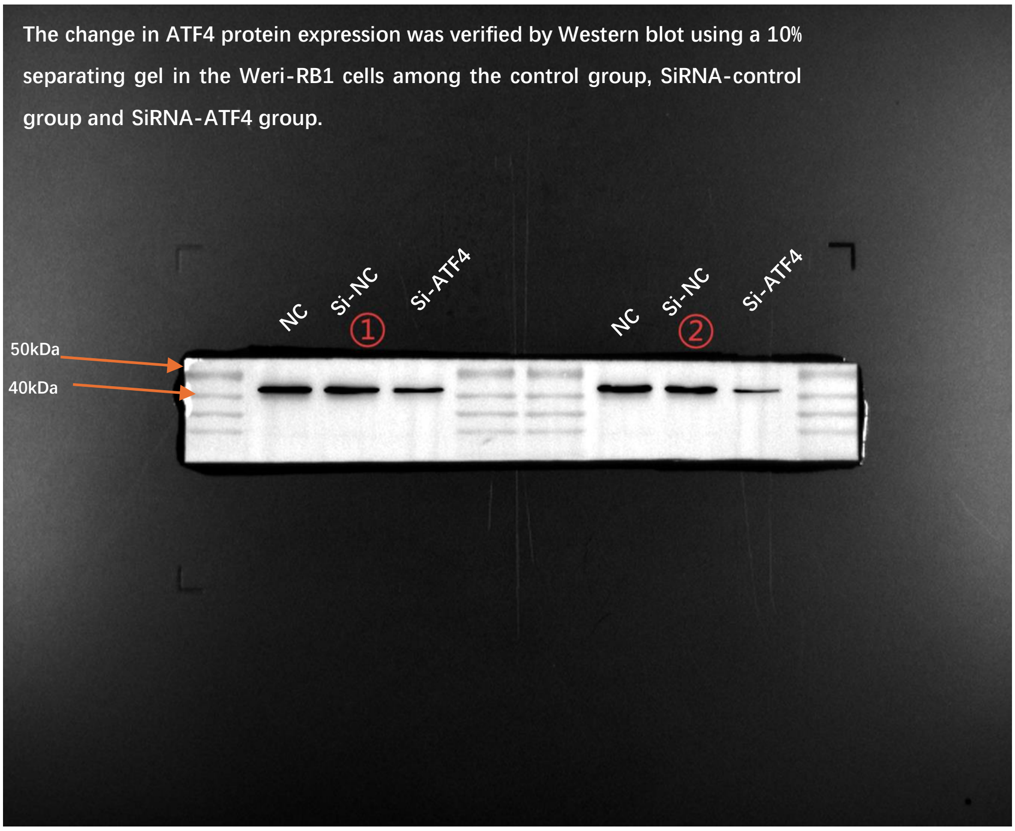

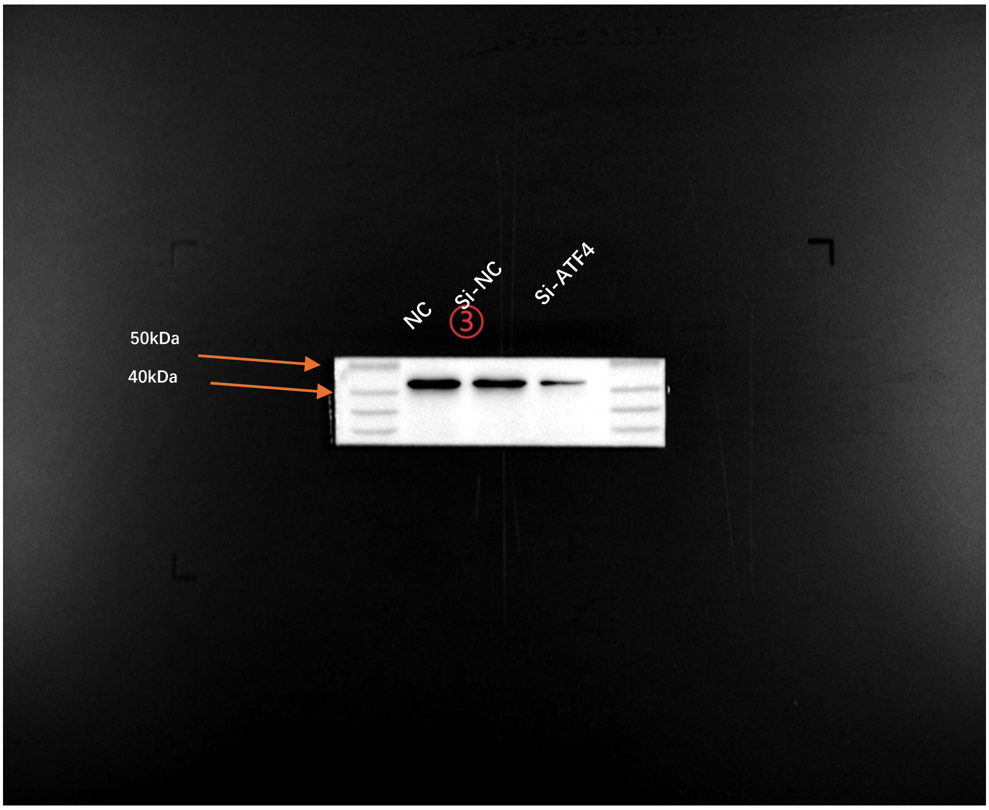

(β-Actin)

The change in β-Actin protein expression was verified by Western blot using a 10% separating gel in the Weri-RB1 cells among the control group, SiRNA-control group and SiRNA-ATF4 group.

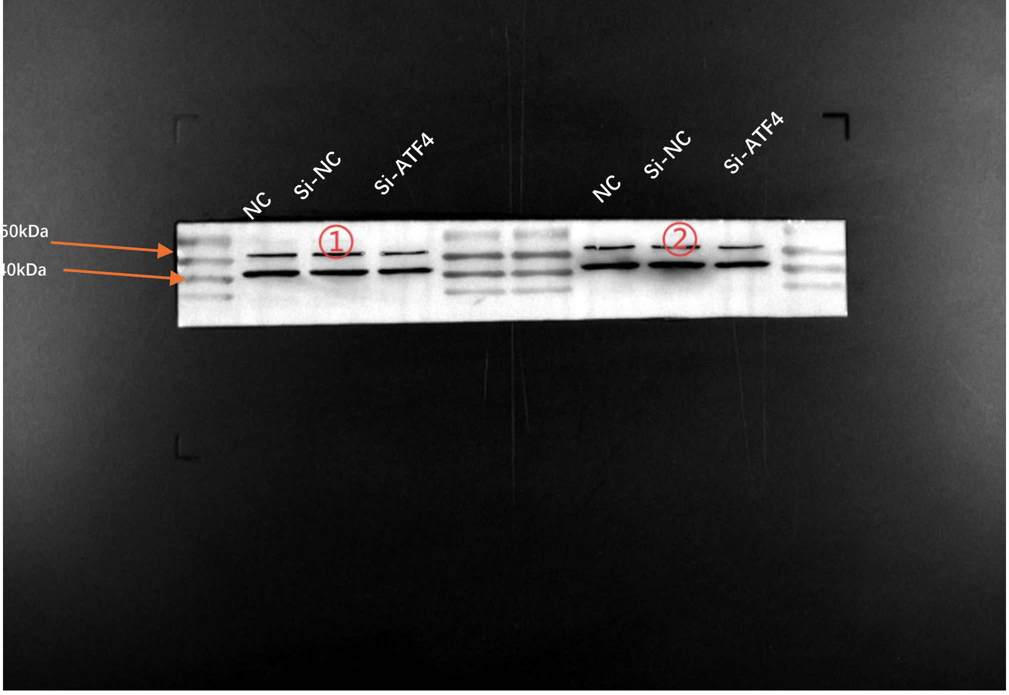

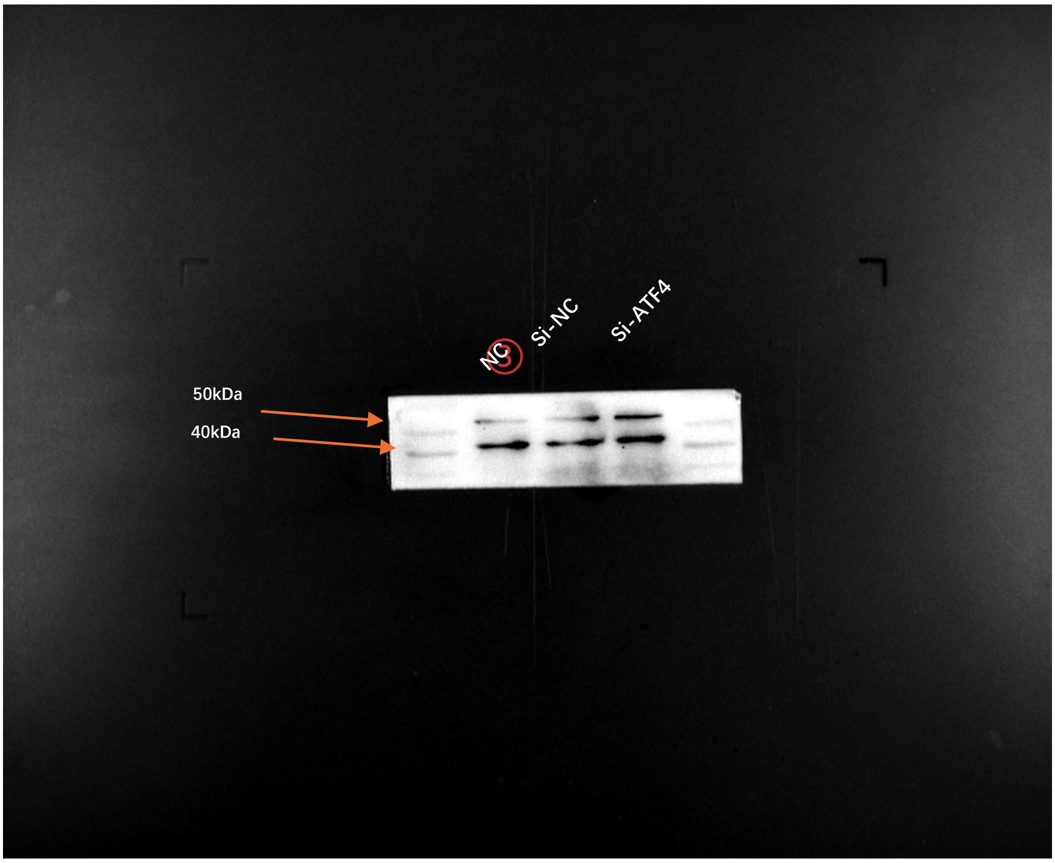

Y79(ARL5B)

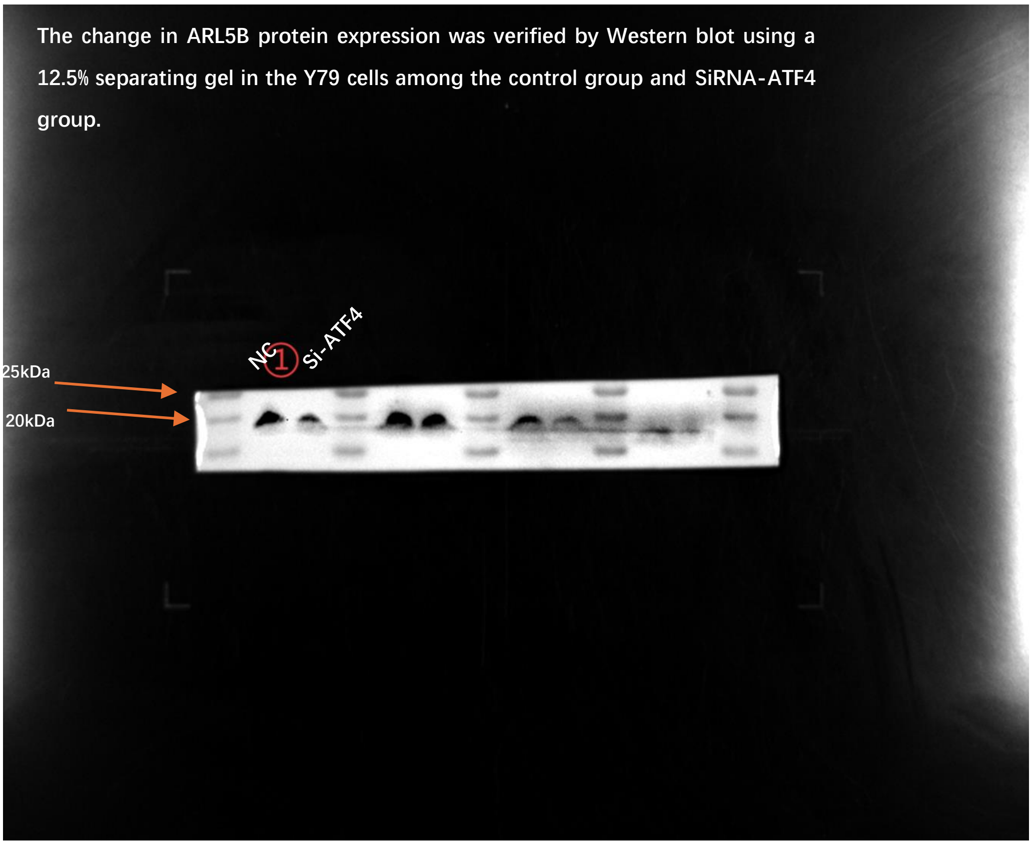

The change in ARL5B protein expression was verified by Western blot using a 12.5% separating gel in the Y79 cells among the control group and SiRNA-ATF4 group.

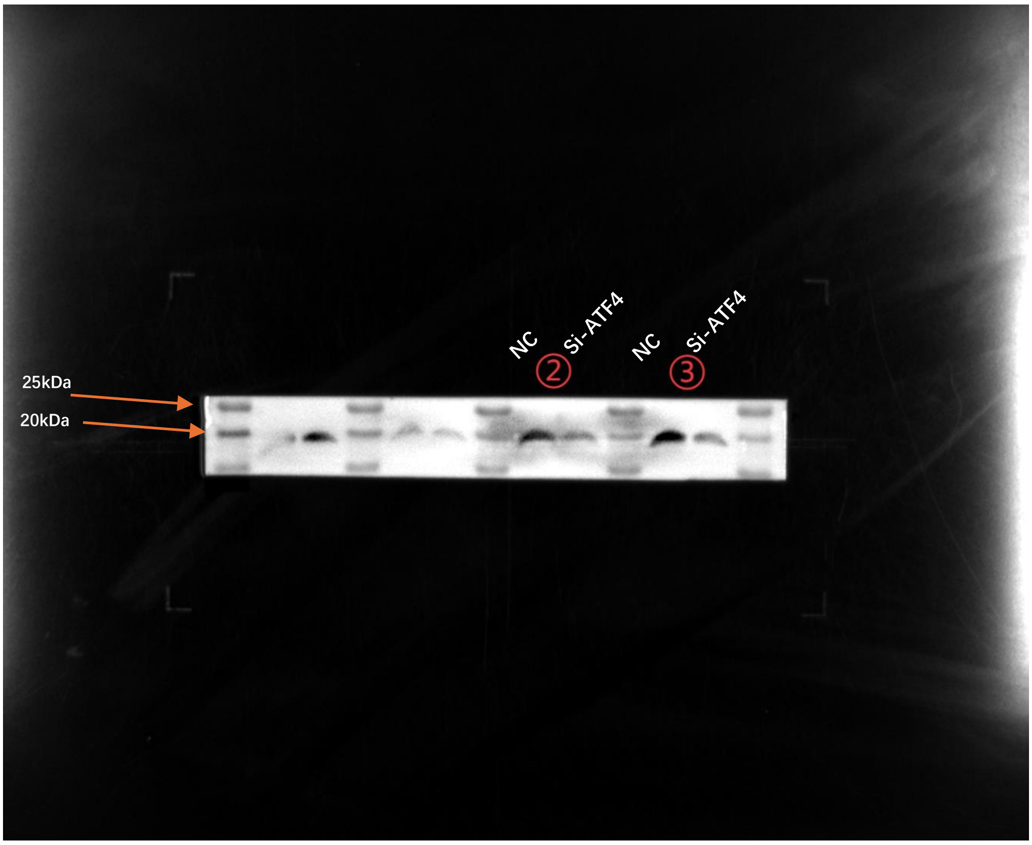
25kDa
20kDa
NC
Si-ATF4
②
NC
Si-ATF4
③

(β-Actin)

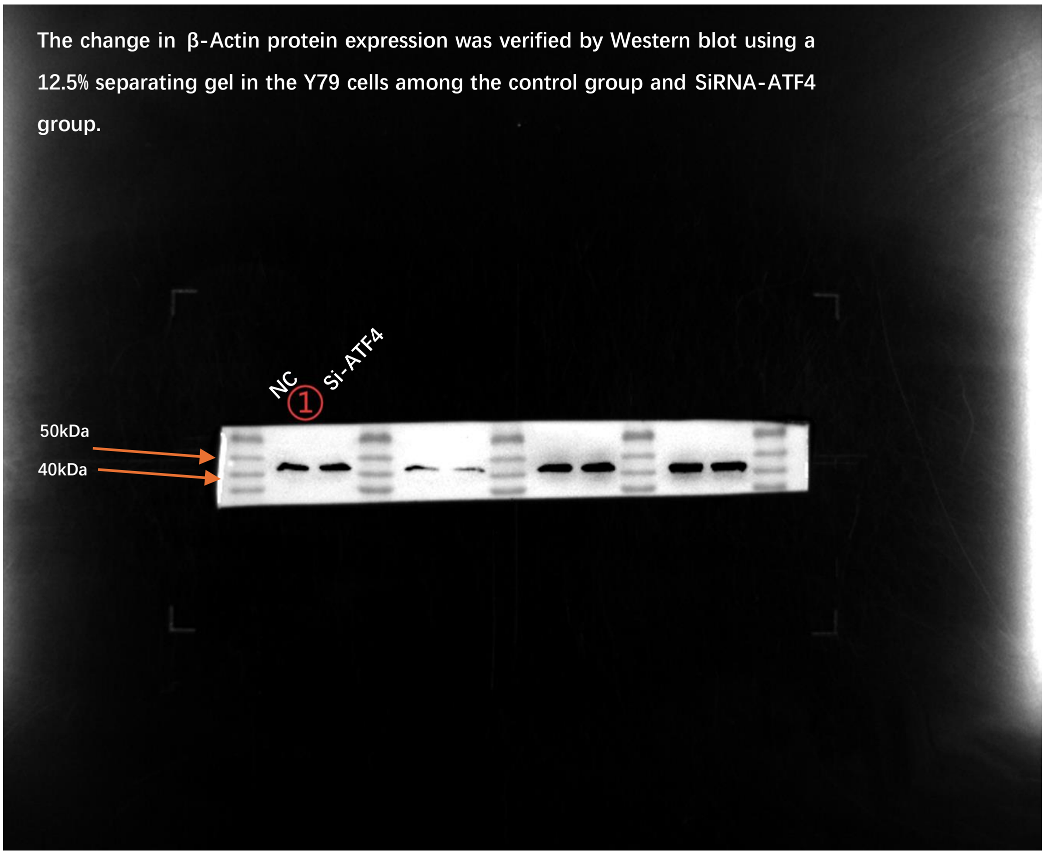
The change in β-Actin protein expression was verified by Western blot using a 12.5% separating gel in the Y79 cells among the control group and SiRNA-ATF4 group.
50kDa
40kDa
NC
Si-ATF4
①

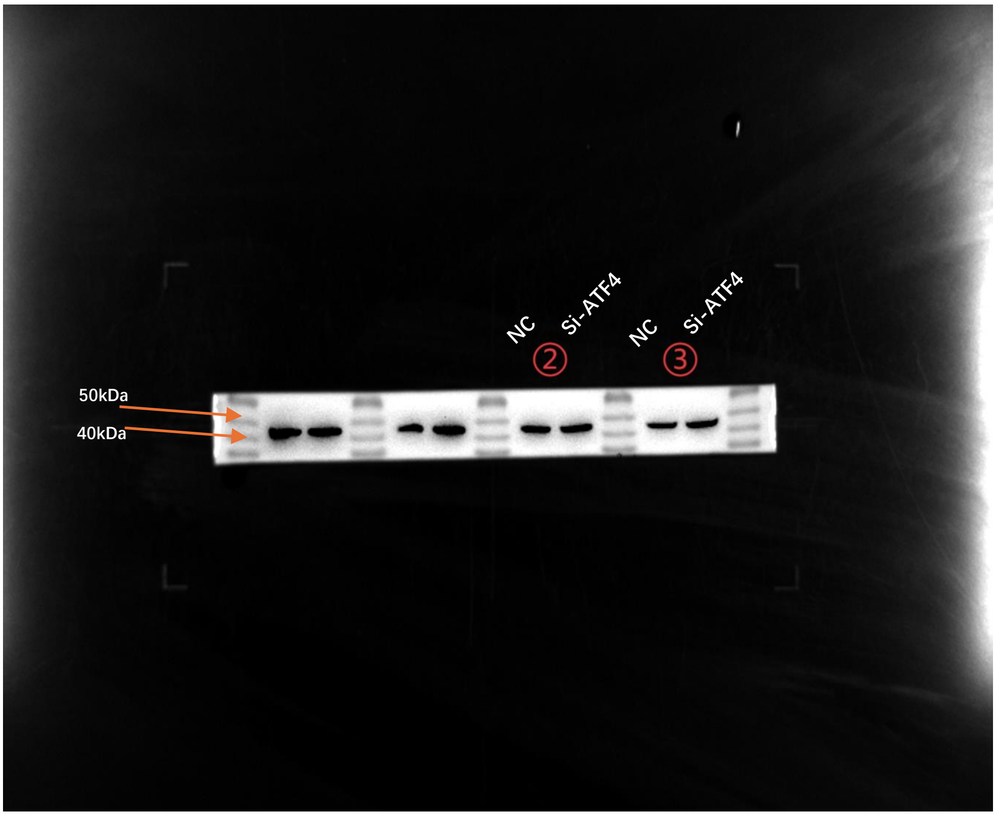

(MERGE)

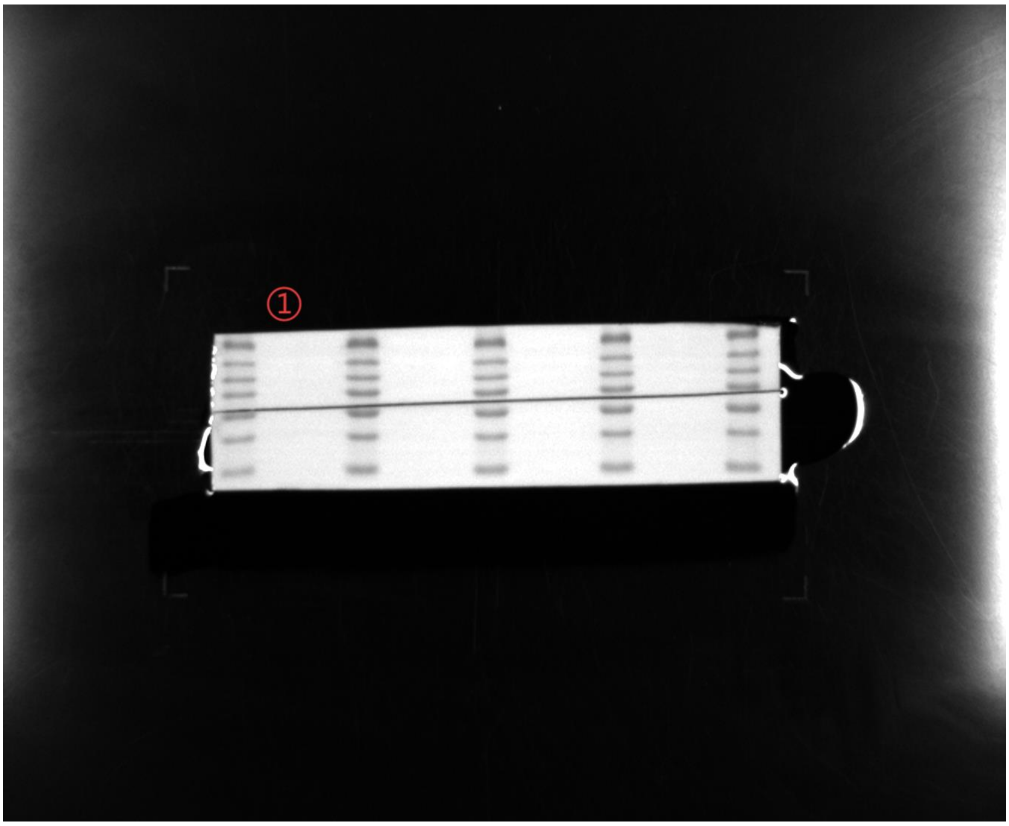

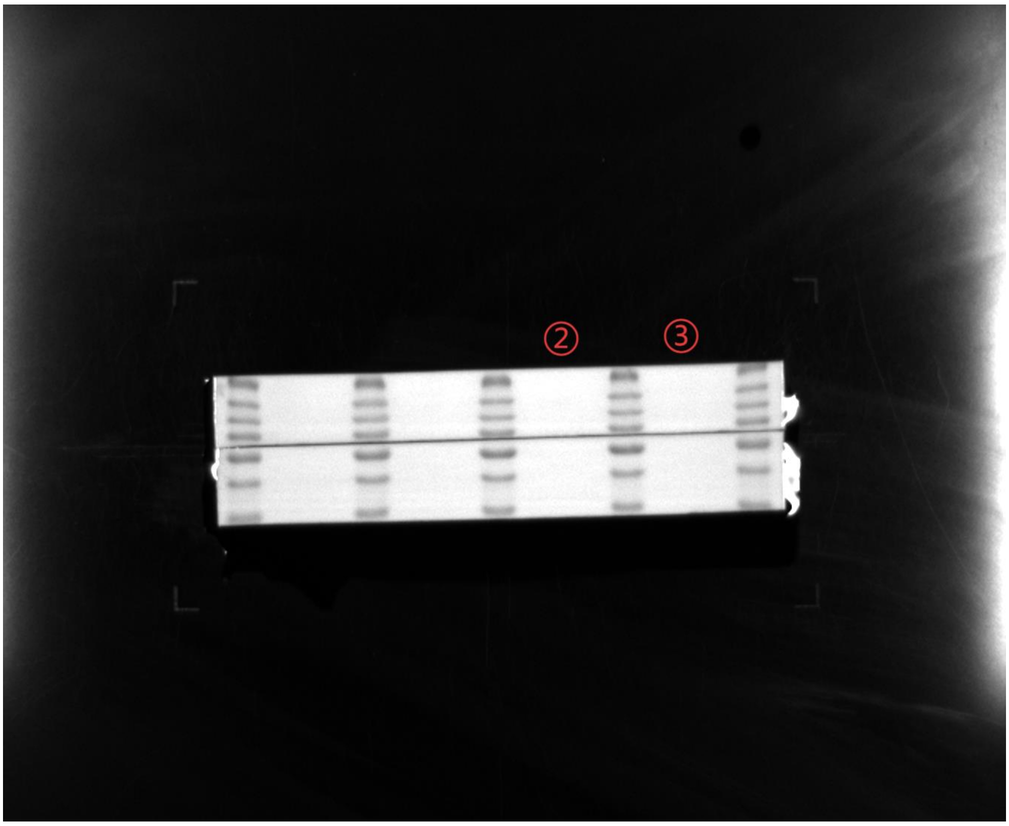

Weri-RB1(ARL5B)

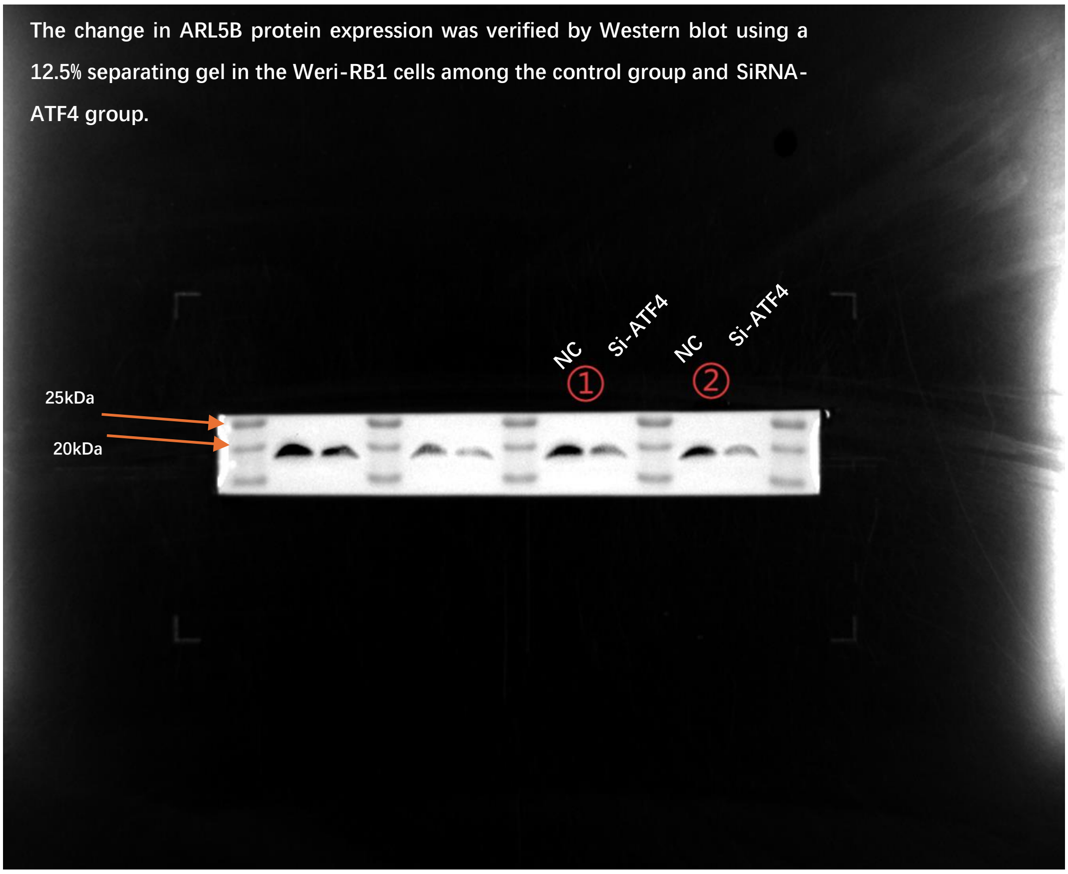

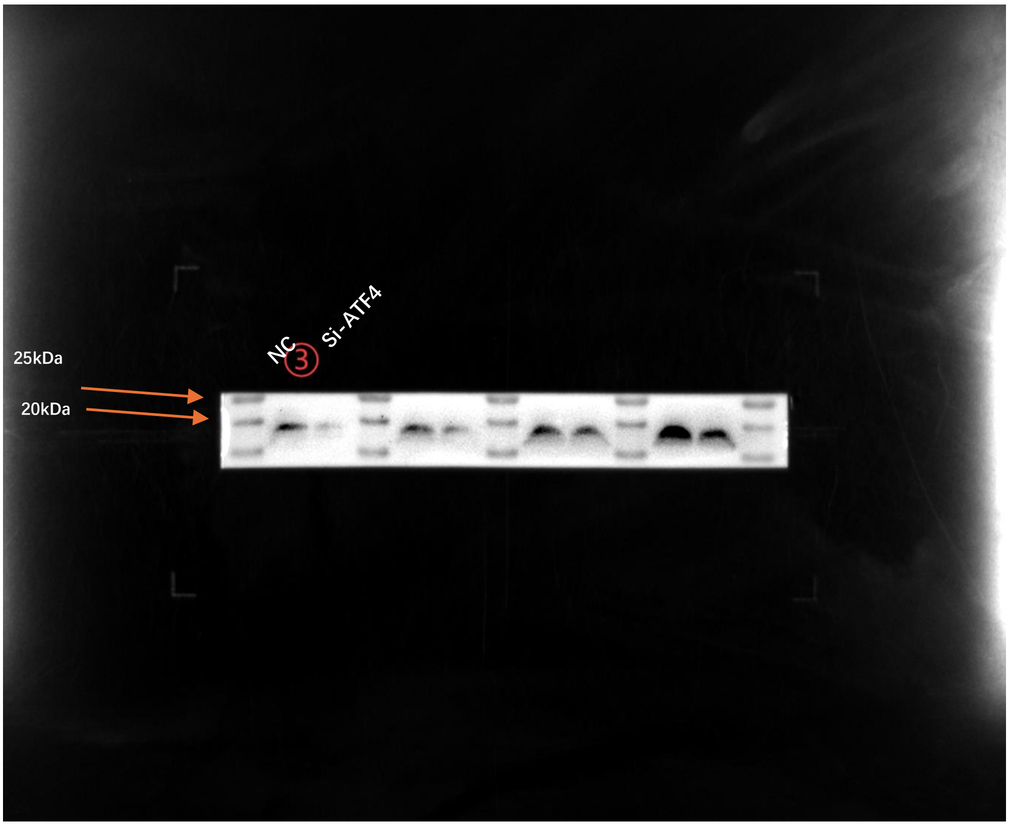

(β-Actin)

The change in β-Actin protein expression was verified by Western blot using a 12.5% separating gel in the Weri-RB1 cells among the control group and SiRNA-ATF4 group.

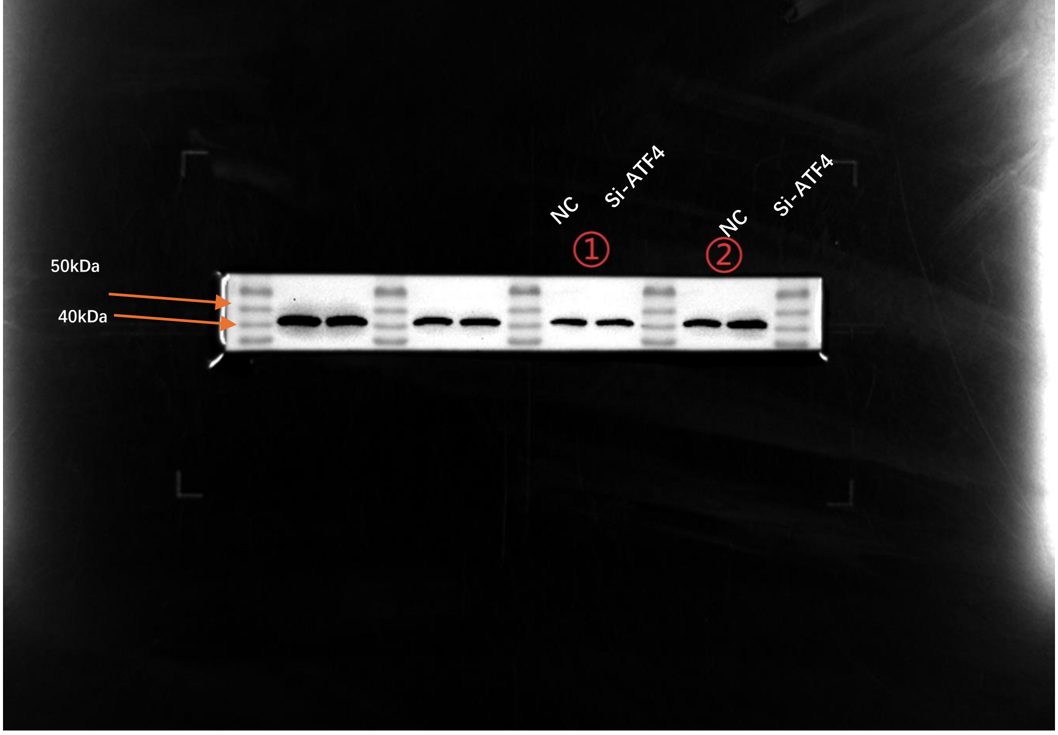

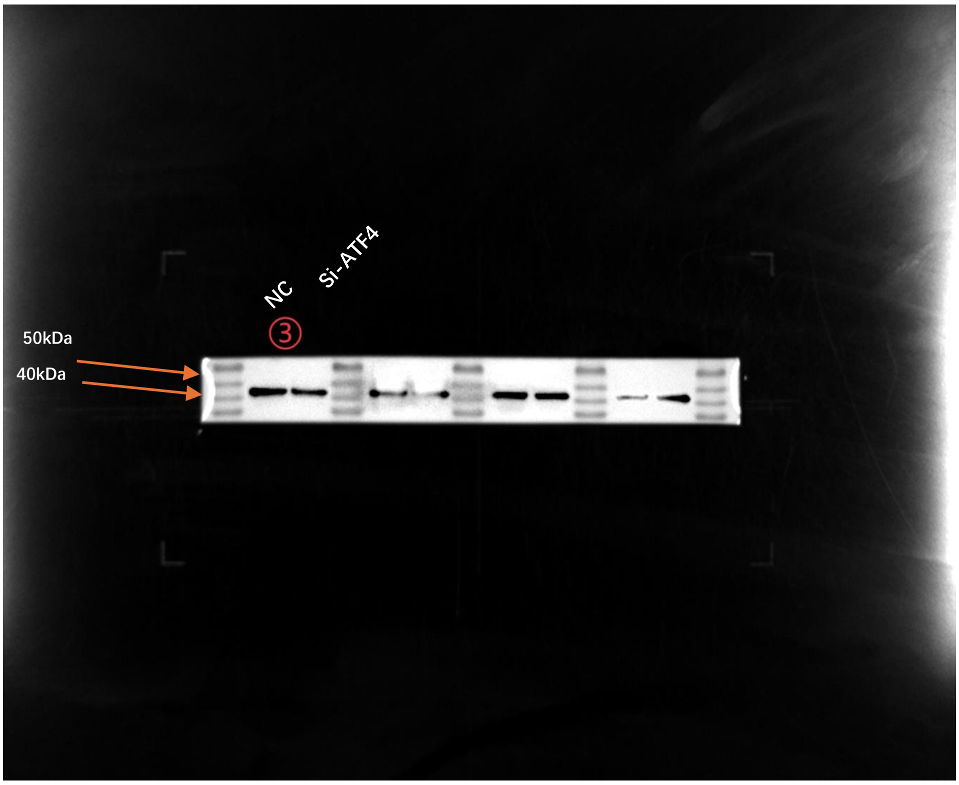

(MERGE)

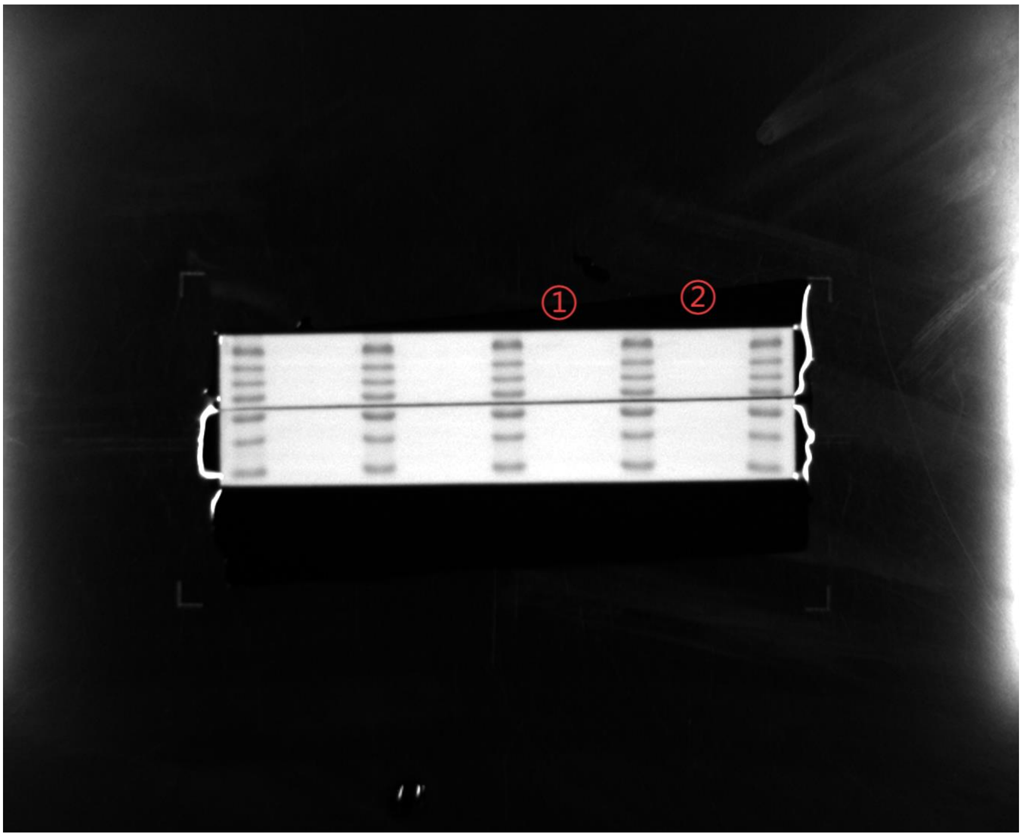

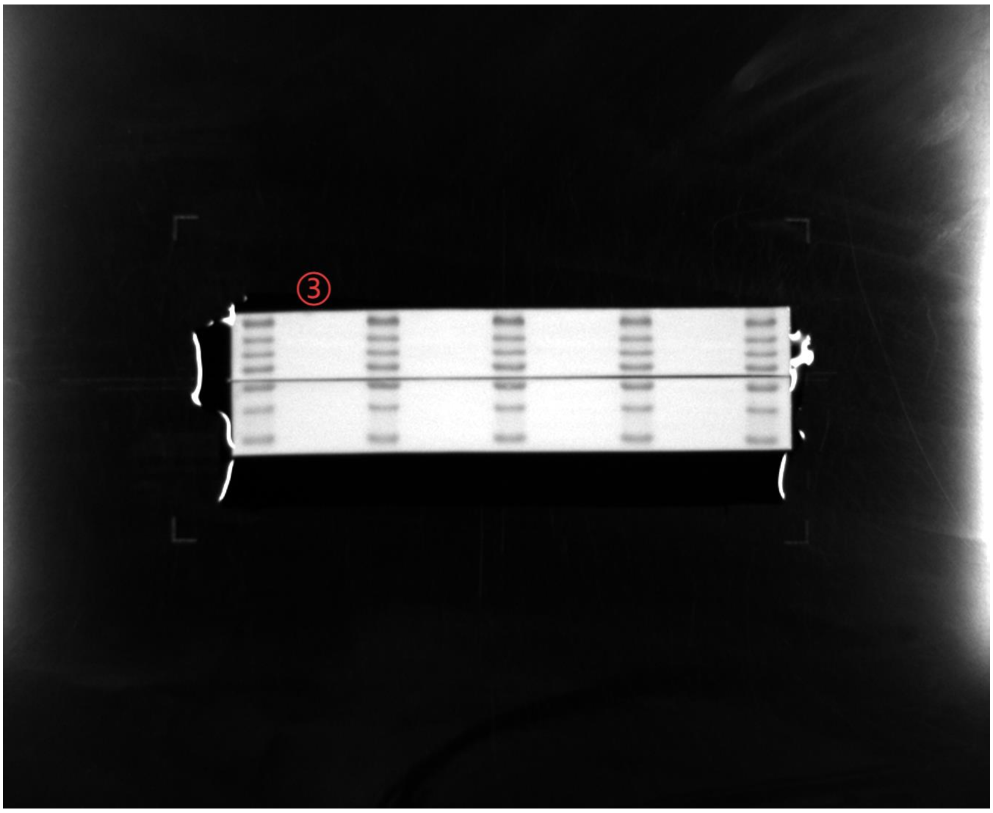
③

FIGURE-8

Y79(ATF4)

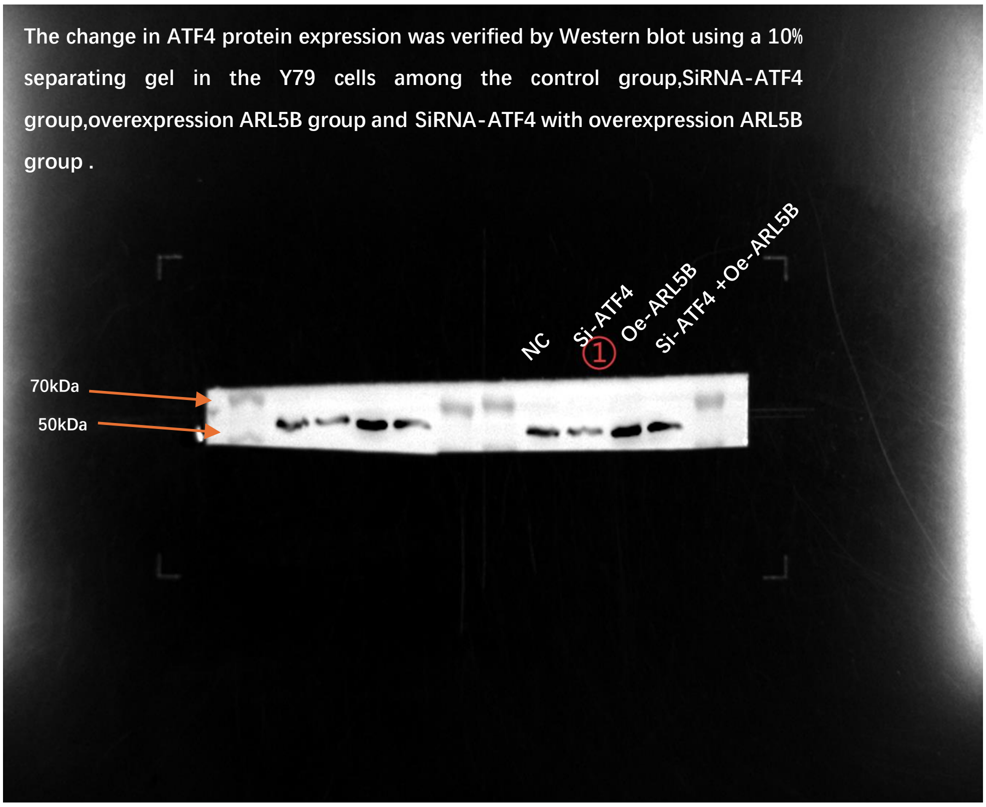

The change in ATF4 protein expression was verified by Western blot using a 10% separating gel in the Y79 cells among the control group,SiRNA-ATF4 group,overexpression ARL5B group and SiRNA-ATF4 with overexpression ARL5B group .

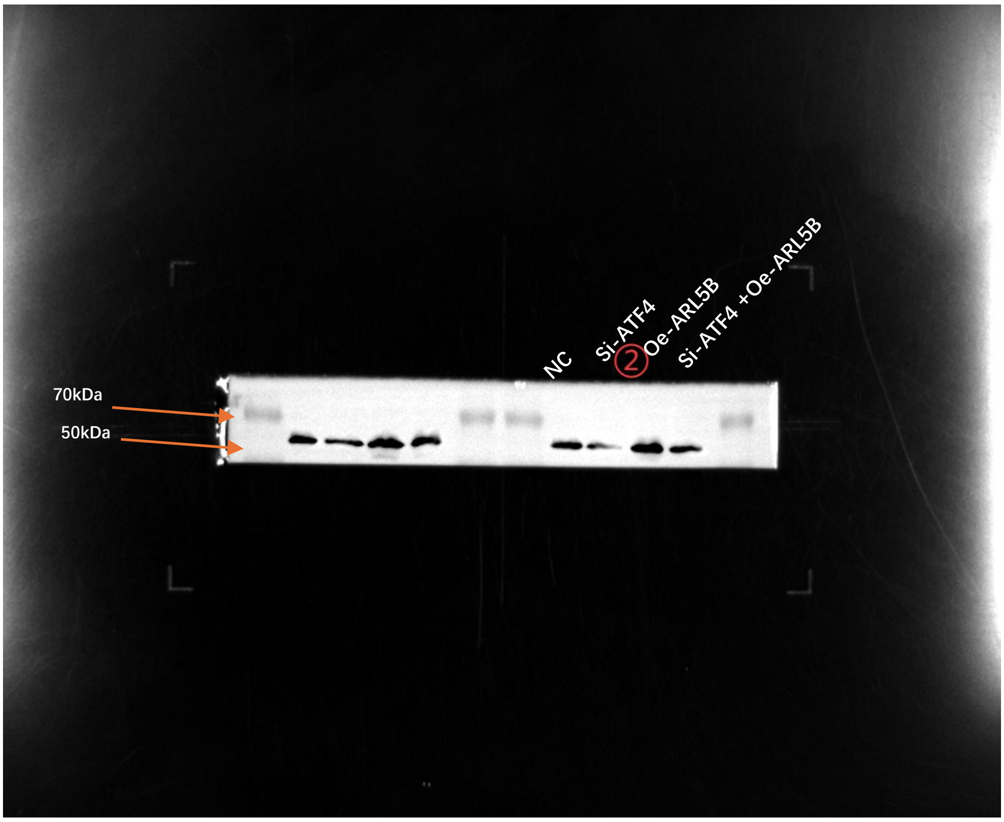

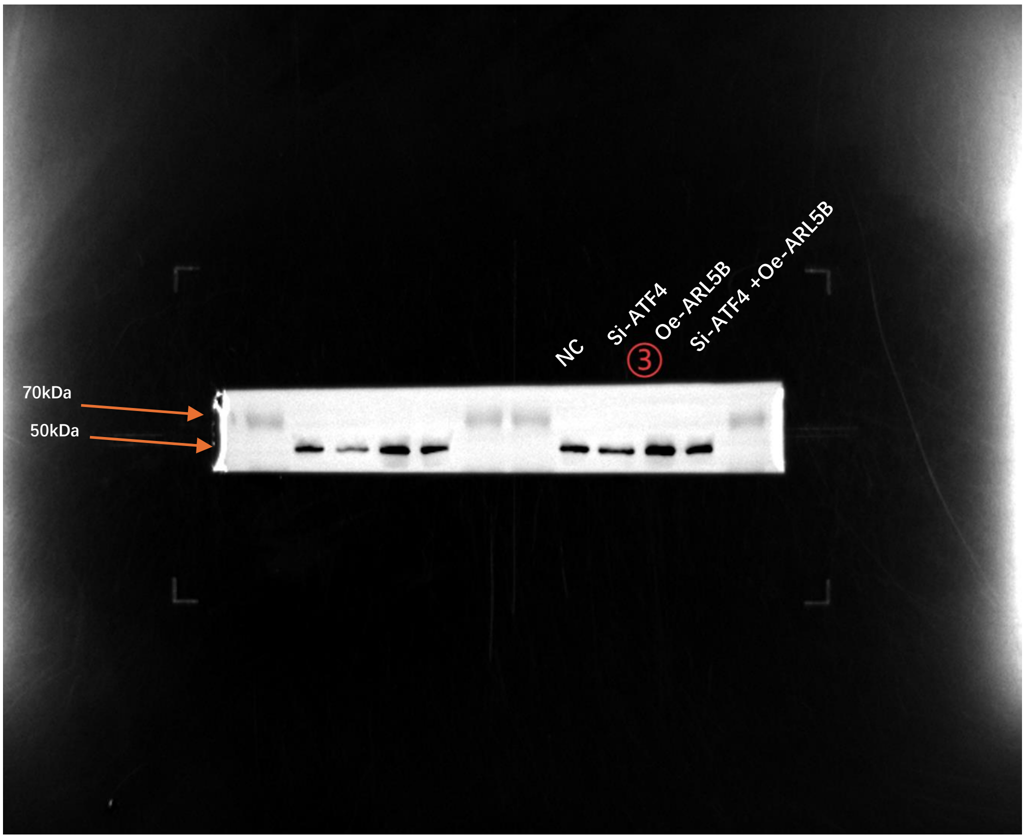
NC
Si-ATF4
Oe-ARL5B
Si-ATF4 +Oe-ARL5B
③
70kDa
50kDa

(β-Actin)

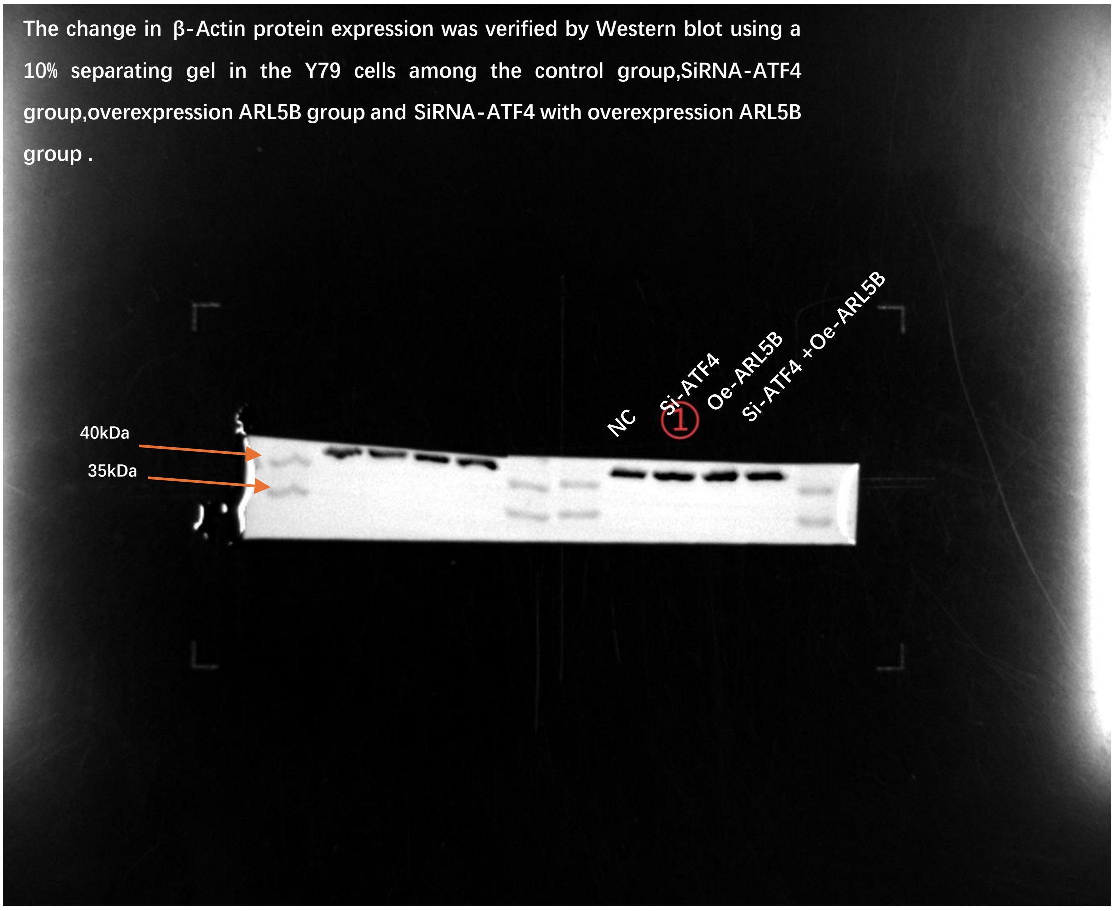
The change in β-Actin protein expression was verified by Western blot using a 10% separating gel in the Y79 cells among the control group,SiRNA-ATF4 group,overexpression ARL5B group and SiRNA-ATF4 with overexpression ARL5B group .
NC
Si-ATF4
Oe-ARL5B
Si-ATF4 +Oe-ARL5B
①
40kDa
35kDa

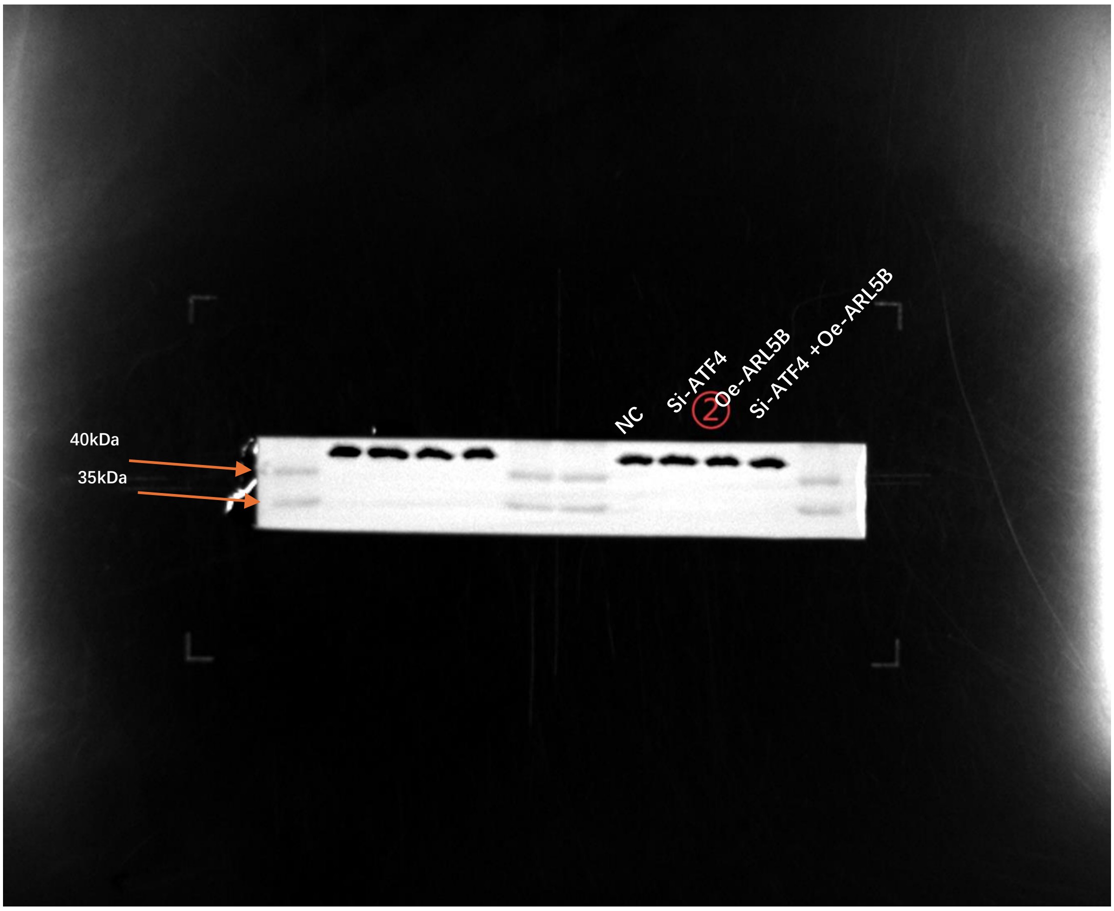
40kDa
35kDa
NC
Si-ATF4
Oe-ARL5B
Si-ATF4 +Oe-ARL5B
②

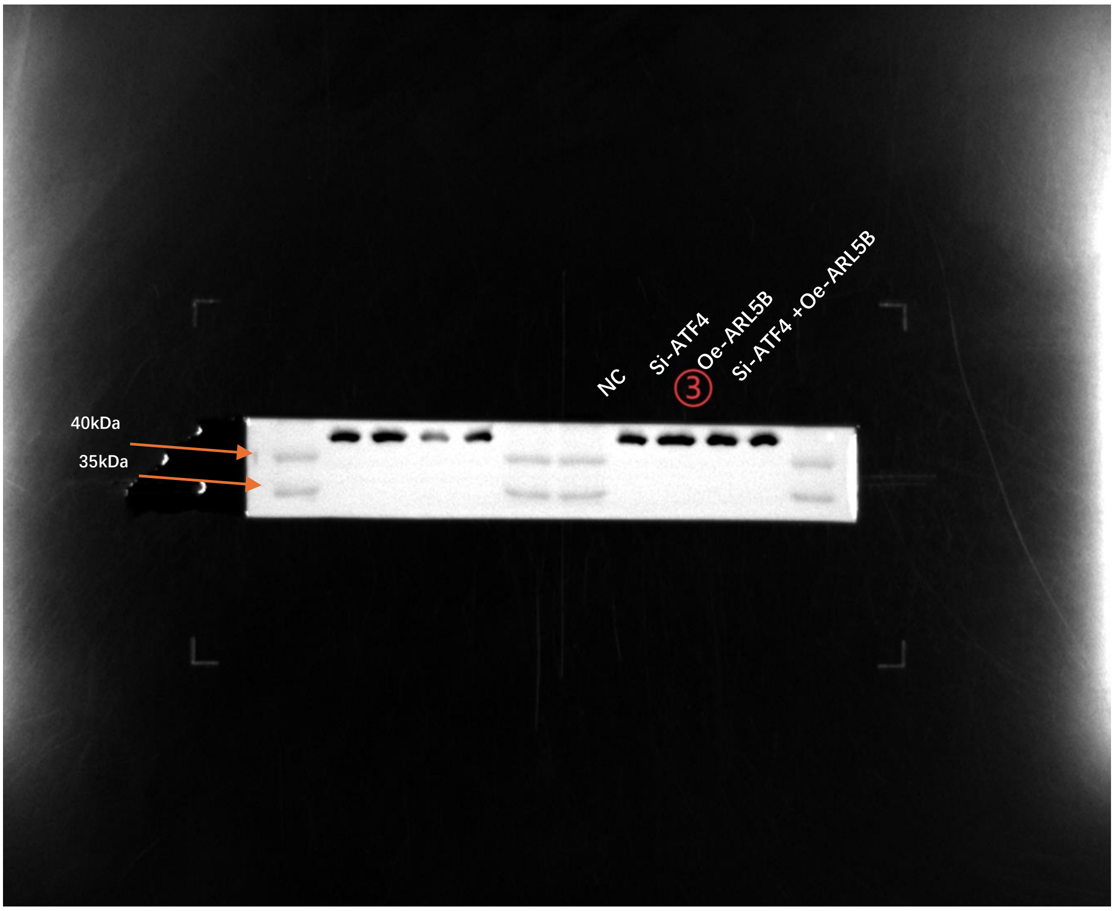
40kDa
35kDa
NC
Si-ATF4
Oe-ARL5B
Si-ATF4 +Oe-ARL5B
③

(MERGE)

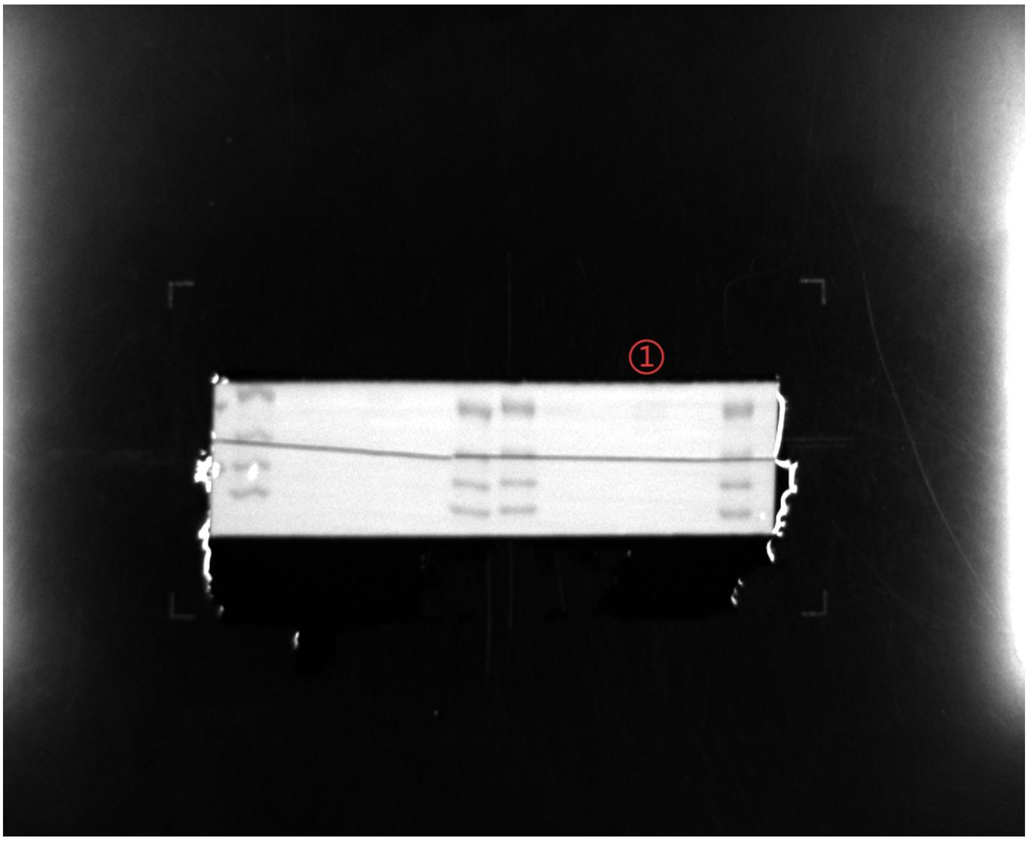

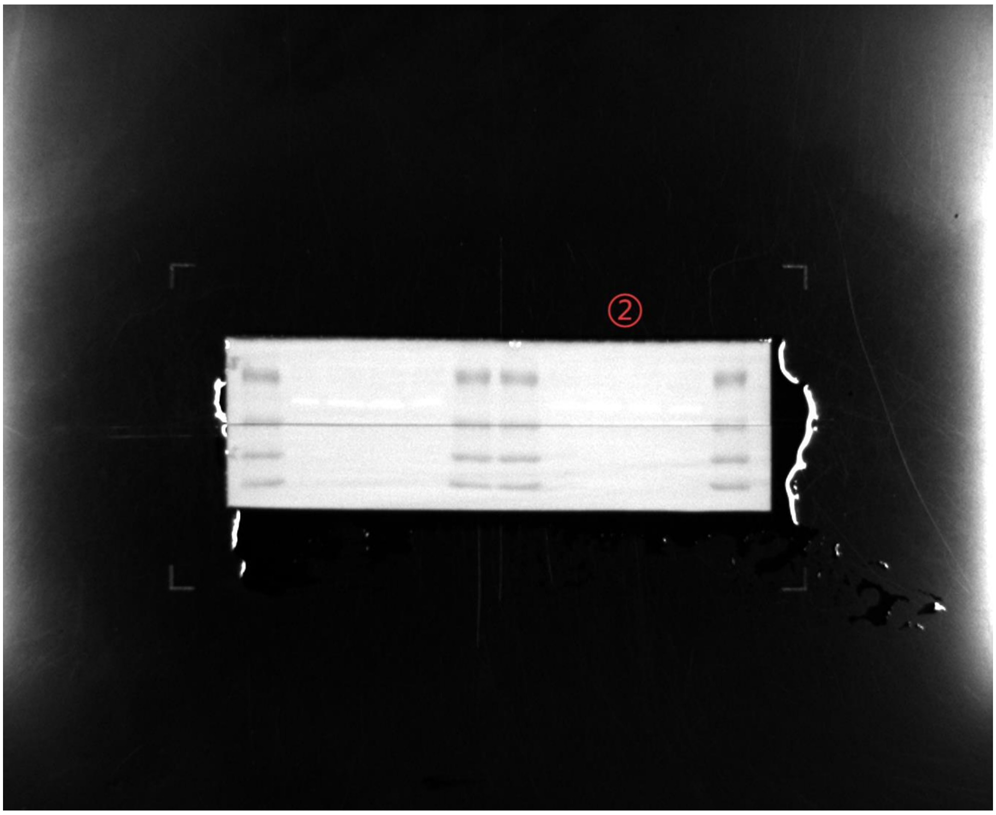

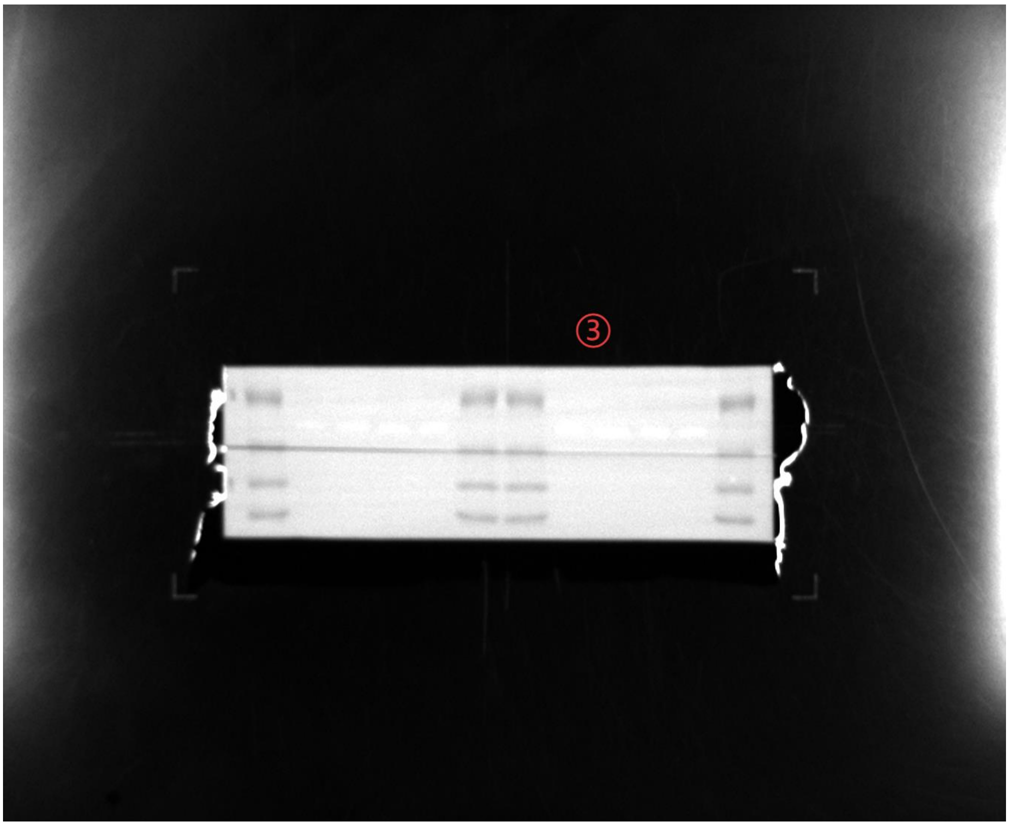
③

Y79(ARL5B)

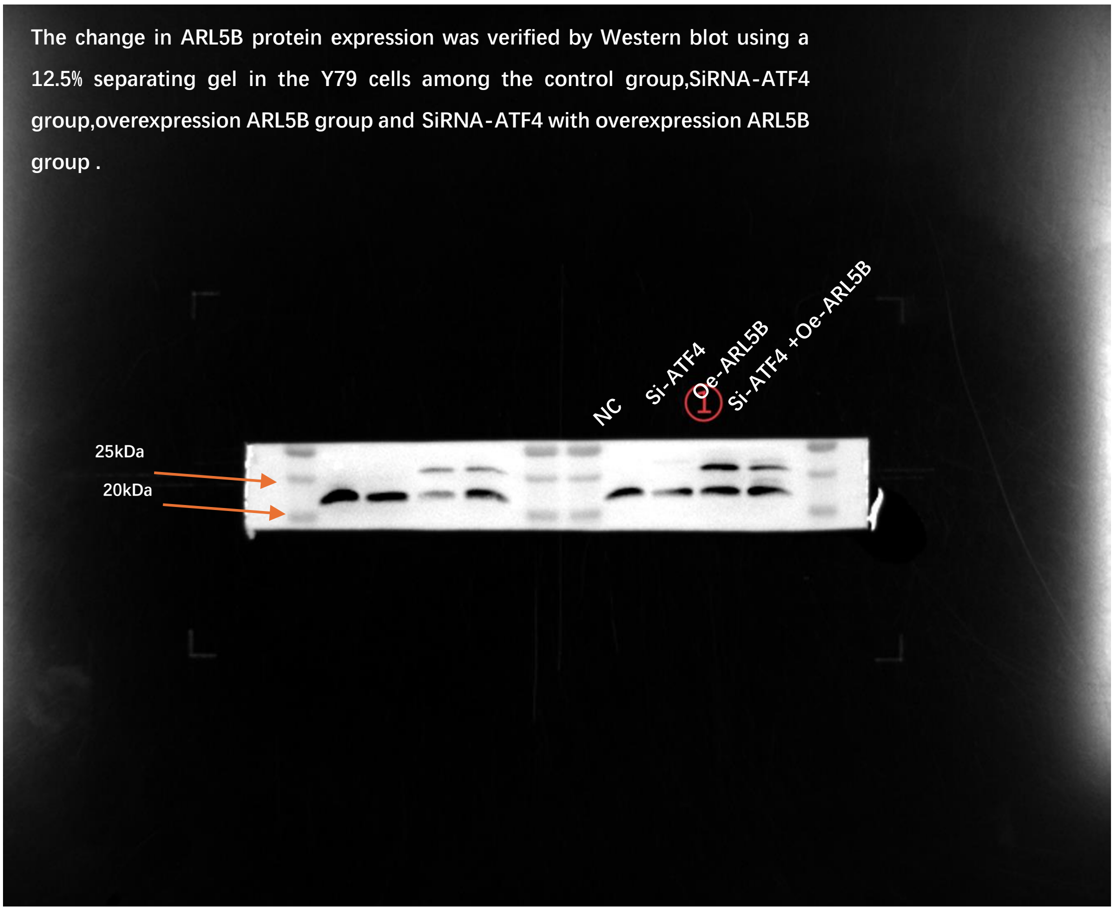
The change in ARL5B protein expression was verified by Western blot using a 12.5% separating gel in the Y79 cells among the control group,SiRNA-ATF4 group,overexpression ARL5B group and SiRNA-ATF4 with overexpression ARL5B group .
NC
Si-ATF4
Oe-ARL5B
Si-ATF4 +Oe-ARL5B
①
25kDa
20kDa

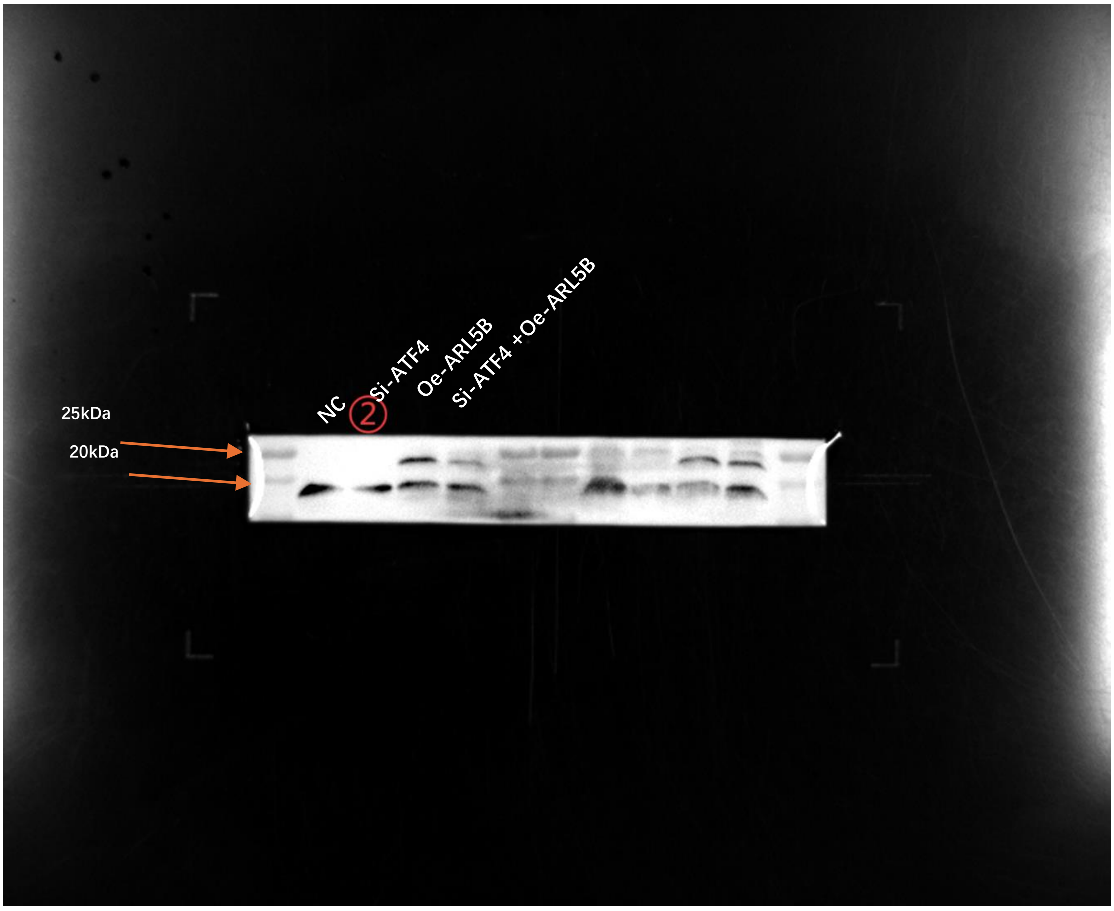
25kDa
20kDa
NC
Si-ATF4
Oe-ARL5B
Si-ATF4 +Oe-ARL5B
2

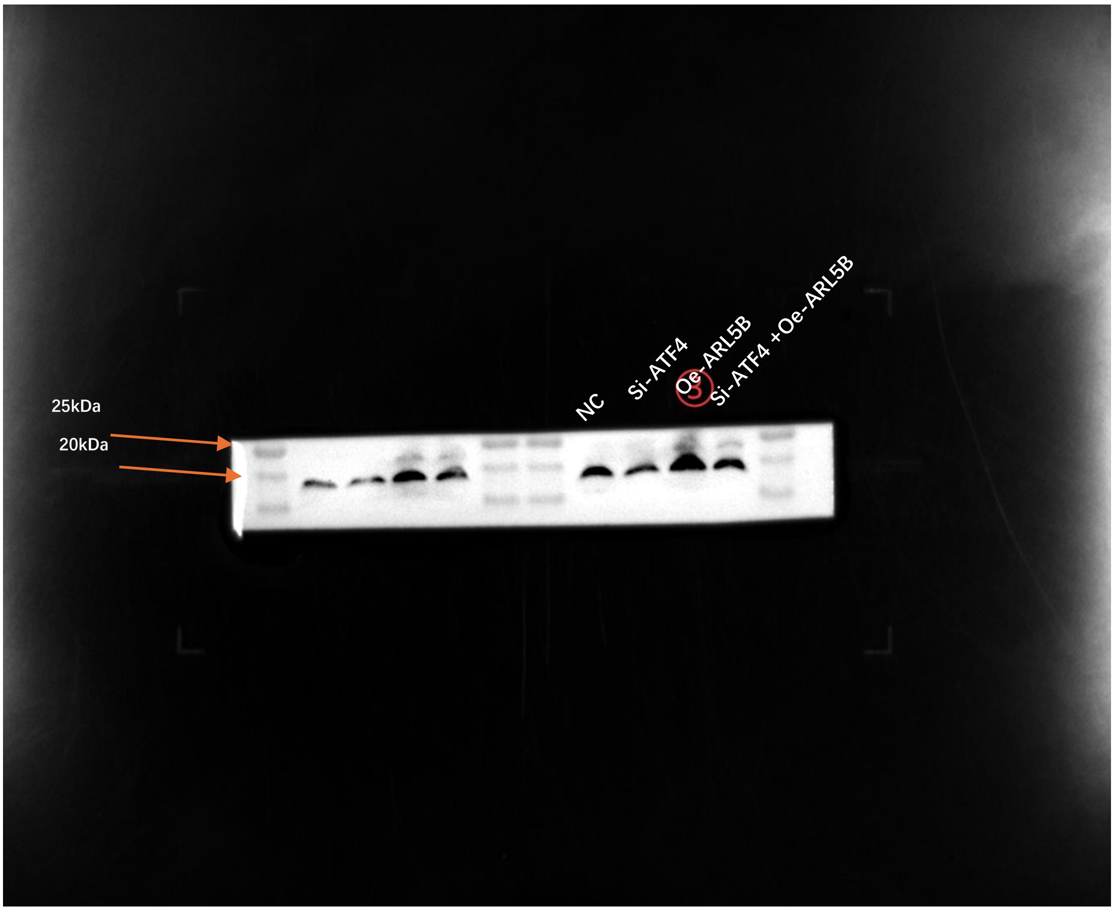
25kDa
20kDa
NC
Si-ATF4
Oe-ARL5B
Si-ATF4 +Oe-ARL5B
3

(β-Actin)

The change in β-Actin protein expression was verified by Western blot using a 12.5% separating gel in the Y79 cells among the control group,SiRNA-ATF4 group,overexpression ARL5B group and SiRNA-ATF4 with overexpression ARL5B group .

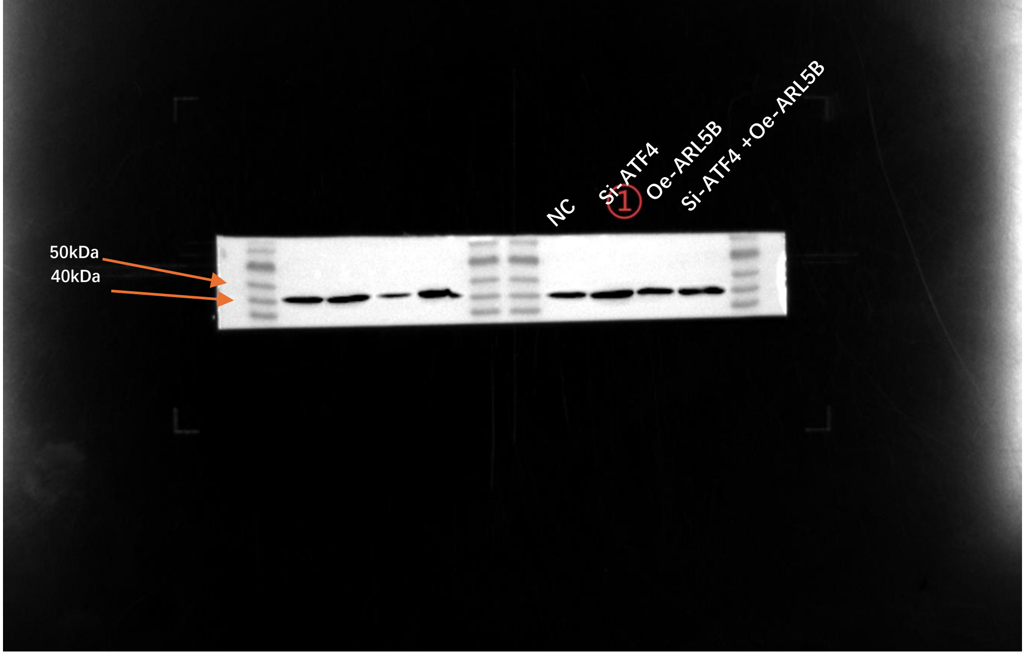

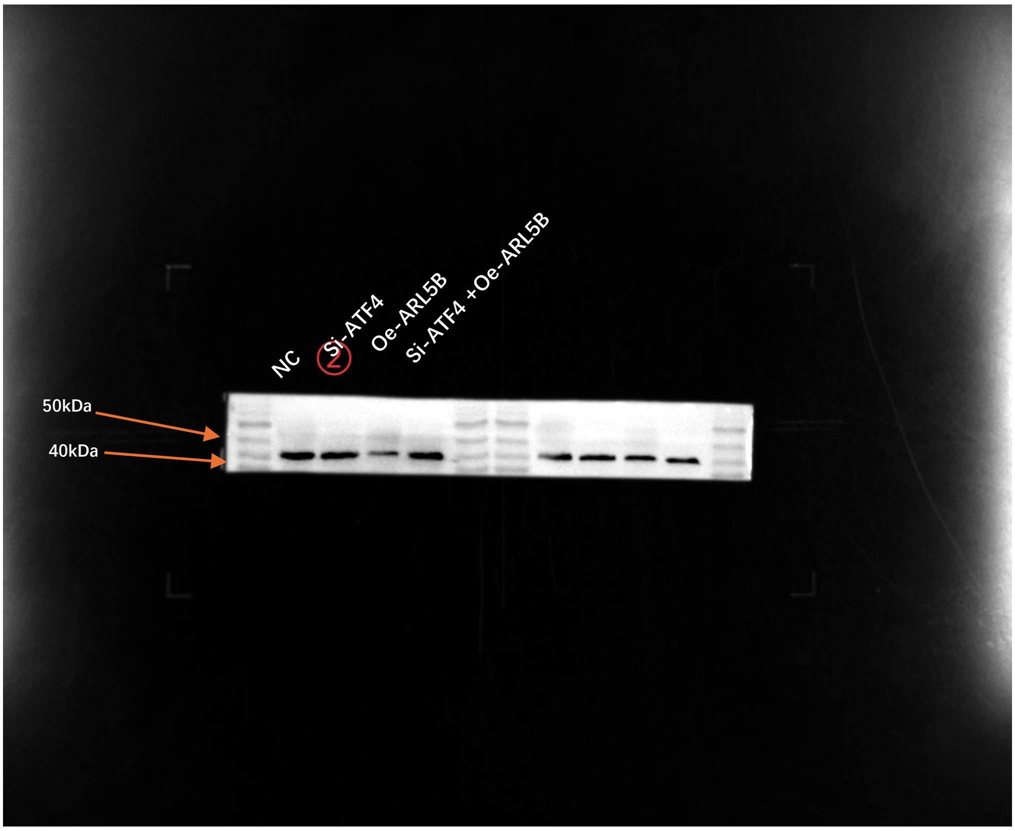

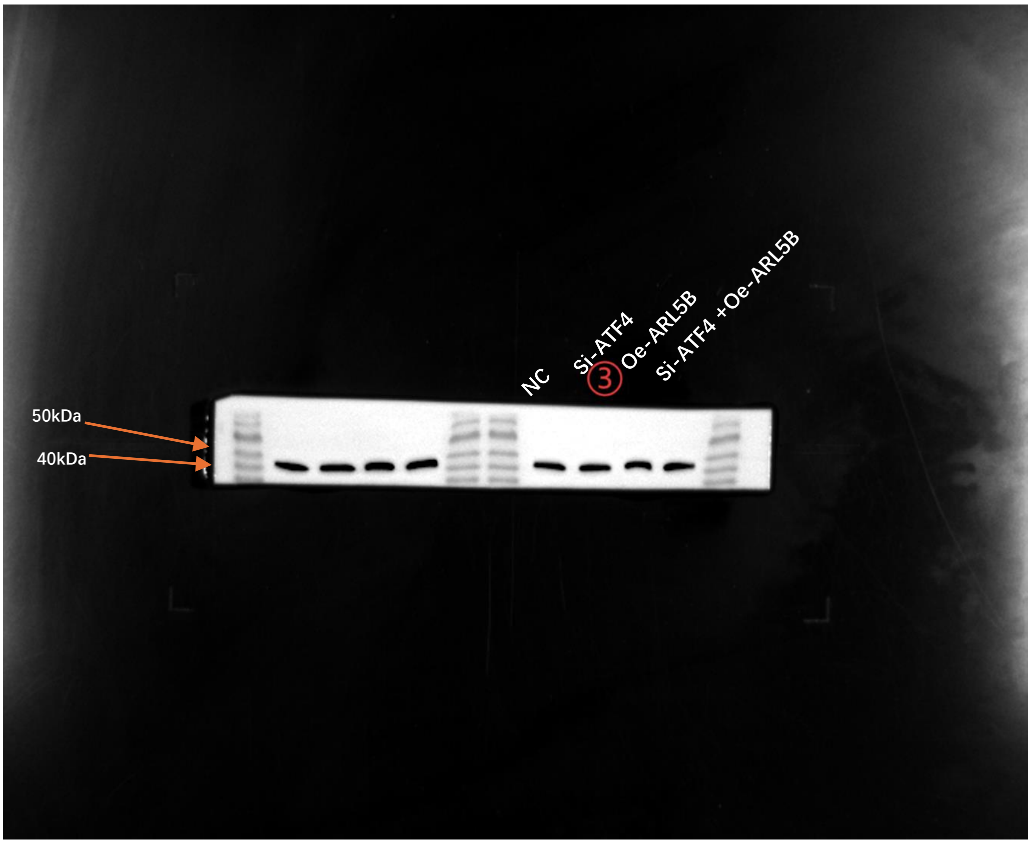

(MERGE)

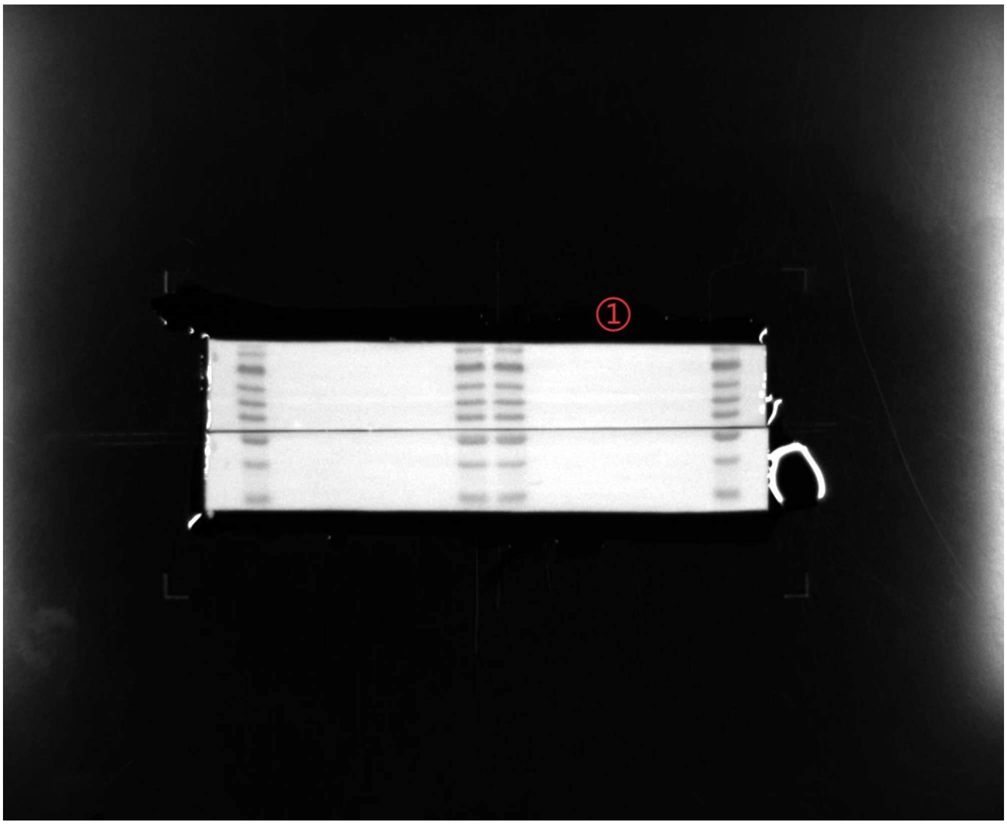

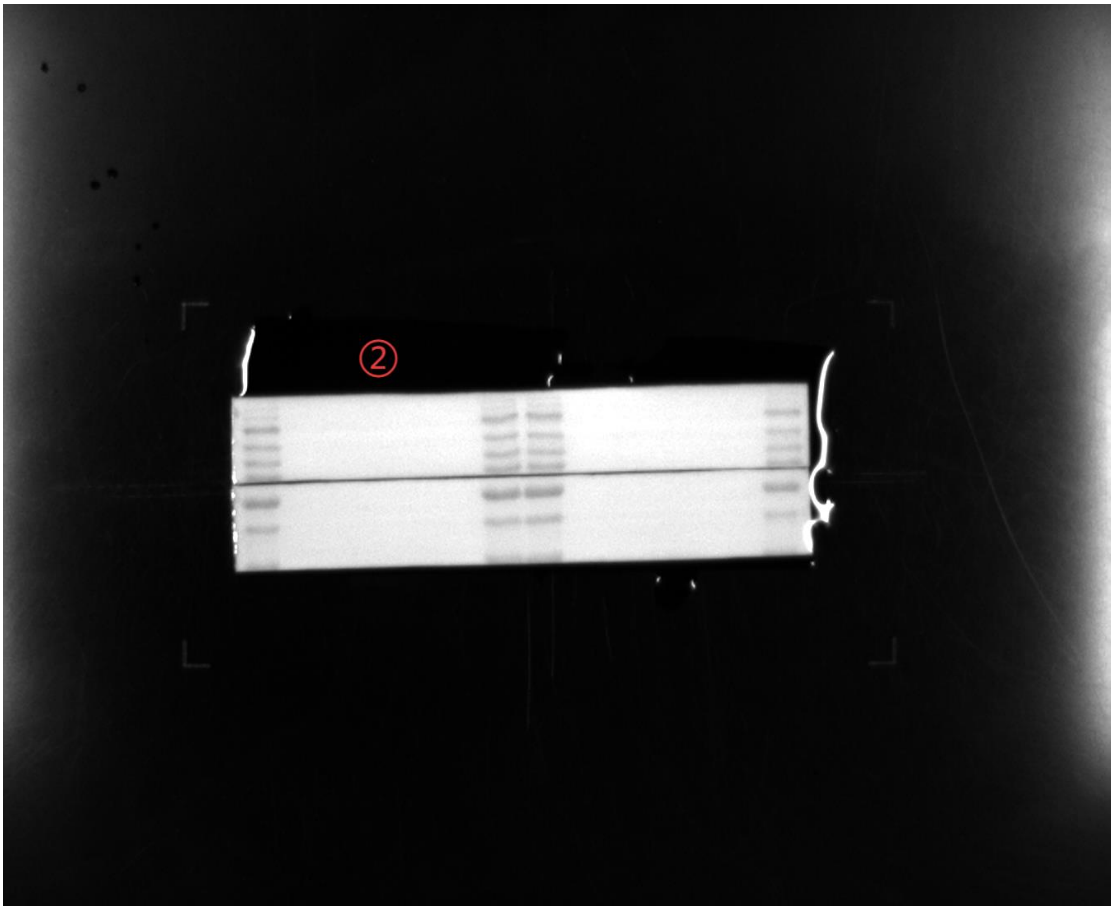
②

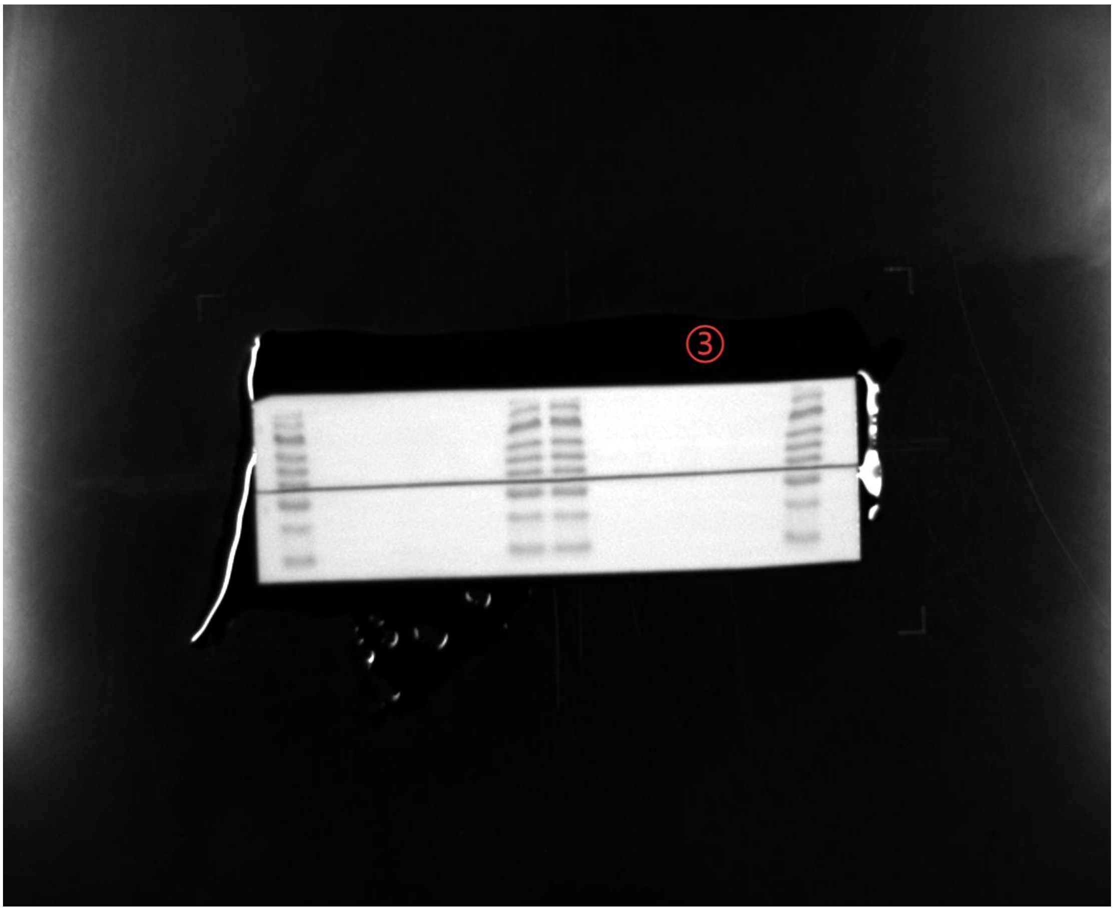
③

Y79(SKIP)

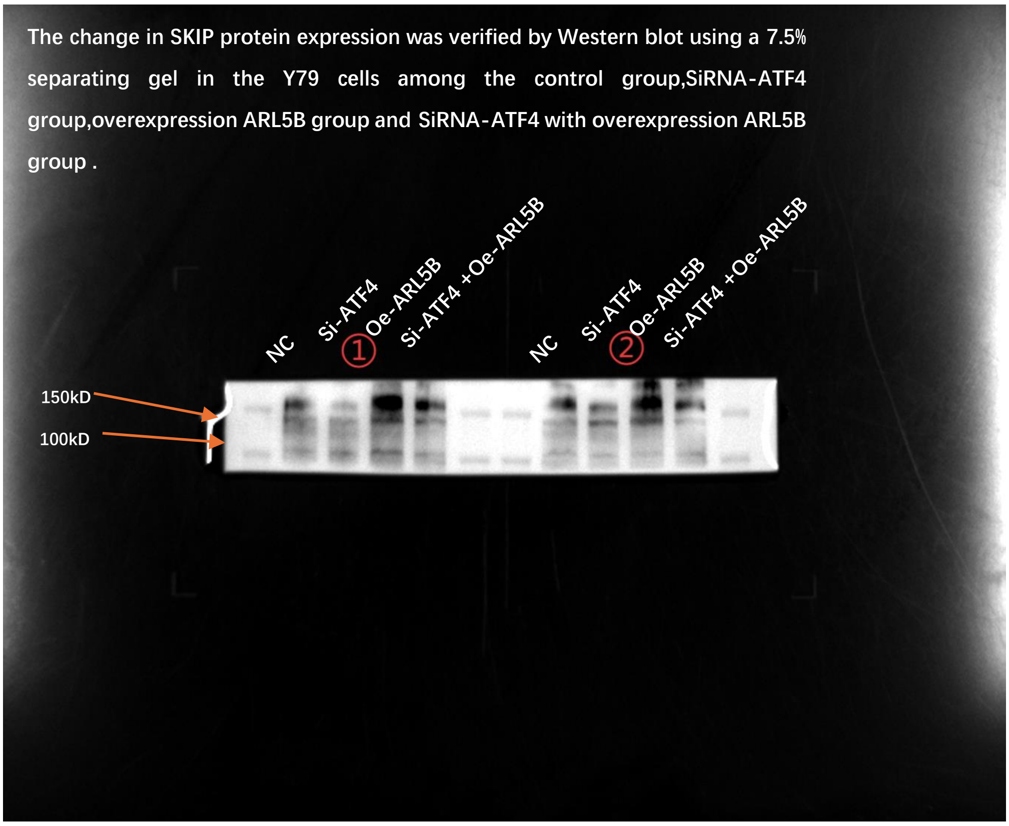
The change in SKIP protein expression was verified by Western blot using a 7.5% separating gel in the Y79 cells among the control group,SiRNA-ATF4 group,overexpression ARL5B group and SiRNA-ATF4 with overexpression ARL5B group .
NC
Si-ATF4
Oe-ARL5B
Si-ATF4 +Oe-ARL5B
①
NC
Si-ATF4
Oe-ARL5B
Si-ATF4 +Oe-ARL5B
②
150kD
100kD

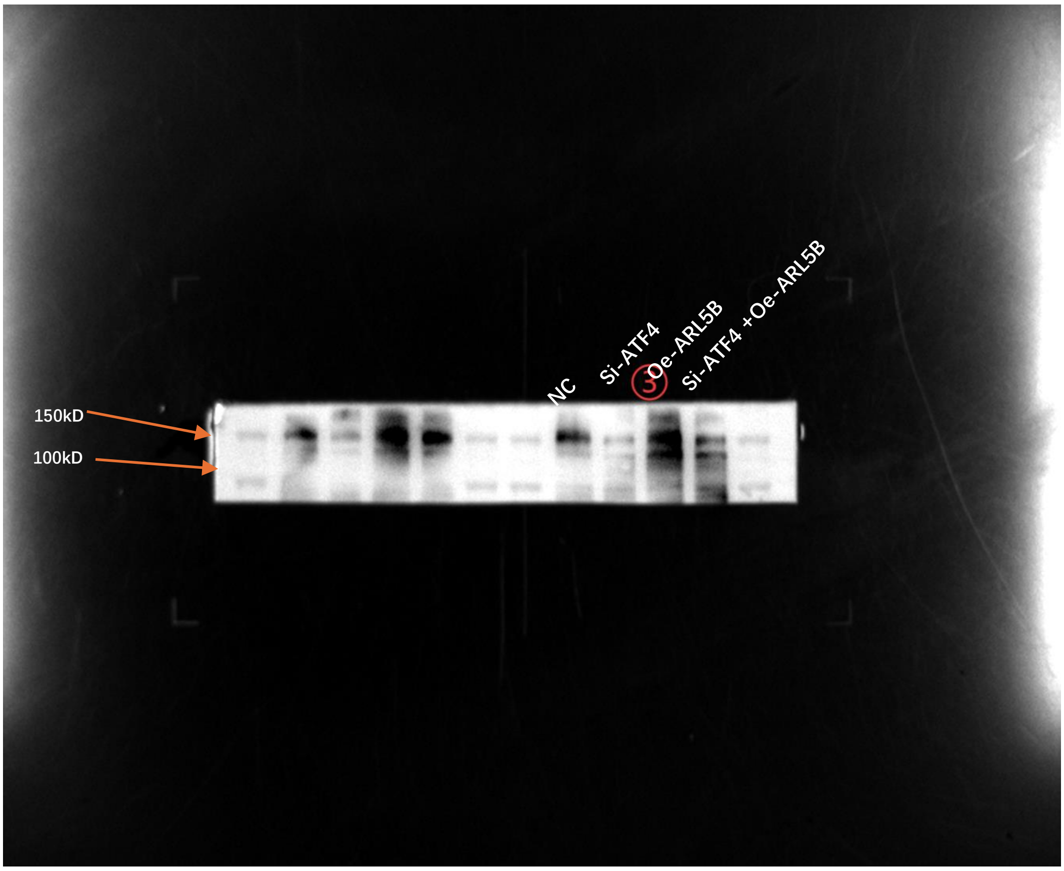
NC
Si-ATF4
Oe-ARL5B
Si-ATF4 +Oe-ARL5B
③
150kD
100kD

(β-Actin)

The change in β-Actin protein expression was verified by Western blot using a 7.5% separating gel in the Y79 cells among the control group,SiRNA-ATF4 group,overexpression ARL5B group and SiRNA-ATF4 with overexpression ARL5B group .

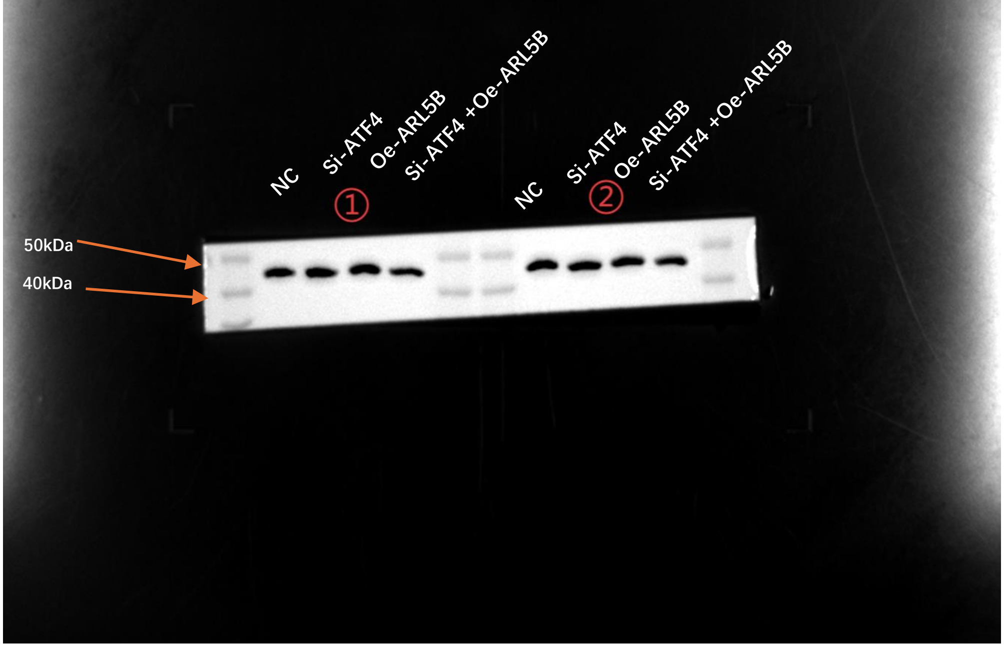

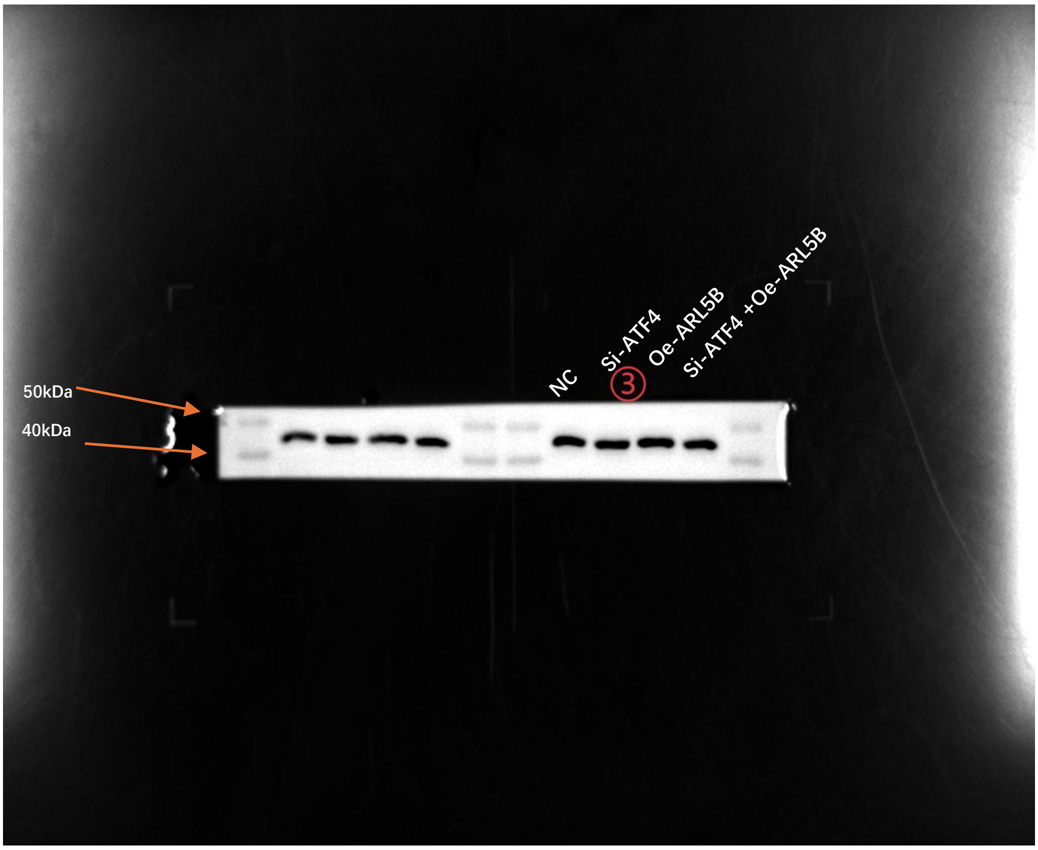

(MERGE)

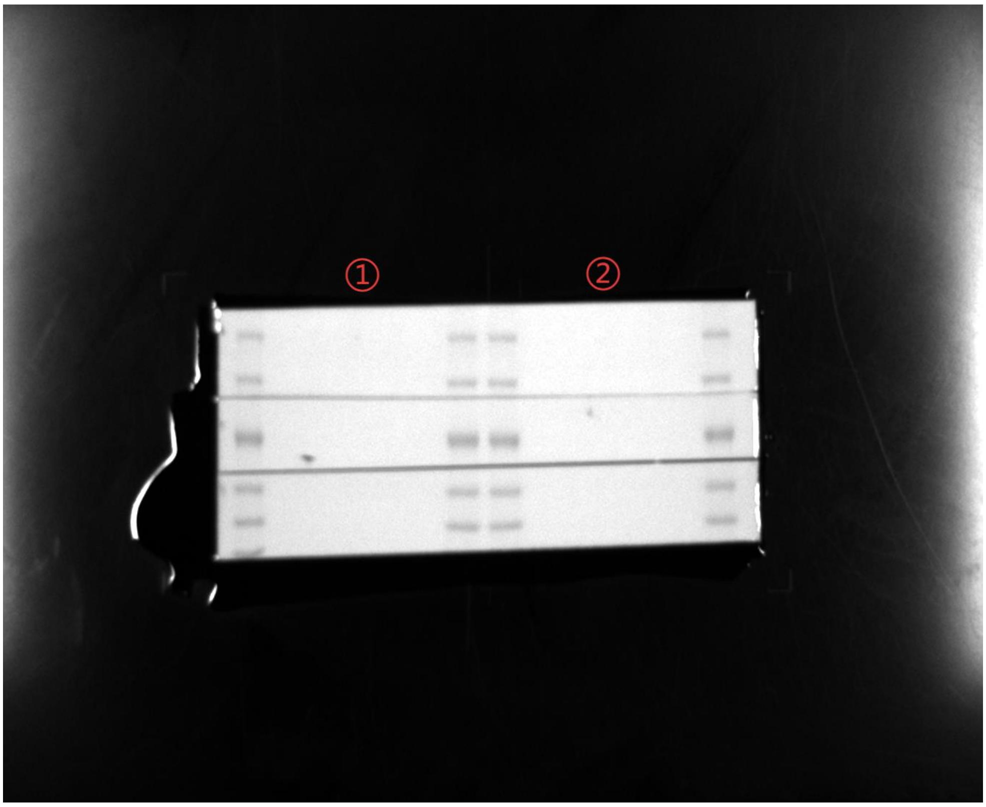

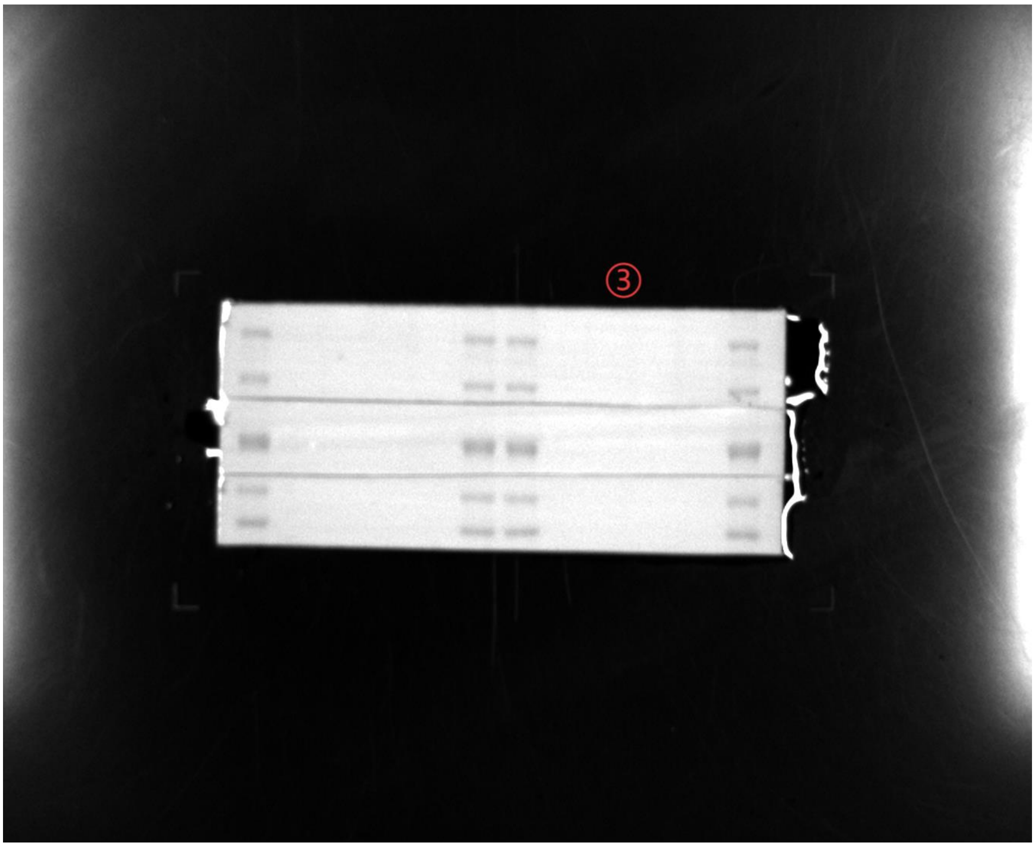

Y79(KIF5B)

The change in KIF5B protein expression was verified by Western blot using a 7.5% separating gel in the Y79 cells among the control group,SiRNA-ATF4 group,overexpression ARL5B group and SiRNA-ATF4 with overexpression ARL5B group .

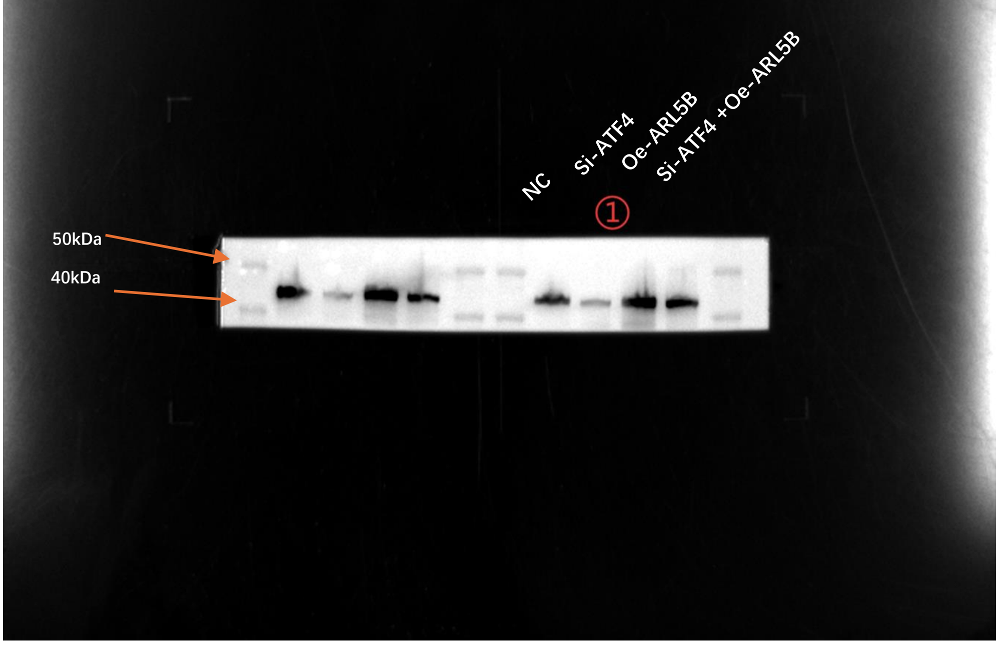

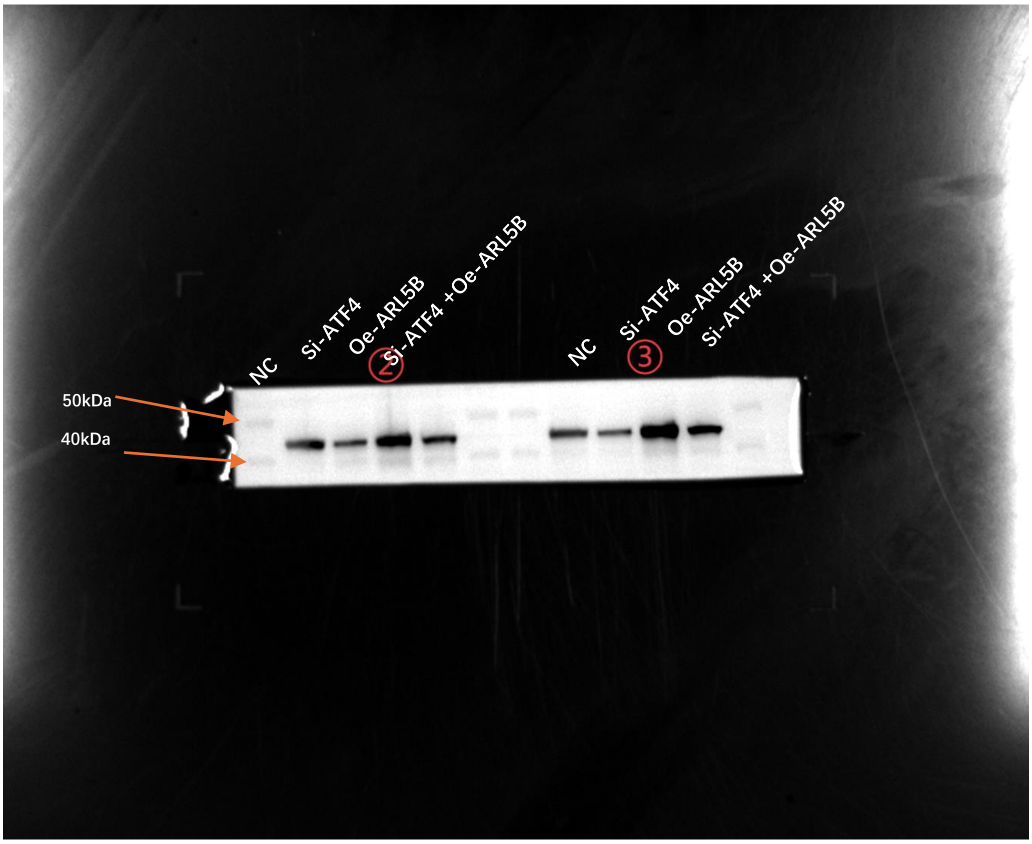

(β-Actin)

The change in β-Actin protein expression was verified by Western blot using a 7.5% separating gel in the Y79 cells among the control group,SiRNA-ATF4 group,overexpression ARL5B group and SiRNA-ATF4 with overexpression ARL5B group .

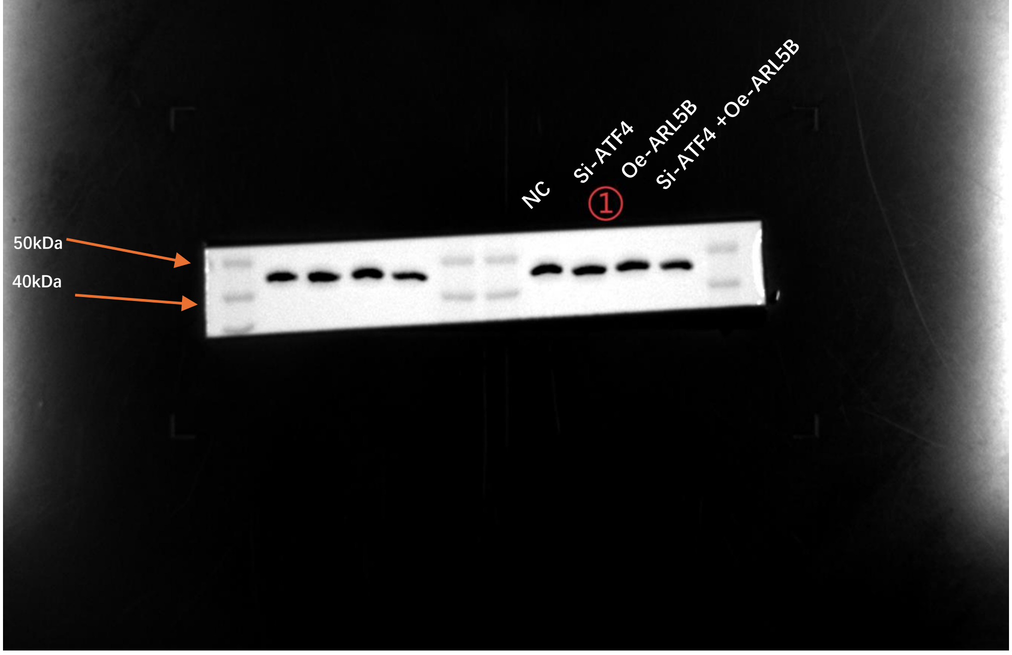

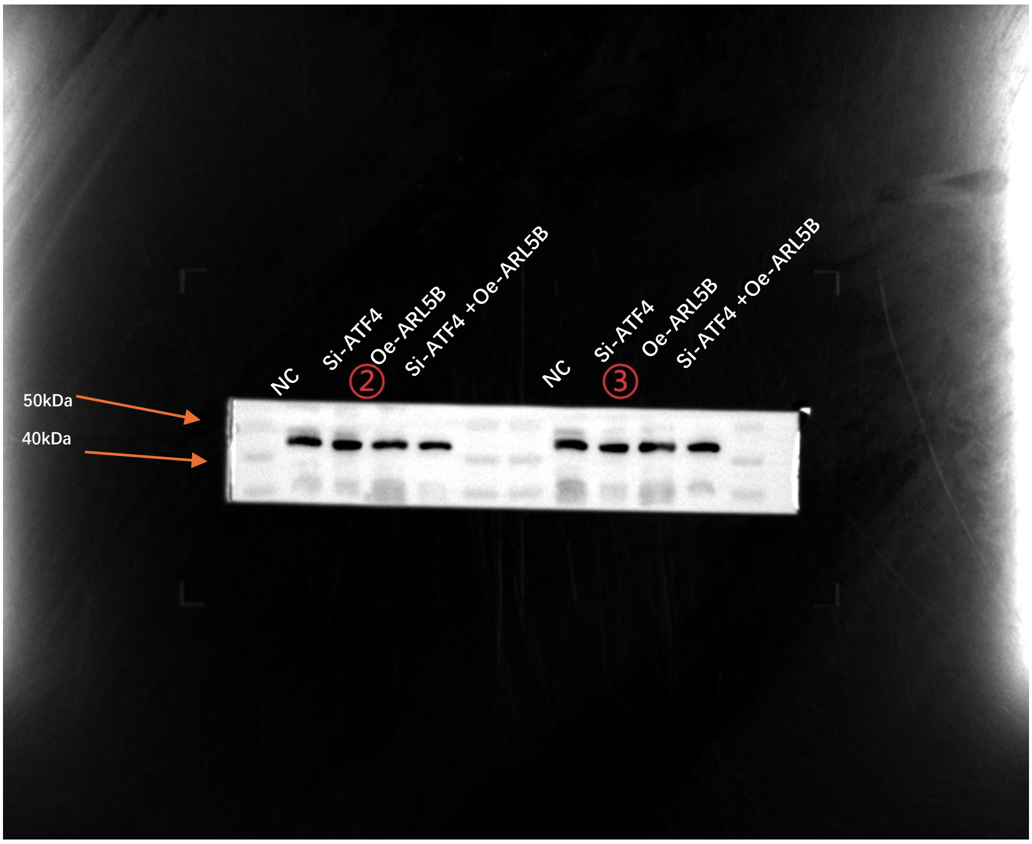

(MERGE)

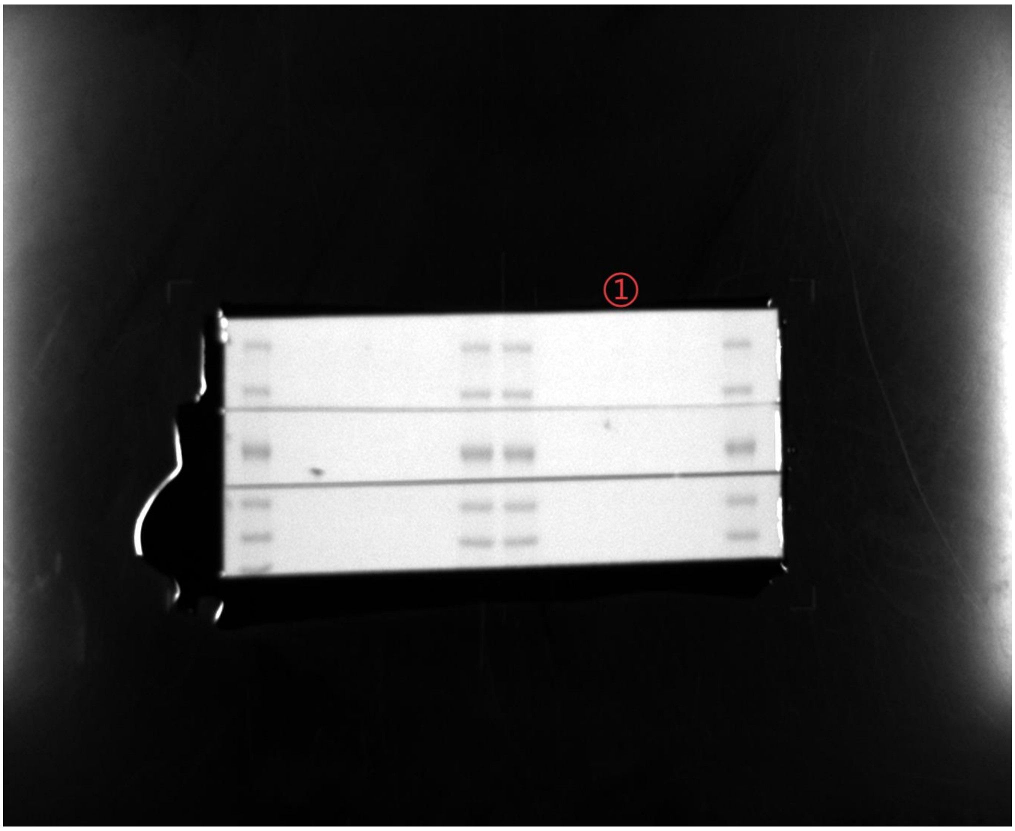

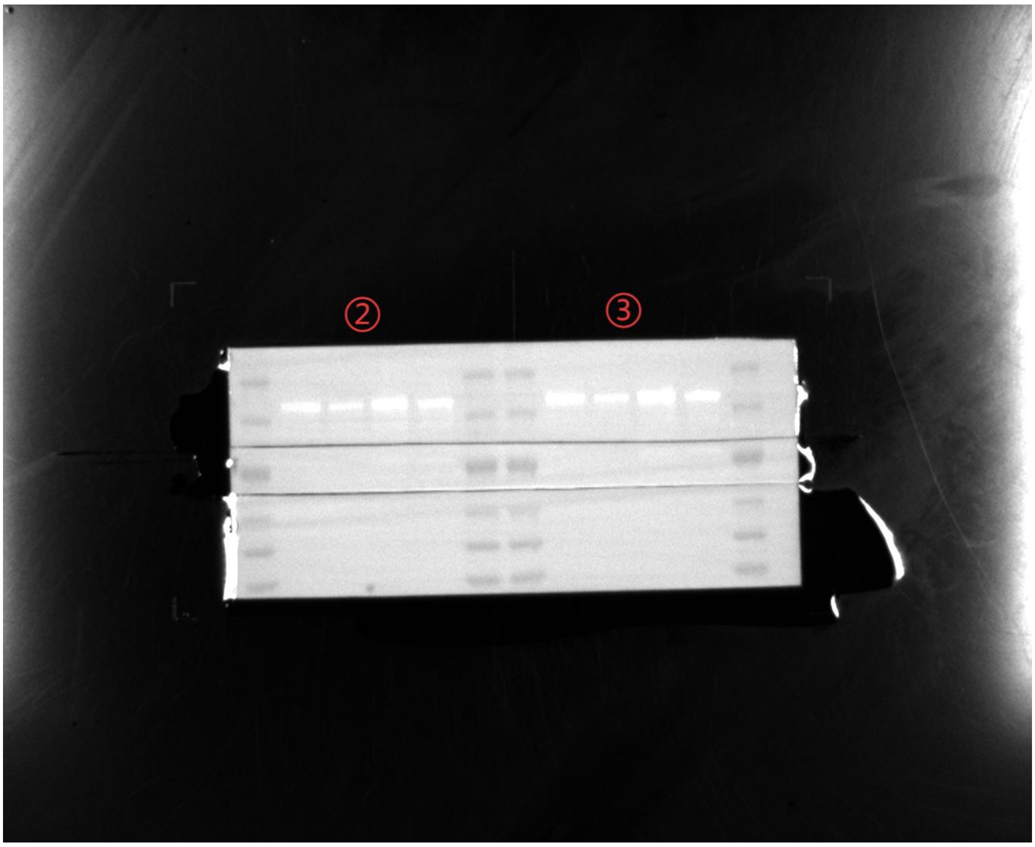

Y79(KLC2)

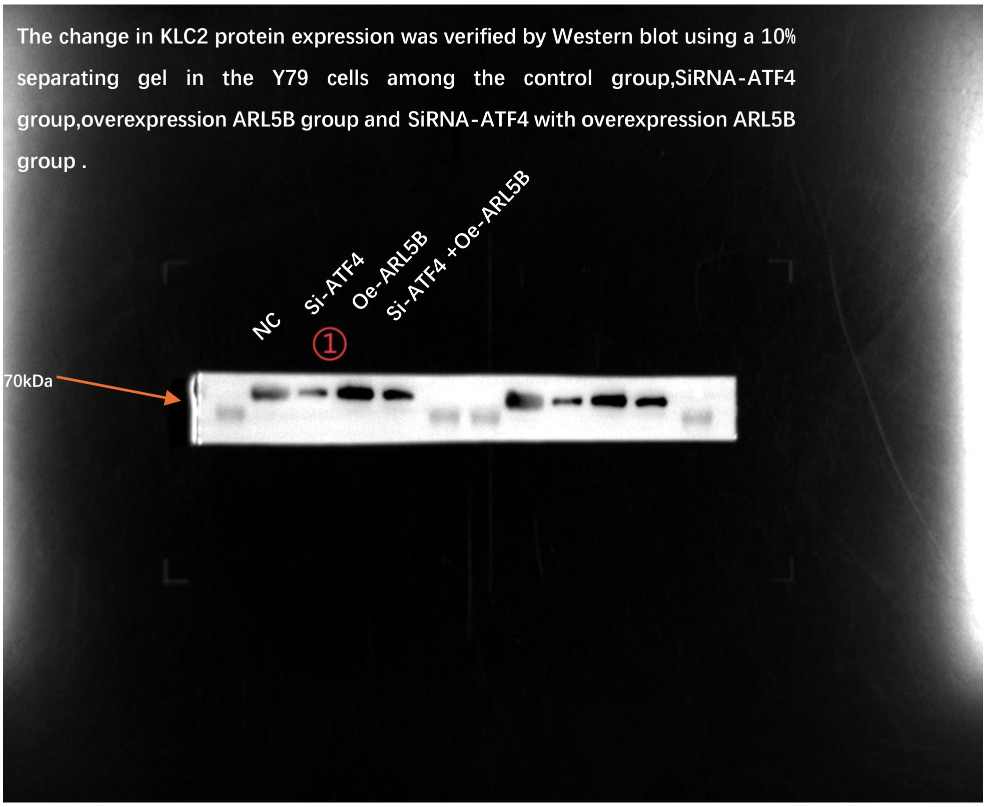

The change in KLC2 protein expression was verified by Western blot using a 10% separating gel in the Y79 cells among the control group,SiRNA-ATF4 group,overexpression ARL5B group and SiRNA-ATF4 with overexpression ARL5B group .

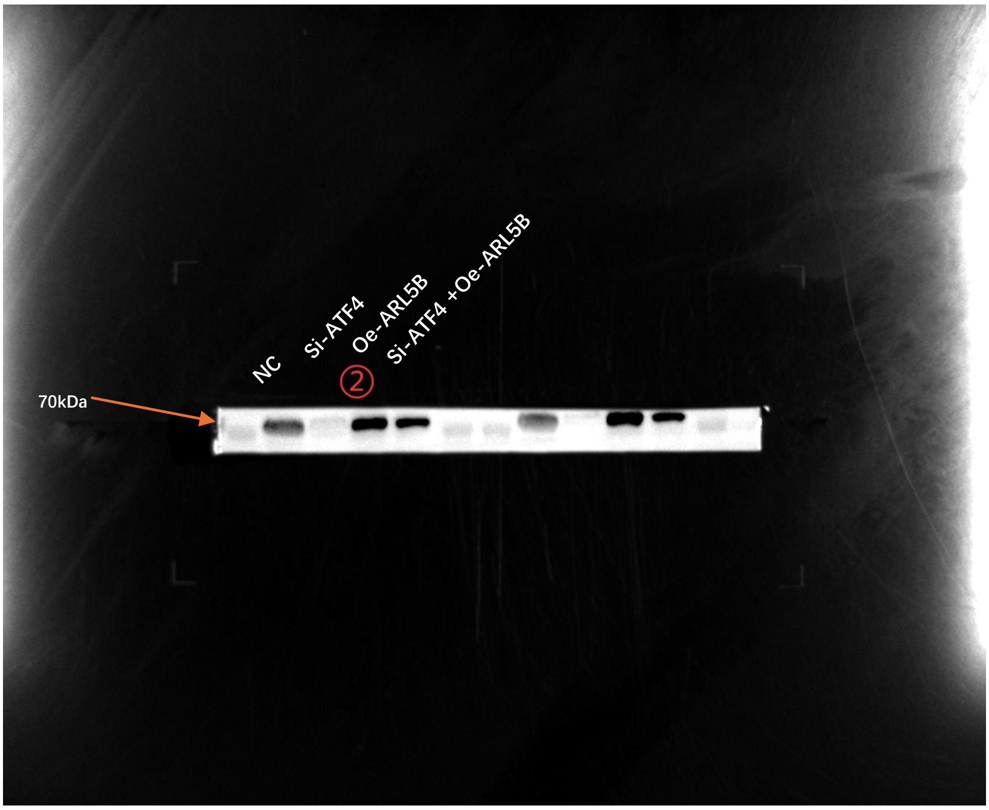

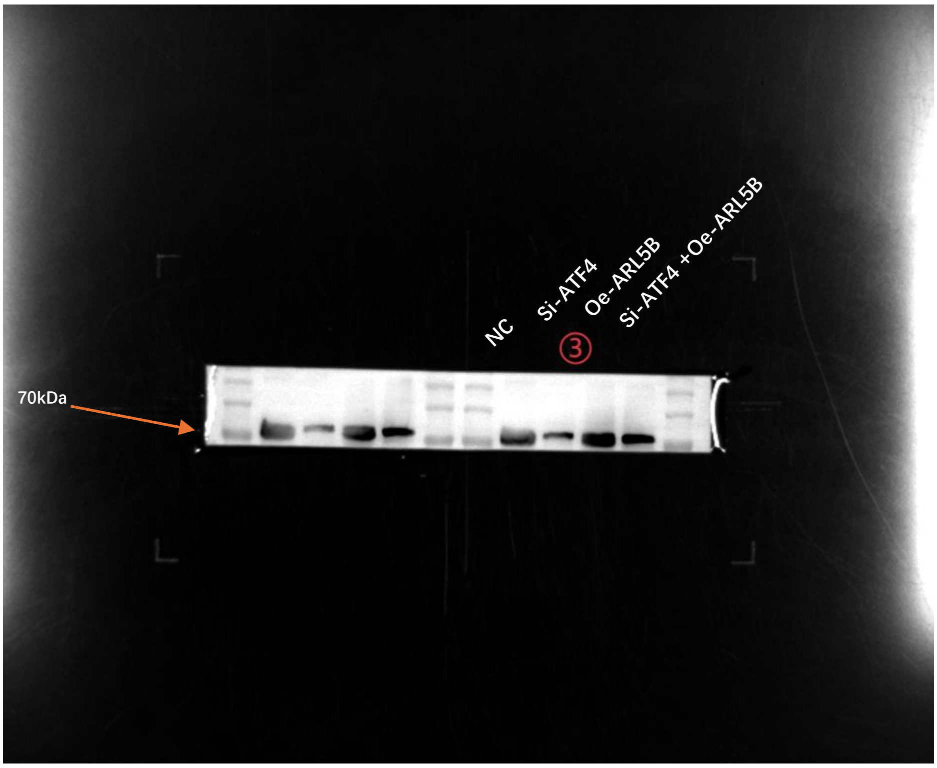
NC
Si-ATF4
Oe-ARL5B
Si-ATF4 +Oe-ARL5B
③
70kDa

(β-Actin)

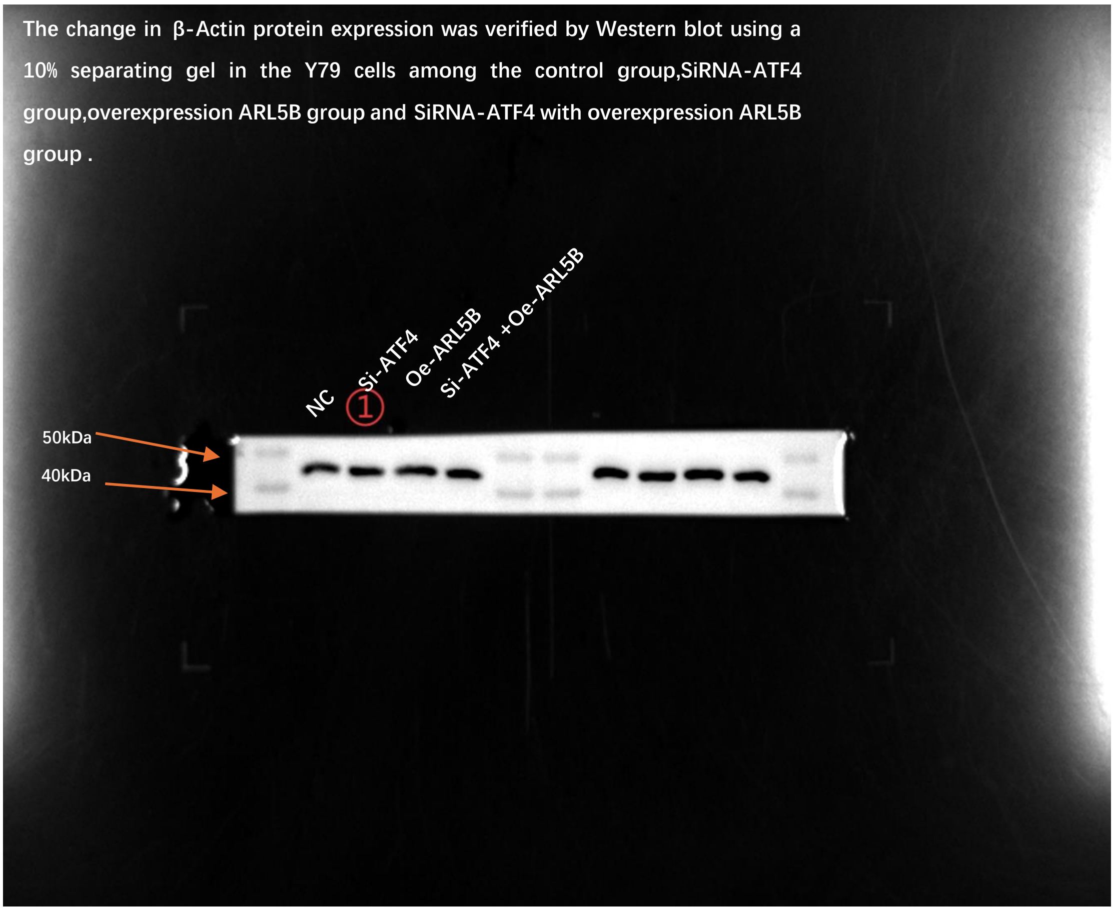
The change in β-Actin protein expression was verified by Western blot using a 10% separating gel in the Y79 cells among the control group,SiRNA-ATF4 group,overexpression ARL5B group and SiRNA-ATF4 with overexpression ARL5B group .
NC
Si-ATF4
Oe-ARL5B
Si-ATF4 +Oe-ARL5B
①
50kDa
40kDa

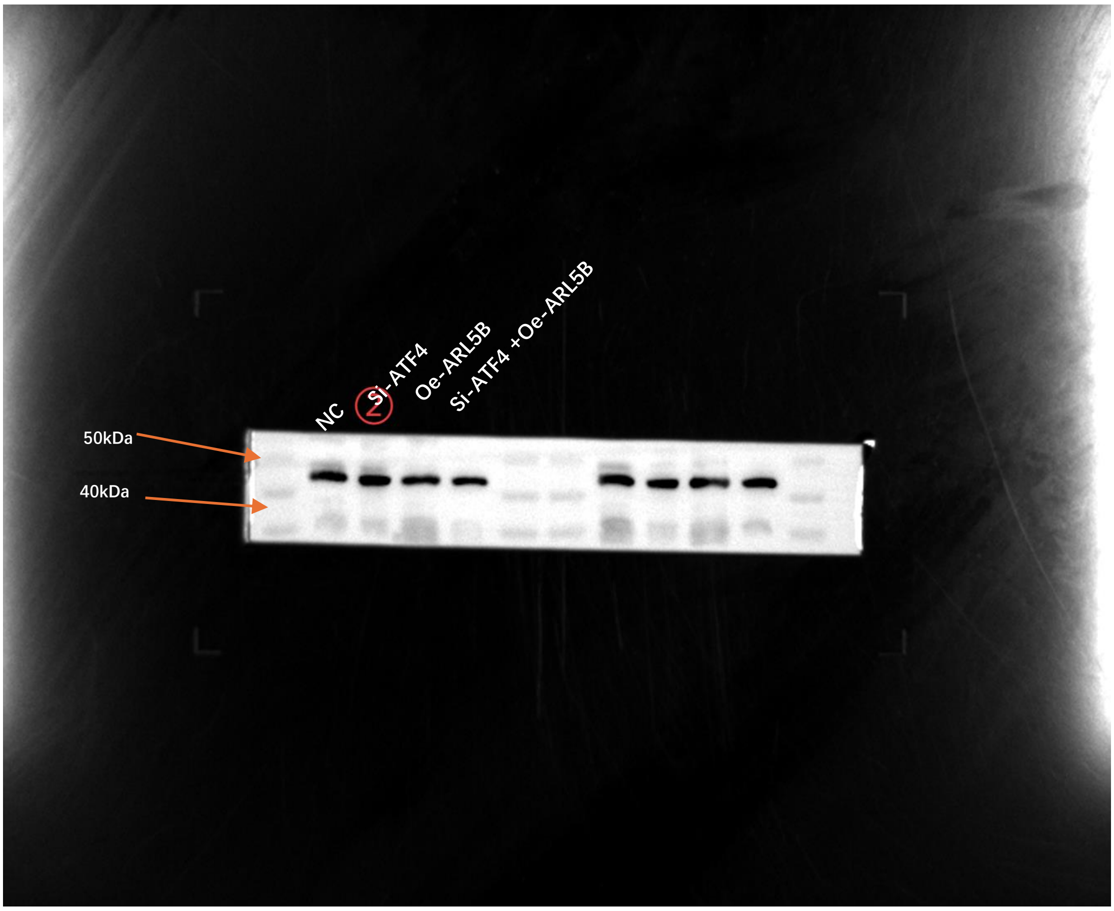
NC
Si-ATF4
②
Oe-ARL5B
Si-ATF4 +Oe-ARL5B
50kDa
40kDa
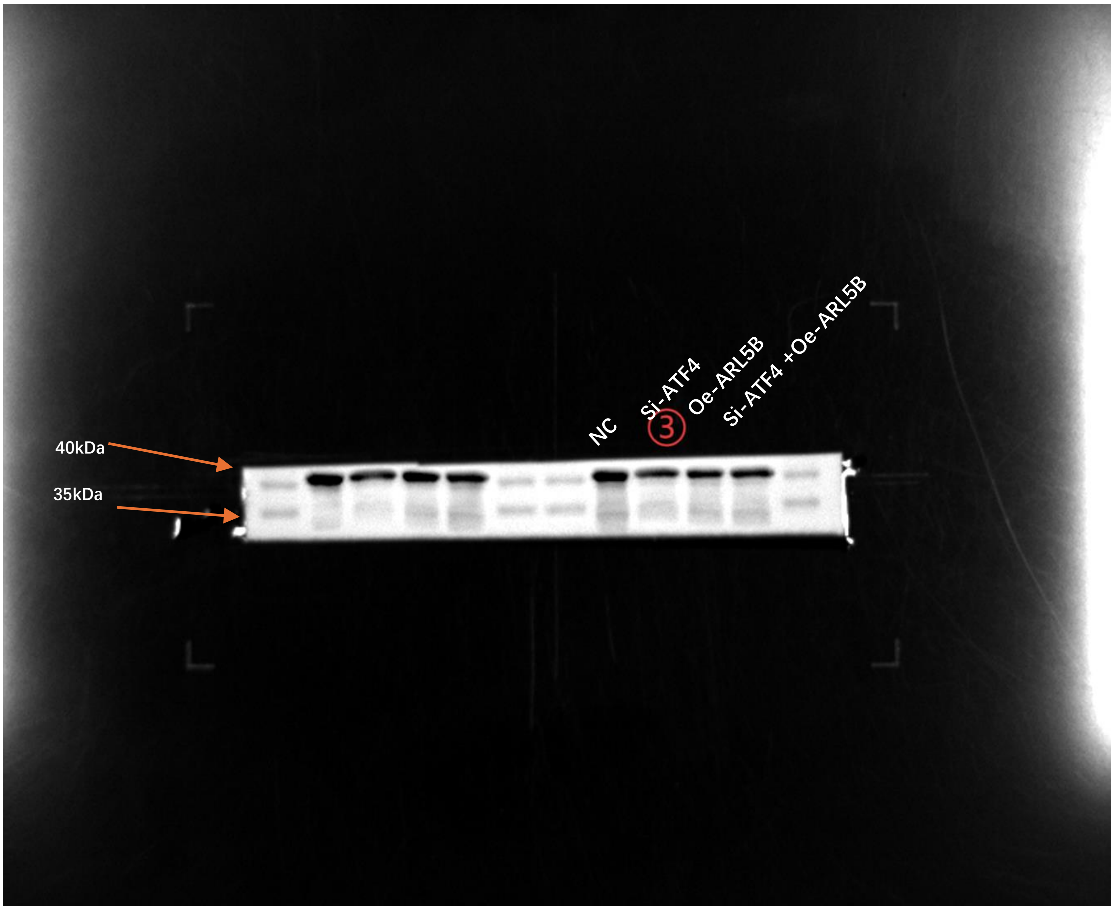
NC
Si-ATF4
③
Oe-ARL5B
Si-ATF4 +Oe-ARL5B
40kDa
35kDa

(MERGE)

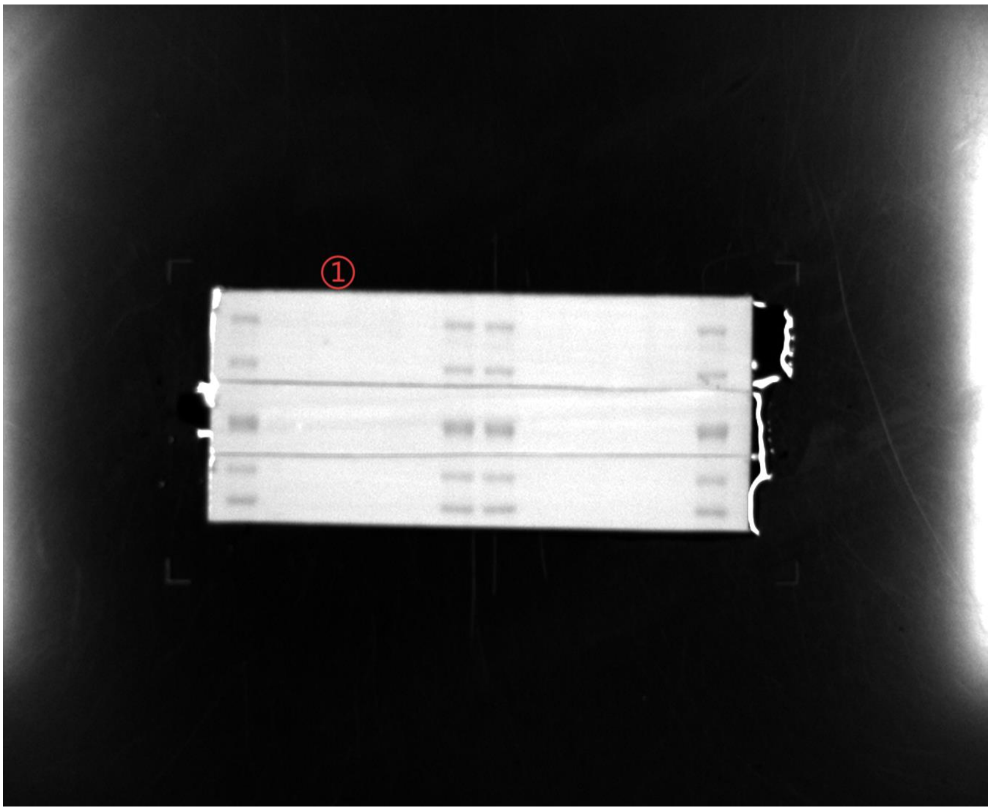

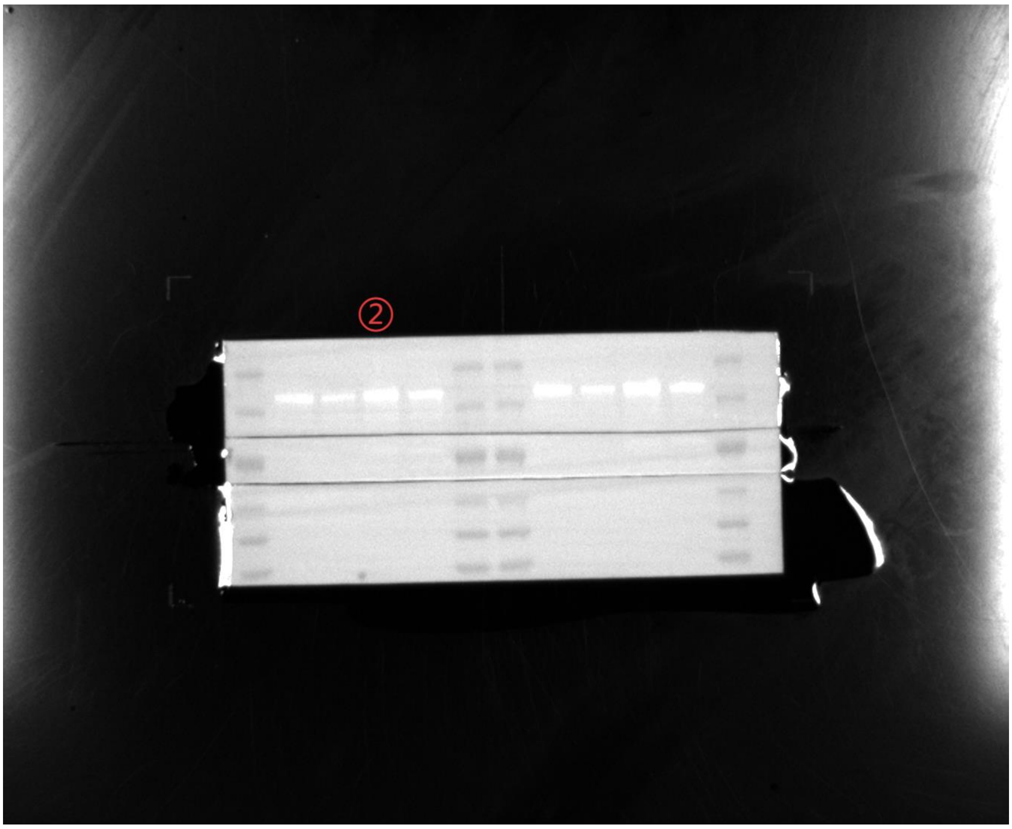

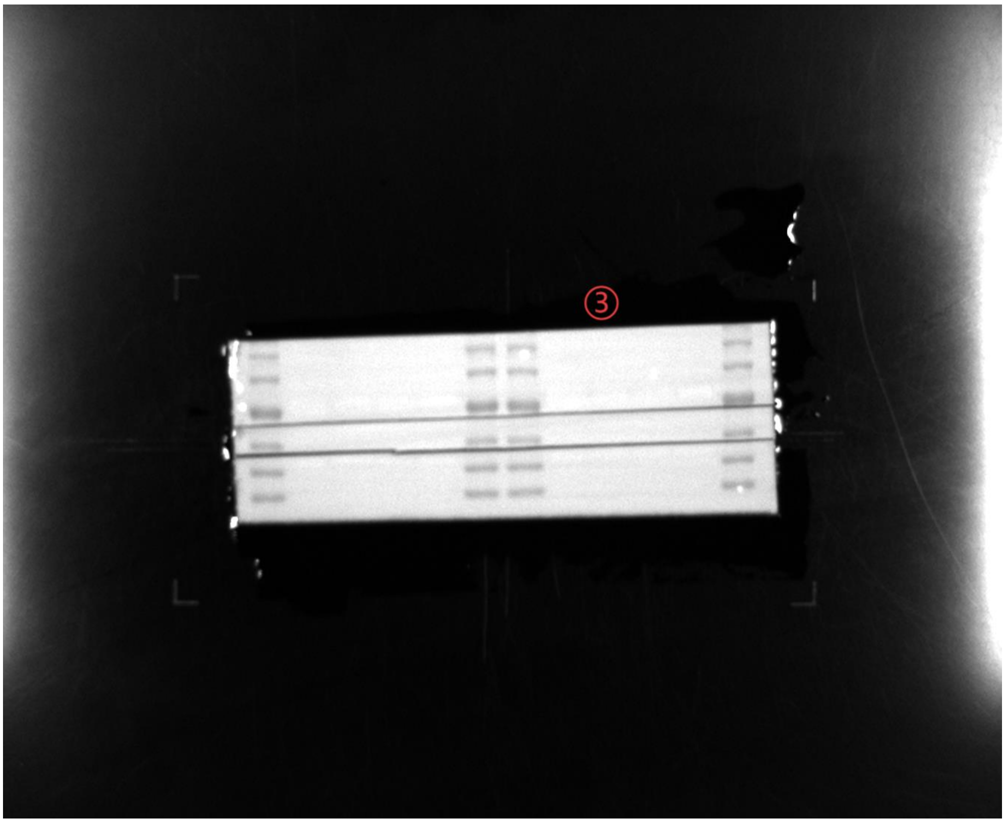

Weri-RB1(ATF4)

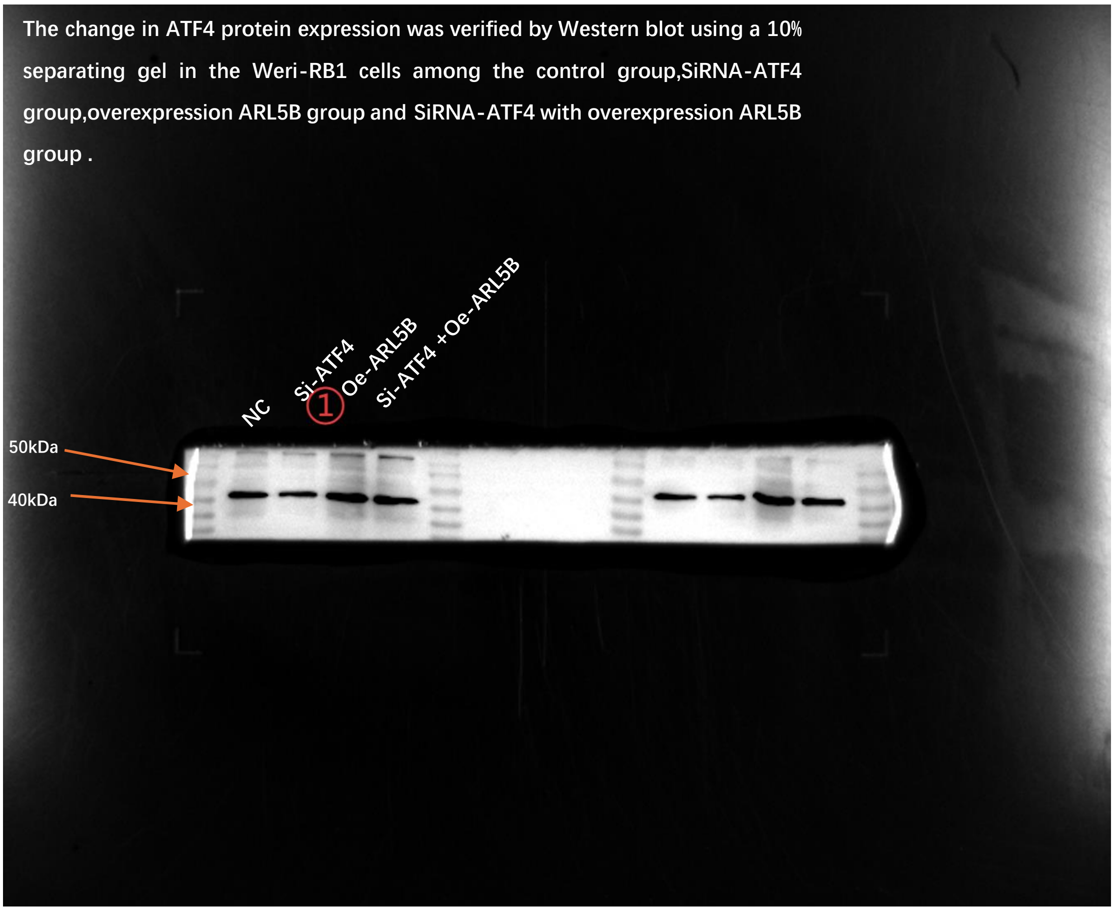

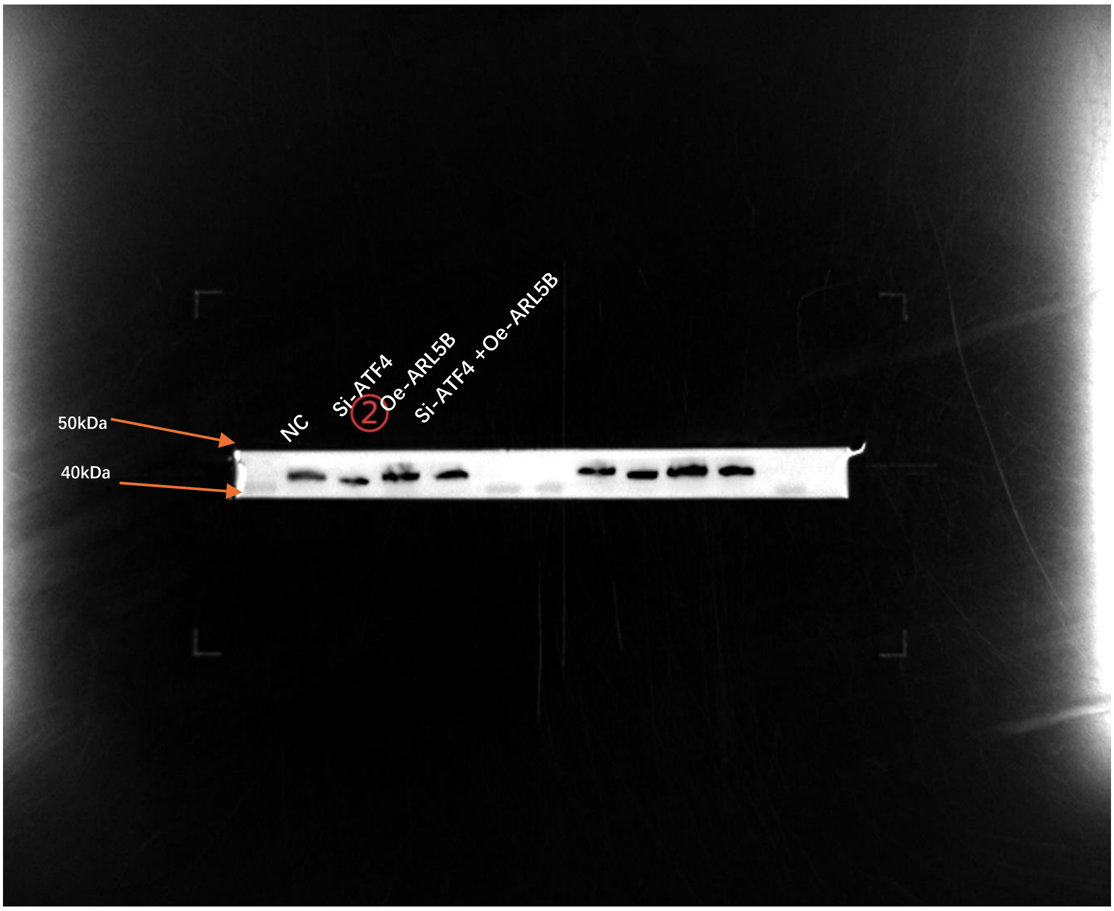
50kDa
40kDa
NC
Si-ATF4
②
Oe-ARL5B
Si-ATF4 +Oe-ARL5B

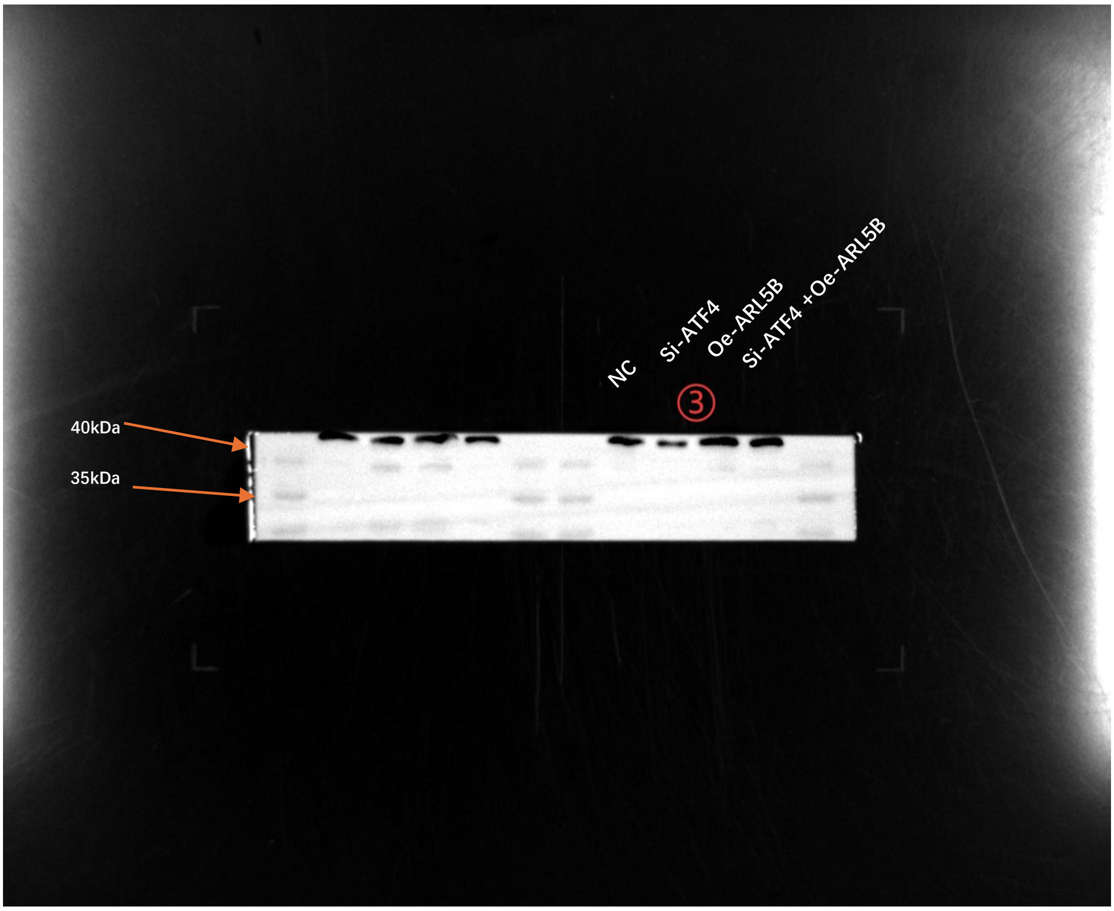
40kDa
35kDa
NC
Si-ATF4
Oe-ARL5B
Si-ATF4 +Oe-ARL5B
③

(β-Actin)

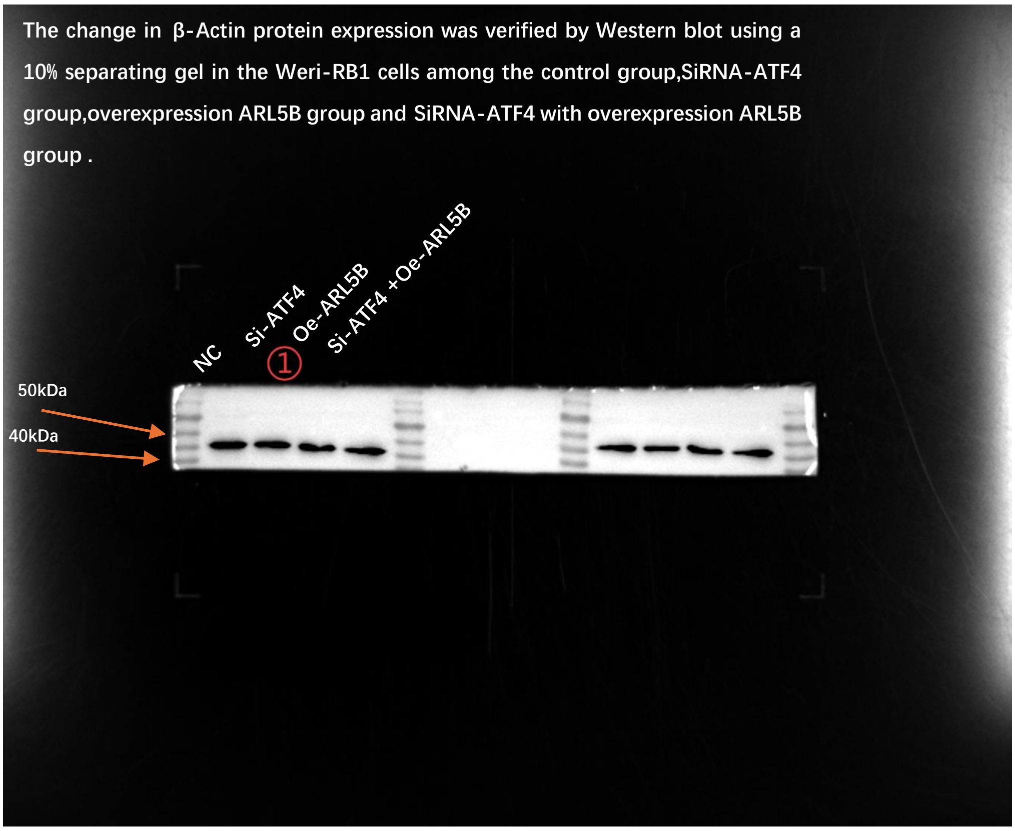
The change in β-Actin protein expression was verified by Western blot using a 10% separating gel in the Weri-RB1 cells among the control group,SiRNA-ATF4 group,overexpression ARL5B group and SiRNA-ATF4 with overexpression ARL5B group .
NC
Si-ATF4
Oe-ARL5B
Si-ATF4 +Oe-ARL5B
①
50kDa
40kDa

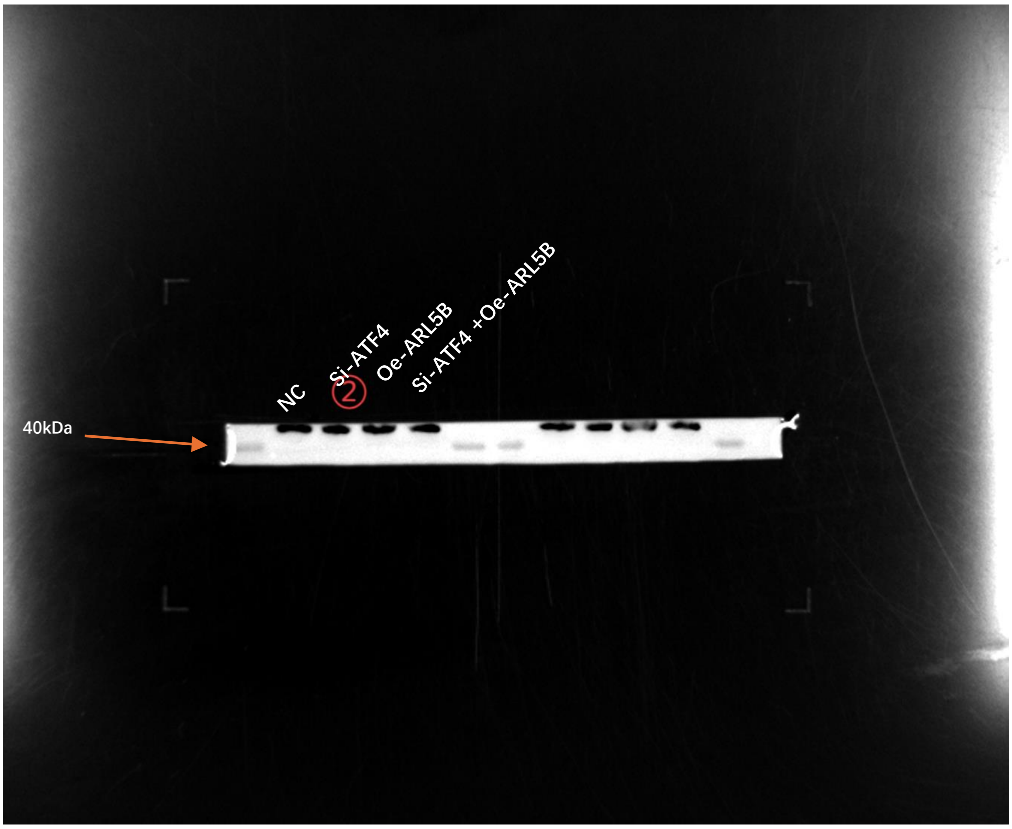
NC
Si-ATF4
Oe-ARL5B
Si-ATF4 +Oe-ARL5B
②
40kDa

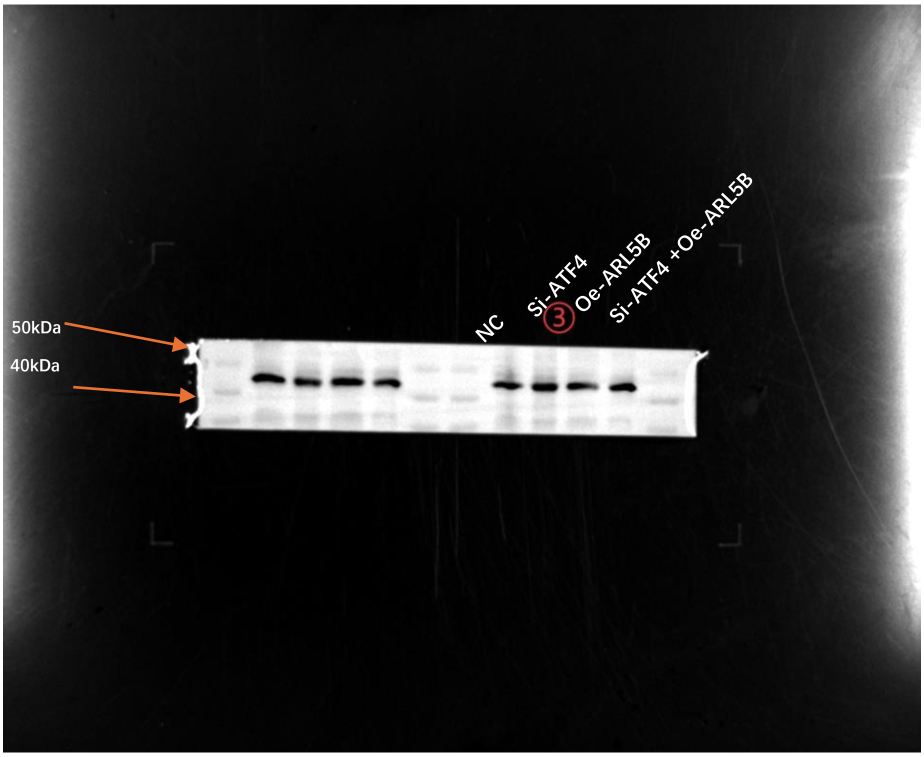

(MERGE)

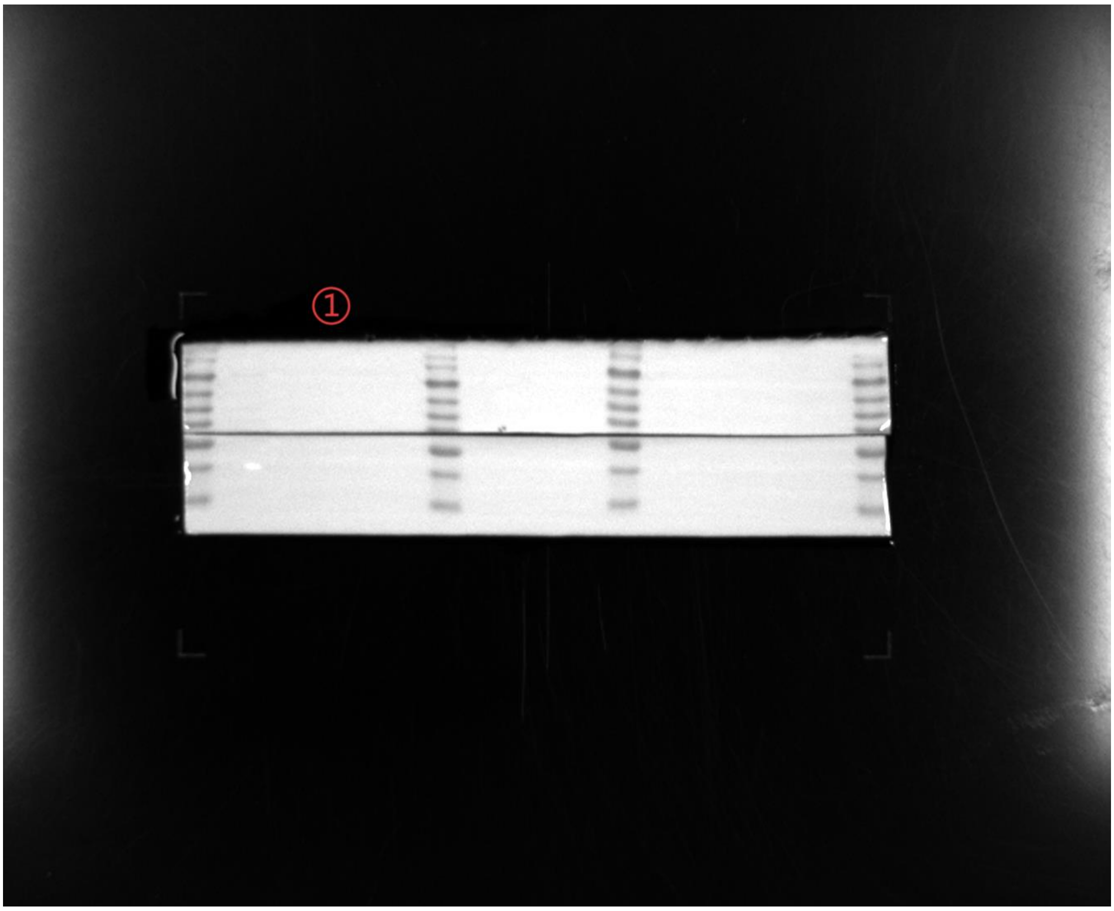

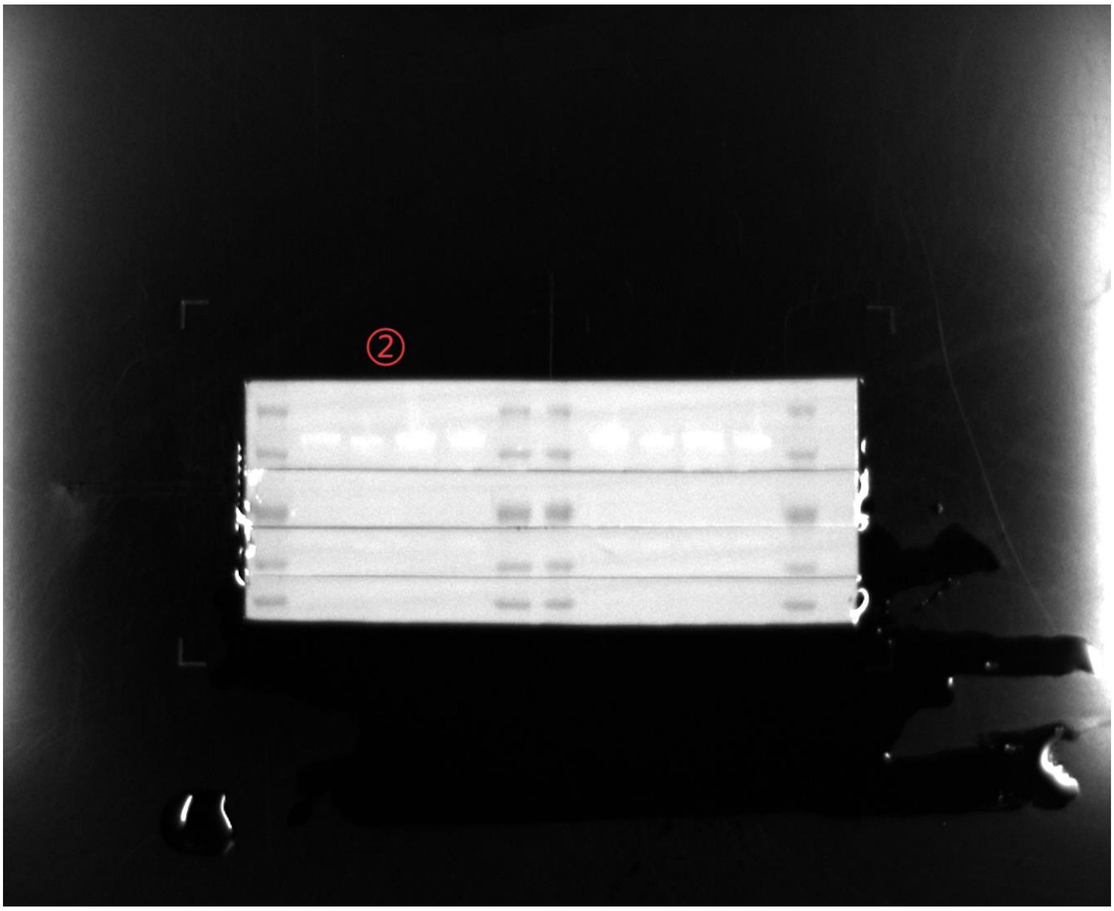
②

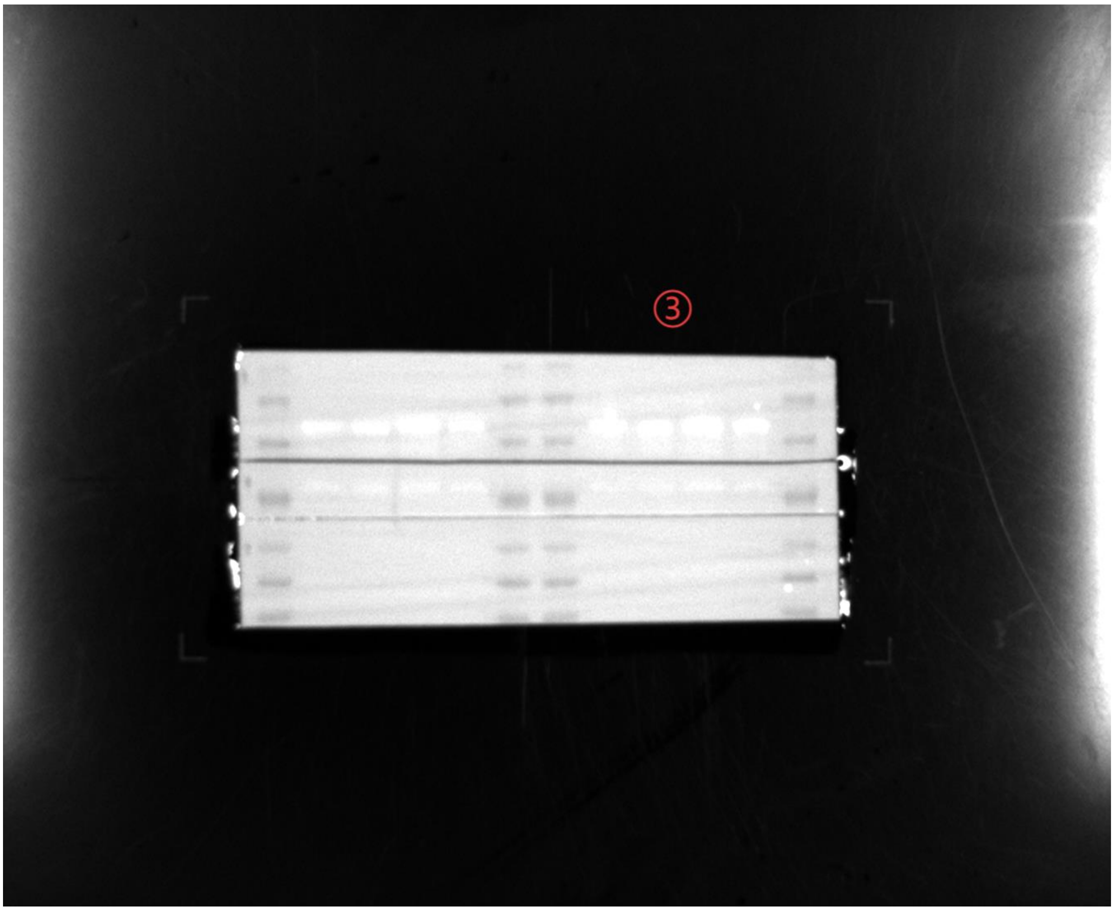
③

Weri-RB1(ARL5B)

The change in ARL5B protein expression was verified by Western blot using a 12.5% separating gel in the Weri-RB1 cells among the control group,SiRNA-ATF4 group,overexpression ARL5B group and SiRNA-ATF4 with overexpression ARL5B group .

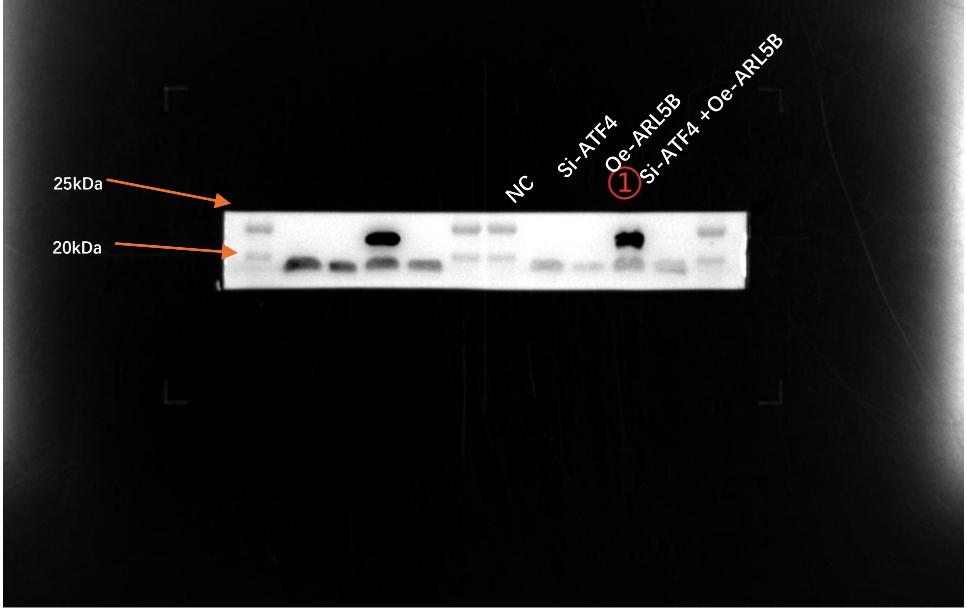

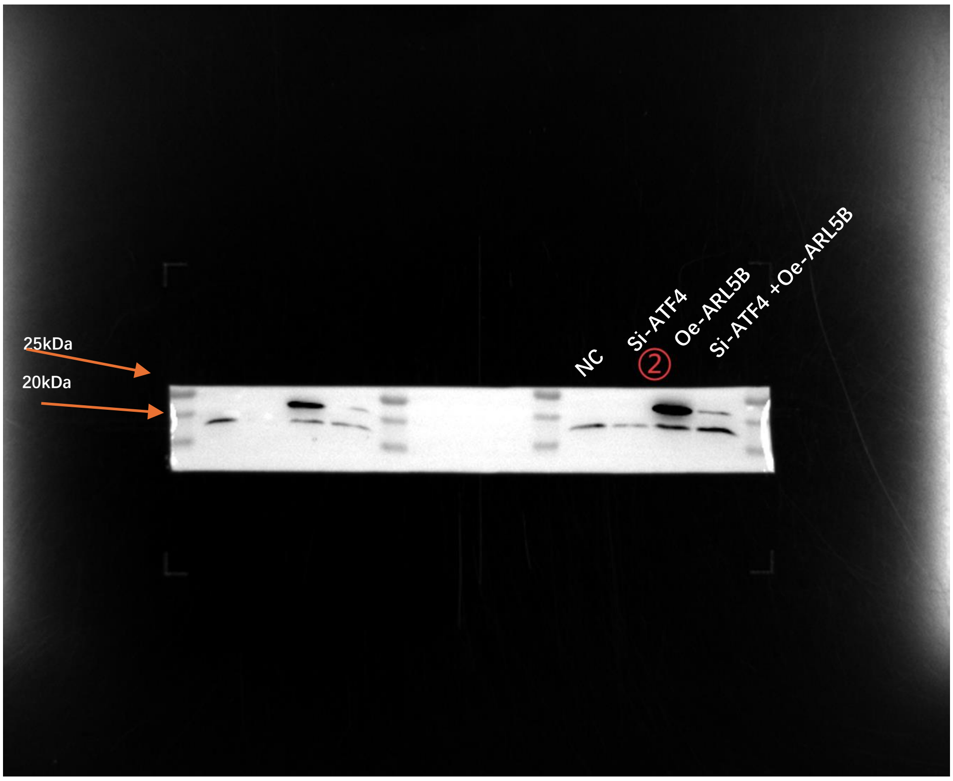

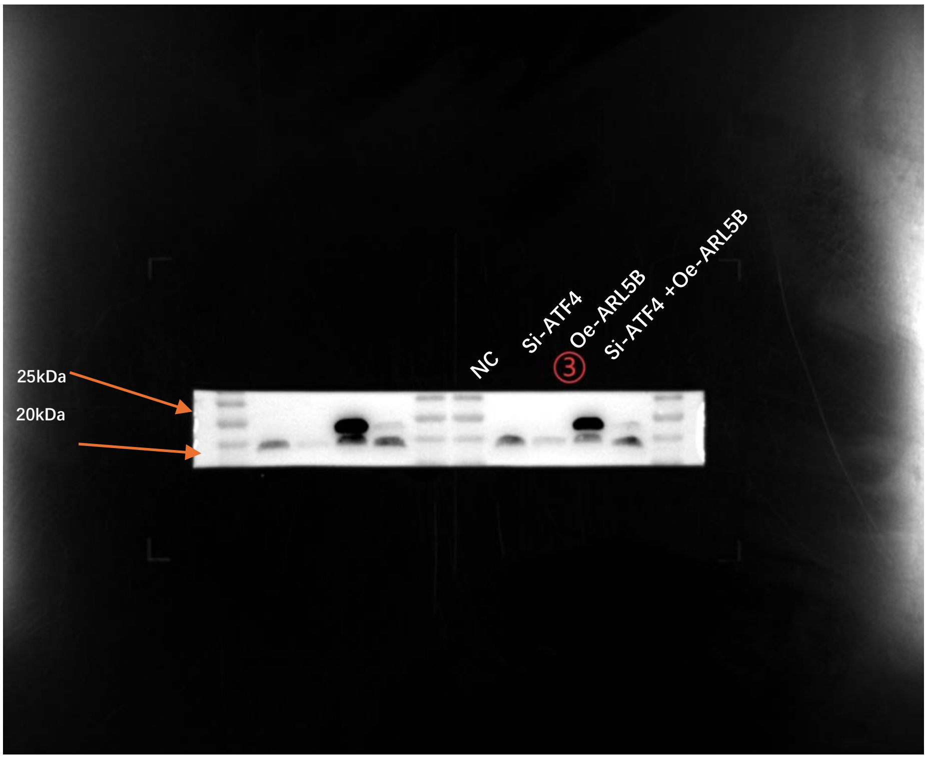

(β-Actin)

The change in β-Actin protein expression was verified by Western blot using a 12.5% separating gel in the Weri-RB1 cells among the control group,SiRNA-ATF4 group,overexpression ARL5B group and SiRNA-ATF4 with overexpression ARL5B group .

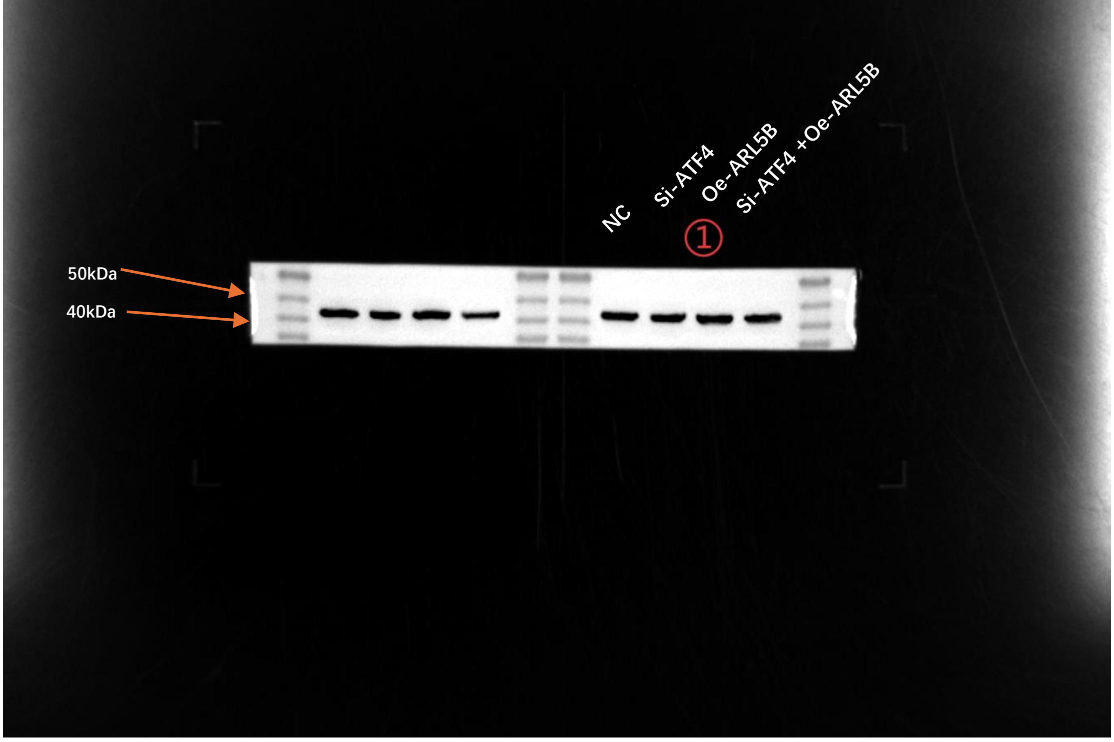

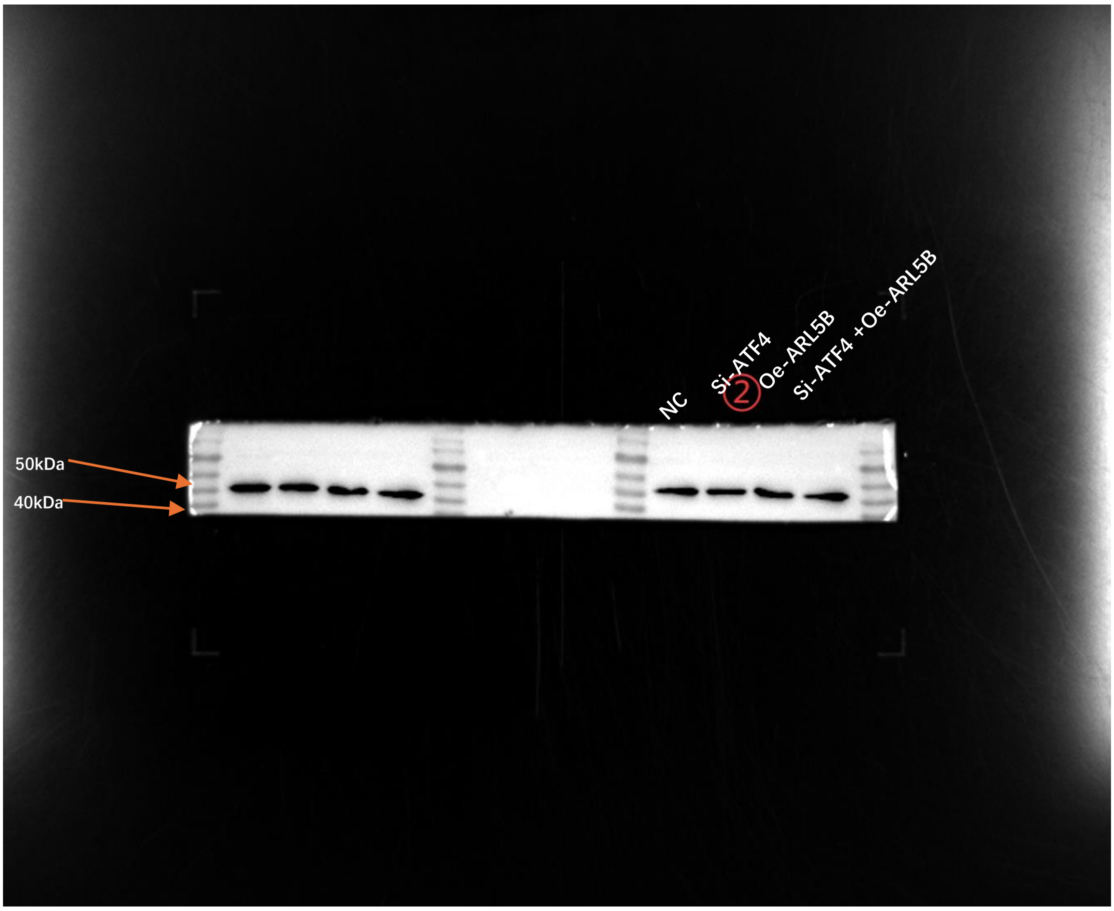

NC
Si-ATF4
2
Oe-ARL5B
Si-ATF4 +Oe-ARL5B
50kDa
40kDa

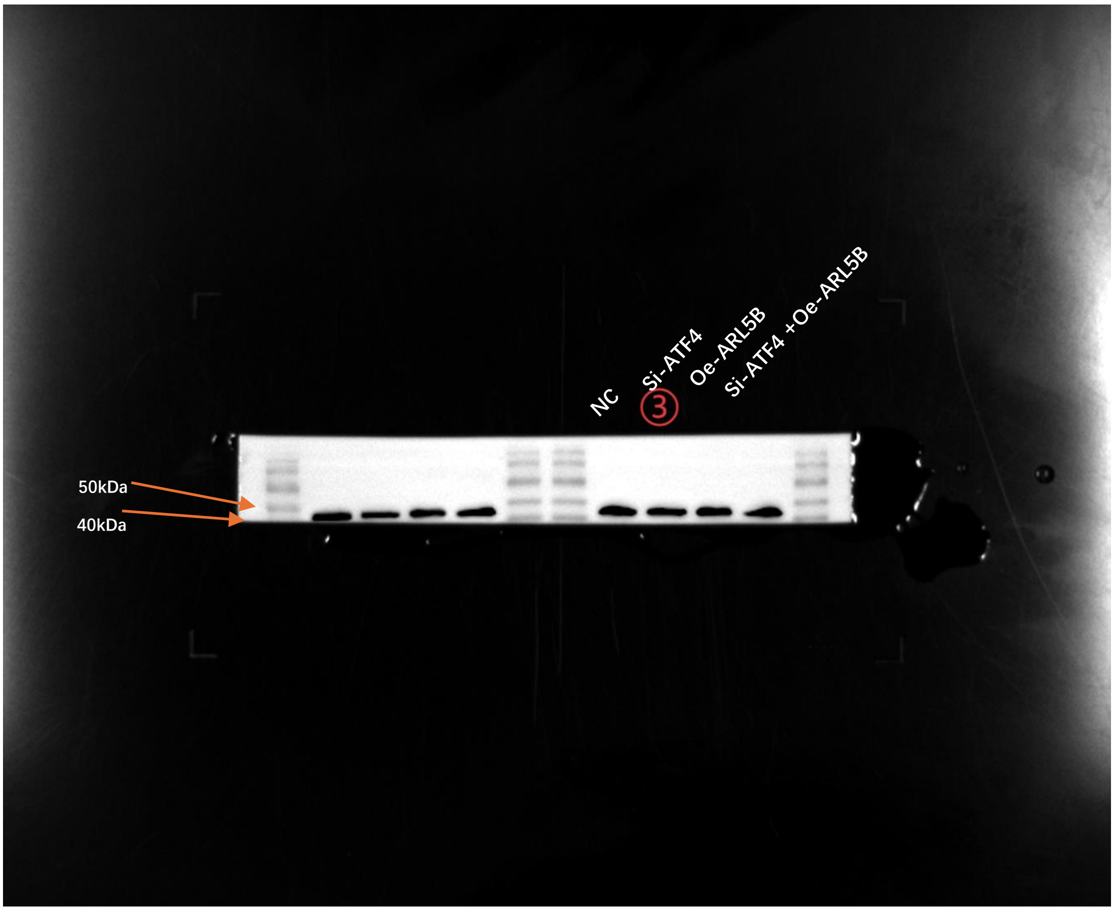

NC
Si-ATF4
3
Oe-ARL5B
Si-ATF4 +Oe-ARL5B
50kDa
40kDa

(MERGE)

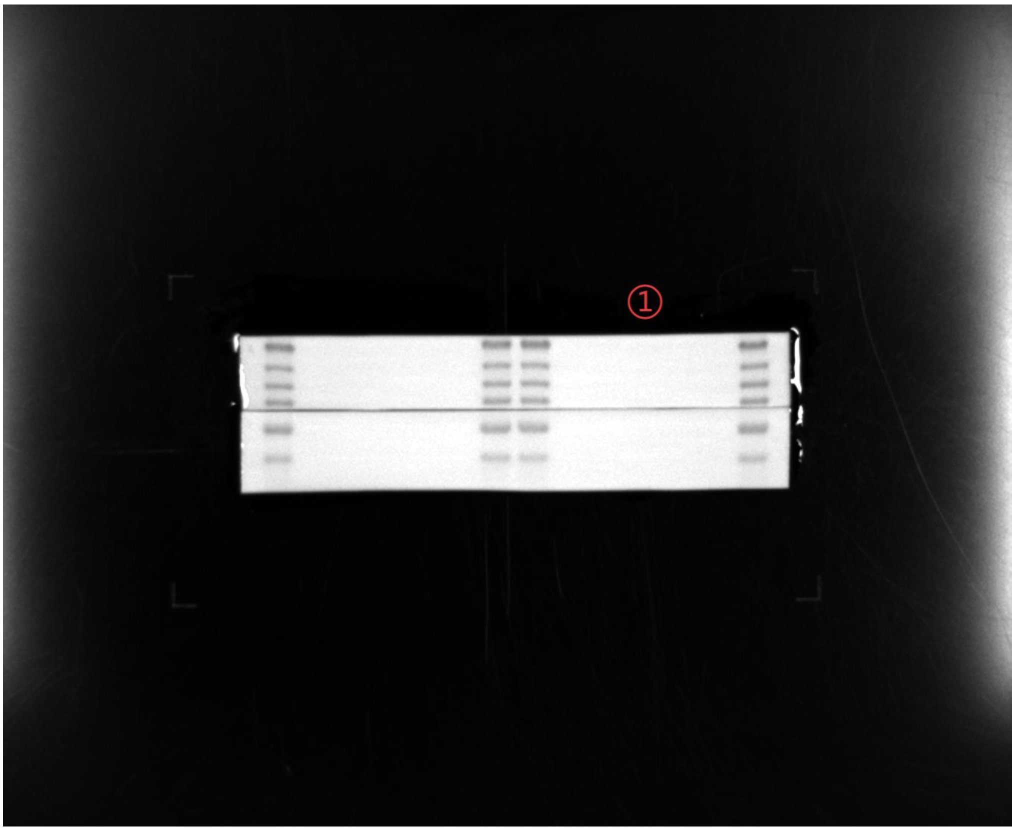

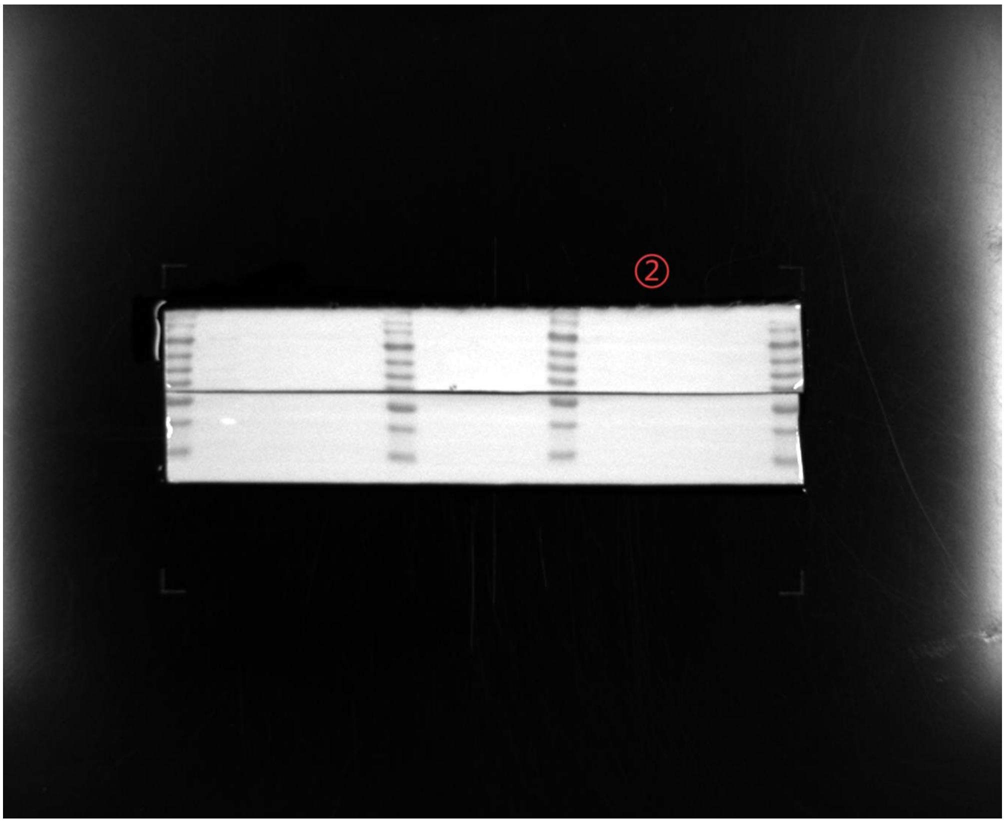

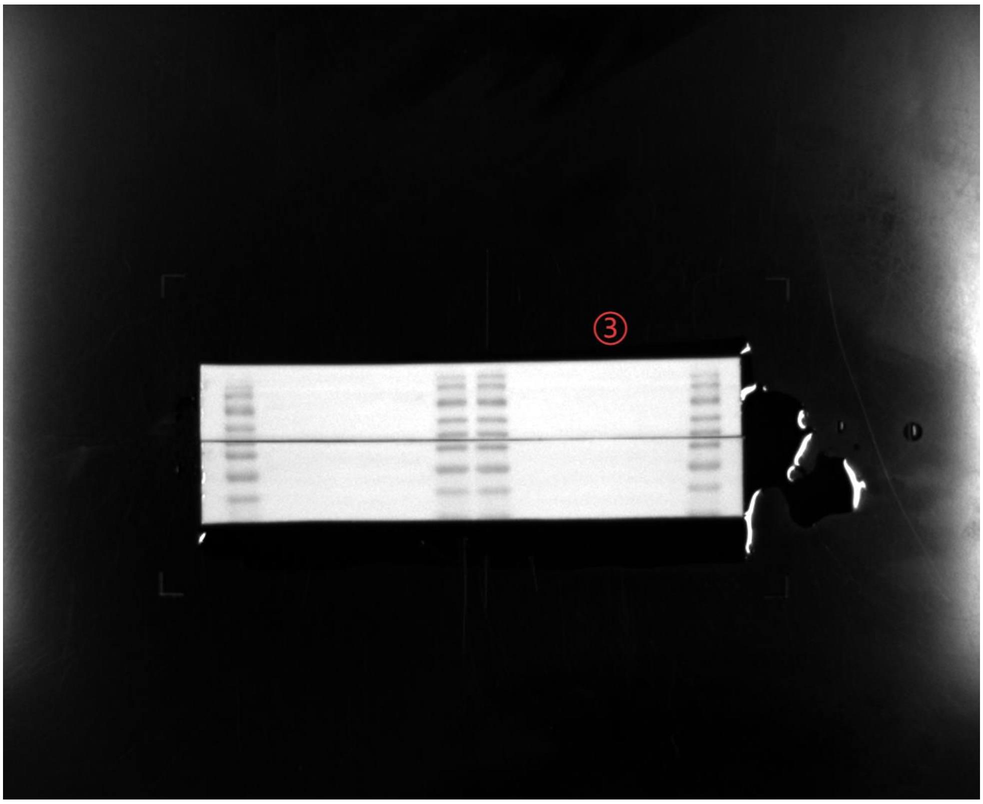

Weri-RB1(SKIP)

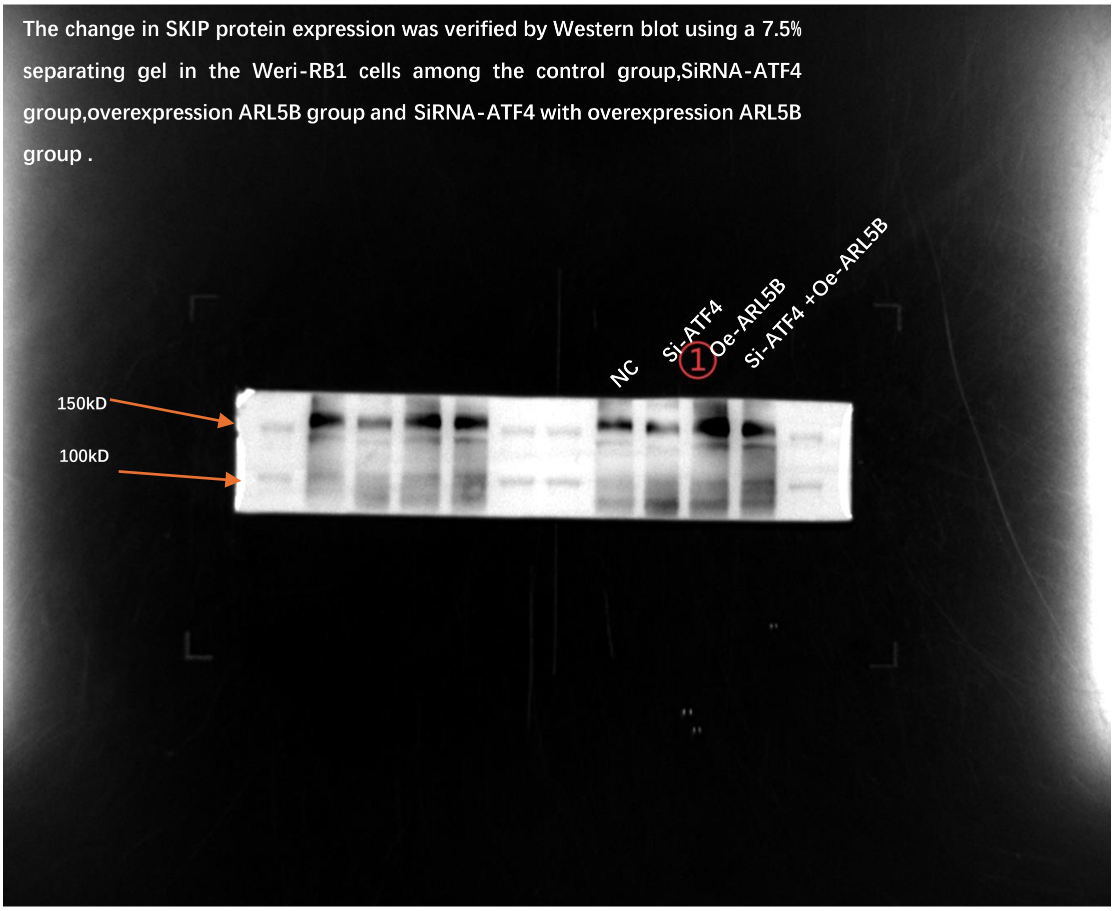

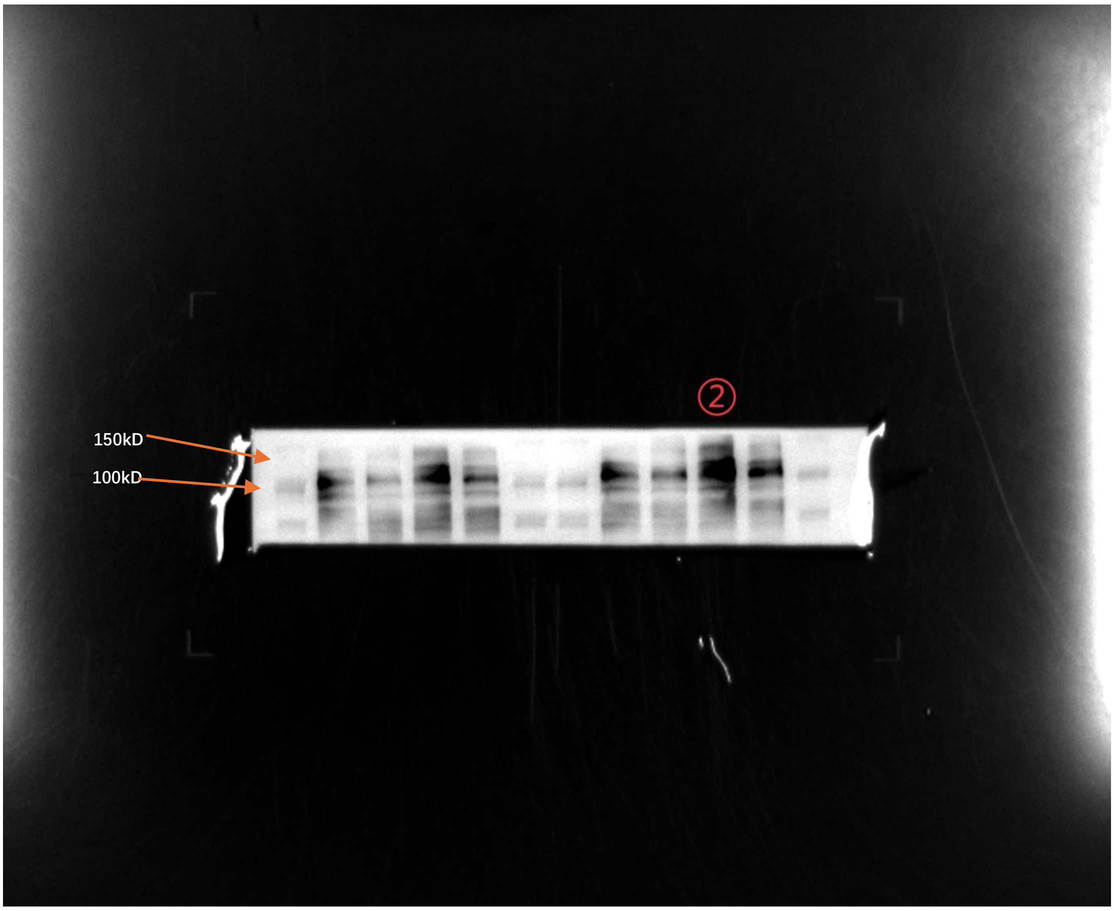
150kD
100kD
②

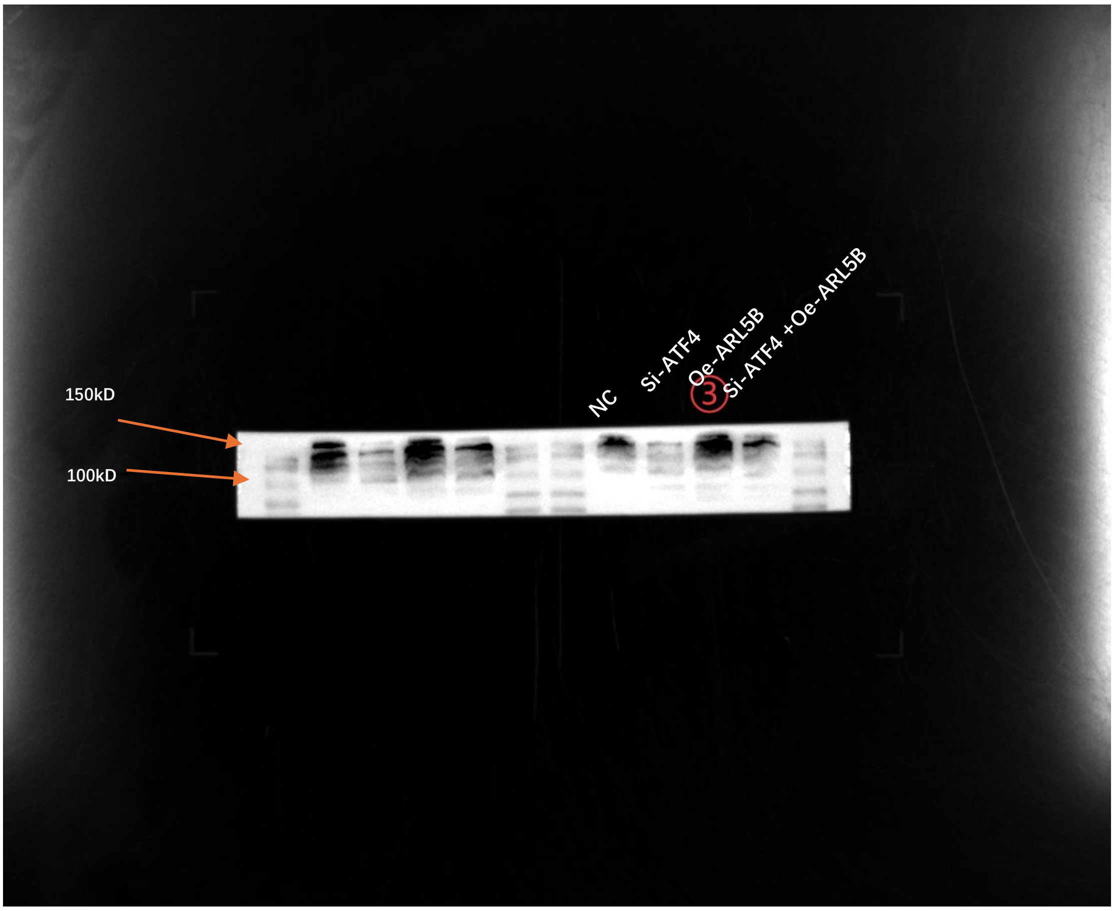
150kD
100kD
NC
Si-ATF4
Oe-ARL5B
Si-ATF4 +Oe-ARL5B
③

(β-Actin)

The change in β-Actin protein expression was verified by Western blot using a 7.5% separating gel in the Weri-RB1 cells among the control group,SiRNA-ATF4 group,overexpression ARL5B group and SiRNA-ATF4 with overexpression ARL5B group .

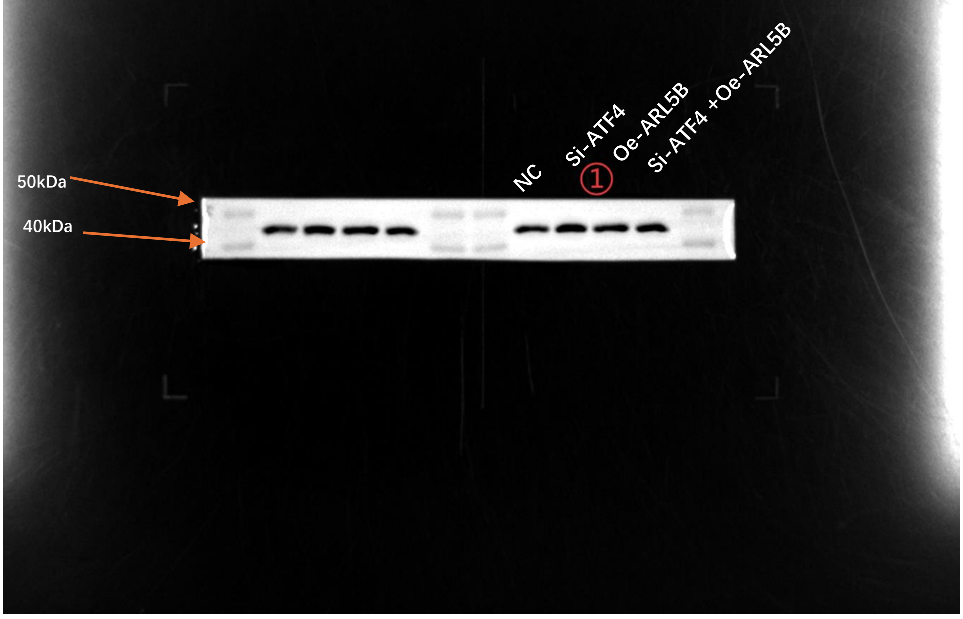

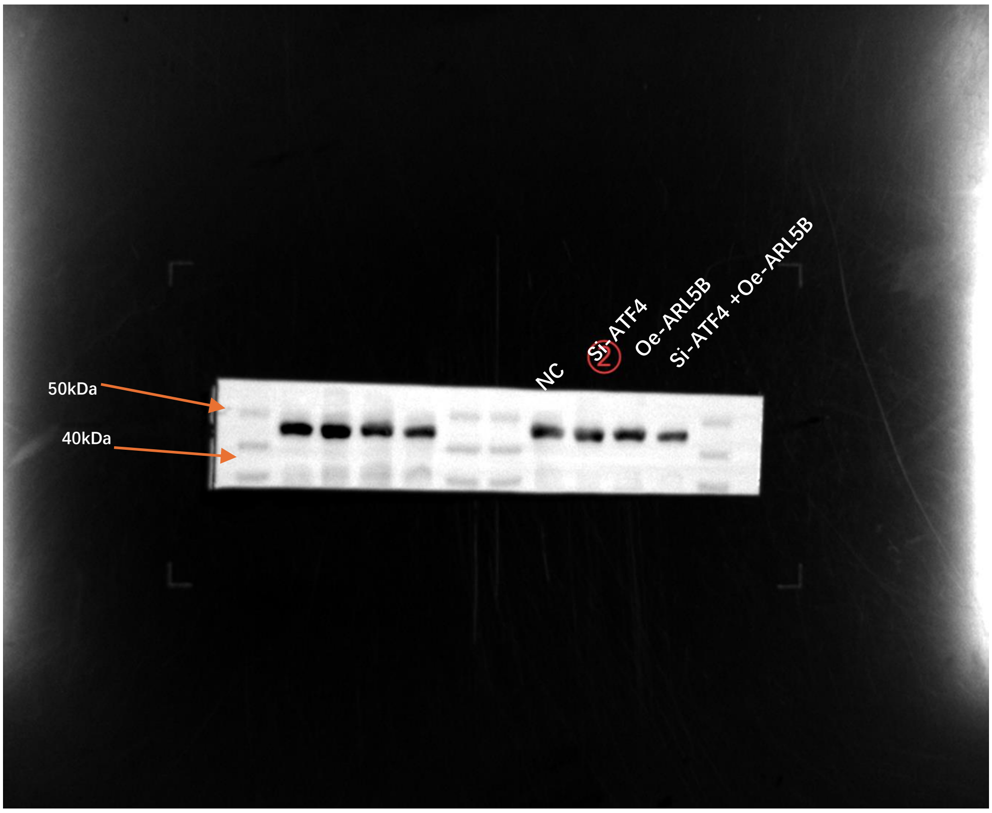

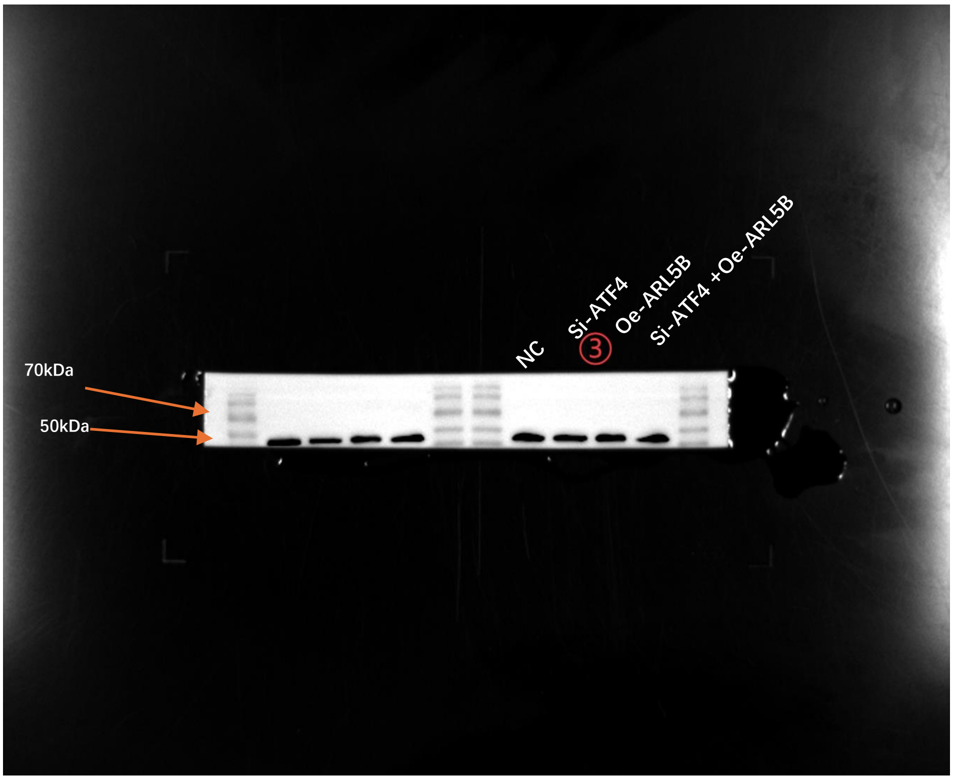

(MERGE)

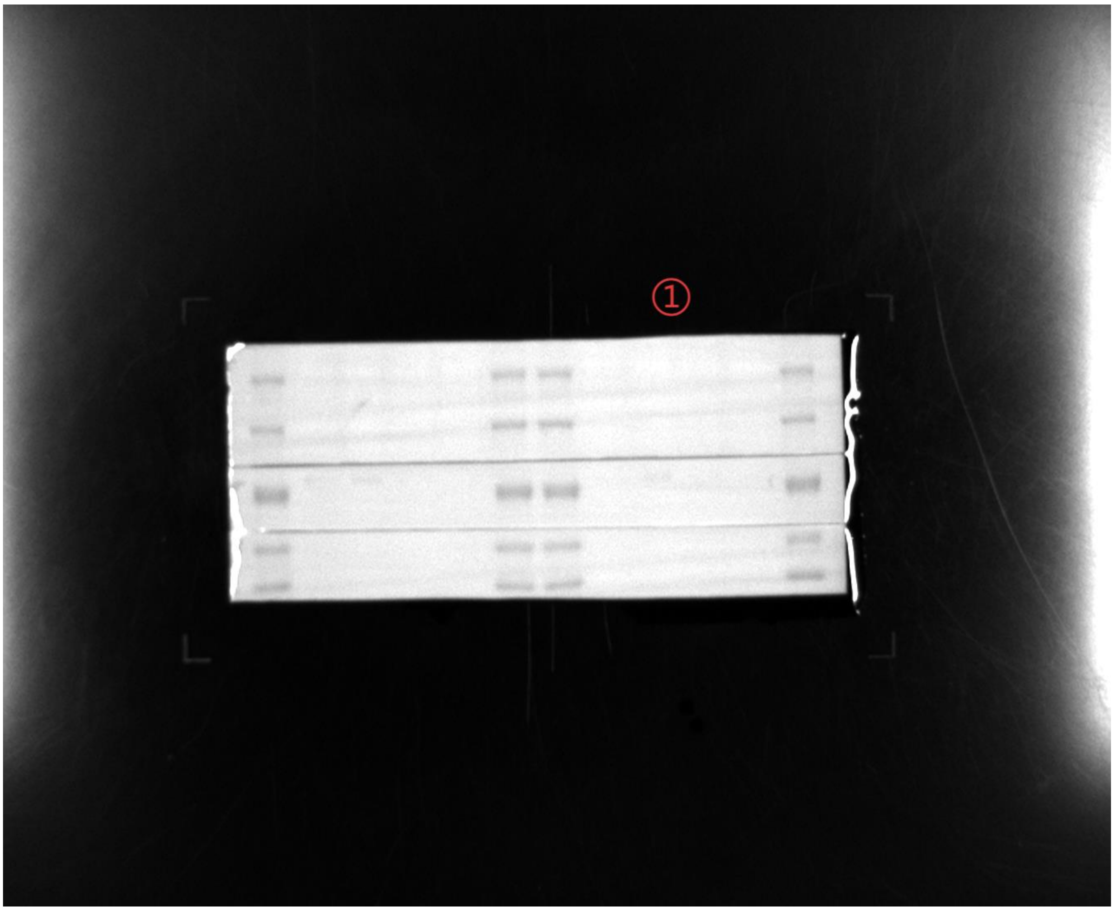

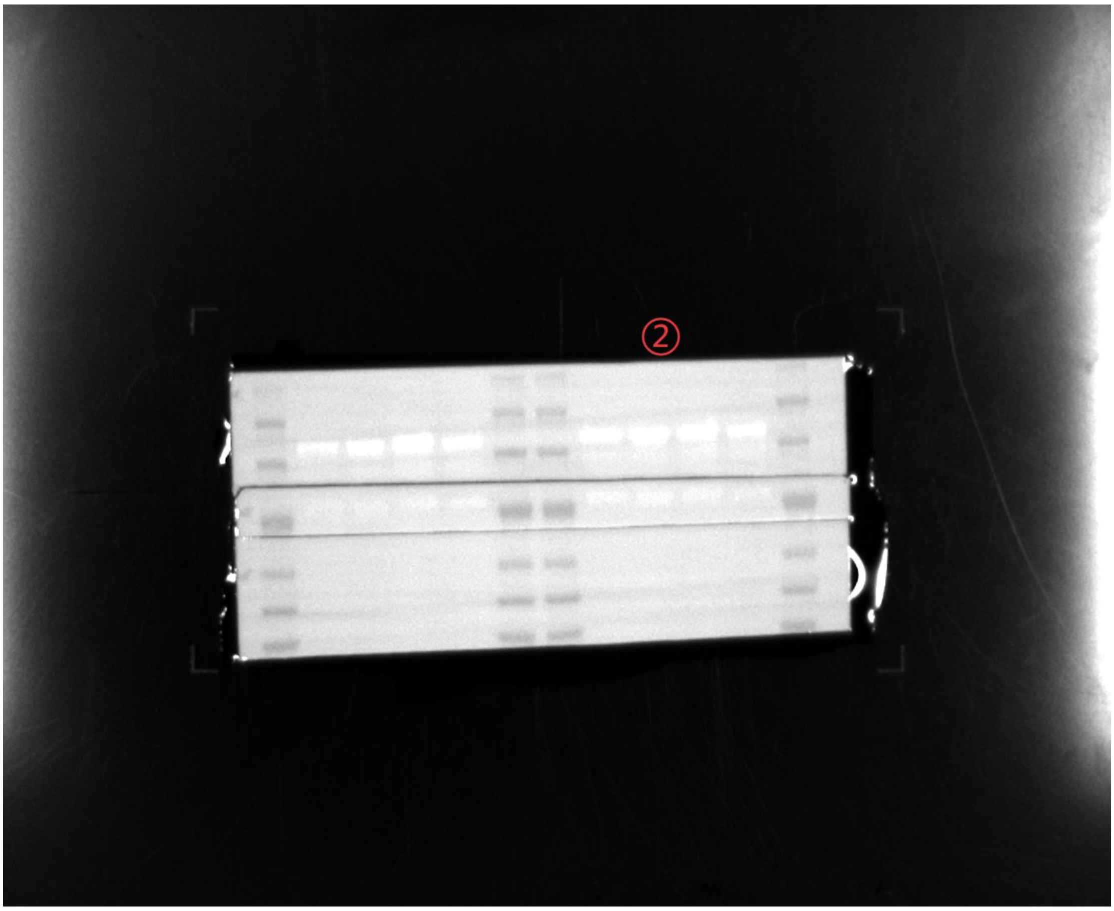
②

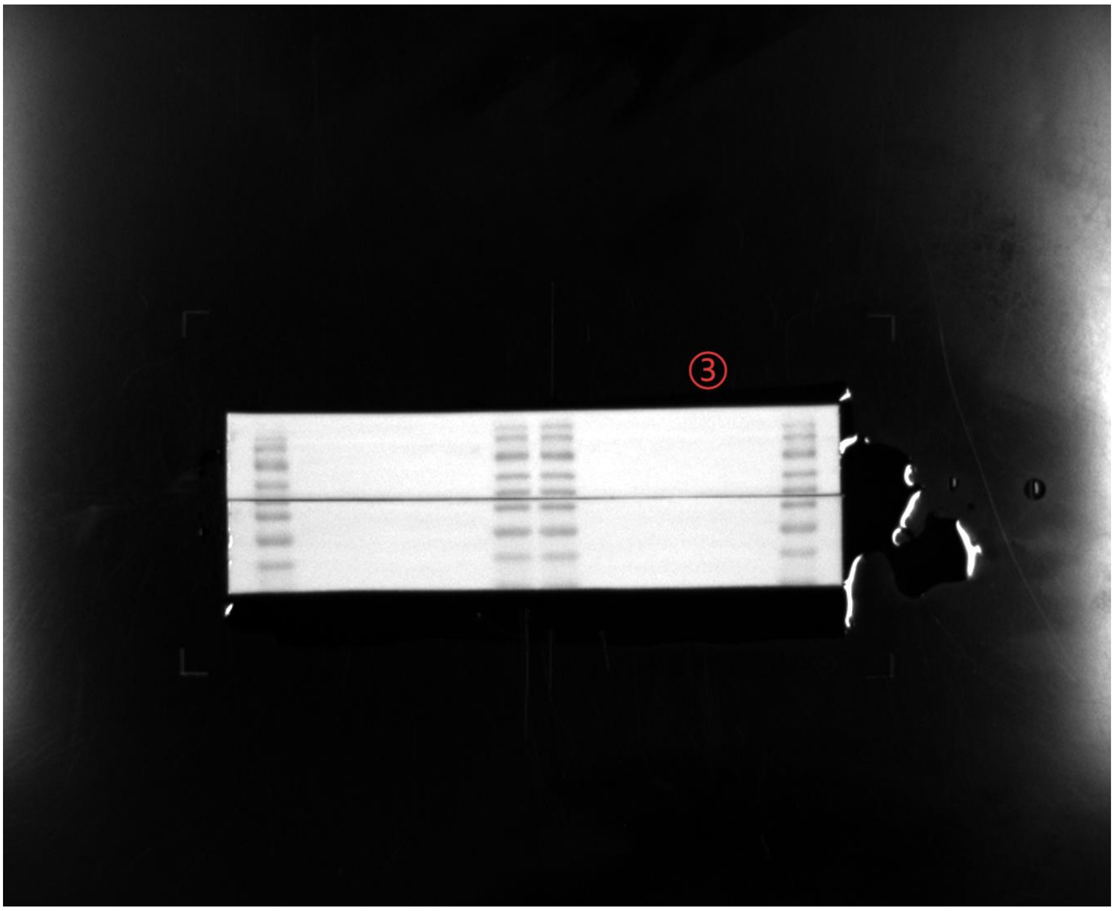
③

Weri-RB1(KIF5B)

The change in KIF5B protein expression was verified by Western blot using a 7.5% separating gel in the Weri-RB1 cells among the control group,SiRNA-ATF4 group,overexpression ARL5B group and SiRNA-ATF4 with overexpression ARL5B group .

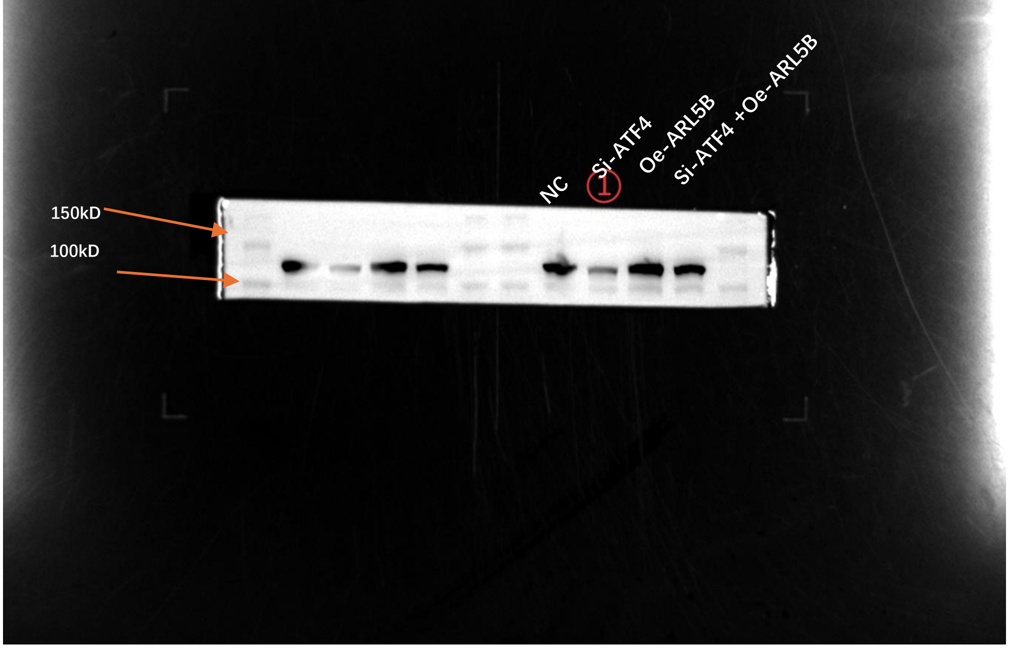

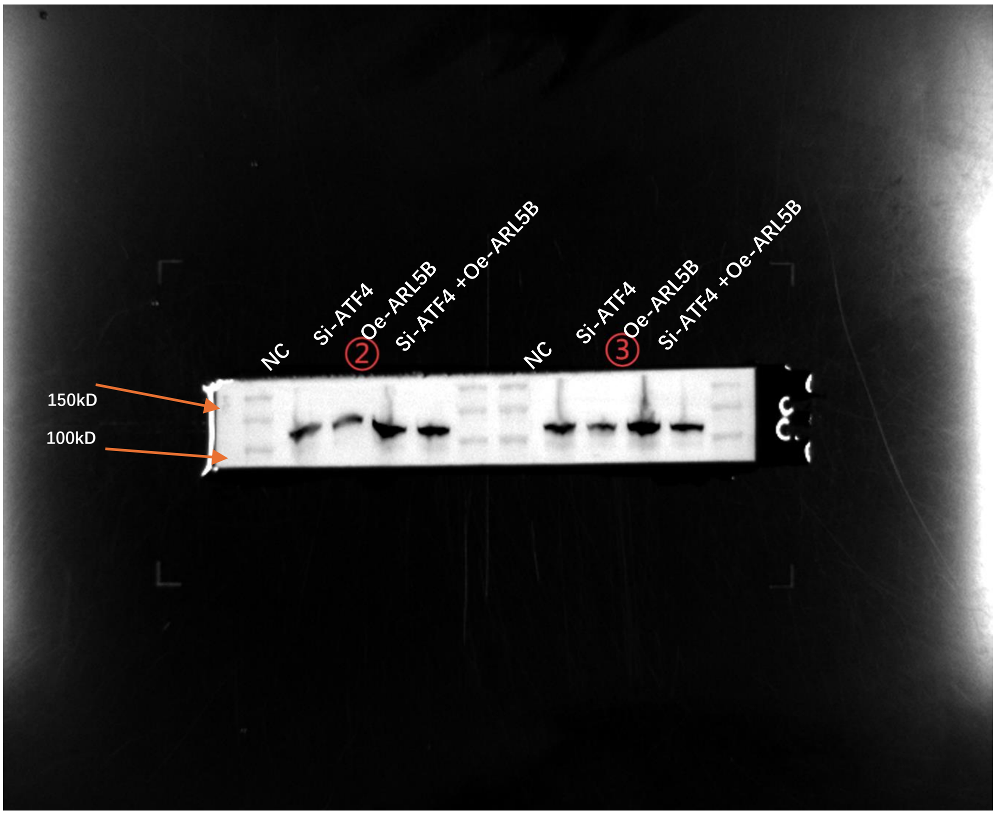

(β-Actin)

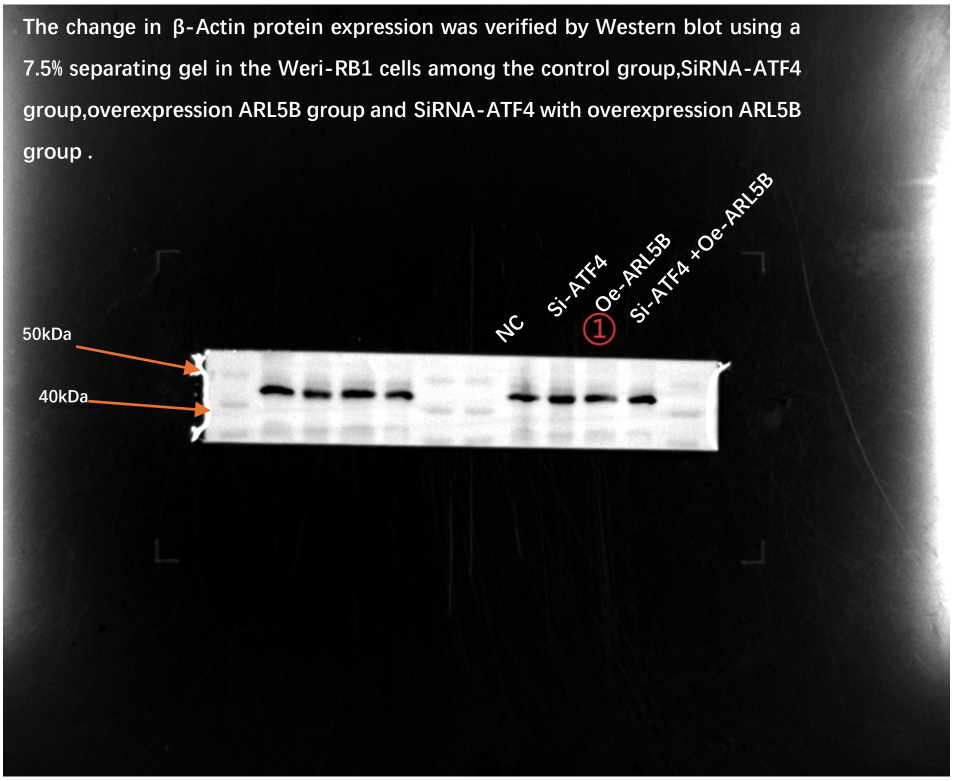
The change in β-Actin protein expression was verified by Western blot using a 7.5% separating gel in the Weri-RB1 cells among the control group,SiRNA-ATF4 group,overexpression ARL5B group and SiRNA-ATF4 with overexpression ARL5B group .
NC
Si-ATF4
Oe-ARL5B
Si-ATF4 +Oe-ARL5B
①
50kDa
40kDa

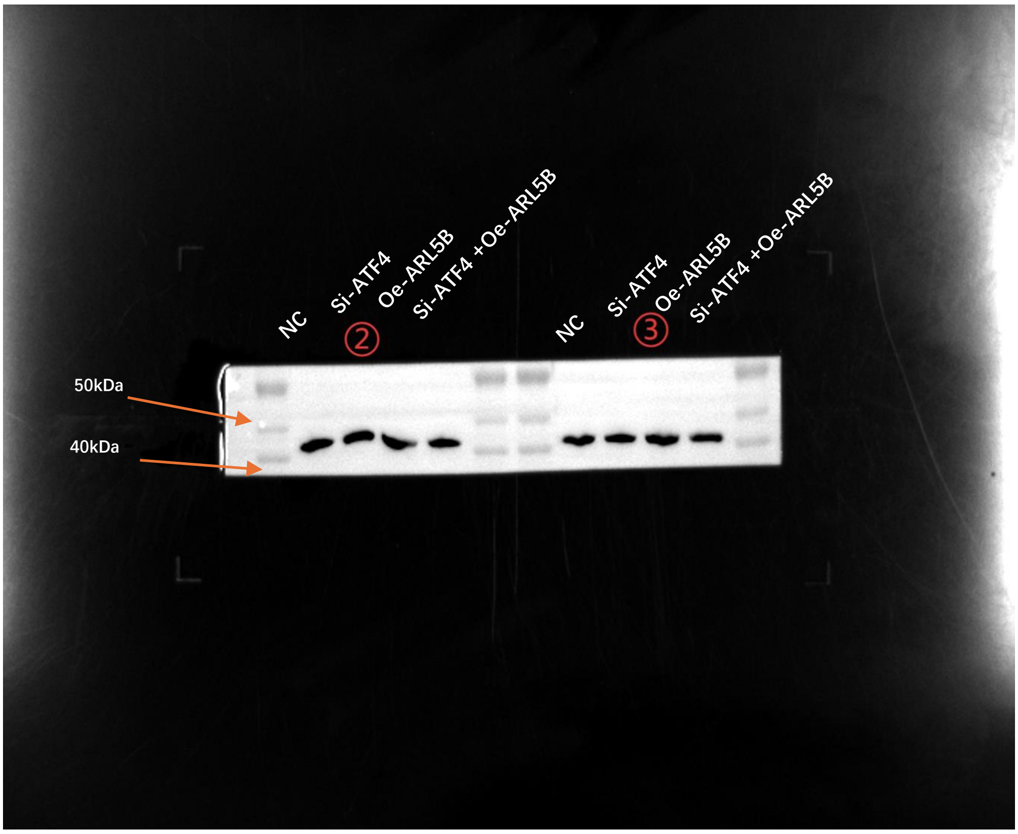
NC
Si-ATF4
Oe-ARL5B
Si-ATF4 +Oe-ARL5B
②
NC
Si-ATF4
Oe-ARL5B
Si-ATF4 +Oe-ARL5B
③
50kDa
40kDa

(MERGE)

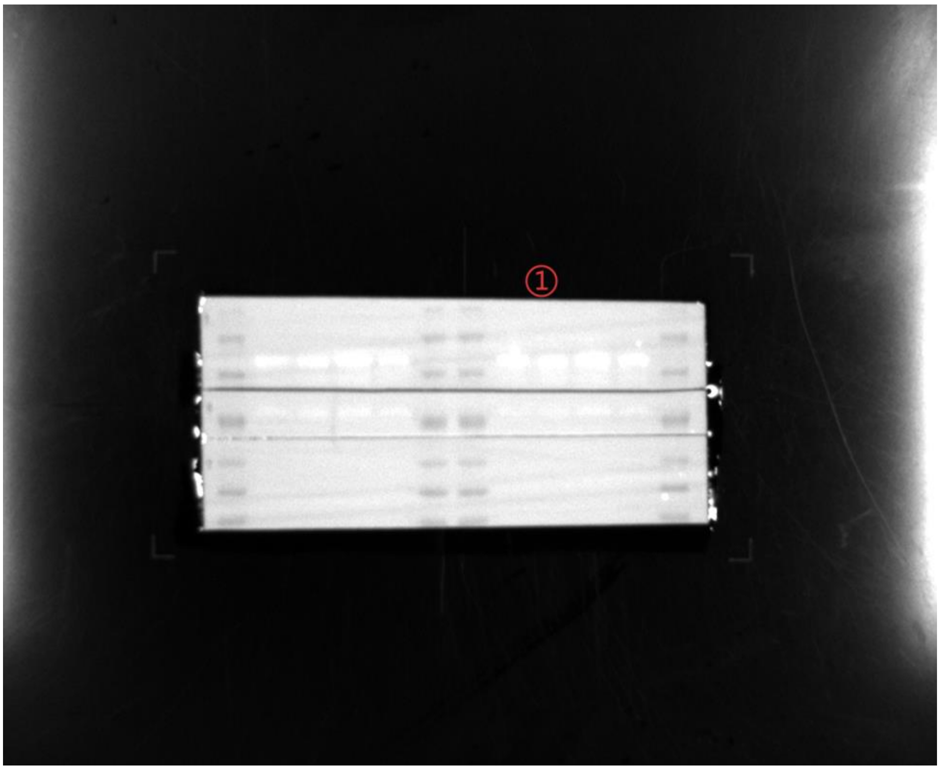

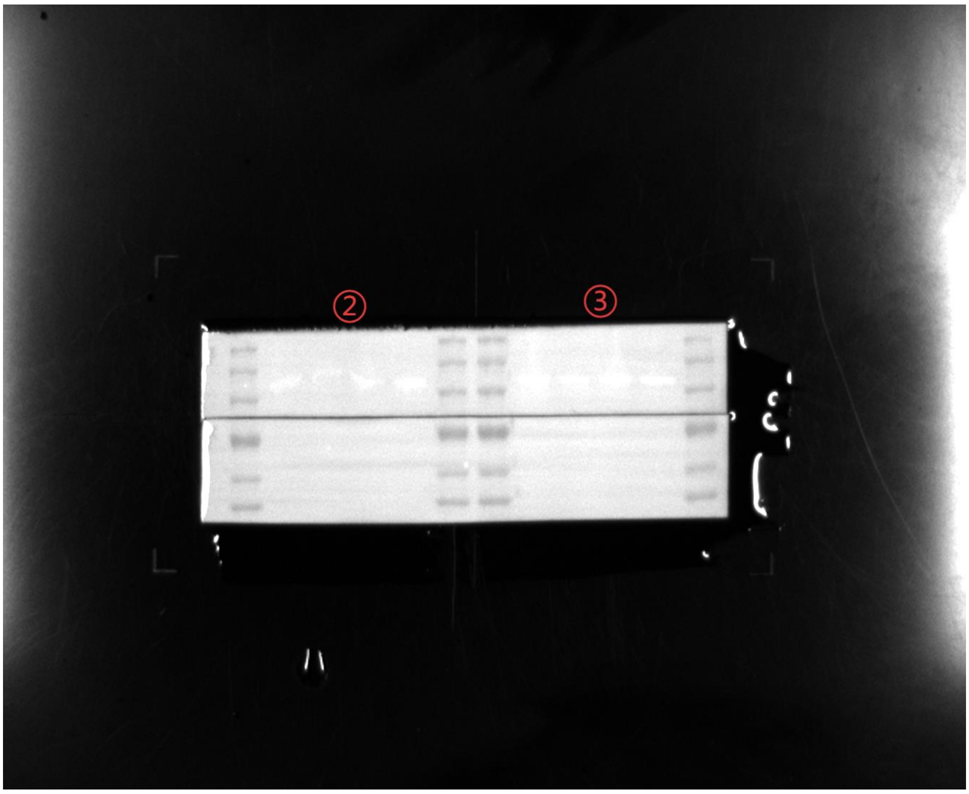

Weri-RB1(KLC2)

The change in KLC2 protein expression was verified by Western blot using a 10% separating gel in the Weri-RB1 cells among the control group,SiRNA-ATF4 group,overexpression ARL5B group and SiRNA-ATF4 with overexpression ARL5B group .

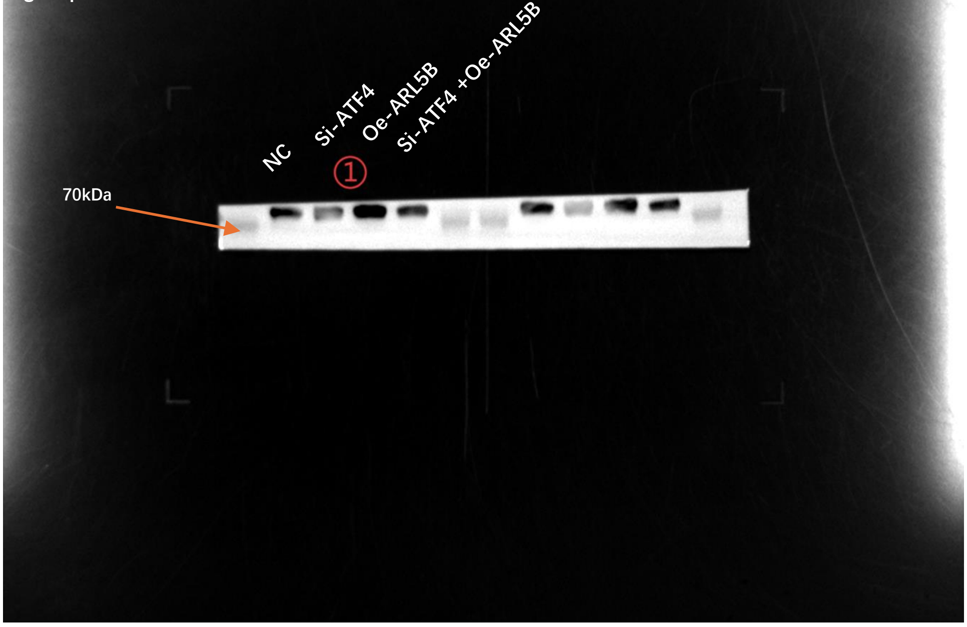

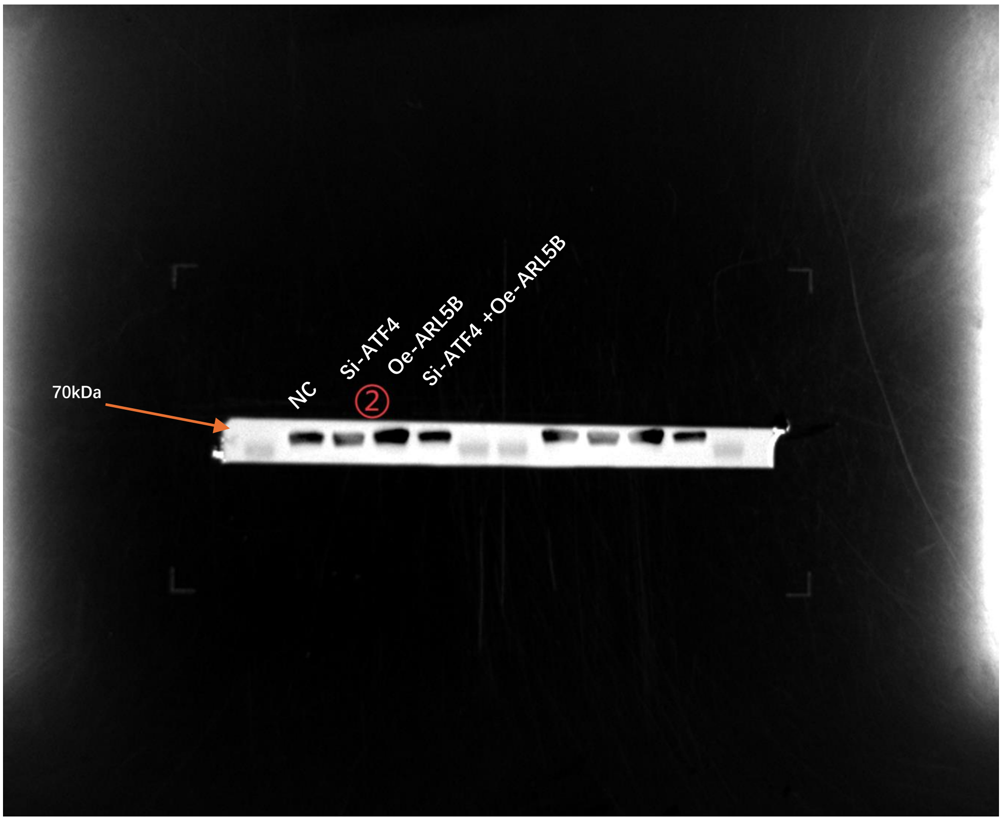

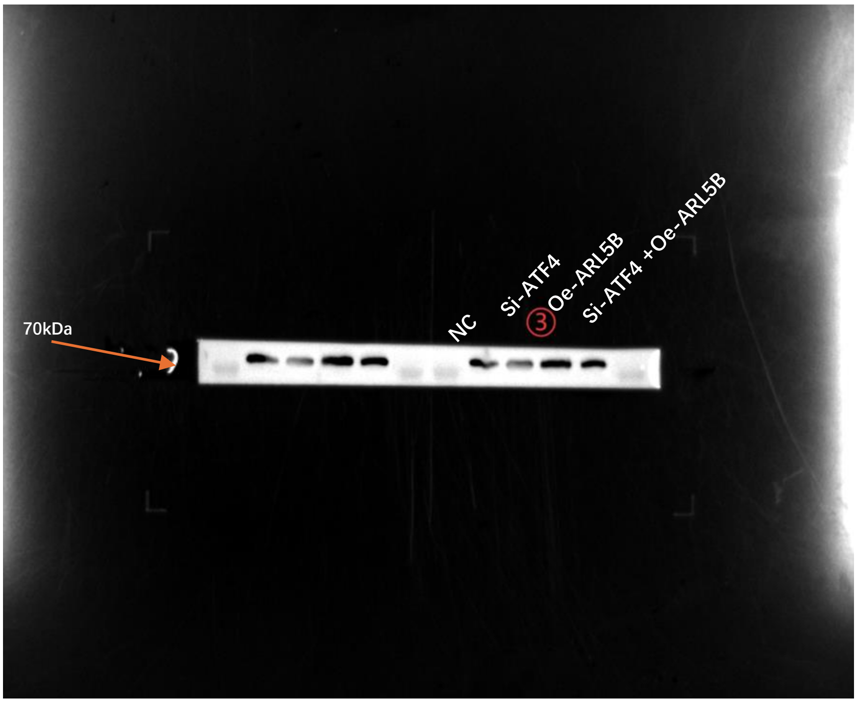

(β-Actin)

The change in β-Actin protein expression was verified by Western blot using a 10% separating gel in the Weri-RB1 cells among the control group,SiRNA-ATF4 group,overexpression ARL5B group and SiRNA-ATF4 with overexpression ARL5B group .

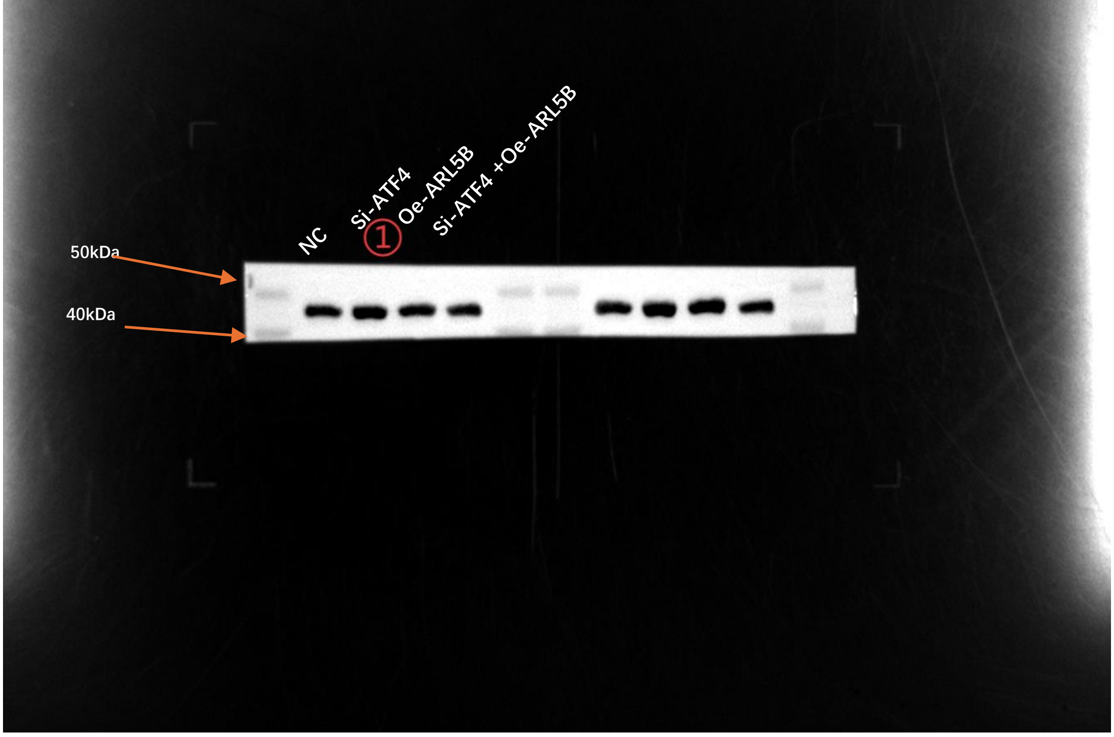

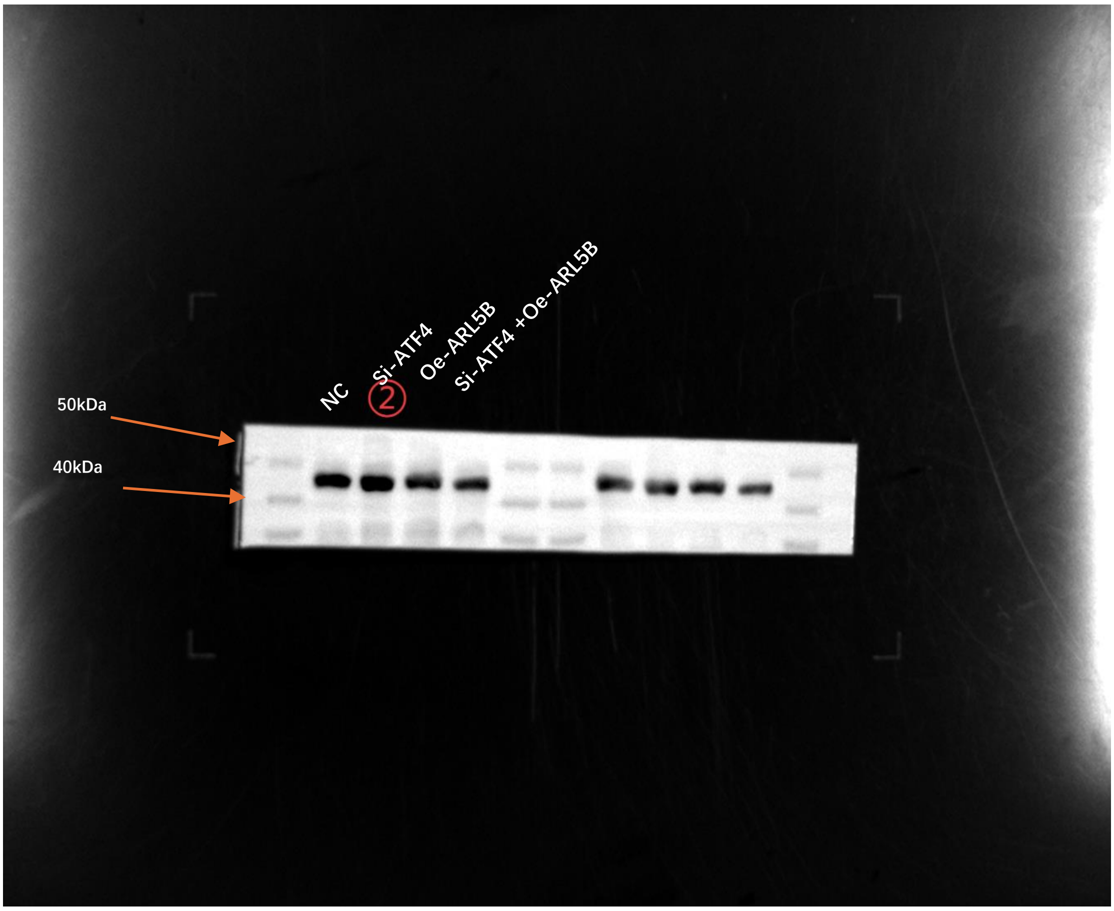

50kDa
40kDa
NC
Si-ATF4
②
Oe-ARL5B
Si-ATF4 +Oe-ARL5B

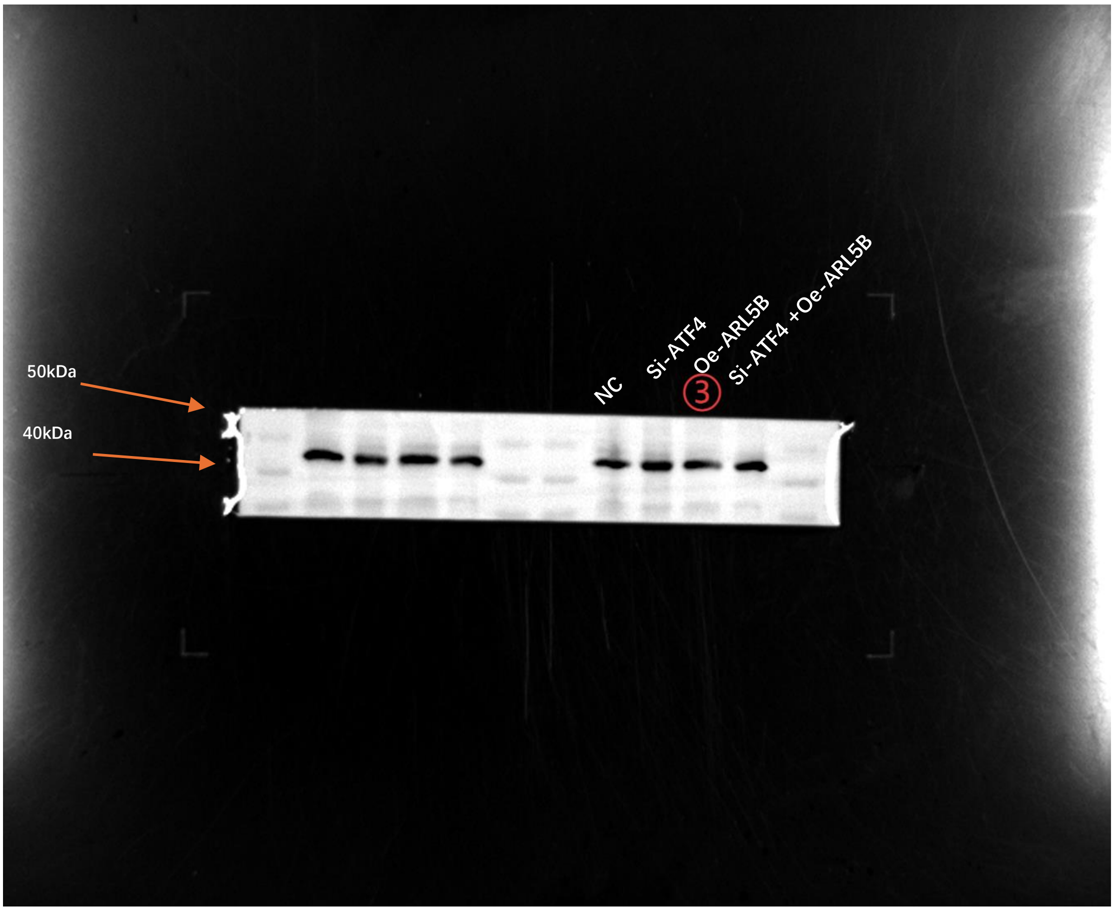

50kDa
40kDa
NC
Si-ATF4
Oe-ARL5B
③
Si-ATF4 +Oe-ARL5B

(MERGE)

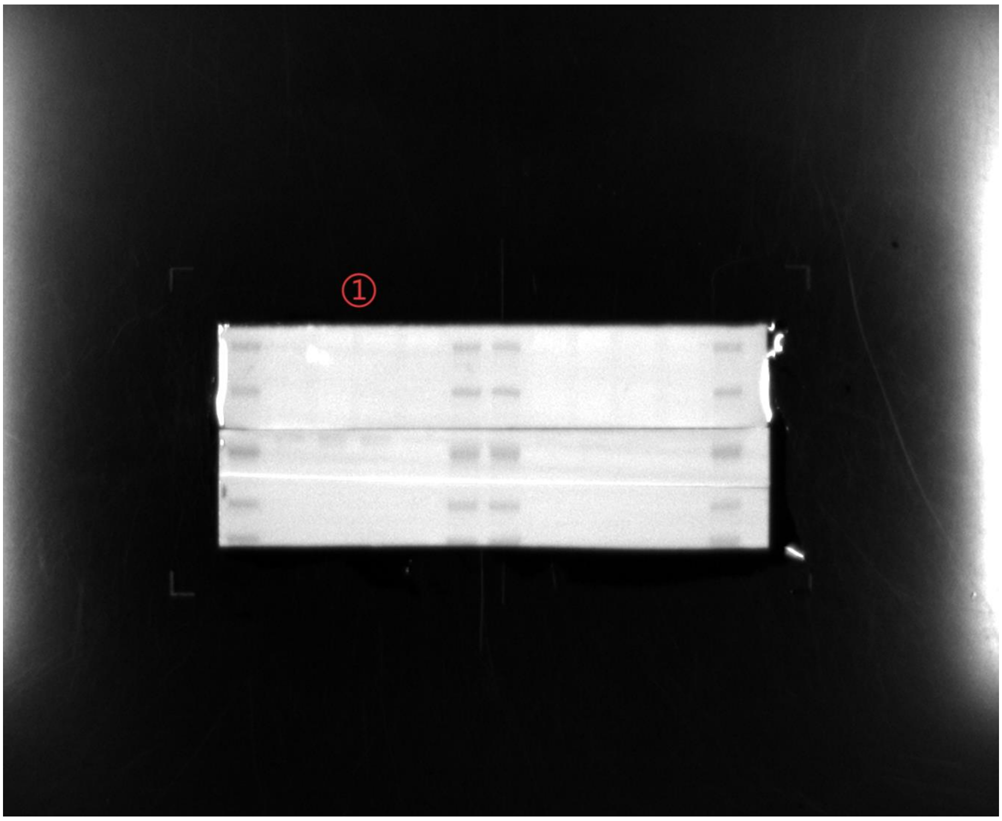

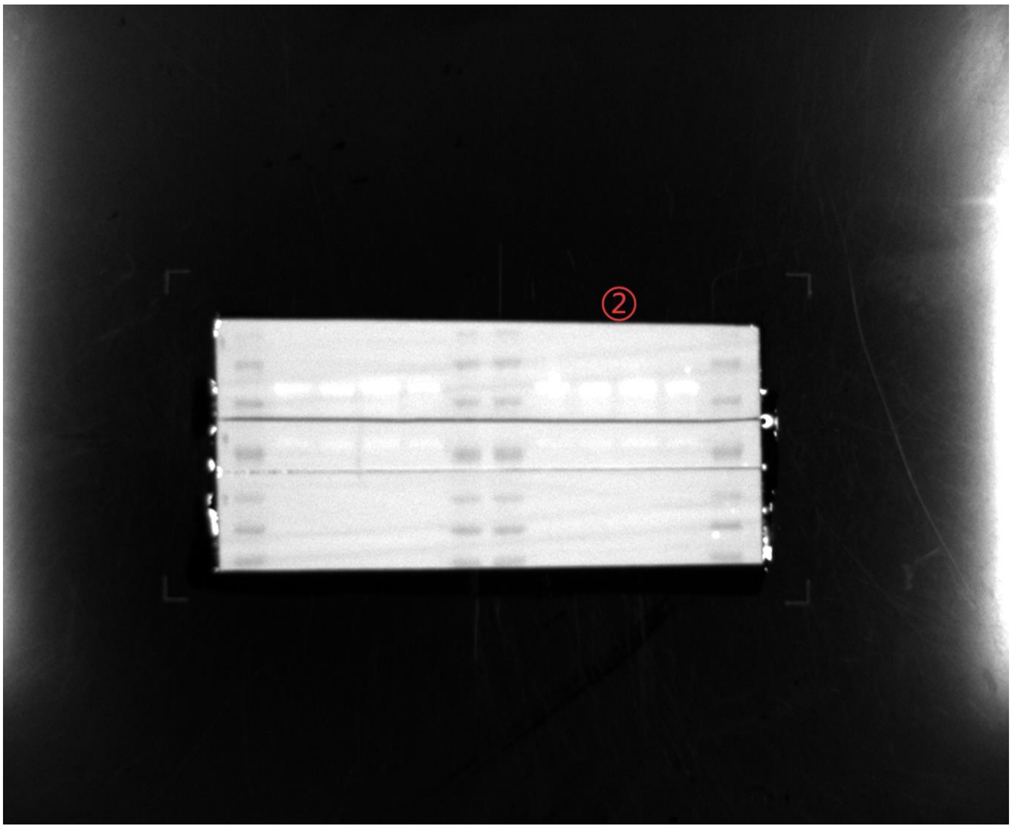

③

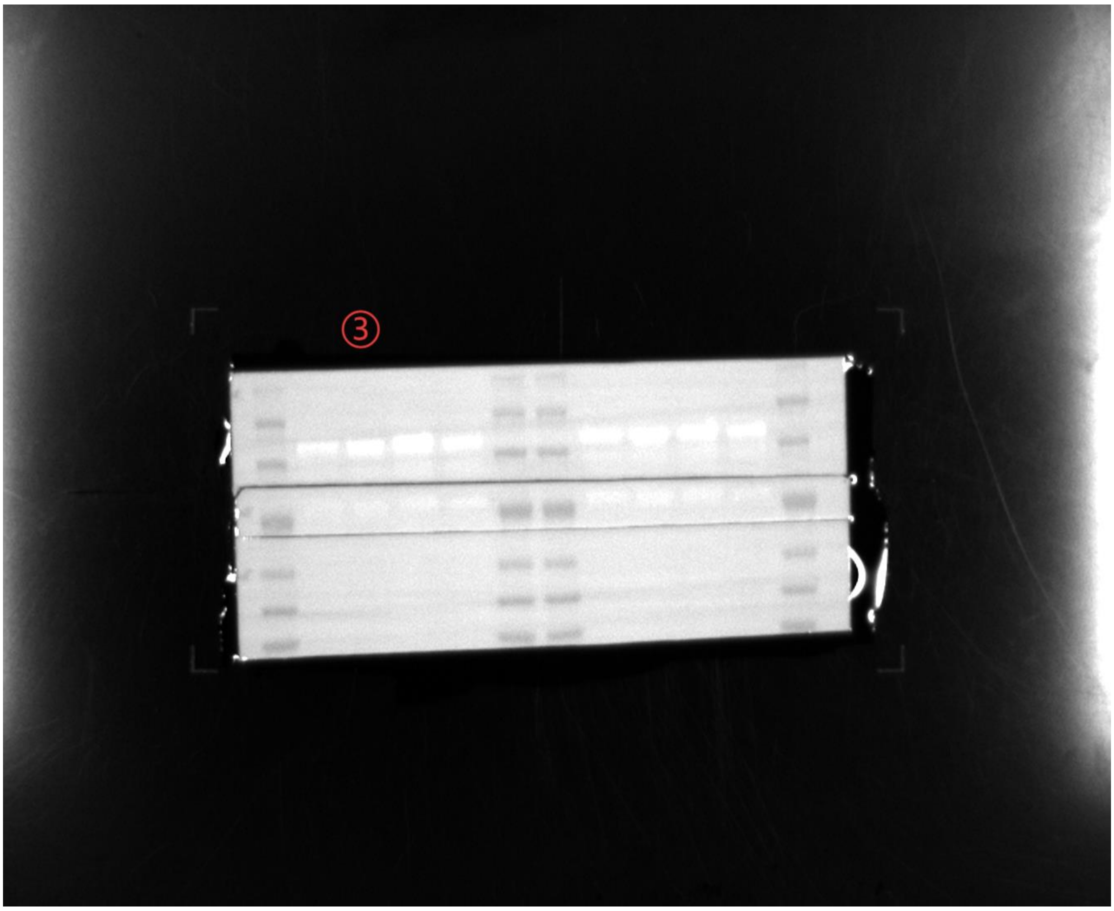

Supplement: Supplementary file 2 [file DataSheet1.pdf]
